# Identification of iron metabolism-related key genes and exploration of their potential mechanisms in hemophagocytic lymphohistiocytosis based on transcriptome sequencing

**DOI:** 10.3389/fmed.2025.1685793

**Published:** 2026-01-14

**Authors:** Li Fen, Duan Hua Nan, Yu Lin Na, Zhao Jie, Gu Xue Zhong

**Affiliations:** 1 Hematology Department of Yunnan First People’s Hospital, Kunming University of Science and Technology Affiliated Hospital, Yunnan Province Clinical Research Center for Hematologic Disease, Hu Yu Expert Workstation, Yunnan Province Major Difficult Diseases Traditional Chinese and Western Medicine Clinical Collaboration Pilot Project - Leukemia, Yunnan Provincial Clinical Medical Center for Blood Diseases and Thrombosis Prevention and Treatment, Yunnan Provincial Atherosclerosis Traditional Chinese and Western Medicine Collaborative Collaboration Base, Kunming, China; 2 Yunnan Provincial Clinical Medical Center for Digestive Diseases/Gastroenterology, The First People's Hospital of Yunnan Province, Kunming, China

**Keywords:** hemophagocytic lymphohistiocytosis, iron metabolism, immune microenvironment, key genes, transcriptomics

## Abstract

**Background:**

Hemophagocytic lymphohistiocytosis (HLH) is a severe inflammatory disorder caused by excessive immune activation. This exploratory study aimed to systematically identify potential diagnostic key genes linked to iron metabolism during HLH progression using comprehensive bioinformatics analysis and explore the underlying mechanisms.

**Methods:**

Thirty peripheral blood mononuclear cell (PBMC) samples—15 from patients with HLH and 15 controls—were analyzed through mRNA sequencing. Total RNA was extracted from these samples. First, the overlap between differentially expressed genes (DEGs) and iron metabolism-related genes (FeRGs) was identified to select candidate genes. Key genes were then derived using the Boruta method and least absolute shrinkage and selection operator (LASSO) regression analysis. Further investigations, including correlation, functional, and enrichment analyses, as well as immune infiltration and drug prediction, were performed to explore the molecular mechanisms. Finally, reverse transcription quantitative PCR (RT-qPCR) was used to validate the expression levels of the key genes in clinical specimens.

**Results:**

The intersection of 464 DEGs and 520 FeRGs identified 10 candidate genes. Machine learning analysis identified ALOX15, CAT, HBZ, MT2A, and CYGB as HLH key genes. A significant positive correlation was observed between ALOX15 and HBZ, while ALOX15 showed a notable negative correlation with CYGB and MT2A (|correlation coefficient (r)| > 0.3, *p* < 0.05). These key genes were functionally associated with antioxidant activity, toxic substance response, and inorganic compound detoxification, primarily involved in oxidative phosphorylation and Huntington’s disease-related pathways. Notably, ALOX15 and CAT were significantly correlated with activated dendritic cells and CD4 memory T cells. Drug prediction revealed 15 compounds targeting ALOX15, MT2A, and CAT. Expression of ALOX15, CAT, and HBZ was down-regulated, while MT2A and CYGB were up-regulated in the HLH group, consistent with differential expression trends.

**Conclusion:**

This study identified five validated key genes—ALOX15, CAT, HBZ, MT2A, and CYGB—offering new insights into HLH pathogenesis and potential therapeutic targets.

## 1 Introduction

Hemophagocytic syndrome, also known as hemophagocytic lymphohistiocytosis (HLH), is a life-threatening inflammatory condition driven by an overactive immune response that can severely impair organ function (1). Clinically, HLH is characterized by persistent fever, elevated ferritin levels, splenomegaly, cytopenias, and coagulopathy (2, 3). Liver involvement is especially prevalent, typically manifesting as liver injury. HLH may be triggered by genetic defects or acquired risk factors, including infections (e.g., viral), autoimmune diseases, and malignancies (such as lymphoma) (4). Mortality rates range from 20 to 88%, with treatment outcomes generally poor (5, 6). Current therapeutic approaches primarily focus on induction therapy to manage cytokine storm symptoms and etiological treatment, yet the mortality rate remains around 50% (7). Although targeted anti-inflammatory agents have shown promise in recent years, their effectiveness is limited due to the narrow scope of single-target therapies, which fail to address the multifactorial nature of HLH, as well as the infection risks associated with agents like IFN-*γ* antibodies (8, 9). Given the incomplete understanding of HLH pathogenesis, exploring its molecular characteristics is of critical scientific and clinical importance, as it could lead to the identification of novel therapeutic targets and improve clinical outcomes.

A key feature of HLH is life-threatening hyperferritinemia, with ferritin playing a pivotal role in sequestering free iron to prevent the generation of free radicals, while serving as the primary form of iron storage (10, 11). Ferritin is primarily localized in the cytoplasm of hepatocytes and macrophages, with smaller amounts present in serum (12). Iron metabolism in the cytoplasm is involved in several vital processes, including erythropoiesis, nuclear translocation to protect genetic material from reactive oxygen species (ROS), and extracellular release to form serum ferritin, which is then internalized by cells (13). During infection and inflammation, iron is redistributed to hepatocytes and macrophages, leading to intracellular iron overload. When these storage cells are damaged, ferritin is released into the bloodstream in large quantities, creating a vicious cycle of “cellular iron overload → cellular damage → serum ferritin elevation” (14–18). Although hemochromatosis is a key feature of HLH (10, 11), the systemic dysregulation of iron metabolism-related genes and their immune regulatory roles remains a research blind spot to this day. Therefore, an in-depth investigation of the intricate relationship between HLH and iron metabolism is essential for accurate diagnosis and the development of effective treatment strategies.

Next-generation sequencing (NGS) is a pivotal technology in genomics, enabling the high-throughput sequencing of millions of DNA fragments simultaneously to provide comprehensive insights into genome structure, genetic variation, and changes in gene activity and behavior (19). Differentially expressed genes (DEGs) are genes whose expression levels significantly differ across various biological samples, treatment conditions, or tissues. These genes serve as important biomarkers for disease diagnosis, prognosis, and monitoring. DEGs are particularly useful in distinguishing subtypes of heterogeneous diseases, such as cancer, and in predicting patient prognosis and response to treatment (20–24). A significant body of research has focused on biomarkers associated with HLH diagnosis and prognosis (25, 26). Identifying DEGs in HLH may enable early disease detection, prognosis prediction, and the identification of novel prevention and treatment targets. However, despite the complexity of HLH’s pathophysiological network, there is a lack of systematic investigation into the specific roles of iron metabolism genes, their interactions with immune cells, and their potential as diagnostic markers or therapeutic targets.

Therefore, this study aims not only to identify individual differentially expressed genes but also to establish an integrated analytical framework. By utilizing transcriptomic data in conjunction with differential expression analysis, iron metabolism gene sets, multi-machine learning algorithm screening, immune infiltration analysis, and drug prediction, we systematically identify iron metabolism-related key genes with core functions in HLH. We also elucidate their interrelationships and potential synergistic mechanisms. We believe that this comprehensive research approach provides more valuable insights into understanding the molecular networks of HLH—a complex disease—than single-molecule discovery.

## 2 Materials and methods

### 2.1 Sample collection and grouping

This study was approved by the Medical Ethics Committee of The First People’s Hospital of Yunnan Province (KHLL2024-KY142). All samples were collected exclusively from this institution. A total of 30 peripheral blood mononuclear cell (PBMC) samples were divided into two groups: 15 PBMC samples from healthy individuals formed the control group, while 15 PBMC samples from patients diagnosed with HLH constituted the disease group. All patients with HLH met the diagnostic criteria outlined in the HLH-2004 guidelines (27), which require the presence of at least five out of eight specific criteria or identification of a pathologically specific molecular defect through genetic analysis. Patients with severe infections, extensive inflammation, cytokine storms, or iron overload due to other causes were excluded from the study. The median age of the HLH group was 39 years (range: 24–87), and the control group had a median age of 35 years (range: 25–80). The gender ratio of 8 males to 7 females was consistent across both groups. Among the 15 HLH patients, the underlying etiologies included Epstein–Barr virus (EBV) infection (*n* = 6), adults still (*n* = 1), and malignancy-associated (*n* = 8). No participants had chronic diseases. Informed consent was obtained from all participants prior to sample collection. PBMC samples from both the HLH and control groups were processed for transcriptomic RNA sequencing (RNA-seq). Additionally, 14 gene sets related to iron metabolism were retrieved from the Molecular Signatures Database (MSigDB)^1^. After removing duplicate genes, a total of 520 iron metabolism-related genes (FeRGs) were identified (Supplementary Table S1).

### 2.2 RNA sequencing and data preprocessing

Total RNA was extracted from the PBMC samples (15 from HLH and 15 from controls) using Trizol reagent. RNA integrity was assessed using an Agilent Bioanalyzer 2,100, and only high-quality samples (RIN > 7.0) were selected for subsequent library construction. Sequencing libraries were prepared following the standard Illumina protocol for poly(A) enrichment and strand-specific construction. The final libraries were quantified and sequenced on an Illumina Novaseq 6,000 platform, generating 150 bp paired-end reads. Raw sequencing data were processed to generate high-quality clean data. Specifically, adapters, polyA/G tails, and low-quality bases were removed using Cutadapt. Sequence quality was verified using FastQC, with results showing Q20% > 99.5%, Q30% > 97%, and stable GC content distribution. The HISAT2 program was used for alignment with the human genome, and gene counts were calculated using featureCounts. All subsequent analyses were conducted using these clean reads.

### 2.3 Principal component analysis (PCA) and differential expression analysis

To assess whether the transcriptome sequencing samples met the criteria for statistical analysis reproducibility, PCA was performed on both sample groups using the Factoextra package (v 1.0.7)^2^. To identify DEGs between the HLH and control groups, the DESeq2 package (v 1.38.0) (28) was utilized, applying a log_2_ fold change (FC) threshold greater than 1.0 and an adjusted *p*-value (p.adj) of less than 0.05. The Benjamini-Hochberg (BH) method was used for multiple testing correction. The DESeq2 normalization method, specifically size factor correction, was employed for data normalization. The DEGs were visualized using the ggplot2 package (v 3.4.1) (29) for the volcano plot and the ComplexHeatmap package (v 2.15.1) (30) for the heatmap. The volcano plot highlighted the top 10 DEGs with the most significant up- and down-regulation, sorted by log_2_FC values, while the heatmap illustrated the expression profiles of these DEGs.

### 2.4 Pinpointing candidate genes and functional evaluation

To identify FERGs in HLH, the VennDiagram package (v 1.7.3) (31) was used to find the intersection between the DEGs and FeRGs, resulting in candidate genes. Gene Ontology (GO) and Kyoto Encyclopedia of Genes and Genomes (KEGG) analyses were performed on these candidate genes using the clusterProfiler package (v 4.2.2) (32), with a significance threshold of *p* < 0.05. The GO analysis covered three components: Biological Processes (BPs), Cellular Components (CCs), and Molecular Functions (MFs). GO terms were ranked by *p*-value, and the top 10 most significantly enriched pathways were presented. Additionally, the top 5 enriched pathways in each category and the candidate genes associated with them were explored. To examine the interactions among candidate genes at the proteomic level, a protein–protein interaction (PPI) network was constructed using the STRING database^3^ with a confidence score > 0.15. Cytoscape (v 3.10.2) (33) was then used to visualize the interaction network of the top 5 genes, utilizing three algorithms—Degree, maximal clique centrality (MCC), and maximum neighborhood component (MNC)—to identify significant genes in these networks. The VennDiagram package was again used to determine the overlap of significant genes identified by the different algorithms.

### 2.5 Identification and evaluation of key genes

After identifying candidate genes, two machine learning algorithms were employed to screen for key genes based on sequencing data. Genes selected by the Boruta algorithm were identified using the Boruta package (v 8.0.0) (34). In this algorithm, the importance of original features was evaluated against shadow features. A gene was considered significant (Boruta-feature gene) if its importance score exceeded the shadowMax threshold, and inconsequential if it fell below shadowMin. Next, least absolute shrinkage and selection operator (LASSO) regression analysis was performed using 10-fold cross-validation (CV) with the glmnet package (v 4.1–2) (35) to identify key genes. The optimal regularization parameter lambda was determined based on the maximum area under the curve (AUC) during CV, corresponding to lambda.min. Genes with non-zero regression coefficients were classified as key genes. To evaluate the diagnostic potential of these key genes for HLH, receiver operating characteristic (ROC) curves were constructed using AUC values (AUC > 0.7) from 15 HLH and 15 control samples. The expression trends of key genes were also examined between the HLH and control groups, and differences in expression levels were compared using the Wilcoxon test (*p* < 0.05), visualized with the ggplot2 package. In addition, the expression differences of key genes between the control group and different etiological subgroups (EBV-associated HLH, Malignancy-associated HLH) were compared using the Wilcoxon test (*p* < 0.05). Due to only one sample in the adult still subgroup, this subgroup was not included in the comparison.

### 2.6 Correlation analysis and GeneMANIA analysis of key genes

The correlations and functional interactions between the key genes were analyzed to uncover their regulatory roles in biological functions and processes. Spearman’s correlation analysis was performed on all sequencing samples using the psych package (v 2.2.9) (36) (|correlation coefficient (r)| > 0.3, *p* < 0.05), with the pheatmap package (v 0.7.7) (37) used to visualize the correlation heatmaps. The GeneMANIA database^4^ was used to predict gene interactions related to the biomarker function.

### 2.7 Gene set enrichment analysis (GSEA)

To explore the biological roles of the key genes in disease development, “c2.cp.kegg.v2023.1.Hs.symbols.gmt” from the MSigDB was used as the background gene set. Spearman’s rank correlation coefficients between each key gene and other genes in the dataset were computed using the psych package, and the results were sorted in descending order. Subsequently, GSEA was conducted using the clusterProfiler package, with the significance thresholds set at |normalized enrichment score (NES)| > 1.0, p.adj < 0.05, and correction using the BH method.

### 2.8 Analysis of immune infiltration

The relative proportions of 22 immune-infiltrating cell types (38) in the HLH and control groups were evaluated from the sequencing data and visualized using the CIBERSORT package (v 1.03) (38) and the reshape2 package (v 1.4.4) (39). To further investigate immune microenvironmental differences between the groups, the Wilcoxon test was performed to identify immune infiltrating cell types with significant differences (*p* < 0.05). Visualization of these results was carried out using the ggplot2 package. Spearman correlation analysis (|cor| > 0.3, *p* < 0.05) was then performed using the “cor” function in R (v 4.2.2) to assess relationships between immune cell types and between immune cells and key genes, with results visualized *via* the pheatmap package.

### 2.9 Drug prediction

Potential HLH-targeting drugs were identified through the DrugBank database^5^, which predicts drugs targeting key genes. The interaction network between drugs and critical genes was visualized using the Cytoscape package, incorporating both FDA-approved drugs and experimental compounds, with detailed drug and drug target information.

### 2.10 Expression of key genes

Whole blood samples from patients with HLH (*n* = 5) and controls (*n* = 5) were collected at The First People’s Hospital of Yunnan Province. Total RNA from each sample was extracted using Trizol (Ambion) and reverse transcribed into complementary DNA (cDNA) using Hifair® III 1st Strand cDNA Synthesis SuperMix for qPCR. RT-qPCR was performed with 2x Universal Blue SYBR Green qPCR Master Mix, and primers for key genes were synthesized by Sangon Biotech (Shanghai, China) (Supplementary Table S2). GAPDH was used as the endogenous reference gene. Three technical replicates were performed for each biological sample. The study was approved by the Ethics Committee of the institution. Data were statistically analyzed and presented using GraphPad Prism (v 10.0) (40).

### 2.11 Statistical analysis

Bioinformatics analyses were performed using R (version 4.2.2). The Wilcoxon rank sum test was used to evaluate group differences, with a *p*-value < 0.05 considered statistically significant. The t-test was applied to compare RT-qPCR results between groups.

## 3 Results

### 3.1 Acquisition of DEGs

**Figure 1:**
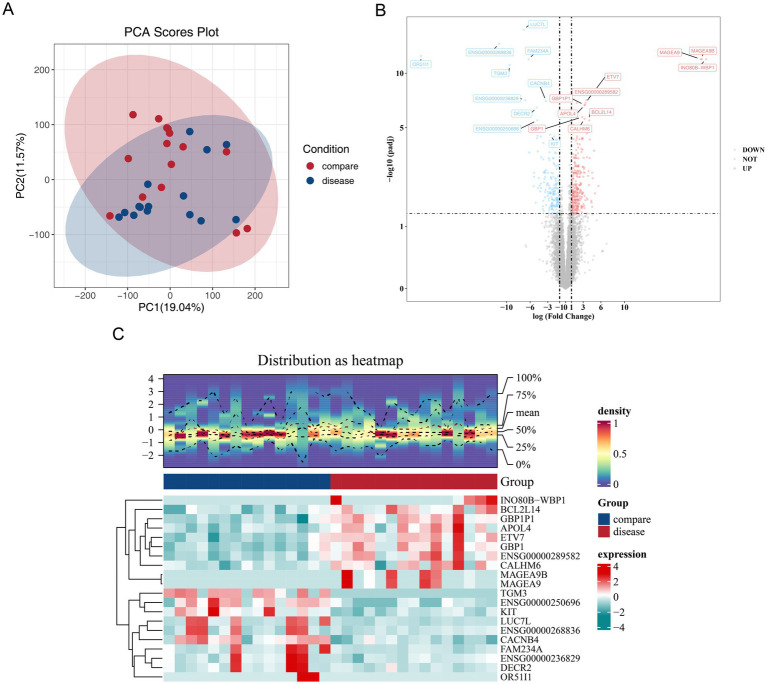
Acquisition of DEGs. **(A)** PCA analysis based on different groups. The purple and orange colors represent samples of compare and disease, respectively, with the first and second principal components displayed on the *x*-axis and *y*-axis. **(B)** Volcanoogram of differentially expressed genes between the disease sample and the control sample. Red dots represent up-regulated genes, blue dots represent down-regulated genes, and gray dots represent genes with no significant difference. Mark the 10 genes with the most significant up-regulation differences. **(C)** Heatmap of differentially expressed genes between the disease sample and the control sample. Red indicates high expression, cyan indicates low expression, and the darker the color, the higher/lower the expression.

The percentage of valid reads and quality scores (Q) for each sample fell within the high-quality range, with GC content percentages ranging from 50 to 60% (Supplementary Table S3). PCA revealed some dispersion of samples between the groups along PC1 and PC2 (Figure 1A). Differential expression analysis identified 464 DEGs between the HLH and control groups, with 294 genes up-regulated and 170 genes down-regulated in the HLH group. Volcano plots highlighted the top 10 DEGs (Figure 1B), and a heatmap was used to illustrate the expression profiles of these 20 genes (Figure 1C).

### 3.2 Enrichment and PPI analysis in 10 candidate genes

**Figure 2:**
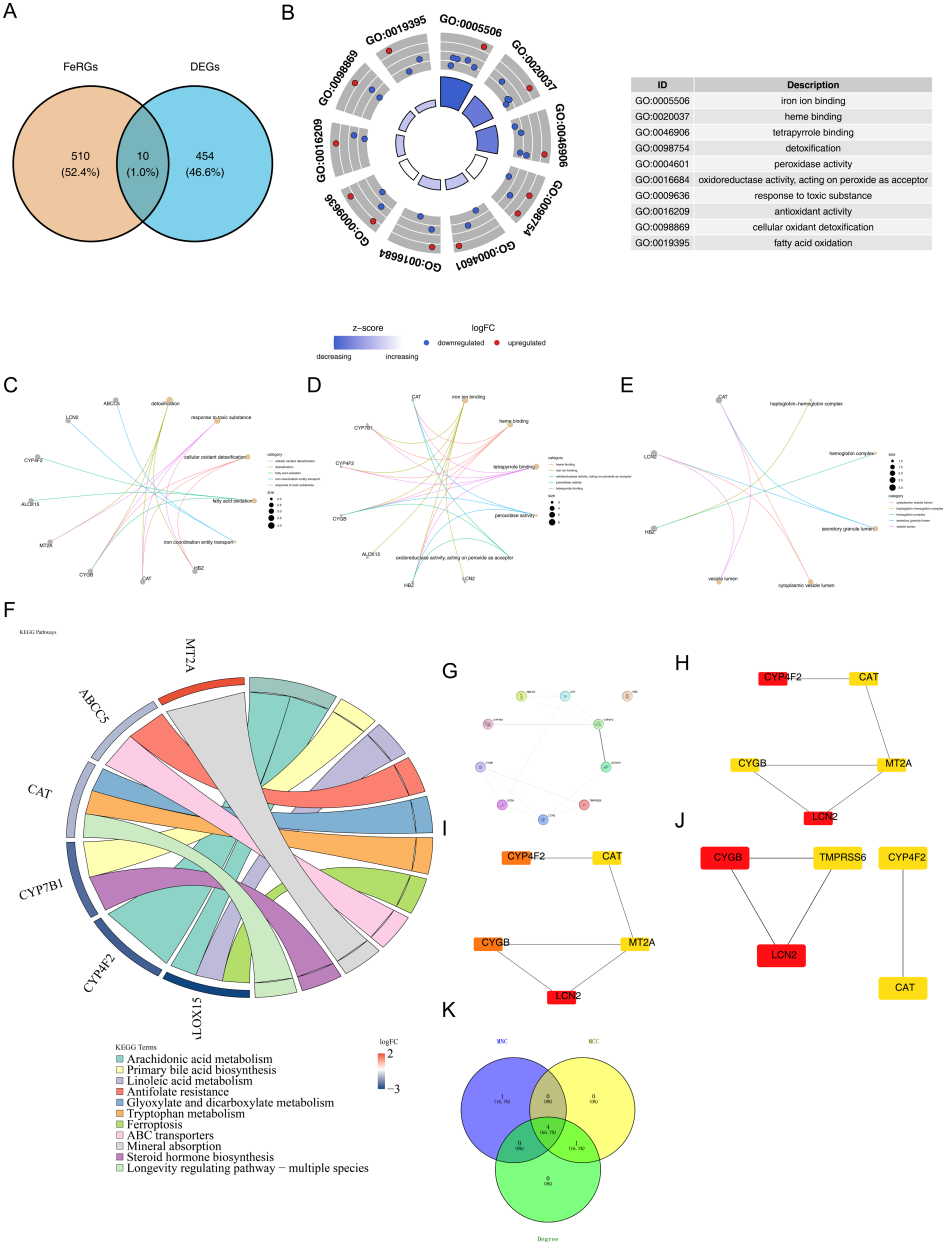
Enrichment and PPI analysis in 10 candidate genes. **(A)** Venn diagram of the intersection of DEGs and Fe-RGs. **(B)** GO enrichment analysis of Fe-DEGs. **(C–E)** Functional annotations of terms, including biological processes (BPs), molecular functions (MFs), and cellular components (CCs). **(F)** KEGG enrichment analysis results. **(G)** Candidate gene PPI network. Each node represents a candidate gene; the deeper the connection between genes, the stronger the interaction relationship at the protein level. **(H–J)** The central gene TOP5 of MCC. **(K)** Based on the plugin CytoHubba three algorithms of the central gene intersection.

A total of 464 DEGs and 520 FeRGs were cross-analyzed, resulting in the identification of 10 candidate genes (Figure 2A). Enrichment analysis of these 10 candidate genes revealed associations with 248 GO terms, with the top 5 being iron ion binding, heme binding, tetrapyrrole binding, detoxification, and peroxidase activity (Figure 2B). Of the 248 terms, 190 were related to BPs, 43 to MFs, and 15 to CCs. These terms covered a wide range of functions, including detoxification, response to toxic substances, and roles in the cytoplasm, vesicle lumen, and cytoplasmic vesicles (Figures 2C–E; Supplementary Table S4). Eleven KEGG pathways were enriched, including arachidonic acid metabolism, primary bile acid biosynthesis, linoleic acid metabolism, antifolate resistance, and glyoxylate and dicarboxylate metabolism (Figure 2F; Supplementary Table S5). The PPI network, with 10 nodes and 12 edges, showed HBZ as a discrete gene, while CYP4F2 and LCN2 exhibited the most extensive interactions with other genes (Figure 2G). The PPI network analysis identified CYGB, LCN2, CYP4F2, and CAT as critical genes using three different algorithms (Figures 2H–K).

### 3.3 ALOX15, CAT, CYGB, HBZ, and MT2A identified as key genes for HLH

**Figure 3:**
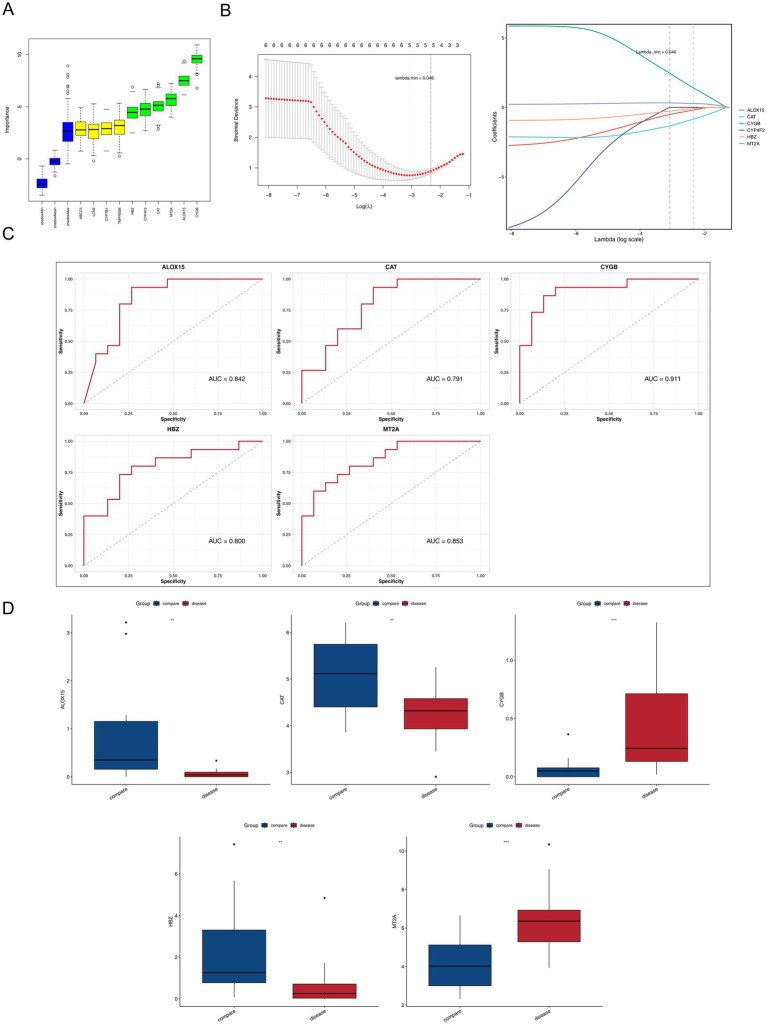
ALOX15, CAT, CYGB, HBZ and MT2A identified as key genes for HLH. **(A)** Boruta algorithm variable selection plot. Blue indicates shadow characteristics, red denotes rejection characteristics, yellow represents pending characteristics, and green signifies confirmed genes that have passed screening. **(B)** LASSO regression analysis cross-validation curves and LASSO regression analysis gene coefficient plot. **(C)** ROC plot predicted by the Lasso model. The horizontal axis is 1-sensitivity and the vertical axis is sensitivity. **(D)** Expression analysis of key genes in sequencing data. ** represents *p* < 0.01, and *** represents *p* < 0.001.

Based on these 10 candidate genes, the Boruta algorithm identified 6 feature genes—ALOX15, CAT, CYGB, CYP4F2, HBZ, and MT2A (Figure 3A). In the LASSO model, 5 genes—ALOX15, CAT, CYGB, HBZ, and MT2A—were selected (lambda.min = 0.046), which were recorded as the final key genes (Figure 3B). The AUC for these 5 genes was greater than 0.7 (Figure 3C). Expression validation showed that all 5 key genes exhibited significant differential expression in the sequencing dataset; ALOX15, CAT, and HBZ were down-regulated in the disease group, while MT2A and CYGB were up-regulated (Figure 3D). Analysis of expression levels between the control samples and different etiological subgroups also showed the same trend: ALOX15, CAT, and HBZ were downregulated in both the EBV-associated HLH and Malignancy-associated HLH subgroups, while MT2A and CYGB were upregulated in these two subgroups (Supplementary Figure S1). These results suggest that the identified key genes possess good discriminatory power for distinguishing patients with HLH from control samples, indicating their potential diagnostic value.

### 3.4 Key gene correlations, enrichment pathways, and drug prediction

**Figure 4:**
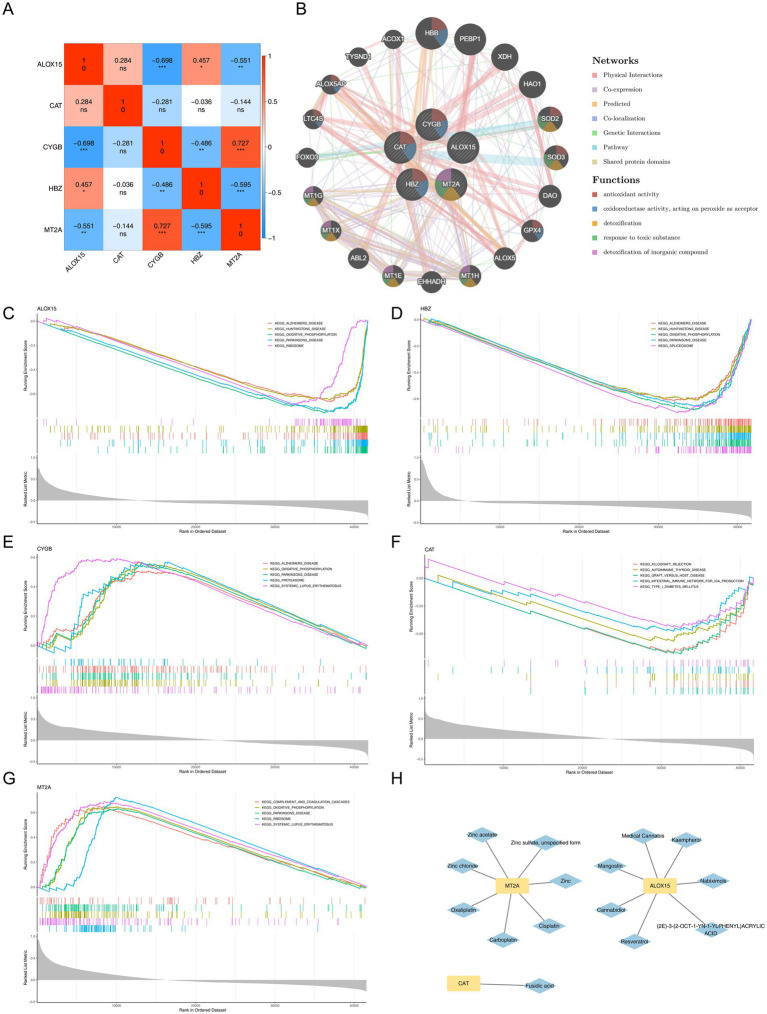
Key gene correlations, enrichment pathways and drug prediction. **(A)** Correlation heatmaps of key genes. Ns indicates no significant difference; *denotes significant (*p* < 0.05); the redder the color, the stronger the positive correlation; the bluer the color, the stronger the negative correlation. **(B)** Co-expression network analysis of key genes. The different colors of the lines indicate different connections between genes. The colors of gene nodes represent different functions. **(C–G)** GSEA enrichment of ALOX15 **(C)**, HBZ **(D)**, CYGB **(E)**, CAT **(F)**, MT2A**(G)**. **(H)** Key genes and corresponding potential therapeutic drug networks. Yellow is the key gene; Blue represents medicine.

Interactions among the five key genes revealed significant correlations. A positive correlation was observed between ALOX15 and HBZ (correlation coefficient = 0.457), while negative correlations were found between ALOX15 and both CYGB and MT2A (correlation coefficients = −0.689 and −0.551, respectively). Furthermore, CYGB and MT2A exhibited a significant positive correlation (correlation coefficient = 0.727) (Figure 4A). These key genes were associated with functions such as antioxidant activity, detoxification, response to toxic substances, and detoxification of inorganic compounds, with connections to genes like HBB, PEBP1, and XHD (Figure 4B). The biological functions of the five key genes were further investigated using the training set. ALOX15 was linked to 116 pathways (Figure 4C; Supplementary Table S6), HBZ to 127 pathways (Figure 4D; Supplementary Table S7), CYGB to 23 pathways (Figure 4E; Supplementary Table S8), CAT to 119 pathways (Figure 4F; Supplementary Table S9), and MT2A to 48 pathways (Figure 4G; Supplementary Table S10). The top 10 enriched pathways across all five key genes included oxidative phosphorylation and Huntington’s disease. Drug prediction results showed that ALOX15, MT2A, and CAT were associated with 15 predicted drugs, including resveratrol, fusidic acid, and cisplatin (Figure 4H; Supplementary Table S11).

### 3.5 Dendritic cell activation and T cell CD4 memory activation in HLH disease

**Figure 5:**
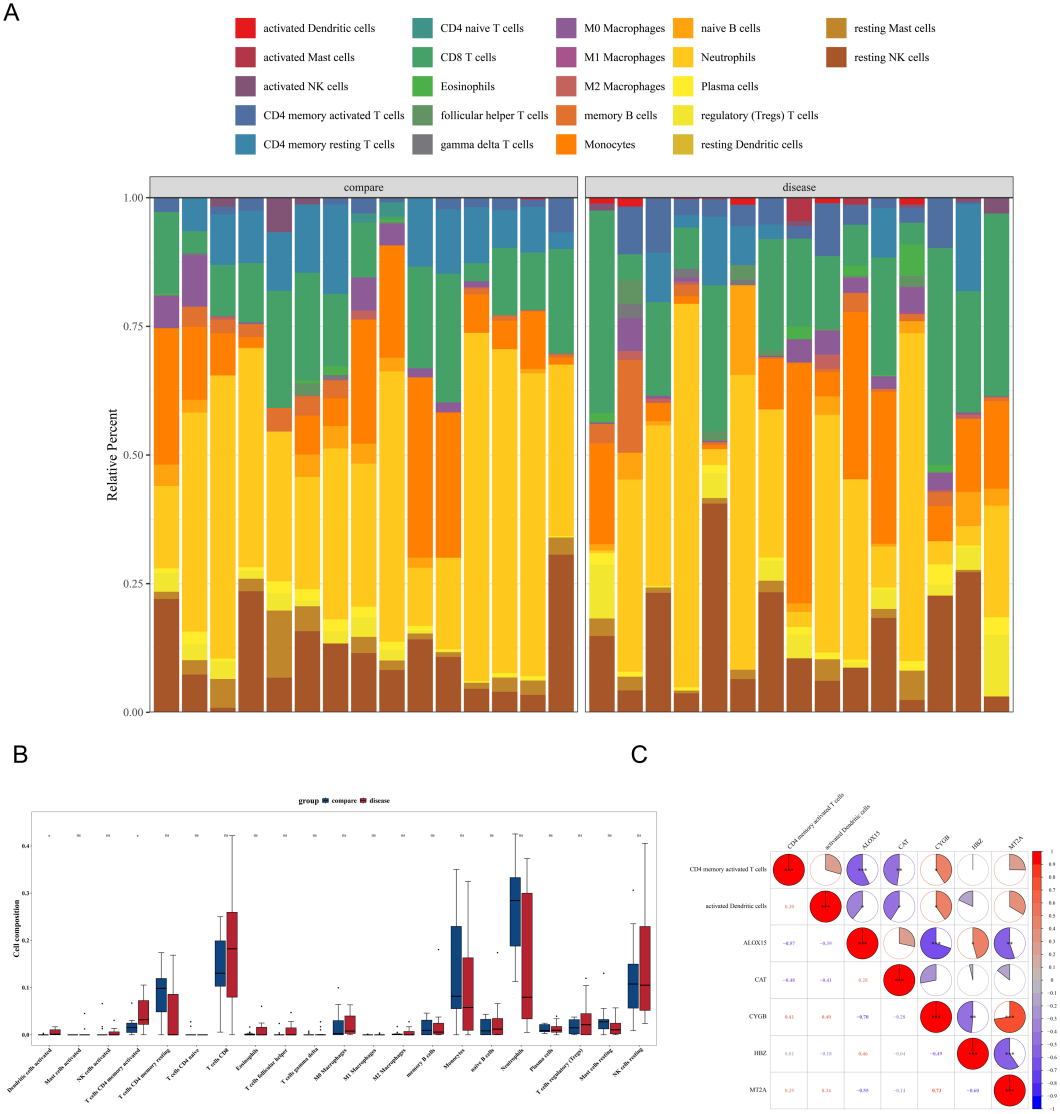
Dendritic cell activation and T cell CD4 memory activation in HLH disease. **(A)** Stacked plot of the abundance of infiltration of 22 immune cells in the disease vs. control group. The horizontal axis represents the sample, different colors represent different cells, and the vertical axis represents the infiltration level. **(B)** Differences in immune infiltrating cells between different groups. **(C)** Heatmap of correlations between key genes and differential immune cells. Red indicates a positive correlation, and blue indicates a negative correlation. The darker the color, the stronger the correlation. ns represents *p* > 0.05, * represents *p* < 0.05, ** represents *p* < 0.01, and *** represents *p* < 0.001.

In the HLH and control groups, significant differences in the relative percentages of immune infiltrating cell types were observed. Specifically, the proportions of monocytes and neutrophils were higher in all samples (Figure 5A). Further analysis revealed significant differences in activated dendritic cells and CD4 memory activated T cells between the two groups, with the disease group showing markedly higher infiltration levels (Figure 5B). Regarding the relationships between genes and immune cell profiles, two key genes, ALOX15 and CAT, exhibited a significant inverse correlation with the differentially expressed immune cells (correlation coefficient < −0.30, *p* < 0.05), whereas CYGB showed a strong positive association (correlation coefficient > 0.30, p < 0.05). Additionally, complex associations among the key genes ALOX15, CYGB, MT2A, and HBZ were observed. Specifically, ALOX15 showed a significant negative correlation with CYGB and MT2A (correlation coefficient < −0.30, p < 0.05), MT2A exhibited a significant positive correlation with CYGB (correlation coefficient > 0.30, p < 0.05), and MT2A displayed a significant negative correlation with HBZ (correlation coefficient < −0.30, *p* < 0.05) (Figure 5C).

### 3.6 Expression verification of key genes

**Figure 6:**
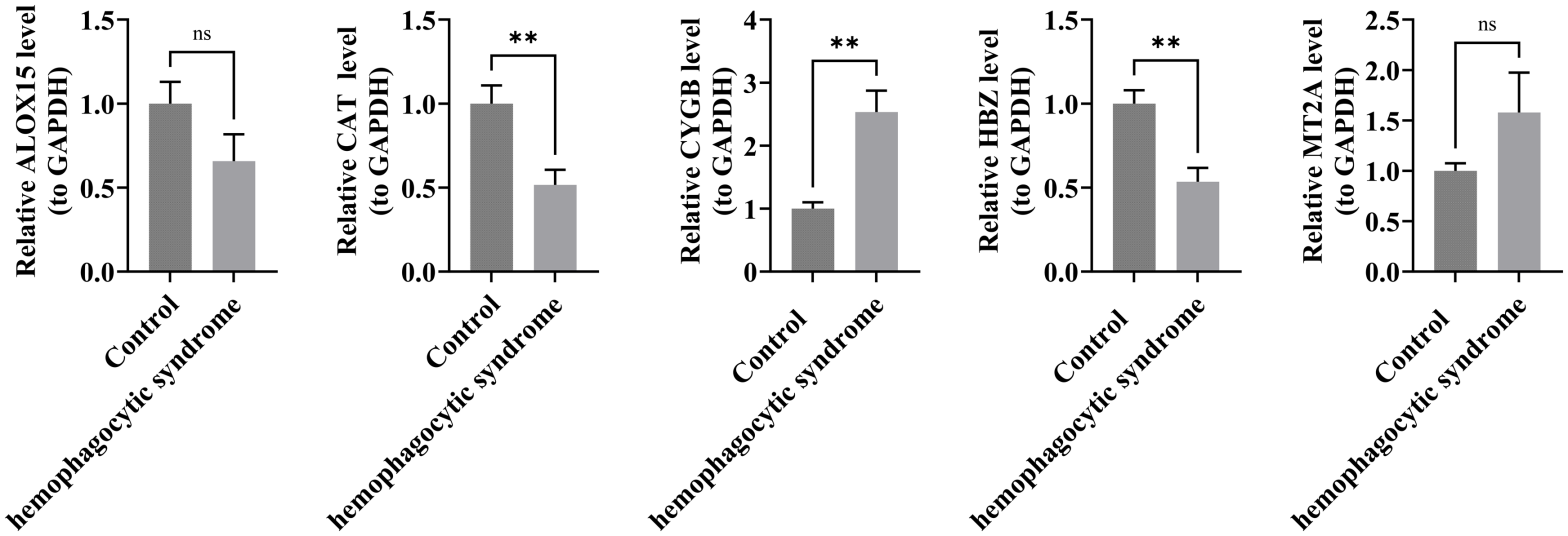
Expression verification of key genes. ns represents *p* > 0.05, ** represents *p* < 0.01.

RT-qPCR results indicated that in the HLH group, the expressions of CAT and HBZ were downregulated, with significant differences in expression levels. ALOX15 also showed a downward trend in expression, while CYGB was upregulated with significant differences. MT2A expression was similarly upregulated. The expression trends of these five key genes were consistent with the differential expression analysis results (Figure 6).

## 4 Discussion

HLH is a life-threatening inflammatory response disorder that causes severe organ dysfunction, high mortality, and poor treatment outcomes (25, 41). Elevated ferritin levels are a hallmark of HLH (26). Continued research will further elucidate the relationship between HLH and iron metabolism, providing more accurate insights for the diagnosis and treatment of this condition. This study employed transcriptomic analysis to systematically identify five core functional key genes (ALOX15, CAT, HBZ, MT2A, and CYGB) within the FeRGs family in the context of HLH. The study also explored their biological roles and molecular mechanisms, offering valuable information on the pathogenesis of HLH and potential therapeutic strategies.

Notably, although hemochromatosis (HLH) is a key diagnostic indicator and iron overload is acknowledged as a pathological feature (10, 11), the molecular regulatory network of iron metabolism-related genes in the pathogenesis of HLH has been long overlooked. This research gap may arise from various factors. Historically, HLH research has primarily concentrated on immunological aspects—especially cytokine storms, cytotoxic lymphocyte dysfunction, and genetic mutations in familial HLH—often considering iron metabolism abnormalities as “secondary phenomena” rather than fundamental causes (42). From an interdisciplinary standpoint, iron metabolism and immunology developed as relatively distinct fields until the recent emergence of the “metabolism-immunity interaction” concept, which has promoted their convergence. Technologically, the rarity and acute lethality of HLH cases make obtaining samples extremely difficult, limiting the feasibility of large-scale transcriptomic studies (14, 43, 44). Methodologically, early research lacked tools that integrated multi-omics data with machine learning algorithms, impeding the systematic analysis of complex gene networks (20, 28). Iron proteins have traditionally been viewed as “diagnostic markers” rather than “mechanistic targets,” leading researchers to focus on exploring classical inflammatory pathways (e.g., IFN-*γ*/JAK–STAT axis) (26, 45). This study, by integrating transcriptome sequencing, iron metabolism gene set enrichment, immune microenvironment analysis, and drug prediction, for the first time, places FeRGs at the center of the HLH pathological network, rather than at the periphery. This paradigm shift suggests that dysregulation of iron metabolism and immune activation may create a “positive feedback loop,” rather than merely indicating the severity of tissue damage, offering a new framework for understanding the multifactorial pathogenic nature of HLH.

15-lipoxygenase-1 (ALOX15) is a critical enzyme in arachidonic acid metabolism (46, 47). Its downregulation in patients with HLH likely reduces the production of lipid mediators, such as lipoxins, which have potent anti-inflammatory properties (48, 49). Consequently, ALOX15 deficiency could disrupt the balance between pro-inflammatory and anti-inflammatory signals, exacerbating uncontrolled macrophage activation and cytokine storms in HLH. Additionally, ALOX15 and its metabolites have been shown to regulate ferroptosis directly or indirectly (50, 51). In the context of HLH, the downregulation of ALOX15 may alter immune cell death patterns, thereby influencing the persistence and severity of inflammation.

Catalase (CAT) is a vital antioxidant enzyme that decomposes H_2_O_2_ into water and oxygen, reducing oxidative stress. The downregulation of its expression directly impairs the body’s ability to clear H_2_O_2_ (52). In the hyperinflammatory state of hemophagocytic lymphohistiocytosis (HLH), immune cells, including activated macrophages and T cells, generate excessive ROS (42, 53). CAT deficiency leads to the accumulation of H_2_O_2_, which not only causes oxidative damage to cellular structures but also acts as a key signaling molecule that activates pro-inflammatory pathways, such as NF-κB (54). This creates a positive feedback loop between ROS and inflammation, amplifying the inflammatory response and exacerbating tissue damage, particularly in organs like the liver.

The HTLV-1 bZIP factor (HBZ), typically associated with HTLV-1 viral infection (55), is aberrantly expressed in HLH, suggesting that viral infection may trigger the development of HLH through the HBZ protein. HBZ has been shown to modulate the epigenetic and metabolic states of T cells by regulating transcription factors, such as AP-1, thereby suppressing T-cell immune function and promoting abnormal proliferation (56, 57). This finding aligns with the significant increase in activated CD4 + memory T cells observed in our immune infiltration analysis. HBZ may drive T cells to differentiate into pathogenic effector/memory phenotypes, which are prone to migrate to various tissues and continuously produce inflammatory factors, thus contributing to T-cell-driven immune dysregulation in HLH.

Metallothionein-2A (MT2A), a member of the metallothionein family (58), is upregulated in HLH, likely reflecting an adaptive cellular response to metal ion dysregulation and severe oxidative stress. In HLH, metal ions such as iron, released during red blood cell phagocytosis, and zinc, present in inflammatory microenvironments, may induce MT2A expression. MT2A acts by chelating these metal ions, which both reduces the availability of Fenton reaction catalysts, thus alleviating oxidative stress (59), and contributes to the dynamic balance of metal ions, which is essential for the activation and signal transduction of immune cells, including T cells (60). However, excessive MT2A expression could disturb intracellular zinc homeostasis, thereby indirectly altering T cell activation thresholds and functions, playing a complex role in the immune dysregulation observed in HLH.

Cytoglobin (CYGB), located on human chromosome 17q25 (61), possesses antioxidant properties and can neutralize toxic substances produced during tissue ischemia–reperfusion, such as ROS and nitrogen oxide (NO) groups (62). This helps protect cells and mitigate oxidative stress damage (63). While its expression is linked to liver and kidney diseases, the precise mechanisms of action remain to be fully elucidated (64, 65). CYGB may suppress cancer cell growth *in vitro*, but its role in tumor development requires further investigation (62). Similarly, its specific role in HLH is not yet fully understood.

In summary, the five key genes related to iron metabolism identified in this study likely form a network that connects iron metabolism, oxidative stress, and immune dysfunction, offering new insights into the molecular mechanisms underlying HLH.

The functional enrichment analysis revealed that these key genes are significantly enriched in pathways, including oxidative phosphorylation, the primary cellular energy-producing pathway. Dysfunction in oxidative phosphorylation exacerbates HLH by facilitating inflammation through multiple mechanisms. First, it impairs the energy supply of immune cells, such as macrophages and lymphocytes, hindering their proliferation, cytotoxicity, and cytokine production (66). Second, it leads to increased mitochondrial ROS generation (67, 68). These reactive molecules induce oxidative stress, damaging cellular components, including DNA, proteins, and lipids (69), and activating pro-inflammatory pathways. This, in turn, promotes the release of pro-inflammatory cytokines such as IFN-*γ*, IL-6, and TNF-*α*, driving the characteristic cytokine storm of HLH (70). Continuous activation of immune cells leads to a vicious cycle of inflammation and tissue damage (45).

Drug target prediction for the key genes ALOX15, CAT, and MT2A was conducted using the DrugBank database, revealing potential interactions with compounds such as resveratrol and fucoidan. While these computational predictions suggest a theoretical “druggable” potential for these targets, they should be regarded with caution. Currently, they lack direct relevance to HLH treatment and remain speculative. For example, while resveratrol has demonstrated anti-inflammatory and antioxidant properties in various models (71, 72), its specific role in the complex HLH immune environment remains unexplored. Thus, these predictions should not be interpreted as therapeutic recommendations but may serve as valuable starting points for future mechanistic studies, including molecular docking and functional screening. It is essential for future research to include *in vitro* and *in vivo* experiments to validate the functional roles and regulatory mechanisms of these targets in HLH.

Marked differences in the immune microenvironment between HLH and control groups were observed, with a notable correlation to key genes. This suggests that these key genes may influence the immune microenvironment by regulating the activity and function of specific immune cells, which could, in turn, impact HLH progression and treatment responses. Understanding these correlations is critical for the development of new immunotherapy strategies aimed at improving HLH treatment outcomes. For instance, a study indicated that pregnant women with HBV infection displayed a more vigorous immune response compared to healthy pregnant women, with increased expression of MT-associated genes (MT2A, MT1E, MT1F, MT1X) in CD4 + and CD8 + T cells from those with HBV infection (73). IFN-*α* and inflammatory cytokines such as TNF, IL-1β, and IL-6 can induce MT expression (74, 75); however, whether MTs suppress or activate T cell function remains contentious. Our prior research found elevated levels of TNF-α and IL-1β in patients with HLH compared to healthy controls (76), and the increased expression of MT2A identified in this study may be associated with inflammatory factor responses. Additionally, other studies have linked the activation and proliferation of CD4 + T cells to increased MT expression (77). HBZ has been shown to drive the transformation of infected T cells into CCR4 + TIGIT+CD4 + effector/memory T cells, which can migrate to various organs and persist within the body (78). This may explain the prominent presence of HBZ cells in HLH, primarily characterized by activated CD4 + memory T cells.

This study has preliminarily revealed the potential role of iron metabolism-related genes in HLH through bioinformatics analysis, identifying five core key genes: ALOX15, CAT, HBZ, MT2A, and CYGB. However, we must acknowledge several limitations of this research. First, the limited sample size for bioinformatics analysis and RT-qPCR could not fully eliminate the risk of overfitting, despite employing high-dimensional small-sample algorithms like LASSO and Boruta. Furthermore, the limited sample size in RT-qPCR validation may result in insufficient statistical power of the findings. As a preliminary exploratory study, it lacks validation through independent external datasets, and high-dimensional data from high-throughput sequencing cannot replace the statistical robustness and generalizability of large-sample cohorts. Moreover, collecting large samples from a single center within a short timeframe poses objective challenges due to HLH’s rarity. Therefore, we cannot yet validate our findings in larger population cohorts.

Secondly, the validation depth of this study requires further enhancement. While RT-qPCR only verified gene expression levels, the specific molecular mechanisms of these genes in HLH pathogenesis, their precise regulation of iron metabolism-immunomicroenvironment interactions, and their effects on oxidative stress and ROS pathways remain unclear. These critical scientific questions need to be further investigated through functional gain/loss experiments (such as cell transfection, gene knockout/downregulation), animal models, and protein expression analysis.

Despite these limitations, we believe this study provides new perspectives and valuable hypotheses for understanding the complex pathophysiological network of HLH. In the future, we plan to conduct multicenter, large-scale collaborative research to validate these findings. Simultaneously, we will utilize cell and animal models to validate the functions of key genes such as ALOX15 and CAT, while exploring their potential as therapeutic targets. Additionally, we intend to employ more advanced technologies like single-cell sequencing to decipher the interactions between immune cells and metabolic disorders in HLH at a higher resolution.

## 5 Conclusion

In conclusion, this study highlights the differential expression of FERGs (ALOX15, CAT, HBZ, MT2A, and CYGB) in patients with HLH compared to healthy controls, highlighting their pivotal role in the pathogenesis of HLH and their potential as key genes. This exploratory research contributes to a better understanding of HLH pathogenesis and sets the stage for the development of enhanced diagnostic tools and targeted therapies. Further investigations into these mechanisms will continue to advance the field.

## Data Availability

The data presented in the study are deposited in the Genome Sequence Archive repository, accession number:subHRA024426.BioProject ID: PRJCA054334.

## Footnotes

1 https://www.gsea-msigdb.org/gsea/msigdb/index.jsp

2 https://rpkgs.datanovia.com/factoextra/

3 https://www.string-db.org

4 https://genemania.org/

5 https://go.drugbank.com/
