# Supplementary material for: Identification of iron metabolism-related key genes and exploration of their potential mechanisms in hemophagocytic lymphohistiocytosis based on transcriptome sequencing
Source: Front Med (Lausanne). 2026 Jan 14;12:1685793. doi: 10.3389/fmed.2025.1685793 (PMC12846933; doi:10.3389/fmed.2025.1685793)

| Table S1 After removing the duplicate genes, a total of 520 iron metabolism-related genes (FeRGs) were obtained | | | | | |
| --- | --- | --- | --- | --- | --- |
| HFE | BOLA2 | EIF2AK1 | AGPAT4 | BCAM | CYP17A1 |
| SLC11A2 | BOLA2B | EPB42 | AHSP | BCL2 | CYP19A1 |
| TF | FBXL5 | ERFE | AIFM3 | BECN1 | CYP1A2 |
| TFRC | GLRX3 | FDX1 | ALDH1L1 | BMP2K | CYP1B1 |
| FTH1 | HEPHL1 | FLVCR2 | ALDH6A1 | BNIP3L | CYP20A1 |
| FXN | HIF1A | FRRS1 | ALKBH1 | BPGM | CYP21A2 |
| HPX | HMOX2 | GDF2 | ALKBH2 | BRIP1 | CYP24A1 |
| IREB2 | ISCA2 | GLRX5 | ALKBH3 | BSG | CYP26A1 |
| ISCU | MELTF | HBQ1 | ALKBH8 | BTBD9 | CYP26B1 |
| LCN2 | MYC | HBZ | ALOX12 | BTG2 | CYP26C1 |
| SLC6A9 | NCOA4 | HJV | ALOX12B | BTRC | CYP27A1 |
| ABCB6 | NFE2L1 | HMBS | ALOX15 | C3 | CYP27B1 |
| ACO1 | NUBP1 | HRG | ALOX15B | CA1 | CYP27C1 |
| ALAS2 | PPOX | ISCA1 | ALOX5 | CA2 | CYP2A13 |
| CP | SCARA5 | KDM7A | ALOXE3 | CALR | CYP2A6 |
| CYBRD1 | SLC11A1 | MCOLN1 | AMBP | CAND1 | CYP2A7 |
| FECH | SLC25A37 | NDFIP1 | ANK1 | CAST | CYP2B6 |
| FLVCR1 | SLC39A14 | NDUFS1 | AQP3 | CAT | CYP2C18 |
| FTL | SNCA | NFU1 | ARHGAP1 | CCDC28A | CYP2C19 |
| FTMT | SRI | P4HA2 | ARHGEF12 | CCND1 | CYP2C8 |
| HEPH | STEAP3 | PGRMC2 | ARL2BP | CCND3 | CYP2C9 |
| HMOX1 | STEAP4 | PICALM | ASNS | CDC27 | CYP2D6 |
| SLC22A17 | ABCC5 | RHAG | ATG4A | CDK5RAP1 | CYP2D7 |
| SLC40A1 | ABCE1 | SDHB | ATG5 | CDKAL1 | CYP2E1 |
| SLC46A1 | ABCG2 | SLC25A28 | ATP5IF1 | CDO1 | CYP2F1 |
| STEAP2 | ACO2 | SLC25A38 | ATP6V0A4 | CDR2 | CYP2G1P |
| TFR2 | ACP5 | SLC48A1 | ATP6V0B | CH25H | CYP2J2 |
| ABCB7 | ALAS1 | SMAD4 | ATP6V0C | CIAO3 | CYP2R1 |
| ALAD | AOX1 | SOD1 | ATP6V0D2 | CIR1 | CYP2S1 |
| CPOX | ASIC3 | TMEM199 | ATP6V0E1 | CISD1 | CYP2U1 |
| FTH1P19 | ATP13A2 | TMPRSS6 | ATP6V0E2 | CISD2 | CYP2W1 |
| FTHL17 | ATP6V0A1 | TSPO | ATP6V1B1 | CISD3 | CYP39A1 |
| HAMP | ATP7A | TTC7A | ATP6V1B2 | CLCN3 | CYP3A4 |
| IFNG | BDH2 | UROD | ATP6V1C1 | CLIC2 | CYP3A43 |
| LTF | BLVRA | UROS | ATP6V1C2 | CLTC | CYP3A5 |
| MIR210 | BLVRB | XDH | ATP6V1D | CROCCP2 | CYP3A7 |
| SLC39A8 | C1orf194 | ABAT | ATP6V1E1 | CTNS | CYP46A1 |
| ATP6AP1 | CCDC115 | ACKR1 | ATP6V1E2 | CTSB | CYP4A11 |
| ATP6V0A2 | CIAPIN1 | ACSL6 | ATP6V1F | CTSE | CYP4A22 |
| ATP6V0D1 | COX10 | ADD1 | ATP6V1G2 | CUL1 | CYP4B1 |
| ATP6V1A | COX15 | ADD2 | ATP6V1G3 | CYGB | CYP4F11 |
| ATP6V1G1 | CYB561 | ADI1 | ATP6V1H | CYP11A1 | CYP4F12 |
| B2M | CYB561A3 | ADIPOR1 | BACH1 | CYP11B1 | CYP4F2 |
| BMP6 | CYP1A1 | AGMO | BBOX1 | CYP11B2 | CYP4F22 |
| CYP4F3 | FN3K | LMO2 | OGFOD3 | RIOK3 | TFDP2 |
| CYP4F8 | FOXJ2 | LMTK2 | OPTN | RIOX1 | TFF1 |
| CYP4V2 | FOXO3 | LPIN2 | OSBP2 | RNF123 | TH |
| CYP4X1 | FTCD | LRP10 | P3H1 | RNF19A | TMCC2 |
| CYP4Z1 | FTO | MAP1LC3A | P3H2 | RPS27A | TMEM14C |
| CYP4Z2P | G6PD | MAP2K3 | P3H3 | RRM2 | TMEM9B |
| CYP51A1 | GAPVD1 | MARCHF2 | P4HA1 | RSAD1 | TMLHE |
| CYP7A1 | GATA1 | MARCHF8 | P4HA3 | RSAD2 | TNRC6B |
| CYP7B1 | GCLC | MARK3 | P4HTM | RTEL1 | TNS1 |
| CYP8B1 | GCLM | MBOAT2 | PAH | SC5D | TOP1 |
| DAAM1 | GDE1 | MFHAS1 | PC | SCD | TPH1 |
| DCAF10 | GLRX2 | MGST3 | PDX1 | SCD5 | TPH2 |
| DCAF11 | GMPS | MINPP1 | PDZK1IP1 | SDCBP | TRAK2 |
| DCUN1D1 | GYPA | MIOX | PGLS | SEC14L1 | TRIM10 |
| DDX11 | GYPB | MKRN1 | PGRMC1 | SELENBP1 | TRIM58 |
| DMTN | GYPC | MMGT1 | PHF2 | SFXN1 | TSPAN5 |
| DNA2 | GYPE | MOCOS | PHF8 | SIDT2 | TSPO2 |
| DNAJC24 | H1-0 | MOCS1 | PHYH | SKP1 | TTYH1 |
| DNM2 | H4C3 | MOSPD1 | PIGQ | SLC10A3 | TYR |
| DOHH | HAAO | MPP1 | PLOD1 | SLC22A4 | TYW1 |
| DPYD | HAGH | MSMO1 | PLOD2 | SLC25A39 | TYW1B |
| DRD2 | HBA1 | MT2A | PLOD3 | SLC2A1 | TYW5 |
| E2F2 | HBA2 | MUTYH | POLD1 | SLC30A1 | UBA52 |
| EGLN1 | HBB | MXI1 | POLE | SLC30A10 | UBAC1 |
| EGLN2 | HBBP1 | MYL4 | PPAT | SLC4A1 | UBB |
| EGLN3 | HBD | NARF | PPEF1 | SLC66A2 | UBC |
| ELL2 | HDGF | NDUFS2 | PPEF2 | SLC6A3 | UCP2 |
| ELP3 | HEBP1 | NDUFS7 | PPP2R5B | SLC6A8 | UGT1A1 |
| ENDOD1 | HIF1AN | NDUFS8 | PRDX2 | SLC7A11 | UGT1A4 |
| EPAS1 | HTATIP2 | NDUFV1 | PRIM2 | SMOX | UQCRFS1 |
| EPB41 | HTRA2 | NDUFV2 | PSMD9 | SOD2 | UQCRFS1P1 |
| EPOR | HYAL2 | NECTIN1 | PTGIS | SPTA1 | USP15 |
| ERCC2 | IBA57 | NEDD8 | RAB11B | SPTB | VEZF1 |
| ERMAP | ICAM4 | NEK7 | RAD23A | SRRD | XK |
| ETFDH | IGSF3 | NEO1 | RANBP10 | STEAP1 | XPO7 |
| ETHE1 | JMJD6 | NFE2 | RAP1GAP | SYNJ1 | YPEL5 |
| EXO5 | KAT2B | NNT | RBM38 | TAL1 |  |
| EZH1 | KDM3A | NR3C1 | RBM5 | TBXAS1 |  |
| FA2H | KEL | NTHL1 | RCL1 | TCEA1 |  |
| FAXDC2 | KHNYN | NUBP2 | REP15 | TCIRG1 |  |
| FBXO34 | KLF1 | NUBPL | REV3L | TENT5C |  |
| FBXO7 | KLF3 | NUDT4 | RFESD | TET1 |  |
| FBXO9 | LAMP2 | OGFOD1 | RHCE | TET2 |  |
| FDX2 | LIAS | OGFOD2 | RHD | TFAP2A |  |

**Table S2** Primers for biomarkers

| **Primer** | **Sequence** |
| --- | --- |
| ALOX15 F | GGGCAAGGAGACAGAACTCAA |
| ALOX15 R | CAGCGGTAACAAGGGAACCT |
| CAT F | ACTTCTGGAGCCTACGTCCT |
| CAT R | AAAGTCTCGCCGCATCTTCA |
| HBZ F | GGTGAAGAGCATCGACGACA |
| HBZ R | CAGCGGTACTTCTCGGTCAG |
| MT2A F | CTCTTCAGCTCGCCATGGAT |
| MT2A R | ATAGCAAACGGTCACGGTCA |
| CYGB F | GGCCGAGTTCTGAAGACCC |
| CYGB R | CCTCCTTCGGGGAAGTTGAG |
| GAPDH F | ATGGGCAGCCGTTAGGAAAG |
| GAPDH R | AGGAAAAGCATCACCCGGAG |

**Table S3** The percentage of valid ratio(reads) and quality scorerates (Q) for each sample

| Sample | Raw Data |  | Valid Data |  | Valid Ratio(reads) | Q20% | Q30% | GC content% |
| --- | --- | --- | --- | --- | --- | --- | --- | --- |
|  | Read | Base | Read | Base |  |  |  |  |
| D1 | 36179842 | 5.43G | 34975468 | 5.25G | 96.67 | 99.74 | 97.56 | 56 |
| D10 | 41504838 | 6.23G | 40009304 | 6.00G | 96.40 | 99.77 | 98.24 | 55.50 |
| D11 | 35909468 | 5.39G | 34583648 | 5.19G | 96.31 | 99.76 | 98.19 | 52.50 |
| D12 | 42577378 | 6.39G | 40950410 | 6.14G | 96.18 | 99.69 | 98.06 | 52.50 |
| D13 | 37965774 | 5.69G | 36647838 | 5.50G | 96.53 | 99.63 | 97.96 | 55 |
| D14 | 39618176 | 5.94G | 38394154 | 5.76G | 96.91 | 99.63 | 98.01 | 58 |
| D15 | 44114622 | 6.62G | 42483712 | 6.37G | 96.30 | 99.76 | 98.06 | 51.50 |
| D16 | 37012152 | 5.55G | 35598210 | 5.34G | 96.18 | 99.67 | 97.94 | 51 |
| D2 | 36442870 | 5.47G | 35224410 | 5.28G | 96.66 | 99.64 | 97.07 | 52.50 |
| D3 | 38746260 | 5.81G | 37595678 | 5.64G | 97.03 | 99.69 | 97.20 | 56 |
| D4 | 39570398 | 5.94G | 38556972 | 5.78G | 97.44 | 99.70 | 97.31 | 56 |
| D5 | 48926176 | 7.34G | 47006794 | 7.05G | 96.08 | 99.65 | 97.91 | 50.50 |
| D6 | 36326270 | 5.45G | 35012764 | 5.25G | 96.38 | 99.67 | 98.04 | 54 |
| D7 | 38159640 | 5.72G | 36565644 | 5.48G | 95.82 | 99.59 | 97.77 | 51 |
| D8 | 40226202 | 6.03G | 38927210 | 5.84G | 96.77 | 99.75 | 98.18 | 56 |
| D9 | 41374108 | 6.21G | 40038690 | 6.01G | 96.77 | 99.63 | 98.00 | 57 |

**Table S4**

|  | Category | ID | term | Genes | pvalue |  |
| --- | --- | --- | --- | --- | --- | --- |
| GO:0005506 | MF | GO:0005506 | iron ion binding | LCN2,HBZ,ALOX15,CYGB,CYP4F2,CYP7B1 | 6.05718746300296e-11 |  |
| GO:0020037 | MF | GO:0020037 | heme binding | HBZ,CAT,CYGB,CYP4F2,CYP7B1 | 5.54709171983964e-09 |  |
| GO:0046906 | MF | GO:0046906 | tetrapyrrole binding | HBZ,CAT,CYGB,CYP4F2,CYP7B1 | 7.8718496972205e-09 |  |
| GO:0098754 | BP | GO:0098754 | detoxification | HBZ,CAT,CYGB,MT2A | 8.56402641067763e-07 |  |
| GO:0004601 | MF | GO:0004601 | peroxidase activity | HBZ,CAT,CYGB | 2.9725452998103e-06 |  |
| GO:0016684 | MF | GO:0016684 | oxidoreductase activity, acting on peroxide as acceptor | HBZ,CAT,CYGB | 3.31340056450849e-06 |  |
| GO:0009636 | BP | GO:0009636 | response to toxic substance | HBZ,CAT,CYGB,MT2A | 5.89213347907548e-06 |  |
| GO:0016209 | MF | GO:0016209 | antioxidant activity | HBZ,CAT,CYGB | 1.1095689792896e-05 |  |
| GO:0098869 | BP | GO:0098869 | cellular oxidant detoxification | HBZ,CAT,CYGB | 1.67804051604433e-05 |  |
| GO:0019395 | BP | GO:0019395 | fatty acid oxidation | ALOX15,CYGB,CYP4F2 | 2.23344191731959e-05 |  |
| GO:1901678 | BP | GO:1901678 | iron coordination entity transport | LCN2,ABCC5 | 2.28441296685985e-05 |  |
| GO:0005344 | MF | GO:0005344 | oxygen carrier activity | HBZ,CYGB | 2.40244887672792e-05 |  |
| GO:1990748 | BP | GO:1990748 | cellular detoxification | HBZ,CAT,CYGB | 2.55157317774115e-05 |  |
| GO:0034440 | BP | GO:0034440 | lipid oxidation | ALOX15,CYGB,CYP4F2 | 2.61858453780334e-05 |  |
| GO:0046916 | BP | GO:0046916 | cellular transition metal ion homeostasis | LCN2,TMPRSS6,MT2A | 2.61858453780334e-05 |  |
| GO:0015671 | BP | GO:0015671 | oxygen transport | HBZ,CYGB | 3.01071281352894e-05 |  |
| GO:0097237 | BP | GO:0097237 | cellular response to toxic substance | HBZ,CAT,CYGB | 3.12039323163591e-05 |  |
| GO:0061687 | BP | GO:0061687 | detoxification of inorganic compound | CAT,MT2A | 3.8364926817646e-05 |  |
| GO:0055076 | BP | GO:0055076 | transition metal ion homeostasis | LCN2,TMPRSS6,MT2A | 4.893076607052e-05 |  |
| GO:0015669 | BP | GO:0015669 | gas transport | HBZ,CYGB | 5.78581620695383e-05 |  |
| GO:0042744 | BP | GO:0042744 | hydrogen peroxide catabolic process | HBZ,CAT | 0.000108707950135841 |  |
| GO:0030258 | BP | GO:0030258 | lipid modification | ALOX15,CYGB,CYP4F2 | 0.000159681919970726 |  |
| GO:0019825 | MF | GO:0019825 | oxygen binding | HBZ,CYGB | 0.000194217447623396 |  |
| GO:0016709 | MF | GO:0016709 | oxidoreductase activity, acting on paired donors, with incorporation or reduction of molecular oxygen, NAD(P)H as one donor, and incorporation of one atom of oxygen | CYP4F2,CYP7B1 | 0.000204380247562955 |  |
| GO:0016712 | MF | GO:0016712 | oxidoreductase activity, acting on paired donors, with incorporation or reduction of molecular oxygen, reduced flavin or flavoprotein as one donor, and incorporation of one atom of oxygen | CYP4F2,CYP7B1 | 0.000204380247562955 |  |
| GO:0042743 | BP | GO:0042743 | hydrogen peroxide metabolic process | HBZ,CAT | 0.000368496953356848 |  |
| GO:0006826 | BP | GO:0006826 | iron ion transport | LCN2,ABCC5 | 0.000409838287233278 |  |
| GO:0019369 | BP | GO:0019369 | arachidonic acid metabolic process | ALOX15,CYP4F2 | 0.000409838287233278 |  |
| GO:0046686 | BP | GO:0046686 | response to cadmium ion | CAT,MT2A | 0.00043859911980405 |  |
| GO:0006879 | BP | GO:0006879 | cellular iron ion homeostasis | LCN2,TMPRSS6 | 0.000530623181504479 |  |
| GO:1901568 | BP | GO:1901568 | fatty acid derivative metabolic process | ALOX15,CYP4F2 | 0.000740360206539358 |  |
| GO:0010038 | BP | GO:0010038 | response to metal ion | ALOX15,CAT,MT2A | 0.000744188879037879 |  |
| GO:0140104 | MF | GO:0140104 | molecular carrier activity | HBZ,CYGB | 0.000880643162840937 |  |
| GO:0055072 | BP | GO:0055072 | iron ion homeostasis | LCN2,TMPRSS6 | 0.000941095337527915 |  |
| GO:0006631 | BP | GO:0006631 | fatty acid metabolic process | ALOX15,CYGB,CYP4F2 | 0.00101029970345818 |  |
| GO:0006875 | BP | GO:0006875 | cellular metal ion homeostasis | LCN2,TMPRSS6,MT2A | 0.00116342318249411 |  |
| GO:0009410 | BP | GO:0009410 | response to xenobiotic stimulus | ABCC5,CAT,CYP4F2 | 0.00126201746360522 |  |
| GO:0000041 | BP | GO:0000041 | transition metal ion transport | LCN2,ABCC5 | 0.00131081225229656 |  |
| GO:0004497 | MF | GO:0004497 | monooxygenase activity | CYP4F2,CYP7B1 | 0.00137764701366483 |  |
| GO:0032963 | BP | GO:0032963 | collagen metabolic process | TMPRSS6,CYGB | 0.00138672940881348 |  |
| GO:0001676 | BP | GO:0001676 | long-chain fatty acid metabolic process | ALOX15,CYP4F2 | 0.00149117041976867 |  |
| GO:0033559 | BP | GO:0033559 | unsaturated fatty acid metabolic process | ALOX15,CYP4F2 | 0.00159927752283803 |  |
| GO:0006805 | BP | GO:0006805 | xenobiotic metabolic process | ABCC5,CYP4F2 | 0.00168275600957922 |  |
| GO:0030003 | BP | GO:0030003 | cellular cation homeostasis | LCN2,TMPRSS6,MT2A | 0.00191051603325597 |  |
| GO:0006690 | BP | GO:0006690 | icosanoid metabolic process | ALOX15,CYP4F2 | 0.00191537077833893 |  |
| GO:0008203 | BP | GO:0008203 | cholesterol metabolic process | CAT,CYP7B1 | 0.00232419213946015 |  |
| GO:1902652 | BP | GO:1902652 | secondary alcohol metabolic process | CAT,CYP7B1 | 0.002664402290366 |  |
| GO:0016125 | BP | GO:0016125 | sterol metabolic process | CAT,CYP7B1 | 0.00284282932103218 |  |
| GO:0120254 | BP | GO:0120254 | olefinic compound metabolic process | ALOX15,CYP4F2 | 0.00306421977481777 |  |
| GO:0071466 | BP | GO:0071466 | cellular response to xenobiotic stimulus | ABCC5,CYP4F2 | 0.00398569860552931 |  |
| GO:0016705 | MF | GO:0016705 | oxidoreductase activity, acting on paired donors, with incorporation or reduction of molecular oxygen | CYP4F2,CYP7B1 | 0.00400966291876933 |  |
| GO:0071248 | BP | GO:0071248 | cellular response to metal ion | ALOX15,MT2A | 0.00464808010317718 |  |
| GO:0006646 | BP | GO:0006646 | phosphatidylethanolamine biosynthetic process | ALOX15 | 0.00527884349777841 |  |
| GO:0010764 | BP | GO:0010764 | negative regulation of fibroblast migration | CYGB | 0.00527884349777841 |  |
| GO:0042939 | BP | GO:0042939 | tripeptide transport | ABCC5 | 0.00527884349777841 |  |
| GO:0097267 | BP | GO:0097267 | omega-hydroxylase P450 pathway | CYP4F2 | 0.00527884349777841 |  |
| GO:1901523 | BP | GO:1901523 | icosanoid catabolic process | CYP4F2 | 0.00527884349777841 |  |
| GO:0015232 | MF | GO:0015232 | heme transmembrane transporter activity | ABCC5 | 0.00541343943692785 |  |
| GO:0031720 | MF | GO:0031720 | haptoglobin binding | HBZ | 0.00541343943692785 |  |
| GO:0043225 | MF | GO:0043225 | ATPase-coupled inorganic anion transmembrane transporter activity | ABCC5 | 0.00541343943692785 |  |
| GO:0031838 | CC | GO:0031838 | haptoglobin-hemoglobin complex | HBZ | 0.00552373830640507 |  |
| GO:0072330 | BP | GO:0072330 | monocarboxylic acid biosynthetic process | ALOX15,CYP7B1 | 0.0056053820681993 |  |
| GO:0015886 | BP | GO:0015886 | heme transport | ABCC5 | 0.00580534598891358 |  |
| GO:0019372 | BP | GO:0019372 | lipoxygenase pathway | ALOX15 | 0.00580534598891358 |  |
| GO:0035673 | MF | GO:0035673 | oligopeptide transmembrane transporter activity | ABCC5 | 0.0059533300897151 |  |
| GO:0005833 | CC | GO:0005833 | hemoglobin complex | HBZ | 0.00602453207299436 |  |
| GO:0071241 | BP | GO:0071241 | cellular response to inorganic substance | ALOX15,MT2A | 0.00616751191096639 |  |
| GO:0014854 | BP | GO:0014854 | response to inactivity | CAT | 0.00633159765840885 |  |
| GO:0032966 | BP | GO:0032966 | negative regulation of collagen biosynthetic process | CYGB | 0.00633159765840885 |  |
| GO:0097264 | BP | GO:0097264 | self proteolysis | TMPRSS6 | 0.00633159765840885 |  |
| GO:0072593 | BP | GO:0072593 | reactive oxygen species metabolic process | HBZ,CAT | 0.00648466241496691 |  |
| GO:0005527 | MF | GO:0005527 | macrolide binding | LCN2 | 0.00649295696659991 |  |
| GO:0006855 | BP | GO:0006855 | xenobiotic transmembrane transport | ABCC5 | 0.00685759861248347 |  |
| GO:0009650 | BP | GO:0009650 | UV protection | CAT | 0.00685759861248347 |  |
| GO:0010713 | BP | GO:0010713 | negative regulation of collagen metabolic process | CYGB | 0.00685759861248347 |  |
| GO:0042363 | BP | GO:0042363 | fat-soluble vitamin catabolic process | CYP4F2 | 0.00685759861248347 |  |
| GO:0072537 | BP | GO:0072537 | fibroblast activation | CYGB | 0.00685759861248347 |  |
| GO:1901617 | BP | GO:1901617 | organic hydroxy compound biosynthetic process | ALOX15,CYP7B1 | 0.00702963605072179 |  |
| GO:0015670 | BP | GO:0015670 | carbon dioxide transport | HBZ | 0.00738334895731518 |  |
| GO:0030213 | BP | GO:0030213 | hyaluronan biosynthetic process | ABCC5 | 0.00738334895731518 |  |
| GO:0008559 | MF | GO:0008559 | ABC-type xenobiotic transporter activity | ABCC5 | 0.00757141985086585 |  |
| GO:0015562 | MF | GO:0015562 | efflux transmembrane transporter activity | ABCC5 | 0.00757141985086585 |  |
| GO:0001977 | BP | GO:0001977 | renal system process involved in regulation of blood volume | CYP4F2 | 0.00790884879904485 |  |
| GO:0009111 | BP | GO:0009111 | vitamin catabolic process | CYP4F2 | 0.00790884879904485 |  |
| GO:0010273 | BP | GO:0010273 | detoxification of copper ion | MT2A | 0.00790884879904485 |  |
| GO:0033147 | BP | GO:0033147 | negative regulation of intracellular estrogen receptor signaling pathway | CYP7B1 | 0.00790884879904485 |  |
| GO:0035358 | BP | GO:0035358 | regulation of peroxisome proliferator activated receptor signaling pathway | ALOX15 | 0.00790884879904485 |  |
| GO:0035672 | BP | GO:0035672 | oligopeptide transmembrane transport | ABCC5 | 0.00790884879904485 |  |
| GO:0042182 | BP | GO:0042182 | ketone catabolic process | CYP4F2 | 0.00790884879904485 |  |
| GO:0060099 | BP | GO:0060099 | regulation of phagocytosis, engulfment | ALOX15 | 0.00790884879904485 |  |
| GO:1905153 | BP | GO:1905153 | regulation of membrane invagination | ALOX15 | 0.00790884879904485 |  |
| GO:1990169 | BP | GO:1990169 | stress response to copper ion | MT2A | 0.00790884879904485 |  |
| GO:1904680 | MF | GO:1904680 | peptide transmembrane transporter activity | ABCC5 | 0.00811025608723759 |  |
| GO:0030522 | BP | GO:0030522 | intracellular receptor signaling pathway | ALOX15,CYP7B1 | 0.00824001878708627 |  |
| GO:0006878 | BP | GO:0006878 | cellular copper ion homeostasis | MT2A | 0.00843409824377217 |  |
| GO:0006857 | BP | GO:0006857 | oligopeptide transport | ABCC5 | 0.0089590973975584 |  |
| GO:0009642 | BP | GO:0009642 | response to light intensity | CAT | 0.0089590973975584 |  |
| GO:0020027 | BP | GO:0020027 | hemoglobin metabolic process | CAT | 0.0089590973975584 |  |
| GO:0033189 | BP | GO:0033189 | response to vitamin A | CAT | 0.0089590973975584 |  |
| GO:0008392 | MF | GO:0008392 | arachidonic acid epoxygenase activity | CYP4F2 | 0.00918713872060817 |  |
| GO:0046942 | BP | GO:0046942 | carboxylic acid transport | ABCC5,CYP4F2 | 0.0097308788292495 |  |
| GO:0019373 | BP | GO:0019373 | epoxygenase P450 pathway | CYP4F2 | 0.0100083452563562 |  |
| GO:0043652 | BP | GO:0043652 | engulfment of apoptotic cell | ALOX15 | 0.0100083452563562 |  |
| GO:0055070 | BP | GO:0055070 | copper ion homeostasis | MT2A | 0.0100083452563562 |  |
| GO:0097501 | BP | GO:0097501 | stress response to metal ion | MT2A | 0.0100083452563562 |  |
| GO:1901569 | BP | GO:1901569 | fatty acid derivative catabolic process | CYP4F2 | 0.0100083452563562 |  |
| GO:0001666 | BP | GO:0001666 | response to hypoxia | CAT,CYGB | 0.0101212418567588 |  |
| GO:0010288 | BP | GO:0010288 | response to lead ion | CAT | 0.0105325941732927 |  |
| GO:0035461 | BP | GO:0035461 | vitamin transmembrane transport | ABCC5 | 0.0105325941732927 |  |
| GO:0055093 | BP | GO:0055093 | response to hyperoxia | CAT | 0.0105325941732927 |  |
| GO:0034774 | CC | GO:0034774 | secretory granule lumen | LCN2,CAT | 0.0108120630629197 |  |
| GO:0036293 | BP | GO:0036293 | response to decreased oxygen levels | CAT,CYGB | 0.0109909249460766 |  |
| GO:0060205 | CC | GO:0060205 | cytoplasmic vesicle lumen | LCN2,CAT | 0.0110059072388333 |  |
| GO:0032305 | BP | GO:0032305 | positive regulation of icosanoid secretion | CYP4F2 | 0.0110565932231402 |  |
| GO:0034755 | BP | GO:0034755 | iron ion transmembrane transport | ABCC5 | 0.0110565932231402 |  |
| GO:0043651 | BP | GO:0043651 | linoleic acid metabolic process | ALOX15 | 0.0110565932231402 |  |
| GO:0031983 | CC | GO:0031983 | vesicle lumen | LCN2,CAT | 0.0111360037568506 |  |
| GO:0008391 | MF | GO:0008391 | arachidonic acid monooxygenase activity | CYP4F2 | 0.0113377478311615 |  |
| GO:0015216 | MF | GO:0015216 | purine nucleotide transmembrane transporter activity | ABCC5 | 0.0113377478311615 |  |
| GO:0032303 | BP | GO:0032303 | regulation of icosanoid secretion | CYP4F2 | 0.0115803425117625 |  |
| GO:0032891 | BP | GO:0032891 | negative regulation of organic acid transport | CYP4F2 | 0.0115803425117625 |  |
| GO:0008202 | BP | GO:0008202 | steroid metabolic process | CAT,CYP7B1 | 0.011963703841929 |  |
| GO:0015849 | BP | GO:0015849 | organic acid transport | ABCC5,CYP4F2 | 0.0120346119350175 |  |
| GO:0046394 | BP | GO:0046394 | carboxylic acid biosynthetic process | ALOX15,CYP7B1 | 0.012176994651499 |  |
| GO:0016053 | BP | GO:0016053 | organic acid biosynthetic process | ALOX15,CYP7B1 | 0.0123201315590314 |  |
| GO:0003091 | BP | GO:0003091 | renal water homeostasis | CYP4F2 | 0.0126270922285953 |  |
| GO:0035357 | BP | GO:0035357 | peroxisome proliferator activated receptor signaling pathway | ALOX15 | 0.0126270922285953 |  |
| GO:0071294 | BP | GO:0071294 | cellular response to zinc ion | MT2A | 0.0126270922285953 |  |
| GO:1903792 | BP | GO:1903792 | negative regulation of anion transport | CYP4F2 | 0.0126270922285953 |  |
| GO:0016702 | MF | GO:0016702 | oxidoreductase activity, acting on single donors with incorporation of molecular oxygen, incorporation of two atoms of oxygen | ALOX15 | 0.0129479466254574 |  |
| GO:0070482 | BP | GO:0070482 | response to oxygen levels | CAT,CYGB | 0.0129735427814909 |  |
| GO:0003071 | BP | GO:0003071 | renal system process involved in regulation of systemic arterial blood pressure | CYP4F2 | 0.0131500928683385 |  |
| GO:0019755 | BP | GO:0019755 | one-carbon compound transport | HBZ | 0.0131500928683385 |  |
| GO:2000193 | BP | GO:2000193 | positive regulation of fatty acid transport | CYP4F2 | 0.0131500928683385 |  |
| GO:0016701 | MF | GO:0016701 | oxidoreductase activity, acting on single donors with incorporation of molecular oxygen | ALOX15 | 0.013484154824813 |  |
| GO:0070330 | MF | GO:0070330 | aromatase activity | CYP4F2 | 0.013484154824813 |  |
| GO:0051238 | BP | GO:0051238 | sequestering of metal ion | LCN2 | 0.0136728441699234 |  |
| GO:0060740 | BP | GO:0060740 | prostate gland epithelium morphogenesis | CYP7B1 | 0.0136728441699234 |  |
| GO:0016324 | CC | GO:0016324 | apical plasma membrane | ABCC5,CYP4F2 | 0.0139537743256043 |  |
| GO:0072349 | MF | GO:0072349 | modified amino acid transmembrane transporter activity | ABCC5 | 0.0140201008481603 |  |
| GO:0036296 | BP | GO:0036296 | response to increased oxygen levels | CAT | 0.0141953462390183 |  |
| GO:0051503 | BP | GO:0051503 | adenine nucleotide transport | ABCC5 | 0.0141953462390183 |  |
| GO:0071280 | BP | GO:0071280 | cellular response to copper ion | MT2A | 0.0141953462390183 |  |
| GO:0022884 | MF | GO:0022884 | macromolecule transmembrane transporter activity | ABCC5 | 0.0145557848094513 |  |
| GO:0001655 | BP | GO:0001655 | urogenital system development | CAT,CYP7B1 | 0.0147117401799451 |  |
| GO:0015868 | BP | GO:0015868 | purine ribonucleotide transport | ABCC5 | 0.0147175991812522 |  |
| GO:0046337 | BP | GO:0046337 | phosphatidylethanolamine metabolic process | ALOX15 | 0.0147175991812522 |  |
| GO:0060512 | BP | GO:0060512 | prostate gland morphogenesis | CYP7B1 | 0.0147175991812522 |  |
| GO:0015215 | MF | GO:0015215 | nucleotide transmembrane transporter activity | ABCC5 | 0.0150912068225945 |  |
| GO:0015865 | BP | GO:0015865 | purine nucleotide transport | ABCC5 | 0.0152396031022157 |  |
| GO:0042759 | BP | GO:0042759 | long-chain fatty acid biosynthetic process | ALOX15 | 0.0152396031022157 |  |
| GO:0006066 | BP | GO:0006066 | alcohol metabolic process | CAT,CYP7B1 | 0.0152600251869427 |  |
| GO:0015711 | BP | GO:0015711 | organic anion transport | ABCC5,CYP4F2 | 0.0156571017114663 |  |
| GO:0042910 | MF | GO:0042910 | xenobiotic transmembrane transporter activity | ABCC5 | 0.016695902311582 |  |
| GO:2000191 | BP | GO:2000191 | regulation of fatty acid transport | CYP4F2 | 0.0173251306837988 |  |
| GO:0015605 | MF | GO:0015605 | organophosphate ester transmembrane transporter activity | ABCC5 | 0.0177643916498876 |  |
| GO:0046486 | BP | GO:0046486 | glycerolipid metabolic process | ALOX15,CAT | 0.0178787458744087 |  |
| GO:0000038 | BP | GO:0000038 | very long-chain fatty acid metabolic process | CYP4F2 | 0.0183664030894353 |  |
| GO:0006691 | BP | GO:0006691 | leukotriene metabolic process | CYP4F2 | 0.0183664030894353 |  |
| GO:0030212 | BP | GO:0030212 | hyaluronan metabolic process | ABCC5 | 0.0183664030894353 |  |
| GO:0033146 | BP | GO:0033146 | regulation of intracellular estrogen receptor signaling pathway | CYP7B1 | 0.0183664030894353 |  |
| GO:0006699 | BP | GO:0006699 | bile acid biosynthetic process | CYP7B1 | 0.0188866668147428 |  |
| GO:0045177 | CC | GO:0045177 | apical part of cell | ABCC5,CYP4F2 | 0.0191573075170096 |  |
| GO:0042887 | MF | GO:0042887 | amide transmembrane transporter activity | ABCC5 | 0.0193651664492359 |  |
| GO:0033144 | BP | GO:0033144 | negative regulation of intracellular steroid hormone receptor signaling pathway | CYP7B1 | 0.0194066823620932 |  |
| GO:0071276 | BP | GO:0071276 | cellular response to cadmium ion | MT2A | 0.0194066823620932 |  |
| GO:0006862 | BP | GO:0006862 | nucleotide transport | ABCC5 | 0.0199264498367233 |  |
| GO:0072337 | BP | GO:0072337 | modified amino acid transport | ABCC5 | 0.0199264498367233 |  |
| GO:0008395 | MF | GO:0008395 | steroid hydroxylase activity | CYP7B1 | 0.0204310448349618 |  |
| GO:1901661 | BP | GO:1901661 | quinone metabolic process | CYP4F2 | 0.0204459693438332 |  |
| GO:0001503 | BP | GO:0001503 | ossification | ALOX15,CAT | 0.0204974727336109 |  |
| GO:0006979 | BP | GO:0006979 | response to oxidative stress | CAT,CYGB | 0.0209489963986787 |  |
| GO:0006882 | BP | GO:0006882 | cellular zinc ion homeostasis | MT2A | 0.0209652409885834 |  |
| GO:0010762 | BP | GO:0010762 | regulation of fibroblast migration | CYGB | 0.0209652409885834 |  |
| GO:0032965 | BP | GO:0032965 | regulation of collagen biosynthetic process | CYGB | 0.0209652409885834 |  |
| GO:0046688 | BP | GO:0046688 | response to copper ion | MT2A | 0.021484264876094 |  |
| GO:0042060 | BP | GO:0042060 | wound healing | ALOX15,CYP4F2 | 0.0216802650137942 |  |
| GO:0055069 | BP | GO:0055069 | zinc ion homeostasis | MT2A | 0.0220030411114476 |  |
| GO:0006775 | BP | GO:0006775 | fat-soluble vitamin metabolic process | CYP4F2 | 0.0235578849548017 |  |
| GO:0010712 | BP | GO:0010712 | regulation of collagen metabolic process | CYGB | 0.0235578849548017 |  |
| GO:0030574 | BP | GO:0030574 | collagen catabolic process | TMPRSS6 | 0.0235578849548017 |  |
| GO:0032369 | BP | GO:0032369 | negative regulation of lipid transport | CYP4F2 | 0.0245932111810018 |  |
| GO:0051180 | BP | GO:0051180 | vitamin transport | ABCC5 | 0.0245932111810018 |  |
| GO:0005782 | CC | GO:0005782 | peroxisomal matrix | CAT | 0.0248873360669896 |  |
| GO:0031907 | CC | GO:0031907 | microbody lumen | CAT | 0.0248873360669896 |  |
| GO:0032309 | BP | GO:0032309 | icosanoid secretion | CYP4F2 | 0.0251105037079531 |  |
| GO:0032892 | BP | GO:0032892 | positive regulation of organic acid transport | CYP4F2 | 0.0251105037079531 |  |
| GO:0008206 | BP | GO:0008206 | bile acid metabolic process | CYP7B1 | 0.0256275493172299 |  |
| GO:0032964 | BP | GO:0032964 | collagen biosynthetic process | CYGB | 0.0261443481136037 |  |
| GO:1901570 | BP | GO:1901570 | fatty acid derivative biosynthetic process | ALOX15 | 0.0261443481136037 |  |
| GO:0140359 | MF | GO:0140359 | ABC-type transporter activity | ABCC5 | 0.0262747603497616 |  |
| GO:0030850 | BP | GO:0030850 | prostate gland development | CYP7B1 | 0.0266609002018047 |  |
| GO:0043277 | BP | GO:0043277 | apoptotic cell clearance | ALOX15 | 0.0266609002018047 |  |
| GO:1903793 | BP | GO:1903793 | positive regulation of anion transport | CYP4F2 | 0.0266609002018047 |  |
| GO:0005048 | MF | GO:0005048 | signal sequence binding | CAT | 0.0268044482351173 |  |
| GO:0015932 | MF | GO:0015932 | nucleobase-containing compound transmembrane transporter activity | ABCC5 | 0.0268044482351173 |  |
| GO:0006636 | BP | GO:0006636 | unsaturated fatty acid biosynthetic process | ALOX15 | 0.0271772056865272 |  |
| GO:0010043 | BP | GO:0010043 | response to zinc ion | MT2A | 0.0271772056865272 |  |
| GO:0030838 | BP | GO:0030838 | positive regulation of actin filament polymerization | ALOX15 | 0.0276932646724238 |  |
| GO:0030520 | BP | GO:0030520 | intracellular estrogen receptor signaling pathway | CYP7B1 | 0.0282090772641092 |  |
| GO:0050661 | MF | GO:0050661 | NADP binding | CAT | 0.0283919563016276 |  |
| GO:0010761 | BP | GO:0010761 | fibroblast migration | CYGB | 0.0287246435661603 |  |
| GO:0055078 | BP | GO:0055078 | sodium ion homeostasis | CYP4F2 | 0.0287246435661603 |  |
| GO:1901505 | MF | GO:1901505 | carbohydrate derivative transmembrane transporter activity | ABCC5 | 0.028920607503379 |  |
| GO:0042908 | BP | GO:0042908 | xenobiotic transport | ABCC5 | 0.029239963683114 |  |
| GO:0071715 | BP | GO:0071715 | icosanoid transport | CYP4F2 | 0.029239963683114 |  |
| GO:0072348 | BP | GO:0072348 | sulfur compound transport | ABCC5 | 0.029239963683114 |  |
| GO:1901682 | MF | GO:1901682 | sulfur compound transmembrane transporter activity | ABCC5 | 0.0294489998161963 |  |
| GO:0033619 | BP | GO:0033619 | membrane protein proteolysis | TMPRSS6 | 0.0302698657796827 |  |
| GO:0035580 | CC | GO:0035580 | specific granule lumen | LCN2 | 0.030776717193586 |  |
| GO:0005778 | CC | GO:0005778 | peroxisomal membrane | CAT | 0.0317551622599954 |  |
| GO:0031903 | CC | GO:0031903 | microbody membrane | CAT | 0.0317551622599954 |  |
| GO:0002820 | BP | GO:0002820 | negative regulation of adaptive immune response | ALOX15 | 0.0323267203469784 |  |
| GO:0022617 | BP | GO:0022617 | extracellular matrix disassembly | TMPRSS6 | 0.0323267203469784 |  |
| GO:0030514 | BP | GO:0030514 | negative regulation of BMP signaling pathway | TMPRSS6 | 0.032840320092031 |  |
| GO:0070542 | BP | GO:0070542 | response to fatty acid | CAT | 0.032840320092031 |  |
| GO:0050891 | BP | GO:0050891 | multicellular organismal water homeostasis | CYP4F2 | 0.0333536744868862 |  |
| GO:0048247 | BP | GO:0048247 | lymphocyte chemotaxis | CYP7B1 | 0.0338667836357338 |  |
| GO:1905953 | BP | GO:1905953 | negative regulation of lipid localization | CYP4F2 | 0.0338667836357338 |  |
| GO:0030104 | BP | GO:0030104 | water homeostasis | CYP4F2 | 0.0359167698534465 |  |
| GO:0014823 | BP | GO:0014823 | response to activity | CAT | 0.0364286543337199 |  |
| GO:0016811 | MF | GO:0016811 | hydrolase activity, acting on carbon-nitrogen (but not peptide) bonds, in linear amides | CAT | 0.0373438943712527 |  |
| GO:0033143 | BP | GO:0033143 | regulation of intracellular steroid hormone receptor signaling pathway | CYP7B1 | 0.0374516895331084 |  |
| GO:0032890 | BP | GO:0032890 | regulation of organic acid transport | CYP4F2 | 0.0389844094874782 |  |
| GO:0006024 | BP | GO:0006024 | glycosaminoglycan biosynthetic process | ABCC5 | 0.0400050021049347 |  |
| GO:1901616 | BP | GO:1901616 | organic hydroxy compound catabolic process | CYP4F2 | 0.0400050021049347 |  |
| GO:0140115 | BP | GO:0140115 | export across plasma membrane | ABCC5 | 0.0405149325191997 |  |
| GO:1901264 | BP | GO:1901264 | carbohydrate derivative transport | ABCC5 | 0.0405149325191997 |  |
| GO:0005758 | CC | GO:0005758 | mitochondrial intermembrane space | CAT | 0.0414908324652944 |  |
| GO:0014068 | BP | GO:0014068 | positive regulation of phosphatidylinositol 3-kinase signaling | CAT | 0.041534062077039 |  |
| GO:0006023 | BP | GO:0006023 | aminoglycan biosynthetic process | ABCC5 | 0.0420432614277927 |  |
| GO:0033273 | BP | GO:0033273 | response to vitamin | CAT | 0.0445856080400099 |  |
| GO:0010232 | BP | GO:0010232 | vascular transport | ABCC5 | 0.0450933480580575 |  |
| GO:0071277 | BP | GO:0071277 | cellular response to calcium ion | ALOX15 | 0.0450933480580575 |  |
| GO:0150104 | BP | GO:0150104 | transport across blood-brain barrier | ABCC5 | 0.0450933480580575 |  |
| GO:0005546 | MF | GO:0005546 | phosphatidylinositol-4,5-bisphosphate binding | ALOX15 | 0.0451809433041221 |  |
| GO:0032370 | BP | GO:0032370 | positive regulation of lipid transport | CYP4F2 | 0.0456008452157647 |  |
| GO:0031970 | CC | GO:0031970 | organelle envelope lumen | CAT | 0.0463255512403223 |  |
| GO:0032273 | BP | GO:0032273 | positive regulation of protein polymerization | ALOX15 | 0.0476284073078636 |  |
| GO:0006835 | BP | GO:0006835 | dicarboxylic acid transport | ABCC5 | 0.0481346917120378 |  |
| GO:0044070 | BP | GO:0044070 | regulation of anion transport | CYP4F2 | 0.0481346917120378 |  |
| GO:0051781 | BP | GO:0051781 | positive regulation of cell division | CAT | 0.0481346917120378 |  |
| GO:0070301 | BP | GO:0070301 | cellular response to hydrogen peroxide | CAT | 0.0481346917120378 |  |
| GO:0031234 | CC | GO:0031234 | extrinsic component of cytoplasmic side of plasma membrane | ALOX15 | 0.0487346705404686 |  |
| GO:0032088 | BP | GO:0032088 | negative regulation of NF-kappaB transcription factor activity | CAT | 0.0491465338993928 |  |
| GO:1903510 | BP | GO:1903510 | mucopolysaccharide metabolic process | ABCC5 | 0.0491465338993928 |  |
| GO:0051213 | MF | GO:0051213 | dioxygenase activity | ALOX15 | 0.0493371805780872 |  |
| GO:0003073 | BP | GO:0003073 | regulation of systemic arterial blood pressure | CYP4F2 | 0.0496520918886003 |  |

**Table S5**

|  | Category | ID | Term | Genes | adj_pval | pvalue |
| --- | --- | --- | --- | --- | --- | --- |
| 1 | KEGG | hsa00590 | Arachidonic acid metabolism | ALOX15, CYP4F2 | 0.0211305754131224 | 0.000960480700596473 |
| 2 | KEGG | hsa00120 | Primary bile acid biosynthesis | CYP7B1 | 0.0961054678929956 | 0.0133781063210365 |
| 3 | KEGG | hsa00591 | Linoleic acid metabolism | ALOX15 | 0.0961054678929956 | 0.0235046369802465 |
| 4 | KEGG | hsa01523 | Antifolate resistance | ABCC5 | 0.0961054678929956 | 0.0235046369802465 |
| 5 | KEGG | hsa00630 | Glyoxylate and dicarboxylate metabolism | CAT | 0.0961054678929956 | 0.0242798969939855 |
| 6 | KEGG | hsa00380 | Tryptophan metabolism | CAT | 0.0961054678929956 | 0.0327729995337025 |
| 7 | KEGG | hsa04216 | Ferroptosis | ALOX15 | 0.0961054678929956 | 0.0327729995337025 |
| 8 | KEGG | hsa02010 | ABC transporters | ABCC5 | 0.0961054678929956 | 0.0350782723321882 |
| 9 | KEGG | hsa04978 | Mineral absorption | MT2A | 0.0961054678929956 | 0.047293691816144 |
| 10 | KEGG | hsa00140 | Steroid hormone biosynthesis | CYP7B1 | 0.0961054678929956 | 0.0480527339464978 |
| 11 | KEGG | hsa04213 | Longevity regulating pathway - multiple species | CAT | 0.0961054678929956 | 0.0480527339464978 |

**Table S6**

|  | ID | Description | setSize | enrichmentScore | NES | pvalue | p.adjust | qvalue | rank | leading_edge | core_enrichment |
| --- | --- | --- | --- | --- | --- | --- | --- | --- | --- | --- | --- |
| KEGG_OXIDATIVE_PHOSPHORYLATION | KEGG_OXIDATIVE_PHOSPHORYLATION | KEGG_OXIDATIVE_PHOSPHORYLATION | 126 | -0.75680261 | -3.707529956 | 1e-10 | 1.69090909090909e-09 | 6.02870813397129e-10 | 5447 | tags=72%, list=13%, signal=63% | LHPP/MT-ND3/ATP6V1G1/ATP6V0B/COX6B2/TCIRG1/MT-CO1/UQCRC2/NDUFB2/NDUFB5/UQCRB/ATP6V0C/PPA2/MT-CO3/ATP6V0D1/NDUFS4/ATP5PB/PPA1/SDHA/NDUFB1/COX7A1/UQCRC1/COX6C/NDUFS8/COX17/MT-CO2/ATP5MC2/SDHC/NDUFA11/NDUFA6/ATP5F1E/NDUFS3/ATP5PO/ATP5F1A/NDUFS7/NDUFS6/COX7A2L/NDUFS2/NDUFB6/ATP5ME/COX7C/NDUFA3/ATP6AP1/NDUFA8/NDUFV2/NDUFC1/NDUFAB1/UQCRHL/ATP6V1H/ATP5F1C/COX4I1/ATP5MC3/NDUFB3/ATP6V1F/NDUFA4L2/NDUFB7/ATP5MC1/NDUFB4/NDUFB10/ATP5PD/NDUFB8/NDUFA4/ATP5F1D/ATP5F1B/NDUFB9/NDUFA9/ATP5MF/NDUFC2/UQCRQ/NDUFS5/UQCRFS1/CYC1/COX6B1/ATP5PF/COX8A/NDUFA7/ATP5MG/ATP6V0E1/ATP6V1D/NDUFA2/COX7A2/COX5B/ATP6V1E1/UQCR11/UQCRH/COX7B/SDHB/UQCR10/NDUFA1/COX5A/COX6A1 |
| KEGG_PARKINSONS_DISEASE | KEGG_PARKINSONS_DISEASE | KEGG_PARKINSONS_DISEASE | 125 | -0.748343215 | -3.656535443 | 1e-10 | 1.69090909090909e-09 | 6.02870813397129e-10 | 4348 | tags=68%, list=10%, signal=61% | MT-CO1/UQCRC2/NDUFB2/GPR37/NDUFB5/UQCRB/CASP9/UBE2J1/MT-CO3/NDUFS4/ATP5PB/HTRA2/SDHA/NDUFB1/COX7A1/UQCRC1/UBE2L6/COX6C/NDUFS8/SLC25A6/UBE2J2/MT-CO2/UBA1/ATP5MC2/SDHC/NDUFA6/ATP5F1E/NDUFS3/ATP5PO/ATP5F1A/NDUFS7/SLC25A5/UCHL1/NDUFS6/COX7A2L/NDUFS2/NDUFB6/COX7C/NDUFA3/NDUFA8/NDUFV2/NDUFC1/NDUFAB1/UQCRHL/ATP5F1C/COX4I1/ATP5MC3/NDUFB3/NDUFA4L2/NDUFB7/ATP5MC1/NDUFB4/NDUFB10/ATP5PD/NDUFB8/VDAC2/NDUFA4/ATP5F1D/ATP5F1B/VDAC1/NDUFB9/NDUFA9/NDUFC2/UQCRQ/NDUFS5/UQCRFS1/CYC1/PARK7/COX6B1/ATP5PF/COX8A/NDUFA7/VDAC2P5/NDUFA2/COX7A2/COX5B/UQCR11/UQCRH/COX7B/SDHB/UQCR10/NDUFA1/COX5A/COX6A1/UBE2L3 |
| KEGG_ALZHEIMERS_DISEASE | KEGG_ALZHEIMERS_DISEASE | KEGG_ALZHEIMERS_DISEASE | 161 | -0.664497075 | -3.396177497 | 1e-10 | 1.69090909090909e-09 | 6.02870813397129e-10 | 4760 | tags=57%, list=11%, signal=50% | COX6B2/BID/MAPT/MT-CO1/UQCRC2/NDUFB2/CHP1/NDUFB5/UQCRB/CASP9/GRIN2B/PLCB3/CDK5/NCSTN/MT-CO3/NDUFS4/CALM3/ATP5PB/LPL/SDHA/NDUFB1/COX7A1/UQCRC1/PSEN2/COX6C/NDUFS8/CAPN1/MT-CO2/FADD/ATP5MC2/SDHC/NDUFA6/ATP5F1E/NDUFS3/ATP5PO/ATP5F1A/NDUFS7/MAPK3/APH1A/NDUFS6/COX7A2L/NDUFS2/NDUFB6/COX7C/NDUFA3/GAPDH/NDUFA8/NDUFV2/NDUFC1/NDUFAB1/UQCRHL/APOE/PSENEN/ATP5F1C/COX4I1/ATP5MC3/HSD17B10/NDUFB3/NDUFA4L2/NDUFB7/ATP5MC1/NDUFB4/NDUFB10/ATP5PD/NDUFB8/NDUFA4/ATP5F1D/ATP5F1B/NDUFB9/NDUFA9/NDUFC2/UQCRQ/NDUFS5/UQCRFS1/CYC1/COX6B1/ATP5PF/BAD/COX8A/NDUFA7/NDUFA2/COX7A2/COX5B/UQCR11/UQCRH/COX7B/SDHB/UQCR10/NDUFA1/COX5A/COX6A1 |
| KEGG_HUNTINGTONS_DISEASE | KEGG_HUNTINGTONS_DISEASE | KEGG_HUNTINGTONS_DISEASE | 176 | -0.648254907 | -3.368756385 | 1e-10 | 1.69090909090909e-09 | 6.02870813397129e-10 | 4760 | tags=58%, list=11%, signal=52% | COX6B2/BBC3/POLR2D/HDAC1/MT-CO1/UQCRC2/AP2A1/NDUFB2/NDUFB5/SOD1/UQCRB/CASP9/GRIN2B/PLCB3/GPX1/TGM2/MT-CO3/NDUFS4/ATP5PB/SDHA/NDUFB1/COX7A1/UQCRC1/COX6C/NDUFS8/SLC25A6/DCTN1/DCTN2/MT-CO2/ATP5MC2/SDHC/NDUFA6/CREB3/ATP5F1E/POLR2C/AP2S1/NDUFS3/CLTB/ATP5PO/ATP5F1A/NDUFS7/POLR2E/SLC25A5/NDUFS6/COX7A2L/NDUFS2/POLR2F/BAX/NDUFB6/COX7C/NDUFA3/NDUFA8/NDUFV2/CLTA/NDUFC1/NDUFAB1/POLR2G/POLR2L/UQCRHL/ATP5F1C/COX4I1/ATP5MC3/NDUFB3/NDUFA4L2/NDUFB7/ATP5MC1/NDUFB4/NDUFB10/ATP5PD/NDUFB8/VDAC2/NDUFA4/ATP5F1D/ATP5F1B/VDAC1/NDUFB9/NDUFA9/PPARG/NDUFC2/POLR2I/UQCRQ/NDUFS5/UQCRFS1/CYC1/COX6B1/POLR2J/AP2M1/ATP5PF/COX8A/NDUFA7/VDAC2P5/NDUFA2/COX7A2/COX5B/UQCR11/UQCRH/COX7B/SDHB/UQCR10/NDUFA1/COX5A/COX6A1 |
| KEGG_RIBOSOME | KEGG_RIBOSOME | KEGG_RIBOSOME | 88 | -0.691859953 | -3.169773606 | 1e-10 | 1.69090909090909e-09 | 6.02870813397129e-10 | 9189 | tags=91%, list=22%, signal=71% | RPL22/RPL7/RPL5/RPL12/RPS27A/RPS3A/RPL4/RPL6/RPS6/RPL3/RSL24D1/RPL31/RPL30/RPL11/RPS13/RPL34/RPL23A/RPS10/RPL10A/RPL17/RPL23/RPS25/RPS24/RPS23/RPL32/RPLP2/RPS27L/RPL13/RPS27/RPS18/RPS20/RPS7/RPL13A/RPS8/RPL14/RPL10/RPL26/RPL36A/RPL41/RPL19/RPS3/RPS4X/RPL24/RPL38/RPS15A/RPS16/RPS28/RPL27A/RPSA/RPS17/RPL18/RPL35A/RPS15/RPL39/RPL18A/RPL9/RPL7A/RPS2/RPS19/RPLP1/RPS21/RPS5/RPL37A/RPS11/RPL15/RPL27/MRPL13/RPL28/RPL36/RPL29/RPLP0/RPL35/RPS29/RPL37/RPS9/RPL36AL/RPL22L1/RPL8/FAU/RPL26L1 |
| KEGG_PROTEASOME | KEGG_PROTEASOME | KEGG_PROTEASOME | 45 | -0.789571312 | -3.087507891 | 1e-10 | 1.69090909090909e-09 | 6.02870813397129e-10 | 2888 | tags=69%, list=7%, signal=64% | PSMB9/PSMA2/PSMD11/PSMC2/PSMA1/PSMD13/PSMA4/PSMA5/PSME2/PSMC5/PSMB7/POMP/PSMD7/PSMB3/PSMB8/PSMB4/PSME1/PSMC4/PSMB1/PSMB5/PSMD8/PSMC3/PSMB10/PSMB6/PSMA7/PSMC1/PSMB2/PSMD3/PSMD2/PSMD4/PSMA6 |
| KEGG_SPLICEOSOME | KEGG_SPLICEOSOME | KEGG_SPLICEOSOME | 126 | -0.592703946 | -2.903620579 | 1e-10 | 1.69090909090909e-09 | 6.02870813397129e-10 | 8312 | tags=52%, list=20%, signal=41% | BCAS2/HNRNPA1L2/HNRNPK/SF3B3/HSPA1B/SNRNP27/SRSF3/SYF2/RBM8A/PPIE/PRPF40B/THOC3/LSM5/DHX8/HSPA8/HNRNPM/PRPF6/SNRPF/SNW1/PRPF4/PRPF19/PPIL1/SNRPB2/CWC15/SNRNP40/SRSF9/U2AF2/EFTUD2/CCDC12/MAGOH/PRPF31/SNRPE/LSM6/SF3A2/ISY1/SNRPD1/PQBP1/LSM4/PCBP1/HSPA2/SNRPD2/EIF4A3/LSM7/SNRPA/XAB2/HNRNPC/SNU13/CTNNBL1/SNRPG/LSM2/PUF60/SNRPB/ALYREF/SNRPC/USP39/SF3B2/TXNL4A/LSM3/SART1/SF3B4/SF3B5/ZMAT2/SF3B6/PHF5A/BUD31 |
| KEGG_PYRIMIDINE_METABOLISM | KEGG_PYRIMIDINE_METABOLISM | KEGG_PYRIMIDINE_METABOLISM | 98 | -0.589168203 | -2.768356755 | 1e-10 | 1.69090909090909e-09 | 6.02870813397129e-10 | 7817 | tags=56%, list=19%, signal=46% | CMPK2/CANT1/TXNRD2/POLR3H/RRM1/POLA1/POLR1C/ENTPD8/POLR2K/POLR3C/POLR2H/DPYS/PRIM2/POLE3/DCTD/UCKL1/POLR2D/CDA/NME5/RRM2/UCK2/POLE2/POLR3GL/DUT/POLR3K/TYMP/NME3/POLA2/TYMS/UPB1/NUDT2/NME1-NME2/POLR1D/POLD2/DTYMK/TK1/UCK1/CTPS1/POLE4/POLR2C/PNP/POLR2E/POLD4/POLR2F/ITPA/NME2/UPP1/POLR2G/POLR2L/POLD1/POLR2I/NT5C/POLR2J/NME1/POLR1H |
| KEGG_CELL_CYCLE | KEGG_CELL_CYCLE | KEGG_CELL_CYCLE | 124 | -0.556603423 | -2.711431953 | 1e-10 | 1.69090909090909e-09 | 6.02870813397129e-10 | 7587 | tags=52%, list=18%, signal=43% | YWHAZ/YWHAB/ABL1/CDKN2B/TTK/CDKN1A/BUB1B/MCM3/CHEK1/CCNA2/FZR1/CDK1/SKP1/CDC6/CUL1/BUB1/ORC6/ORC1/MCM7/ANAPC10/CCNA1/GADD45B/MCM4/HDAC1/ESPL1/MCM6/YWHAQ/CCND1/SMC1B/CDC25A/CDC45/MAD2L1/CDK2/MAD1L1/PCNA/MCM5/E2F2/CCNB2/GADD45A/CCNB1/ZBTB17/CDC20/CDC25C/CDKN2A/CCND3/YWHAH/CDC26/PKMYT1/PLK1/CHEK2/MCM2/CCNE1/CDKN2D/CDK4/TGFB1/E2F1/CDK7/PTTG1/ANAPC11/RBX1/TFDP1/E2F4/YWHAE/CDKN2C/MAD2L2 |
| KEGG_LYSOSOME | KEGG_LYSOSOME | KEGG_LYSOSOME | 120 | -0.546437604 | -2.645636273 | 1e-10 | 1.69090909090909e-09 | 6.02870813397129e-10 | 7483 | tags=51%, list=18%, signal=42% | AP3D1/CLTCL1/HEXB/SORT1/CTNS/AP3S2/LAPTM4B/CLN3/GNPTG/NAGA/NAGPA/ATP6V0B/PPT1/AGA/TCIRG1/PSAP/GGA1/CTSW/AP1M1/SUMF1/ATP6V0C/GAA/NAGLU/HEXA/MAN2B1/ATP6V0D1/CTSL/CD68/CTSC/GNS/CTSD/CTSZ/PLA2G15/ARSB/LAPTM5/LAPTM4A/SMPD1/GALNS/GUSB/NPC2/AP3S1/GLB1/CLTB/SLC17A5/TPP1/ATP6AP1/CLTA/ACP2/CTSH/LAMP1/ATP6V1H/CTSB/NEU1/AP1B1/CD63/DNASE2/CTSA/GM2A/GLA/AP1S1/ACP5 |
| KEGG_SYSTEMIC_LUPUS_ERYTHEMATOSUS | KEGG_SYSTEMIC_LUPUS_ERYTHEMATOSUS | KEGG_SYSTEMIC_LUPUS_ERYTHEMATOSUS | 127 | -0.507737735 | -2.487853871 | 1e-10 | 1.69090909090909e-09 | 6.02870813397129e-10 | 12105 | tags=67%, list=29%, signal=48% | CD80/C7/H3C7/FCGR2A/ACTN3/SNRPD3/H2BC4/H2AC15/H4C8/H3C10/H2BC21/H2AZ2/H2BC11/H3-3A/GRIN2A/HLA-DMB/CTSG/C8G/C1S/H2BC7/H2AC12/H2BC3/H3-3B/IFNG/H4C3/ELANE/ACTN2/H4C6/H2BC14/H4C4/H2AC8/H3C8/H2AB1/H2BC13/H4C15/H2BC8/H2AC13/CD40/H4C14/H4C1/FCGR1A/HLA-DPA1/HLA-DRB1/C1QC/HLA-DRB5/HLA-DPB1/H2AC17/HLA-DRA/HLA-DMA/H3C11/H2AC16/C1QB/H3C12/H2AC20/H3-5/C3/H2AC19/ACTN4/H2AC18/GRIN2B/H2BC5/H3C1/C1QA/H2BC10/HLA-DQA2/MACROH2A1/H2AZ1/H2AC4/H4C5/SNRPD1/H4C2/H3C3/H3C2/C2/H2AC21/IL10/H2AJ/H2BC12/C1R/H2AX/TRIM21/SNRPB/H2AC14/H4C11/H4C12 |
| KEGG_PEROXISOME | KEGG_PEROXISOME | KEGG_PEROXISOME | 77 | -0.547666364 | -2.457122185 | 7.52172011929294e-10 | 1.16586661849041e-08 | 4.15674006592505e-09 | 7322 | tags=48%, list=18%, signal=40% | PEX10/PEX7/ACSL1/MPV17L/PEX2/PEX11B/CRAT/SLC27A2/DDO/PECR/ACSL3/ACOX2/PEX19/SOD1/MVK/PRDX5/ABCD1/ACOX3/MPV17/DHRS4/PEX14/IDH1/NUDT19/HACL1/PAOX/PEX16/HMGCL/PIPOX/PEX11G/GSTK1/PXMP2/ACAA1/ACOT8/ECH1/PMVK/IDH2/PRDX1 |
| KEGG_PATHOGENIC_ESCHERICHIA_COLI_INFECTION | KEGG_PATHOGENIC_ESCHERICHIA_COLI_INFECTION | KEGG_PATHOGENIC_ESCHERICHIA_COLI_INFECTION | 56 | -0.606517551 | -2.523109735 | 1.04250822052399e-09 | 1.49158868474971e-08 | 5.31805812898877e-09 | 7673 | tags=55%, list=18%, signal=45% | CLDN1/YWHAZ/ARPC5/ABL1/LY96/TUBA1A/CDC42/EZR/YWHAQ/CD14/WAS/TUBB6/HCLS1/TUBB2A/TUBA4A/ARPC2/ARPC5L/RHOA/ARPC1A/ACTB/ARPC4/ACTG1/ARPC1B/TUBB4A/TUBB/ARPC3/TUBA1B/TUBA1C/TUBB4B/TUBB3/TUBB2B |
| KEGG_OOCYTE_MEIOSIS | KEGG_OOCYTE_MEIOSIS | KEGG_OOCYTE_MEIOSIS | 109 | -0.476114916 | -2.291226277 | 3.29550727811165e-09 | 4.37831681234833e-08 | 1.56102976331604e-08 | 7587 | tags=43%, list=18%, signal=35% | YWHAZ/YWHAB/CPEB1/PRKACA/ADCY5/RPS6KA1/REC8/AURKA/CDK1/SKP1/PPP2CA/PPP2R5D/CUL1/BUB1/CALM2/ANAPC10/MAP2K1/ADCY2/ADCY8/ESPL1/CHP1/YWHAQ/SMC1B/MAD2L1/CDK2/CAMK2A/CCNB2/CCNB1/CDC20/CDC25C/CALM3/FBXO43/YWHAH/CDC26/PKMYT1/PPP1CA/PLK1/CCNE1/CAMK2B/MAPK3/PTTG1/ADCY3/ANAPC11/RBX1/PPP2R1A/YWHAE/MAD2L2 |
| KEGG_DNA_REPLICATION | KEGG_DNA_REPLICATION | KEGG_DNA_REPLICATION | 36 | -0.666849848 | -2.474425354 | 8.02191885099499e-09 | 9.8220518176769e-08 | 3.50192000290687e-08 | 10241 | tags=81%, list=25%, signal=61% | POLE/RFC5/RPA4/LIG1/RPA2/POLA1/RPA1/MCM3/SSBP1/RFC4/PRIM2/POLE3/MCM7/MCM4/MCM6/RNASEH2C/POLE2/PCNA/MCM5/POLA2/RPA3/POLD2/FEN1/MCM2/POLE4/POLD4/RFC2/RNASEH2A/POLD1 |
| KEGG_PURINE_METABOLISM | KEGG_PURINE_METABOLISM | KEGG_PURINE_METABOLISM | 158 | -0.423066286 | -2.150206101 | 8.44907683241024e-09 | 9.8220518176769e-08 | 3.50192000290687e-08 | 12830 | tags=58%, list=31%, signal=40% | ALLC/PDE11A/PDE4D/POLR2A/NPR1/AK7/NUDT5/PRIM1/PDE1B/ADPRM/NME7/PDE6B/GUCY2F/AMPD1/ADK/ADSS1/POLE/ENTPD6/PKLR/URAD/PDE1C/GMPS/NUDT9/ADCY10/ADCY1/PDE2A/CANT1/PAICS/POLR3H/RRM1/POLA1/POLR1C/ADCY5/ENTPD8/POLR2K/GART/IMPDH1/POLR3C/PDE6G/POLR2H/PRIM2/POLE3/GDA/PRPS1/PRPS2/ADCY2/GUCY1A2/POLR2D/AK2/ADCY8/ADA/NME5/RRM2/GUCY2C/IMPDH2/PDE1A/POLE2/POLR3GL/ATIC/PDE10A/POLR3K/PKM/HPRT1/NME3/ADSL/POLA2/NUDT2/NME1-NME2/POLR1D/POLD2/GMPR2/APRT/POLE4/POLR2C/AK4/PNP/POLR2E/POLD4/POLR2F/ITPA/NME2/POLR2G/POLR2L/ADCY3/PDE6D/POLD1/POLR2I/NT5C/POLR2J/NME1/DGUOK/POLR1H |
| KEGG_UBIQUITIN_MEDIATED_PROTEOLYSIS | KEGG_UBIQUITIN_MEDIATED_PROTEOLYSIS | KEGG_UBIQUITIN_MEDIATED_PROTEOLYSIS | 133 | -0.438002203 | -2.150926349 | 1.53012426075906e-08 | 1.67413595588933e-07 | 5.96890578500439e-08 | 5951 | tags=32%, list=14%, signal=27% | UBE2N/UBE2E2/FZR1/SKP1/CUL1/UBE2A/ANAPC10/DDB2/UBE2D2/UBE2J1/PIAS4/PRPF19/ELOB/UBE2QL1/PML/SOCS1/CDC20/UBOX5/UBE2C/KEAP1/SOCS3/UBE2NL/DDB1/CDC26/UBE2Z/UBE2L6/STUB1/RNF7/UBE2J2/UBA1/UBE2Q1/UBE2E1/MID1/SAE1/FBXO2/ANAPC11/RBX1/ELOC/UBE2S/UBE2M/UBE2L3/UBE2F |
| KEGG_GLUTATHIONE_METABOLISM | KEGG_GLUTATHIONE_METABOLISM | KEGG_GLUTATHIONE_METABOLISM | 45 | -0.613195221 | -2.397813922 | 1.81815807945577e-08 | 1.87876334877097e-07 | 6.69847713483706e-08 | 7277 | tags=62%, list=17%, signal=51% | ODC1/RRM1/GSTT2/GGCT/SMS/GGT1/LAP3/PGD/OPLAH/RRM2/GSS/GPX1/G6PD/GPX4/MGST2/IDH1/GPX7/MGST1/GSR/GSTP1/GPX3/MGST3/GSTK1/GSTZ1/GSTO1/TXNDC12/SRM/IDH2 |
| KEGG_RNA_POLYMERASE | KEGG_RNA_POLYMERASE | KEGG_RNA_POLYMERASE | 29 | -0.694858175 | -2.437186976 | 4.75421851499709e-08 | 4.4214232189473e-07 | 1.57639877076219e-07 | 7313 | tags=59%, list=18%, signal=48% | POLR3H/POLR1C/POLR2K/POLR3C/POLR2H/POLR2D/POLR3GL/POLR3K/POLR1D/POLR2C/POLR2E/POLR2F/POLR2G/POLR2L/POLR2I/POLR2J/POLR1H |
| KEGG_ARGININE_AND_PROLINE_METABOLISM | KEGG_ARGININE_AND_PROLINE_METABOLISM | KEGG_ARGININE_AND_PROLINE_METABOLISM | 54 | -0.568713588 | -2.340391556 | 4.57219345829449e-08 | 4.4214232189473e-07 | 1.57639877076219e-07 | 9808 | tags=65%, list=23%, signal=50% | P4HA3/ARG2/GAMT/ASS1/MAOA/NOS1/ALDH18A1/NOS3/ODC1/PYCR2/AGMAT/ALDH7A1/OAT/ARG1/GOT2/PYCR1/SMS/GLUD1/PRODH/LAP3/CKB/ALDH1B1/SAT2/PYCR3/GOT1/CKMT1A/ALDH2/ASL/MAOB/CKMT1B/ALDH4A1/GLUD2/P4HA2/ACY1/SRM |
| KEGG_ENDOCYTOSIS | KEGG_ENDOCYTOSIS | KEGG_ENDOCYTOSIS | 181 | -0.388068377 | -2.029755877 | 8.14409041696159e-08 | 7.21333722645169e-07 | 2.57181802640892e-07 | 9249 | tags=44%, list=22%, signal=34% | AP2A2/KDR/PRKCZ/MET/AGAP2/SMAP2/RAB11FIP5/SRC/TFRC/RAB4A/FLT1/HGS/CCR5/SMAP1/HSPA1B/SH3GL3/CLTCL1/ERBB3/HLA-F/PLD1/ERBB4/GIT1/SH3GLB1/HSPA8/ARRB2/HLA-C/GRK7/AP2B1/GRK2/EGFR/CDC42/ARF6/ACAP1/HLA-E/ARFGAP2/AP2A1/FGFR4/VPS28/GRK1/CHMP5/GRK6/SH3GL2/VPS37D/EPN3/EHD2/LDLR/EHD4/CHMP4A/FGFR3/SH3GLB2/VPS37B/PSD/CHMP6/TSG101/PSD2/VPS4A/DNM2/HLA-B/HRAS/ASAP3/PARD6A/RAB11B/RAB5C/HSPA2/AP2S1/CLTB/CHMP2A/MVB12A/CLTA/HLA-A/EHD1/EPN1/VPS25/AP2M1/MVB12B/IL2RG/CHMP4B/SH3GL1/SNF8 |
| KEGG_GLYCOLYSIS_GLUCONEOGENESIS | KEGG_GLYCOLYSIS_GLUCONEOGENESIS | KEGG_GLYCOLYSIS_GLUCONEOGENESIS | 59 | -0.550237936 | -2.298726901 | 9.91243961035649e-08 | 8.01614681533177e-07 | 2.85804894943917e-07 | 6773 | tags=51%, list=16%, signal=43% | GALM/PGAM2/ALDH7A1/PGM1/PDHA1/ACSS2/HK3/ALDH1A3/ADH5/ALDH1B1/PFKL/PDHB/ALDH3B1/FBP1/ALDH2/PKM/PGK1/GPI/PFKP/PGAM4/ALDOA/ENO1/GAPDH/LDHA/PCK2/PGAM1/ALDOC/AKR1A1/TPI1/HK1 |
| KEGG_NEUROTROPHIN_SIGNALING_PATHWAY | KEGG_NEUROTROPHIN_SIGNALING_PATHWAY | KEGG_NEUROTROPHIN_SIGNALING_PATHWAY | 124 | -0.440254895 | -2.144652977 | 9.86538393051127e-08 | 8.01614681533177e-07 | 2.85804894943917e-07 | 9015 | tags=44%, list=22%, signal=35% | NRAS/MAGED1/SHC3/RIPK2/NFKBIA/YWHAZ/IRAK3/YWHAB/ABL1/TP73/BEX3/SHC1/SORT1/MAP2K5/RPS6KA1/SH2B2/RPS6KA4/SHC2/CALM2/AKT2/IRAK2/MAP2K1/CDC42/NFKBIE/MAPK14/MAPK11/PRKCD/YWHAQ/CAMK2A/RELA/NTRK2/CALM3/YWHAH/MAPK10/SHC4/NTRK3/ARHGDIB/NFKB1/RHOA/ATF4/RAC1/HRAS/CAMK2B/CSK/GRB2/MAPK3/NGFR/BAX/MAP2K2/NFKBIB/IRAK1/BAD/ARHGDIA/YWHAE/MAPKAPK2 |
| KEGG_FATTY_ACID_METABOLISM | KEGG_FATTY_ACID_METABOLISM | KEGG_FATTY_ACID_METABOLISM | 40 | -0.61160921 | -2.320103231 | 1.28357194239978e-07 | 9.94768255359833e-07 | 3.54671194610467e-07 | 8395 | tags=60%, list=20%, signal=48% | ACADL/ACSL5/ACSL1/ALDH7A1/ACSL3/ADH5/HADHB/ALDH1B1/ACAT1/CPT1B/ACOX3/ALDH2/ACADVL/GCDH/ACAA2/HADHA/CPT2/CPT1A/ACAT2/ECI1/ACAA1/ECHS1/ACADS/CPT1C |
| KEGG_AMINO_SUGAR_AND_NUCLEOTIDE_SUGAR_METABOLISM | KEGG_AMINO_SUGAR_AND_NUCLEOTIDE_SUGAR_METABOLISM | KEGG_AMINO_SUGAR_AND_NUCLEOTIDE_SUGAR_METABOLISM | 43 | -0.599521402 | -2.309770719 | 1.69023651256097e-07 | 1.23229895580859e-06 | 4.39359559795932e-07 | 7301 | tags=53%, list=17%, signal=44% | HEXB/AMDHD2/GFUS/CHIT1/GMPPB/NANP/PGM1/MPI/HK3/GALK2/GALK1/HEXA/GNPDA1/RENBP/CYB5R1/GPI/PMM1/CYB5R3/GMPPA/GMDS/NANS/GALE/HK1 |
| KEGG_REGULATION_OF_ACTIN_CYTOSKELETON | KEGG_REGULATION_OF_ACTIN_CYTOSKELETON | KEGG_REGULATION_OF_ACTIN_CYTOSKELETON | 206 | -0.360204652 | -1.906190516 | 1.73762932067718e-07 | 1.23229895580859e-06 | 4.39359559795932e-07 | 8168 | tags=37%, list=20%, signal=30% | FGF13/ACTN2/ITGA7/ENAH/MYLK2/PIP4K2C/ARPC5/FGF14/FGF17/FGD1/ITGA3/PAK5/GIT1/ITGA9/F2/ITGB4/PDGFA/EGFR/CHRM1/MAP2K1/CDC42/NCKAP1L/DOCK1/EZR/ITGAM/ARAF/FGFR4/DIAPH2/CD14/PAK4/RRAS/PFN2/ACTN4/WAS/APC2/ITGAE/FGFR3/ITGAD/GSN/FGF12/ARPC2/CHRM4/TMSB4X/DIAPH3/FN1/PPP1CA/ARPC5L/IQGAP3/CYFIP1/ITGB2/RHOA/TMSB4XP8/RAC1/HRAS/RAC3/CSK/FGF1/ARPC1A/MYH14/ACTB/PFN1/VAV1/BCAR1/ARPC4/GNG12/CFL1/MAPK3/ACTG1/ARPC1B/MAP2K2/ARPC3/RAC2/MYL12B/BRK1/MYL12A/LIMK1 |
| KEGG_PATHWAYS_IN_CANCER | KEGG_PATHWAYS_IN_CANCER | KEGG_PATHWAYS_IN_CANCER | 319 | -0.314026033 | -1.774538524 | 1.78882106488344e-07 | 1.23229895580859e-06 | 4.39359559795932e-07 | 8329 | tags=33%, list=20%, signal=27% | EPAS1/NFKBIA/FGF13/BMP2/HSP90AA1/IL6/COL4A6/BCR/TFG/TRAF3/FGF14/PTCH2/ABL1/WNT5A/PRKCG/CTNNA3/STAT1/CDKN2B/DVL2/MECOM/MLH1/CYCS/FGF17/FH/CTBP1/CDKN1A/ITGA3/WNT11/GLI2/PLD1/FLT3/STAT5A/DCC/RUNX1/MMP9/CEBPA/RALB/BRCA2/PDGFA/SMO/PAX8/BMP4/AKT2/EGFR/MAP2K1/CDC42/CCNA1/BID/HSP90AB1/ARAF/COL4A1/HDAC1/TRAF2/CCND1/MMP1/CASP9/CDK2/WNT7B/APC2/E2F2/RELA/PIAS4/SPI1/CTNNA2/ELOB/STAT3/RAD51/PML/COL4A2/LAMA4/FGFR3/CDKN2A/BIRC5/FGF12/FN1/MAPK10/LAMB3/CCNE1/RUNX1T1/TPM3/NFKB1/GSTP1/RHOA/RAC1/HRAS/RAC3/FGF1/FADD/GRB2/CTNNA1/MAPK3/CDK4/BAX/TGFB1/E2F1/MAP2K2/DAPK3/RAC2/FZD5/CKS1B/PPARG/RBX1/BAD/ELOC/ARNT2 |
| KEGG_CARDIAC_MUSCLE_CONTRACTION | KEGG_CARDIAC_MUSCLE_CONTRACTION | KEGG_CARDIAC_MUSCLE_CONTRACTION | 76 | -0.501339588 | -2.240202818 | 1.96278321800011e-07 | 1.30384885195722e-06 | 4.64869709526342e-07 | 4760 | tags=45%, list=11%, signal=40% | COX6B2/MT-CO1/UQCRC2/UQCRB/ATP1A2/MT-CO3/ATP1A1/CACNA2D1/COX7A1/UQCRC1/COX6C/TPM3/CACNG7/CACNG4/MT-CO2/COX7A2L/ATP1B2/COX7C/UQCRHL/COX4I1/UQCRQ/UQCRFS1/CYC1/COX6B1/COX8A/ATP1B3/COX7A2/COX5B/UQCR11/UQCRH/COX7B/UQCR10/COX5A/COX6A1 |
| KEGG_PPAR_SIGNALING_PATHWAY | KEGG_PPAR_SIGNALING_PATHWAY | KEGG_PPAR_SIGNALING_PATHWAY | 67 | -0.517224582 | -2.227800785 | 4.15569891997284e-07 | 2.66537930729292e-06 | 9.50305016182536e-07 | 8795 | tags=52%, list=21%, signal=41% | FADS2/SORBS1/ACADL/ACSL5/OLR1/APOA1/ACSL1/SLC27A4/PLTP/SLC27A2/FABP7/ACSL3/ANGPTL4/ACOX2/APOA2/SCD5/CYP27A1/FABP5/ILK/MMP1/NR1H3/CPT1B/ACOX3/SLC27A5/FABP3/LPL/CPT2/CPT1A/UBC/ACAA1/PCK2/PPARG/SCD/CPT1C/DBI |
| KEGG_CITRATE_CYCLE_TCA_CYCLE | KEGG_CITRATE_CYCLE_TCA_CYCLE | KEGG_CITRATE_CYCLE_TCA_CYCLE | 30 | -0.652325839 | -2.32481917 | 6.26977904496783e-07 | 3.7618674269807e-06 | 1.34124305546001e-06 | 6634 | tags=67%, list=16%, signal=56% | FH/CS/PDHA1/OGDH/OGDHL/PC/PDHB/IDH3B/ACLY/IDH1/IDH3G/SDHA/MDH1/SUCLG1/SDHC/ACO2/PCK2/IDH2/MDH2/SDHB |
| KEGG_GLYOXYLATE_AND_DICARBOXYLATE_METABOLISM | KEGG_GLYOXYLATE_AND_DICARBOXYLATE_METABOLISM | KEGG_GLYOXYLATE_AND_DICARBOXYLATE_METABOLISM | 15 | -0.774443551 | -2.304812753 | 6.15130035114691e-07 | 3.7618674269807e-06 | 1.34124305546001e-06 | 7093 | tags=80%, list=17%, signal=66% | PGP/HYI/CS/GLYCTK/AFMID/MTHFD1/MDH1/GRHPR/ACO2/MTHFD1L/MDH2/MTHFD2 |
| KEGG_VIBRIO_CHOLERAE_INFECTION | KEGG_VIBRIO_CHOLERAE_INFECTION | KEGG_VIBRIO_CHOLERAE_INFECTION | 54 | -0.534293112 | -2.198743118 | 1.03595908995988e-06 | 6.02151221039178e-06 | 2.14688890353527e-06 | 5152 | tags=41%, list=12%, signal=36% | ATP6V1G1/ATP6V0B/PDIA4/TCIRG1/KDELR2/ATP6V0C/ATP6V0D1/SEC61A1/ACTB/ACTG1/ATP6AP1/TJP1/ATP6V1H/ATP6V1F/ADCY3/SEC61G/KDELR1/ATP6V0E1/ATP6V1D/ARF1/ATP6V1E1/SEC61B |
| KEGG_VIRAL_MYOCARDITIS | KEGG_VIRAL_MYOCARDITIS | KEGG_VIRAL_MYOCARDITIS | 67 | -0.501282431 | -2.159134411 | 1.81960809479084e-06 | 1.0255972897912e-05 | 3.65662870723518e-06 | 7895 | tags=48%, list=19%, signal=39% | SGCB/MYH15/ABL1/CD55/CYCS/HLA-F/ICAM1/CD40/HLA-C/HLA-DPA1/HLA-DRB1/HLA-DRB5/BID/HLA-DPB1/HLA-E/HLA-DRA/HLA-DMA/CCND1/CASP9/CAV1/HLA-DQA2/CXADR/ITGB2/HLA-B/RAC1/RAC3/MYH14/ACTB/ACTG1/HLA-A/EIF4G1/RAC2 |
| KEGG_ANTIGEN_PROCESSING_AND_PRESENTATION | KEGG_ANTIGEN_PROCESSING_AND_PRESENTATION | KEGG_ANTIGEN_PROCESSING_AND_PRESENTATION | 74 | -0.476733645 | -2.111402611 | 2.04783893148916e-06 | 1.12028835663819e-05 | 3.99423692519558e-06 | 9783 | tags=51%, list=23%, signal=39% | LGMN/HLA-DMB/CD8A/PSME3/HSP90AA1/HSPA1B/RFX5/HLA-F/HSPA4/HSPA8/HLA-C/HLA-DPA1/HLA-DRB1/B2M/HLA-DRB5/HLA-DPB1/HLA-E/HSP90AB1/HLA-DRA/HLA-DMA/NFYC/TAP1/CD74/HLA-DQA2/CTSL/KIR2DL4/HSPA5/HLA-B/HSPA2/PSME2/IFI30/HLA-A/CTSB/RFXANK/TAPBP/PDIA3/PSME1/CALR |
| KEGG_PENTOSE_PHOSPHATE_PATHWAY | KEGG_PENTOSE_PHOSPHATE_PATHWAY | KEGG_PENTOSE_PHOSPHATE_PATHWAY | 26 | -0.656135428 | -2.289967847 | 3.09235612322694e-06 | 1.64336639691489e-05 | 5.85920107558789e-06 | 7431 | tags=69%, list=18%, signal=57% | RBKS/TKTL2/PGM1/PRPS1/PRPS2/TKT/PGD/PFKL/TALDO1/FBP1/RPIA/G6PD/PGLS/GPI/PFKP/DERA/ALDOA/ALDOC |
| KEGG_FRUCTOSE_AND_MANNOSE_METABOLISM | KEGG_FRUCTOSE_AND_MANNOSE_METABOLISM | KEGG_FRUCTOSE_AND_MANNOSE_METABOLISM | 34 | -0.61008359 | -2.225962639 | 3.55560407073792e-06 | 1.83706210321459e-05 | 6.54979697241195e-06 | 6322 | tags=53%, list=15%, signal=45% | GFUS/GMPPB/MPI/AKR1B1/HK3/KHK/PFKL/FBP1/PFKFB3/PMM1/PFKP/ALDOA/GMPPA/PHPT1/ALDOC/TPI1/GMDS/HK1 |
| KEGG_BIOSYNTHESIS_OF_UNSATURATED_FATTY_ACIDS | KEGG_BIOSYNTHESIS_OF_UNSATURATED_FATTY_ACIDS | KEGG_BIOSYNTHESIS_OF_UNSATURATED_FATTY_ACIDS | 22 | -0.68190101 | -2.237384086 | 4.07511707185084e-06 | 1.98375918024269e-05 | 7.07282560018616e-06 | 8948 | tags=68%, list=21%, signal=54% | ACOT4/FADS2/FADS1/PECR/ELOVL2/SCD5/HSD17B12/ACOX3/ACOT2/HADHA/TECR/ACOT7/ACOT1/ACAA1/SCD |
| KEGG_NUCLEOTIDE_EXCISION_REPAIR | KEGG_NUCLEOTIDE_EXCISION_REPAIR | KEGG_NUCLEOTIDE_EXCISION_REPAIR | 44 | -0.55394224 | -2.151876026 | 4.15949505534757e-06 | 1.98375918024269e-05 | 7.07282560018616e-06 | 10241 | tags=61%, list=25%, signal=46% | POLE/RFC5/RPA4/ERCC1/LIG1/RPA2/RPA1/RAD23A/RFC4/POLE3/DDB2/MNAT1/POLE2/ERCC2/PCNA/RAD23B/RPA3/DDB1/POLD2/POLE4/POLD4/GTF2H4/RFC2/CDK7/POLD1/RBX1/CETN2 |
| KEGG_P53_SIGNALING_PATHWAY | KEGG_P53_SIGNALING_PATHWAY | KEGG_P53_SIGNALING_PATHWAY | 68 | -0.492932474 | -2.140880753 | 4.05705115372128e-06 | 1.98375918024269e-05 | 7.07282560018616e-06 | 8638 | tags=50%, list=21%, signal=40% | GADD45G/PIDD1/THBS1/TP73/CYCS/CDKN1A/CHEK1/CDK1/SERPINE1/BBC3/BID/GADD45B/DDB2/STEAP3/TP53I3/CCND1/SESN2/RRM2/GTSE1/CASP9/CDK2/CCNB2/GADD45A/CCNB1/ADGRB1/EI24/CDKN2A/CCND3/SHISA5/CHEK2/CCNE1/CD82/CDK4/BAX |
| KEGG_GALACTOSE_METABOLISM | KEGG_GALACTOSE_METABOLISM | KEGG_GALACTOSE_METABOLISM | 26 | -0.65125023 | -2.272918094 | 4.85921689875938e-06 | 2.20442522724206e-05 | 7.85958060646575e-06 | 5811 | tags=54%, list=14%, signal=46% | PGM1/AKR1B1/HK3/GALK2/B4GALT1/PFKL/GALK1/GAA/PFKP/GLB1/GALE/B4GALT2/GLA/HK1 |
| KEGG_AMINOACYL_TRNA_BIOSYNTHESIS | KEGG_AMINOACYL_TRNA_BIOSYNTHESIS | KEGG_AMINOACYL_TRNA_BIOSYNTHESIS | 41 | -0.571235572 | -2.194611389 | 4.78179988583701e-06 | 2.20442522724206e-05 | 7.85958060646575e-06 | 8141 | tags=51%, list=19%, signal=41% | VARS2/TARS2/WARS1/DARS1/TARS1/CARS2/DARS2/SARS2/LARS2/YARS1/QARS1/AARS1/VARS1/GARS1/SARS1/WARS2/MTFMT/FARS2/FARSA/CARS1/KARS1 |
| KEGG_PROTEIN_EXPORT | KEGG_PROTEIN_EXPORT | KEGG_PROTEIN_EXPORT | 24 | -0.65343801 | -2.220814838 | 1.14086509473701e-05 | 4.93490482839729e-05 | 1.75947370791754e-05 | 7799 | tags=62%, list=19%, signal=51% | SEC11C/SRP19/SRPRA/SRPRB/SPCS2/SEC11A/SRP68/SRP9P1/SEC61A1/HSPA5/OXA1L/SPCS1/SEC61G/SRP14/SEC61B |
| KEGG_TIGHT_JUNCTION | KEGG_TIGHT_JUNCTION | KEGG_TIGHT_JUNCTION | 128 | -0.382122291 | -1.864856932 | 1.13493803207118e-05 | 4.93490482839729e-05 | 1.75947370791754e-05 | 12383 | tags=48%, list=30%, signal=34% | OCLN/MAP3K20/EPB41L3/CLDN6/PRKCB/RRAS2/ACTN3/MYL5/CLDN16/PRKCE/MRAS/PPP2R2D/CTNNB1/PRKCZ/PPP2R1B/NRAS/LLGL1/CRB3/SYMPK/SRC/CLDN10/ACTN2/MYH15/CLDN1/PRKCG/MAGI1/CTNNA3/PPP2CA/AKT2/CDC42/CLDN11/MAGI2/PRKCD/VAPA/JAM2/CSNK2A2/CLDN23/RRAS/ACTN4/HCLS1/PPP2R2C/CTNNA2/EXOC4/RAB3B/GNAI2/RHOA/HRAS/MYH14/PARD6A/ACTB/CTNNA1/ACTG1/CDK4/CSNK2B/MPDZ/RAB13/TJP1/MYL12B/PPP2R1A/EPB41L1/MYL12A |
| KEGG_CELL_ADHESION_MOLECULES_CAMS | KEGG_CELL_ADHESION_MOLECULES_CAMS | KEGG_CELL_ADHESION_MOLECULES_CAMS | 130 | -0.368643812 | -1.805473862 | 1.53893841890794e-05 | 6.50551240720173e-05 | 2.3194526409378e-05 | 12105 | tags=48%, list=29%, signal=34% | CD80/CLDN6/CD8B/CD274/ITGA8/SELE/CTLA4/HLA-G/CLDN16/SDC1/SIGLEC1/PDCD1LG2/HLA-DMB/CD8A/ICAM3/CLDN10/PDCD1/CLDN1/CD58/CDH2/NLGN1/ICAM2/CADM1/HLA-F/ITGA9/ICAM1/CD40/HLA-C/NRXN2/CD99/CNTN1/MAG/HLA-DPA1/NRXN3/HLA-DRB1/SELPLG/CLDN11/HLA-DRB5/ITGAM/HLA-DPB1/HLA-E/SELL/HLA-DRA/HLA-DMA/CNTN2/SDC3/NCAM2/JAM2/CLDN23/NLGN4X/SDC4/CADM3/NFASC/HLA-DQA2/CD276/MPZ/ITGB2/VCAM1/HLA-B/HLA-A/CDH5/NECTIN2 |
| KEGG_PROGESTERONE_MEDIATED_OOCYTE_MATURATION | KEGG_PROGESTERONE_MEDIATED_OOCYTE_MATURATION | KEGG_PROGESTERONE_MEDIATED_OOCYTE_MATURATION | 84 | -0.43686481 | -2.002900254 | 1.63220068239639e-05 | 6.74642948723843e-05 | 2.40534837405784e-05 | 8048 | tags=42%, list=19%, signal=34% | ADCY1/HSP90AA1/CPEB1/PRKACA/ADCY5/RPS6KA1/CCNA2/FZR1/CDK1/BUB1/AKT2/ANAPC10/MAP2K1/CCNA1/ADCY2/HSP90AB1/MAPK14/ARAF/MAPK11/ADCY8/CDC25A/MAD2L1/CDK2/CCNB2/CCNB1/CDC25C/MAPK10/CDC26/PKMYT1/PLK1/GNAI2/MAPK3/ADCY3/ANAPC11/MAD2L2 |
| KEGG_ACUTE_MYELOID_LEUKEMIA | KEGG_ACUTE_MYELOID_LEUKEMIA | KEGG_ACUTE_MYELOID_LEUKEMIA | 57 | -0.479055802 | -1.992938909 | 2.22313053586903e-05 | 8.79792084407743e-05 | 3.13677992742998e-05 | 6301 | tags=42%, list=15%, signal=36% | FLT3/STAT5A/RUNX1/CEBPA/PIM2/AKT2/MAP2K1/CCNA1/ARAF/CCND1/RELA/SPI1/RPS6KB2/EIF4EBP1/STAT3/PML/RUNX1T1/NFKB1/HRAS/GRB2/PIM1/MAPK3/MAP2K2/BAD |
| KEGG_CHEMOKINE_SIGNALING_PATHWAY | KEGG_CHEMOKINE_SIGNALING_PATHWAY | KEGG_CHEMOKINE_SIGNALING_PATHWAY | 185 | -0.331175545 | -1.730210443 | 2.21977439424554e-05 | 8.79792084407743e-05 | 3.13677992742998e-05 | 10448 | tags=43%, list=25%, signal=33% | CXCL3/XCL1/CXCL10/GNG10/CXCR3/CCL3/GNB3/GNG3/CCL22/IKBKG/PRKCZ/NRAS/CXCL12/CCL4/ELMO1/SHC3/CCL18/NCF1/NFKBIA/ADCY1/CCL2/GNG8/CCR5/CXCL13/CXCR6/STAT1/JAK3/CCL5/CCL24/SHC1/PRKACA/ADCY5/CCL19/HCK/GNG13/ARRB2/SHC2/GRK7/CXCL16/GRK2/AKT2/MAP2K1/CDC42/ADCY2/CXCL11/GSK3A/CCL25/ADCY8/PRKCD/GRK1/GRK6/FGR/WAS/GNGT2/PLCB3/RELA/CXCL9/STAT3/GNB1/GNB2/SHC4/CXCL14/GNAI2/GNG4/NFKB1/RHOA/RAC1/HRAS/PTK2B/CSK/GNG5/GRB2/VAV1/BCAR1/GNG12/MAPK3/NFKBIB/RAC2/ADCY3/CX3CL1 |
| KEGG_AMYOTROPHIC_LATERAL_SCLEROSIS_ALS | KEGG_AMYOTROPHIC_LATERAL_SCLEROSIS_ALS | KEGG_AMYOTROPHIC_LATERAL_SCLEROSIS_ALS | 52 | -0.503070249 | -2.032148753 | 2.41332168185524e-05 | 9.35162151718904e-05 | 3.33419442887894e-05 | 4763 | tags=52%, list=11%, signal=46% | GRIN2A/NEFM/NOS1/GRIN1/MAP2K6/CYCS/TNFRSF1B/BID/MAPK14/MAPK11/CHP1/DAXX/SOD1/GRIA1/SLC1A2/CASP9/GRIN2B/CCS/GPX1/DERL1/GRIA2/RAC1/TOMM40/BAX/MAP2K3/TOMM40L/BAD |
| KEGG_LEUKOCYTE_TRANSENDOTHELIAL_MIGRATION | KEGG_LEUKOCYTE_TRANSENDOTHELIAL_MIGRATION | KEGG_LEUKOCYTE_TRANSENDOTHELIAL_MIGRATION | 112 | -0.392350211 | -1.891133754 | 2.58901179512806e-05 | 9.82767742640448e-05 | 3.5039257377673e-05 | 8860 | tags=39%, list=21%, signal=31% | CXCL12/MSN/NCF1/CYBB/CLDN10/ACTN2/CLDN1/PRKCG/CTNNA3/RAPGEF4/NCF4/MMP9/RHOH/ICAM1/CD99/CDC42/EZR/CLDN11/ITGAM/MAPK14/MAPK11/VASP/JAM2/CLDN23/ACTN4/CTNNA2/SIPA1/ITGB2/GNAI2/VCAM1/RHOA/RAC1/PTK2B/ACTB/CYBA/VAV1/BCAR1/THY1/CTNNA1/ACTG1/CDH5/RAC2/MYL12B/MYL12A |
| KEGG_N_GLYCAN_BIOSYNTHESIS | KEGG_N_GLYCAN_BIOSYNTHESIS | KEGG_N_GLYCAN_BIOSYNTHESIS | 46 | -0.523417187 | -2.063112621 | 3.23398671530303e-05 | 0.000120304305809273 | 4.28928764345455e-05 | 10552 | tags=57%, list=25%, signal=42% | MGAT5B/ALG14/MGAT2/MGAT4B/MOGS/ALG5/B4GALT3/STT3A/GANAB/ALG2/ALG12/ALG1/RFT1/DOLPP1/DPAGT1/B4GALT1/RPN2/ALG8/DPM3/DDOST/ALG3/MGAT1/RPN1/TUSC3/DAD1/B4GALT2 |
| KEGG_BASE_EXCISION_REPAIR | KEGG_BASE_EXCISION_REPAIR | KEGG_BASE_EXCISION_REPAIR | 34 | -0.572517456 | -2.088898123 | 3.55826890509369e-05 | 0.000129772160068123 | 4.62685120786176e-05 | 10459 | tags=62%, list=25%, signal=46% | MUTYH/POLE/PARP1/LIG1/SMUG1/NEIL3/APEX1/POLE3/PARP3/POLE2/PCNA/NTHL1/APEX2/POLD2/UNG/FEN1/XRCC1/POLE4/POLD4/POLD1/MPG |
| KEGG_VALINE_LEUCINE_AND_ISOLEUCINE_DEGRADATION | KEGG_VALINE_LEUCINE_AND_ISOLEUCINE_DEGRADATION | KEGG_VALINE_LEUCINE_AND_ISOLEUCINE_DEGRADATION | 44 | -0.519456901 | -2.017912286 | 3.84523983688911e-05 | 0.000137541271088726 | 4.90384837498003e-05 | 11421 | tags=59%, list=27%, signal=43% | HADH/EHHADH/ALDH9A1/MCCC1/HIBADH/HMGCS1/AOX1/BCAT1/ALDH7A1/OXCT2/IL4I1/BCAT2/HADHB/ALDH1B1/ACAT1/ALDH2/ACAA2/HADHA/PCCB/HMGCL/BCKDHA/ACAT2/ACAA1/ECHS1/HSD17B10/ACADS |
| KEGG_INSULIN_SIGNALING_PATHWAY | KEGG_INSULIN_SIGNALING_PATHWAY | KEGG_INSULIN_SIGNALING_PATHWAY | 136 | -0.359315304 | -1.77399837 | 4.72121395482418e-05 | 0.000165687885961754 | 5.90737793751585e-05 | 10247 | tags=41%, list=25%, signal=31% | EIF4E/PRKAR1B/PKLR/INPP5D/INSR/PYGL/PRKCZ/NRAS/PHKG2/SHC3/SORBS1/LIPE/RPS6/SHC1/PRKACA/ACACA/MKNK1/SH2B2/PHKA1/SHC2/CALM2/FLOT2/PPP1R3C/AKT2/HK3/MAP2K1/ARAF/EXOC7/PRKAA2/FBP1/EIF4E2/PRKAG1/INPP5K/RPS6KB2/EIF4EBP1/SOCS1/GYS1/FASN/CALM3/SOCS3/MAPK10/SHC4/PPP1CA/PTPN1/FLOT1/HRAS/GRB2/MAPK3/MAP2K2/TRIP10/PCK2/RHEB/ELK1/BAD/PRKAB1/HK1 |
| KEGG_FOCAL_ADHESION | KEGG_FOCAL_ADHESION | KEGG_FOCAL_ADHESION | 197 | -0.318754072 | -1.670385736 | 5.30692846801665e-05 | 0.00018279420278724 | 6.51728057475729e-05 | 12070 | tags=40%, list=29%, signal=28% | PDGFRA/LAMC1/COL5A2/ITGA8/PRKCB/FLNA/ACTN3/PARVB/MYL5/ITGA1/THBS3/RELN/LAMA1/CTNNB1/PARVG/JUN/KDR/MET/SHC3/SRC/ACTN2/THBS1/ITGA7/FLT1/COL4A6/VTN/MYLK2/PRKCG/THBS2/SHC1/ITGA3/PAK5/ITGA9/ITGB4/SHC2/PDGFA/SPP1/AKT2/EGFR/MAP2K1/CDC42/DOCK1/COL4A1/FLNC/TNR/VASP/CCND1/ILK/PAK4/ACTN4/CAV1/COL4A2/LAMA4/ZYX/COL11A1/TNC/CCND3/FN1/MAPK10/SHC4/PPP1CA/TLN2/LAMB3/RHOA/RAC1/HRAS/RAC3/ACTB/GRB2/VAV1/BCAR1/MAPK3/ACTG1/RAC2/MYL12B/ELK1/BAD/MYL12A |
| KEGG_FC_GAMMA_R_MEDIATED_PHAGOCYTOSIS | KEGG_FC_GAMMA_R_MEDIATED_PHAGOCYTOSIS | KEGG_FC_GAMMA_R_MEDIATED_PHAGOCYTOSIS | 96 | -0.38671846 | -1.813267974 | 6.61212054577234e-05 | 0.000223609894820665 | 7.97250898341928e-05 | 6383 | tags=34%, list=15%, signal=29% | PLD1/HCK/SYK/PLPP2/FCGR1A/PLPP3/AMPH/AKT2/MAP2K1/CDC42/ARF6/VASP/PRKCD/SPHK1/WAS/SPHK2/RPS6KB2/GSN/ARPC2/ARPC5L/MYO10/DNM2/RAC1/ASAP3/ARPC1A/VAV1/ARPC4/CFL1/MAPK3/ARPC1B/ARPC3/RAC2/LIMK1 |
| KEGG_STEROID_BIOSYNTHESIS | KEGG_STEROID_BIOSYNTHESIS | KEGG_STEROID_BIOSYNTHESIS | 17 | -0.682278708 | -2.109742587 | 7.73524954581703e-05 | 0.000256920788486066 | 9.16016393583595e-05 | 4975 | tags=53%, list=12%, signal=47% | CYP51A1/DHCR7/TM7SF2/DHCR24/EBP/SQLE/MSMO1/FDFT1/NSDHL |
| KEGG_MISMATCH_REPAIR | KEGG_MISMATCH_REPAIR | KEGG_MISMATCH_REPAIR | 23 | -0.622383444 | -2.071826094 | 7.98252550656202e-05 | 0.000260482411266761 | 9.28714878879792e-05 | 10081 | tags=65%, list=24%, signal=49% | RFC5/RPA4/LIG1/RPA2/RPA1/MLH1/EXO1/SSBP1/RFC4/PCNA/RPA3/POLD2/POLD4/RFC2/POLD1 |
| KEGG_TYPE_I_DIABETES_MELLITUS | KEGG_TYPE_I_DIABETES_MELLITUS | KEGG_TYPE_I_DIABETES_MELLITUS | 40 | -0.518735302 | -1.967791576 | 8.36614853472603e-05 | 0.000268293728872248 | 9.56565077473212e-05 | 12105 | tags=65%, list=29%, signal=46% | CD80/HLA-G/HSPD1/PRF1/IL12A/HLA-DMB/IFNG/PTPRN/HLA-F/GAD2/HLA-C/HLA-DPA1/GAD1/HLA-DRB1/ICA1/HLA-DRB5/HLA-DPB1/HLA-E/GZMB/HLA-DRA/HLA-DMA/PTPRN2/HLA-DQA2/CPE/HLA-B/HLA-A |
| KEGG_AXON_GUIDANCE | KEGG_AXON_GUIDANCE | KEGG_AXON_GUIDANCE | 128 | -0.35622406 | -1.738466773 | 0.000104671205991623 | 0.000329980412109186 | 0.000117650062042324 | 9040 | tags=40%, list=22%, signal=31% | MET/NRAS/RGS3/EPHA5/CXCL12/PLXNB3/EPHA8/EFNA4/RHOD/EFNA2/ABL1/UNC5D/SEMA6B/EFNB3/PAK5/ROBO2/SLIT3/DCC/EFNA3/SLIT2/EPHB2/EPHA7/SEMA3F/SEMA5B/CDC42/SRGAP2/SEMA3B/CHP1/UNC5B/PAK4/SEMA6D/LRRC4C/SEMA4A/DPYSL5/CDK5/FES/UNC5C/SRGAP1/NGEF/UNC5A/GNAI2/RHOA/RAC1/HRAS/RAC3/EFNA1/CFL1/MAPK3/SLIT1/RAC2/LIMK1 |
| KEGG_HOMOLOGOUS_RECOMBINATION | KEGG_HOMOLOGOUS_RECOMBINATION | KEGG_HOMOLOGOUS_RECOMBINATION | 28 | -0.568278756 | -1.988273216 | 0.000113240080703296 | 0.000351044250180217 | 0.00012516008919838 | 10595 | tags=64%, list=25%, signal=48% | XRCC2/BLM/RPA4/RAD51C/MUS81/EME1/RPA2/RPA1/SEM1/SSBP1/BRCA2/RAD54L/RAD51/RPA3/POLD2/RAD51B/POLD4/POLD1 |
| KEGG_CYTOSOLIC_DNA_SENSING_PATHWAY | KEGG_CYTOSOLIC_DNA_SENSING_PATHWAY | KEGG_CYTOSOLIC_DNA_SENSING_PATHWAY | 47 | -0.495302156 | -1.951483531 | 0.000127062168295587 | 0.000387435463983267 | 0.000138134885291148 | 9378 | tags=53%, list=22%, signal=41% | IKBKG/ZBP1/CCL4/IRF3/NFKBIA/IL6/POLR3H/IRF7/CCL5/POLR1C/POLR3C/TREX1/IL33/RIPK3/IL18/POLR3GL/AIM2/POLR3K/RELA/STING1/IKBKE/POLR1D/PYCARD/NFKB1/NFKBIB |
| KEGG_GLYCEROLIPID_METABOLISM | KEGG_GLYCEROLIPID_METABOLISM | KEGG_GLYCEROLIPID_METABOLISM | 45 | -0.495340759 | -1.936960573 | 0.000131676693099522 | 0.000395030079298566 | 0.000140842642873852 | 10941 | tags=51%, list=26%, signal=38% | ALDH9A1/DGKI/DGAT1/PLPP1/DGKB/LIPC/ALDH7A1/LIPG/GPAT2/PLPP2/AGPAT1/PLPP3/GLYCTK/AKR1B1/AGPAT3/ALDH1B1/ALDH2/LPL/GPAT4/AGPAT2/AKR1A1/TKFC/GLA |
| KEGG_EPITHELIAL_CELL_SIGNALING_IN_HELICOBACTER_PYLORI_INFECTION | KEGG_EPITHELIAL_CELL_SIGNALING_IN_HELICOBACTER_PYLORI_INFECTION | KEGG_EPITHELIAL_CELL_SIGNALING_IN_HELICOBACTER_PYLORI_INFECTION | 68 | -0.438831654 | -1.90591266 | 0.00015018196322028 | 0.000443394367602732 | 0.000158086277073979 | 9378 | tags=46%, list=22%, signal=35% | IKBKG/JUN/MET/ATP6V1B1/SRC/NFKBIA/CCL5/GIT1/ATP6V1G1/ATP6V0B/EGFR/CDC42/TCIRG1/MAPK14/MAPK11/JAM2/ATP6V0C/RELA/ATP6V0D1/PTPRZ1/MAPK10/NFKB1/RAC1/CSK/ATP6AP1/TJP1/ATP6V1H/ATP6V1F/ATP6V0E1/ATP6V1D/ATP6V1E1 |
| KEGG_PANCREATIC_CANCER | KEGG_PANCREATIC_CANCER | KEGG_PANCREATIC_CANCER | 70 | -0.424645993 | -1.857177233 | 0.000171504778249551 | 0.000498435761787757 | 0.000177710543252002 | 6383 | tags=36%, list=15%, signal=30% | PLD1/RALB/BRCA2/AKT2/EGFR/MAP2K1/CDC42/ARAF/CCND1/CASP9/E2F2/RELA/STAT3/RAD51/CDKN2A/MAPK10/NFKB1/RAC1/RAC3/MAPK3/CDK4/TGFB1/E2F1/RAC2/BAD |
| KEGG_GLYCOSAMINOGLYCAN_BIOSYNTHESIS_KERATAN_SULFATE | KEGG_GLYCOSAMINOGLYCAN_BIOSYNTHESIS_KERATAN_SULFATE | KEGG_GLYCOSAMINOGLYCAN_BIOSYNTHESIS_KERATAN_SULFATE | 15 | -0.664894853 | -1.978786104 | 0.000221888162898452 | 0.000634941512294033 | 0.00022637982611502 | 8023 | tags=67%, list=19%, signal=54% | B4GALT3/B3GNT2/B3GNT7/B4GALT4/B4GALT1/ST3GAL2/B4GAT1/CHST6/CHST1/B4GALT2 |
| KEGG_ONE_CARBON_POOL_BY_FOLATE | KEGG_ONE_CARBON_POOL_BY_FOLATE | KEGG_ONE_CARBON_POOL_BY_FOLATE | 17 | -0.660293277 | -2.041759225 | 0.000230279889764905 | 0.000648970598428368 | 0.000231381707419282 | 10584 | tags=82%, list=25%, signal=62% | MTHFR/AMT/MTHFS/SHMT1/GART/ALDH1L1/MTHFD1/ATIC/MTFMT/DHFR/TYMS/SHMT2/MTHFD1L/MTHFD2 |
| KEGG_SNARE_INTERACTIONS_IN_VESICULAR_TRANSPORT | KEGG_SNARE_INTERACTIONS_IN_VESICULAR_TRANSPORT | KEGG_SNARE_INTERACTIONS_IN_VESICULAR_TRANSPORT | 38 | -0.500977832 | -1.878788389 | 0.000374663019122655 | 0.00104010927696737 | 0.000370836923876312 | 7650 | tags=50%, list=18%, signal=41% | VTI1A/STX4/STX1A/GOSR2/VAMP3/STX5/STX11/VAMP5/SNAP25/STX18/BNIP1/VTI1B/SNAP47/STX10/SNAP29/USE1/STX8/VAMP8/YKT6 |
| KEGG_GAP_JUNCTION | KEGG_GAP_JUNCTION | KEGG_GAP_JUNCTION | 90 | -0.386128911 | -1.773036981 | 0.000459863355832763 | 0.00125786153213079 | 0.000448473551353933 | 9015 | tags=40%, list=22%, signal=31% | NRAS/DRD1/SRC/ADCY1/DRD2/GJD2/PRKCG/PRKACA/MAP2K5/ADCY5/TUBA1A/CDK1/PDGFA/EGFR/MAP2K1/ADCY2/GUCY1A2/ADCY8/PLCB3/TUBB6/TUBB2A/TUBA4A/GNAI2/HRAS/GRB2/MAPK3/TUBB4A/MAP2K2/TJP1/TUBB/ADCY3/TUBA1B/TUBA1C/TUBB4B/TUBB3/TUBB2B |
| KEGG_MAPK_SIGNALING_PATHWAY | KEGG_MAPK_SIGNALING_PATHWAY | KEGG_MAPK_SIGNALING_PATHWAY | 257 | -0.276345978 | -1.530891405 | 0.000536000714671539 | 0.00144487149172328 | 0.000515149428288436 | 9378 | tags=37%, list=22%, signal=29% | IKBKG/JUN/LAMTOR3/CACNB3/NRAS/TAB1/GADD45G/DUSP14/STK3/MAP2K6/MAPK8IP2/DUSP2/FGF13/HSPA1B/CACNA1B/DUSP4/FGF14/PTPRR/DUSP5/PRKCG/DUSP10/MECOM/DUSP8/PRKACA/MAP2K5/FGF17/RPS6KA1/CACNA1E/MKNK1/RPS6KA4/HSPA8/ARRB2/CACNG3/STMN1/PDGFA/MAP3K13/AKT2/PPP5C/EGFR/MAP2K1/CDC42/MAP3K11/CACNA1G/JUND/PTPN7/IL1R2/GADD45B/MAPK14/MAP3K8/MAPK11/MAPT/FLNC/TRAF2/FGFR4/CHP1/DAXX/MAP3K6/PTPN5/MAPKAPK3/CD14/RRAS/ECSIT/RELA/GADD45A/FGFR3/NTRK2/CACNA2D1/FGF12/MAPK10/SRF/RELB/NFKB1/CACNA1A/CACNG7/MAPK8IP1/ATF4/RAC1/HRAS/CACNG4/RAC3/FGF1/GRB2/GNG12/HSPA2/HSPB1/MAPK3/DUSP3/TGFB1/MAP2K2/MAP2K3/PLA2G2C/RAC2/ELK1/MAPKAPK2 |
| KEGG_LEISHMANIA_INFECTION | KEGG_LEISHMANIA_INFECTION | KEGG_LEISHMANIA_INFECTION | 70 | -0.403313198 | -1.763878857 | 0.000656025714423546 | 0.00174315404118256 | 0.000621498045243359 | 9454 | tags=49%, list=23%, signal=38% | IL12A/JUN/HLA-DMB/TAB1/NCF1/IFNG/NFKBIA/STAT1/NCF4/FCGR1A/HLA-DPA1/HLA-DRB1/HLA-DRB5/ITGAM/HLA-DPB1/MAPK14/MAPK11/HLA-DRA/HLA-DMA/C3/RELA/PTPN6/HLA-DQA2/ITGB2/NFKB1/CYBA/MYD88/IL10/MAPK3/TGFB1/NFKBIB/IRAK1/ELK1/IFNGR2 |
| KEGG_ADIPOCYTOKINE_SIGNALING_PATHWAY | KEGG_ADIPOCYTOKINE_SIGNALING_PATHWAY | KEGG_ADIPOCYTOKINE_SIGNALING_PATHWAY | 67 | -0.413549351 | -1.781248611 | 0.000709988296827523 | 0.00185996934098478 | 0.000663146963678784 | 9387 | tags=42%, list=22%, signal=32% | AGRP/IKBKG/NFKBIA/ACSL5/STK11/ADIPOR2/ACSL1/NPY/ACSL3/AKT2/NFKBIE/TNFRSF1B/TRAF2/TRADD/PRKAA2/CPT1B/RELA/PRKAG1/POMC/STAT3/SOCS3/MAPK10/NFKB1/CPT1A/NFKBIB/PCK2/CPT1C/PRKAB1 |
| KEGG_TERPENOID_BACKBONE_BIOSYNTHESIS | KEGG_TERPENOID_BACKBONE_BIOSYNTHESIS | KEGG_TERPENOID_BACKBONE_BIOSYNTHESIS | 15 | -0.632323323 | -1.88185034 | 0.000802483330323923 | 0.00206364870456531 | 0.000735766091610723 | 9390 | tags=67%, list=22%, signal=52% | IDI1/HMGCS1/PDSS2/PDSS1/MVK/ACAT1/ACAT2/FDPS/PMVK/MVD |
| KEGG_TOLL_LIKE_RECEPTOR_SIGNALING_PATHWAY | KEGG_TOLL_LIKE_RECEPTOR_SIGNALING_PATHWAY | KEGG_TOLL_LIKE_RECEPTOR_SIGNALING_PATHWAY | 95 | -0.364723409 | -1.702231922 | 0.000809926642114344 | 0.00206364870456531 | 0.000735766091610723 | 10320 | tags=44%, list=25%, signal=33% | CXCL10/TLR8/CCL3/IL12A/IKBKG/JUN/TICAM2/TAB1/CCL4/IRF3/MAP2K6/NFKBIA/IL6/TRAF3/IRF7/STAT1/CCL5/TOLLIP/LY96/CD40/SPP1/AKT2/MAP2K1/CXCL11/MAPK14/MAP3K8/MAPK11/TICAM1/CD14/RELA/CXCL9/IRF5/IKBKE/MAPK10/NFKB1/RAC1/FADD/MYD88/MAPK3/MAP2K2/MAP2K3/IRAK1 |
| KEGG_TRYPTOPHAN_METABOLISM | KEGG_TRYPTOPHAN_METABOLISM | KEGG_TRYPTOPHAN_METABOLISM | 40 | -0.469602817 | -1.781410408 | 0.00103268371406544 | 0.00256105561088229 | 0.000913109810331548 | 12138 | tags=68%, list=29%, signal=48% | CYP1A2/HADH/EHHADH/ALDH9A1/ASMT/CYP1B1/AANAT/MAOA/AOX1/WARS1/ALDH7A1/HAAO/IL4I1/OGDH/KYNU/AFMID/OGDHL/ALDH1B1/INMT/ACAT1/ALDH2/WARS2/MAOB/GCDH/HADHA/ACAT2/ECHS1 |
| KEGG_NATURAL_KILLER_CELL_MEDIATED_CYTOTOXICITY | KEGG_NATURAL_KILLER_CELL_MEDIATED_CYTOTOXICITY | KEGG_NATURAL_KILLER_CELL_MEDIATED_CYTOTOXICITY | 123 | -0.338893686 | -1.646573324 | 0.00102742970780772 | 0.00256105561088229 | 0.000913109810331548 | 7331 | tags=29%, list=18%, signal=24% | ICAM2/PRKCG/SHC1/CD48/ULBP2/ULBP3/SYK/ICAM1/HLA-C/SHC2/MAP2K1/BID/HLA-E/GZMB/ARAF/CHP1/PTPN6/KIR2DL4/TYROBP/SHC4/ITGB2/HLA-B/RAC1/HRAS/PTK2B/RAC3/GRB2/VAV1/MAPK3/MICB/FCER1G/MAP2K2/HLA-A/RAC2/HCST/IFNGR2 |
| KEGG_PROXIMAL_TUBULE_BICARBONATE_RECLAMATION | KEGG_PROXIMAL_TUBULE_BICARBONATE_RECLAMATION | KEGG_PROXIMAL_TUBULE_BICARBONATE_RECLAMATION | 23 | -0.557934469 | -1.857284608 | 0.00142194133911594 | 0.00348001432994164 | 0.00124075213800975 | 8932 | tags=57%, list=21%, signal=44% | CA4/ATP1A3/AQP1/GLUD1/SLC38A3/ATP1A2/SLC25A10/ATP1A1/MDH1/GLUD2/ATP1B2/PCK2/ATP1B3 |
| KEGG_CHRONIC_MYELOID_LEUKEMIA | KEGG_CHRONIC_MYELOID_LEUKEMIA | KEGG_CHRONIC_MYELOID_LEUKEMIA | 73 | -0.392043052 | -1.729062324 | 0.00148158170749975 | 0.00357888568305134 | 0.00127600338444955 | 9378 | tags=42%, list=22%, signal=33% | IKBKG/NRAS/SHC3/NFKBIA/BCR/ABL1/SHC1/MECOM/CTBP1/CDKN1A/STAT5A/RUNX1/SHC2/AKT2/MAP2K1/ARAF/HDAC1/CCND1/E2F2/RELA/CDKN2A/SHC4/NFKB1/HRAS/GRB2/MAPK3/CDK4/TGFB1/E2F1/MAP2K2/BAD |
| KEGG_ALANINE_ASPARTATE_AND_GLUTAMATE_METABOLISM | KEGG_ALANINE_ASPARTATE_AND_GLUTAMATE_METABOLISM | KEGG_ALANINE_ASPARTATE_AND_GLUTAMATE_METABOLISM | 31 | -0.517457219 | -1.849953586 | 0.00153833027507965 | 0.00366832604057456 | 0.0013078921367074 | 10295 | tags=58%, list=25%, signal=44% | ADSS1/GFPT2/ASS1/NIT2/ACY3/GAD2/DDO/GOT2/IL4I1/GAD1/ASPA/GLUD1/GOT1/ASL/GPT2/ADSL/ALDH4A1/GLUD2 |
| KEGG_COMPLEMENT_AND_COAGULATION_CASCADES | KEGG_COMPLEMENT_AND_COAGULATION_CASCADES | KEGG_COMPLEMENT_AND_COAGULATION_CASCADES | 65 | -0.389911222 | -1.664088315 | 0.00166124121468317 | 0.00391127678393758 | 0.00139451294503717 | 12043 | tags=57%, list=29%, signal=41% | CFH/C5/CR1/CPB2/C7/F11/PLG/CFD/C4BPA/BDKRB2/SERPINA5/C8G/C1S/PLAUR/SERPING1/CFI/CD55/F2/CFB/SERPINE1/C1QC/C4BPB/C1QB/SERPINA1/C3/F12/PLAT/C3AR1/C1QA/MASP1/A2M/CD59/C2/F3/PLAU/F8/C1R |
| KEGG_BUTANOATE_METABOLISM | KEGG_BUTANOATE_METABOLISM | KEGG_BUTANOATE_METABOLISM | 33 | -0.497822768 | -1.81756434 | 0.00177016413070005 | 0.00411563160387762 | 0.00146737289781715 | 11421 | tags=61%, list=27%, signal=44% | HADH/EHHADH/ALDH9A1/ACSM4/HMGCS1/AACS/ALDH7A1/GAD2/OXCT2/PDHA1/GAD1/ALDH1B1/ACAT1/PDHB/ALDH2/HADHA/HMGCL/ACAT2/ECHS1/ACADS |
| KEGG_CALCIUM_SIGNALING_PATHWAY | KEGG_CALCIUM_SIGNALING_PATHWAY | KEGG_CALCIUM_SIGNALING_PATHWAY | 174 | -0.286048397 | -1.485324808 | 0.00193439870576157 | 0.00444195258360065 | 0.00158371823863521 | 12698 | tags=47%, list=30%, signal=33% | ATP2A2/DRD5/ATP2B3/HTR2C/PDE1B/PDGFRA/SLC8A3/PHKG1/ADRA1A/HRH1/PRKCB/PTAFR/CALM1/GRM5/ADORA2B/CCKBR/HTR5A/CACNA1C/TACR1/PDE1C/GRM1/HTR4/GRIN2A/BDKRB2/P2RX5/ATP2B2/ADRA1B/HRH2/DRD1/PHKG2/NOS1/GRIN1/CHRM3/VDAC3/CD38/ADCY1/PLCD3/ATP2A1/MYLK2/CACNA1B/NOS3/PRKCG/PRKACA/P2RX1/ERBB3/CACNA1E/ERBB4/PHKA1/PTGER1/PTGFR/CALM2/EGFR/CHRM1/CACNA1G/ADCY2/EDNRA/ADCY8/CHP1/NTSR1/PDE1A/SPHK1/PLCB3/CAMK2A/BST1/SPHK2/ITPKA/CALM3/ADORA2A/GNA15/PLCD1/CACNA1A/SLC25A6/CAMK2B/PTK2B/SLC25A5/P2RX4/ADCY3/VDAC2/VDAC1/SLC8A2/VDAC2P5/EDNRB |
| KEGG_PYRUVATE_METABOLISM | KEGG_PYRUVATE_METABOLISM | KEGG_PYRUVATE_METABOLISM | 39 | -0.483219571 | -1.823001005 | 0.00196771530565581 | 0.00446335422990221 | 0.00159134870675631 | 6684 | tags=64%, list=16%, signal=54% | ALDH9A1/ACYP1/PKLR/LDHD/ME2/ACACA/ALDH7A1/PDHA1/GLO1/HAGHL/AKR1B1/ACSS2/PC/ALDH1B1/ACYP2/ACAT1/PDHB/ALDH2/PKM/MDH1/GRHPR/ACAT2/LDHA/PCK2/MDH2 |
| KEGG_GRAFT_VERSUS_HOST_DISEASE | KEGG_GRAFT_VERSUS_HOST_DISEASE | KEGG_GRAFT_VERSUS_HOST_DISEASE | 37 | -0.458935379 | -1.713549587 | 0.00202171926047089 | 0.00453059978852514 | 0.00161532420303952 | 9785 | tags=46%, list=23%, signal=35% | PRF1/HLA-DMB/IFNG/IL6/HLA-F/HLA-C/HLA-DPA1/HLA-DRB1/HLA-DRB5/HLA-DPB1/HLA-E/GZMB/HLA-DRA/HLA-DMA/HLA-DQA2/HLA-B/HLA-A |
| KEGG_GLIOMA | KEGG_GLIOMA | KEGG_GLIOMA | 65 | -0.380310673 | -1.623114473 | 0.00231073853941153 | 0.00511663533726839 | 0.00182426726795647 | 7228 | tags=35%, list=17%, signal=29% | PRKCG/SHC1/CDKN1A/SHC2/PDGFA/CALM2/AKT2/EGFR/MAP2K1/ARAF/CCND1/CAMK2A/E2F2/CDKN2A/CALM3/SHC4/HRAS/CAMK2B/GRB2/MAPK3/CDK4/E2F1/MAP2K2 |
| KEGG_ECM_RECEPTOR_INTERACTION | KEGG_ECM_RECEPTOR_INTERACTION | KEGG_ECM_RECEPTOR_INTERACTION | 84 | -0.350639384 | -1.607581326 | 0.00309714233530855 | 0.00677727616902812 | 0.00241634634209831 | 12007 | tags=43%, list=29%, signal=31% | LAMC1/HSPG2/COL5A2/ITGA8/ITGA1/GP9/SDC1/THBS3/DAG1/SV2A/RELN/LAMA1/GP1BB/AGRN/THBS1/ITGA7/COL4A6/VTN/THBS2/ITGA3/SV2B/ITGA9/ITGB4/HMMR/SPP1/COL4A1/TNR/SDC3/SDC4/COL4A2/LAMA4/COL11A1/CD44/TNC/FN1/LAMB3 |
| KEGG_DRUG_METABOLISM_OTHER_ENZYMES | KEGG_DRUG_METABOLISM_OTHER_ENZYMES | KEGG_DRUG_METABOLISM_OTHER_ENZYMES | 44 | -0.426195334 | -1.655623013 | 0.00332825194228169 | 0.00719831234028364 | 0.0025664611060434 | 9835 | tags=48%, list=24%, signal=37% | GMPS/TK2/TPMT/UPP2/NAT1/IMPDH1/DPYS/UCKL1/CDA/CES2/IMPDH2/UCK2/CES1/TYMP/HPRT1/UPB1/TK1/UCK1/GUSB/ITPA/UPP1 |
| KEGG_GLYCOSPHINGOLIPID_BIOSYNTHESIS_GLOBO_SERIES | KEGG_GLYCOSPHINGOLIPID_BIOSYNTHESIS_GLOBO_SERIES | KEGG_GLYCOSPHINGOLIPID_BIOSYNTHESIS_GLOBO_SERIES | 14 | -0.619867381 | -1.800923293 | 0.00369680488995152 | 0.00781370124467025 | 0.00278586971371944 | 9800 | tags=71%, list=23%, signal=55% | FUT1/B3GALNT1/HEXB/B3GALT5/GBGT1/NAGA/ST3GAL2/FUT9/HEXA/GLA |
| KEGG_BLADDER_CANCER | KEGG_BLADDER_CANCER | KEGG_BLADDER_CANCER | 42 | -0.439586127 | -1.697536425 | 0.00366311759062548 | 0.00781370124467025 | 0.00278586971371944 | 6531 | tags=40%, list=16%, signal=34% | CDKN1A/MMP9/EGFR/MAP2K1/ARAF/CCND1/MMP1/E2F2/TYMP/FGFR3/CDKN2A/HRAS/MAPK3/CDK4/E2F1/MAP2K2/DAPK3 |
| KEGG_LYSINE_DEGRADATION | KEGG_LYSINE_DEGRADATION | KEGG_LYSINE_DEGRADATION | 44 | -0.422055528 | -1.639541286 | 0.00381315618337252 | 0.00796906797873359 | 0.00284126362569449 | 11421 | tags=55%, list=27%, signal=40% | HADH/EHHADH/ALDH9A1/DLST/DOT1L/NSD2/SETD1A/ALDH7A1/OGDH/TMLHE/OGDHL/ALDH1B1/ACAT1/PLOD1/KMT5A/BBOX1/ALDH2/GCDH/SUV39H1/HADHA/PLOD3/PIPOX/ACAT2/ECHS1 |
| KEGG_ADHERENS_JUNCTION | KEGG_ADHERENS_JUNCTION | KEGG_ADHERENS_JUNCTION | 73 | -0.3732717 | -1.64627336 | 0.00425033195408757 | 0.00878401937178098 | 0.00313182354511716 | 4964 | tags=27%, list=12%, signal=24% | EGFR/CDC42/CSNK2A2/ACTN4/WAS/NECTIN4/PTPN6/CTNNA2/PTPN1/RHOA/RAC1/RAC3/ACTB/CTNNA1/MAPK3/ACTG1/CSNK2B/TJP1/RAC2/NECTIN2 |
| KEGG_ALLOGRAFT_REJECTION | KEGG_ALLOGRAFT_REJECTION | KEGG_ALLOGRAFT_REJECTION | 35 | -0.454899585 | -1.662414343 | 0.00480579209904031 | 0.00982282780682965 | 0.00350219667136541 | 9785 | tags=54%, list=23%, signal=42% | PRF1/IL12A/HLA-DMB/IFNG/HLA-F/CD40/HLA-C/HLA-DPA1/HLA-DRB1/HLA-DRB5/HLA-DPB1/HLA-E/GZMB/HLA-DRA/HLA-DMA/HLA-DQA2/HLA-B/IL10/HLA-A |
| KEGG_PORPHYRIN_AND_CHLOROPHYLL_METABOLISM | KEGG_PORPHYRIN_AND_CHLOROPHYLL_METABOLISM | KEGG_PORPHYRIN_AND_CHLOROPHYLL_METABOLISM | 34 | -0.458991173 | -1.67468396 | 0.00530896467491958 | 0.0106179293498392 | 0.0037856793946795 | 10068 | tags=41%, list=24%, signal=31% | FECH/COX10/HMBS/COX15/UROS/HMOX2/FTH1/BLVRA/HCCS/GUSB/MMAB/HMOX1/ALAS1/UROD |
| KEGG_OLFACTORY_TRANSDUCTION | KEGG_OLFACTORY_TRANSDUCTION | KEGG_OLFACTORY_TRANSDUCTION | 178 | 0.324468112173511 | 1.50029979410849 | 0.00527303484451634 | 0.0106179293498392 | 0.0037856793946795 | 3476 | tags=12%, list=8%, signal=11% | OR11H4/OR4N4/OR4D6/OR2A14/OR4X2/OR1D4/OR2K2/OR2T34/OR2A42/OR2A4/OR6N2/OR2A1/OR1K1/OR2AE1/OR2F1/GNAL/CAMK2G/OR10G2/OR2C1/OR2AG2/OR5AN1/GUCA1B |
| KEGG_OTHER_GLYCAN_DEGRADATION | KEGG_OTHER_GLYCAN_DEGRADATION | KEGG_OTHER_GLYCAN_DEGRADATION | 14 | -0.604845688 | -1.757280218 | 0.00550523903055526 | 0.0108933453157796 | 0.0038838752399214 | 7301 | tags=57%, list=17%, signal=47% | HEXB/NEU4/AGA/HEXA/MAN2B1/FUCA2/GLB1/NEU1 |
| KEGG_VASOPRESSIN_REGULATED_WATER_REABSORPTION | KEGG_VASOPRESSIN_REGULATED_WATER_REABSORPTION | KEGG_VASOPRESSIN_REGULATED_WATER_REABSORPTION | 44 | -0.414365275 | -1.609667284 | 0.00562617978957031 | 0.0110154677985271 | 0.00392741636280254 | 14582 | tags=59%, list=35%, signal=38% | DYNLL2/RAB5A/NSF/AQP2/RAB5B/DYNC1I2/AVP/DCTN4/CREB3L3/CREB3L1/DCTN5/DYNC1I1/CREB3L4/PRKACA/STX4/DCTN6/AQP4/DYNLL1/ARHGDIB/DCTN1/DCTN2/RAB11B/CREB3/RAB5C/ADCY3/ARHGDIA |
| KEGG_NON_SMALL_CELL_LUNG_CANCER | KEGG_NON_SMALL_CELL_LUNG_CANCER | KEGG_NON_SMALL_CELL_LUNG_CANCER | 54 | -0.38718605 | -1.5933626 | 0.00623505540130039 | 0.0120804198400195 | 0.00430711063905619 | 5030 | tags=28%, list=12%, signal=24% | AKT2/EGFR/MAP2K1/ARAF/CCND1/CASP9/E2F2/CDKN2A/HRAS/GRB2/MAPK3/CDK4/E2F1/MAP2K2/BAD |
| KEGG_GLYCEROPHOSPHOLIPID_METABOLISM | KEGG_GLYCEROPHOSPHOLIPID_METABOLISM | KEGG_GLYCEROPHOSPHOLIPID_METABOLISM | 74 | -0.352390838 | -1.560701546 | 0.00642974610629149 | 0.0123292038739198 | 0.00439581122839245 | 12546 | tags=43%, list=30%, signal=30% | GPAT3/PLA2G2D/GPD1/ADPRM/DGKI/PLPP1/CHKA/CDS2/DGKB/ACHE/ETNK2/PLD1/GPAT2/TAFAZZIN/PLPP2/AGPAT1/LYPLA1/PLPP3/AGPAT3/PTDSS1/PGS1/CRLS1/PEMT/PCYT2/GPAT4/AGPAT2/LPCAT3/PLA2G15/PTDSS2/LYPLA2/PLA2G2C/CDIPT |
| KEGG_ERBB_SIGNALING_PATHWAY | KEGG_ERBB_SIGNALING_PATHWAY | KEGG_ERBB_SIGNALING_PATHWAY | 87 | -0.332662736 | -1.518093706 | 0.0065324767617064 | 0.0123983742620142 | 0.00442047299664343 | 9346 | tags=36%, list=22%, signal=28% | JUN/NRAS/SHC3/SRC/ABL1/PRKCG/SHC1/CDKN1A/ERBB3/PAK5/ERBB4/STAT5A/SHC2/AKT2/EGFR/MAP2K1/ARAF/NRG3/PAK4/CAMK2A/RPS6KB2/EIF4EBP1/MAPK10/SHC4/HRAS/CAMK2B/GRB2/MAPK3/MAP2K2/ELK1/BAD |
| KEGG_SPHINGOLIPID_METABOLISM | KEGG_SPHINGOLIPID_METABOLISM | KEGG_SPHINGOLIPID_METABOLISM | 37 | -0.419032308 | -1.564561531 | 0.00746817366917143 | 0.0140311141663221 | 0.00500260437169378 | 10580 | tags=51%, list=25%, signal=38% | SGPP2/PLPP1/SPTLC1/GALC/ARSA/UGCG/PLPP2/PLPP3/NEU4/DEGS1/SMPD2/SPHK1/SPTLC2/SMPD4/SPHK2/SMPD1/GLB1/NEU1/GLA |
| KEGG_GLYCOSPHINGOLIPID_BIOSYNTHESIS_GANGLIO_SERIES | KEGG_GLYCOSPHINGOLIPID_BIOSYNTHESIS_GANGLIO_SERIES | KEGG_GLYCOSPHINGOLIPID_BIOSYNTHESIS_GANGLIO_SERIES | 15 | -0.552926462 | -1.645558232 | 0.00834732505244407 | 0.015526024597546 | 0.00553559450846291 | 7301 | tags=53%, list=17%, signal=44% | HEXB/ST6GALNAC3/ST8SIA5/ST3GAL2/B3GALT4/HEXA/GLB1/B4GALNT1 |
| KEGG_MELANOGENESIS | KEGG_MELANOGENESIS | KEGG_MELANOGENESIS | 99 | -0.308849002 | -1.455144976 | 0.00864154215628516 | 0.0159141271392974 | 0.00567396723132845 | 11705 | tags=39%, list=28%, signal=28% | PRKCB/CALM1/EDN1/WNT9A/WNT6/DVL1/CREB3L3/CTNNB1/FZD10/FZD2/CREB3L1/NRAS/GNAO1/CREB3L4/ADCY1/WNT5A/PRKCG/DVL2/PRKACA/ADCY5/WNT11/CALM2/MAP2K1/ADCY2/ADCY8/WNT7B/PLCB3/CAMK2A/POMC/CALM3/GNAI2/HRAS/CAMK2B/CREB3/MAPK3/MAP2K2/ADCY3/FZD5/EDNRB |
| KEGG_HISTIDINE_METABOLISM | KEGG_HISTIDINE_METABOLISM | KEGG_HISTIDINE_METABOLISM | 29 | -0.476589202 | -1.671617372 | 0.00972750901095482 | 0.0177383987846823 | 0.00632438666346908 | 13455 | tags=62%, list=32%, signal=42% | AOC1/FTCD/ALDH3A1/ALDH9A1/METTL6/MAOA/ACY3/BUD23/UROC1/ALDH7A1/ASPA/ALDH1A3/ALDH1B1/ALDH3B1/ALDH2/MAOB/CNDP1/LCMT1 |
| KEGG_FOLATE_BIOSYNTHESIS | KEGG_FOLATE_BIOSYNTHESIS | KEGG_FOLATE_BIOSYNTHESIS | 11 | -0.64368717 | -1.741195752 | 0.00995979384251446 | 0.0179208825597501 | 0.00638944879040327 | 7556 | tags=73%, list=18%, signal=60% | ALPG/ALPL/GCH1/SPR/FPGS/GGH/DHFR/QDPR |
| KEGG_LIMONENE_AND_PINENE_DEGRADATION | KEGG_LIMONENE_AND_PINENE_DEGRADATION | KEGG_LIMONENE_AND_PINENE_DEGRADATION | 10 | -0.644697562 | -1.674414758 | 0.0100202784205054 | 0.0179208825597501 | 0.00638944879040327 | 11264 | tags=80%, list=27%, signal=58% | EHHADH/ALDH9A1/NAA80/ALDH7A1/ALDH1B1/ALDH2/HADHA/ECHS1 |
| KEGG_GLYCOSPHINGOLIPID_BIOSYNTHESIS_LACTO_AND_NEOLACTO_SERIES | KEGG_GLYCOSPHINGOLIPID_BIOSYNTHESIS_LACTO_AND_NEOLACTO_SERIES | KEGG_GLYCOSPHINGOLIPID_BIOSYNTHESIS_LACTO_AND_NEOLACTO_SERIES | 26 | -0.477297921 | -1.665809902 | 0.0103429163783228 | 0.0183217375844574 | 0.00653236823894069 | 8023 | tags=50%, list=19%, signal=40% | B4GALT3/B3GNT2/B3GALT5/FUT7/FUT4/B4GALT4/FUT5/B3GALT1/B4GALT1/B4GAT1/FUT9/ST3GAL4/B4GALT2 |
| KEGG_GNRH_SIGNALING_PATHWAY | KEGG_GNRH_SIGNALING_PATHWAY | KEGG_GNRH_SIGNALING_PATHWAY | 97 | -0.305267241 | -1.435570606 | 0.0108290856835124 | 0.0190019805389934 | 0.00677489968283296 | 8376 | tags=33%, list=20%, signal=26% | MAP2K6/SRC/ADCY1/GNRH2/PRKACA/ADCY5/PLD1/CALM2/EGFR/MAP2K1/CDC42/ADCY2/MAPK14/MAPK11/ADCY8/PRKCD/PLCB3/CAMK2A/MMP14/CALM3/MAPK10/ATF4/HRAS/CAMK2B/PTK2B/GRB2/MAPK3/MAP2K2/MAP2K3/PLA2G2C/ADCY3/ELK1 |
| KEGG_GLYCOSAMINOGLYCAN_DEGRADATION | KEGG_GLYCOSAMINOGLYCAN_DEGRADATION | KEGG_GLYCOSAMINOGLYCAN_DEGRADATION | 21 | -0.522182847 | -1.683178325 | 0.0113057372139389 | 0.0196529637550713 | 0.00700699896190998 | 7773 | tags=57%, list=19%, signal=47% | HPSE/HYAL2/HEXB/HS3ST3A1/HYAL3/NAGLU/HEXA/GNS/ARSB/GALNS/GUSB/GLB1 |
| KEGG_VEGF_SIGNALING_PATHWAY | KEGG_VEGF_SIGNALING_PATHWAY | KEGG_VEGF_SIGNALING_PATHWAY | 72 | -0.332778399 | -1.471196366 | 0.011737301066263 | 0.0202142407252307 | 0.0072071146898106 | 5701 | tags=29%, list=14%, signal=25% | SHC2/AKT2/MAP2K1/CDC42/MAPK14/MAPK11/CHP1/MAPKAPK3/CASP9/SPHK1/SPHK2/RAC1/HRAS/RAC3/HSPB1/MAPK3/MAP2K2/PLA2G2C/RAC2/BAD/MAPKAPK2 |
| KEGG_SULFUR_METABOLISM | KEGG_SULFUR_METABOLISM | KEGG_SULFUR_METABOLISM | 13 | -0.586055948 | -1.655854578 | 0.0121962010570255 | 0.0208118660239152 | 0.00742018992363698 | 14475 | tags=85%, list=35%, signal=55% | PAPSS2/PAPSS1/CHST13/SUOX/SULT1E1/SULT1A3/SULT2B1/SULT1A2/BPNT1/SULT1A1/SULT1A4 |
| KEGG_RNA_DEGRADATION | KEGG_RNA_DEGRADATION | KEGG_RNA_DEGRADATION | 55 | -0.372671474 | -1.535881779 | 0.0123745751244398 | 0.0209242815740527 | 0.00746027017071488 | 11027 | tags=44%, list=26%, signal=32% | HSPD1/CNOT10/HSPA9/EDC4/EXOSC6/XRN2/PATL1/CNOT9/MPHOSPH6/LSM5/C1DP2/EXOSC5/EXOSC7/EXOSC4/EXOSC1/DCPS/LSM6/LSM4/C1D/LSM7/ENO1/LSM1/LSM2/LSM3 |
| KEGG_ENDOMETRIAL_CANCER | KEGG_ENDOMETRIAL_CANCER | KEGG_ENDOMETRIAL_CANCER | 52 | -0.375731424 | -1.517764462 | 0.0125336313599724 | 0.0210023011977916 | 0.00748808701449276 | 5030 | tags=31%, list=12%, signal=27% | AKT2/EGFR/MAP2K1/ARAF/CCND1/ILK/CASP9/APC2/CTNNA2/HRAS/GRB2/CTNNA1/MAPK3/MAP2K2/ELK1/BAD |
| KEGG_VALINE_LEUCINE_AND_ISOLEUCINE_BIOSYNTHESIS | KEGG_VALINE_LEUCINE_AND_ISOLEUCINE_BIOSYNTHESIS | KEGG_VALINE_LEUCINE_AND_ISOLEUCINE_BIOSYNTHESIS | 10 | -0.632200798 | -1.641958041 | 0.0134858817138874 | 0.0223961964177059 | 0.00798506154111755 | 8141 | tags=70%, list=19%, signal=56% | VARS2/BCAT1/PDHA1/LARS2/BCAT2/PDHB/VARS1 |
| KEGG_NEUROACTIVE_LIGAND_RECEPTOR_INTERACTION | KEGG_NEUROACTIVE_LIGAND_RECEPTOR_INTERACTION | KEGG_NEUROACTIVE_LIGAND_RECEPTOR_INTERACTION | 250 | -0.230093967 | -1.262031998 | 0.0245480982977578 | 0.0404066042777252 | 0.0144064293689682 | 12928 | tags=44%, list=31%, signal=31% | CHRNA1/TSHR/FPR2/PRLHR/TAAR1/DRD5/HTR1F/HTR2C/GLRA2/MLNR/GHR/GRM8/ADRA1A/GABRG3/HRH1/PTAFR/GCGR/PLG/GRM5/NPFFR1/ADORA2B/CHRND/GRM6/CCKBR/HTR5A/RXFP2/RXFP1/TACR1/CHRNA3/NTSR2/GRM1/UTS2R/HTR4/NPY5R/GRIN2A/GPR156/BDKRB2/GZMA/P2RX5/CTSG/ADRA1B/GLP2R/SSTR4/HRH2/S1PR4/DRD1/GLRB/GABRA3/GRIN1/GABRB3/OPRL1/CHRM3/GRID1/CHRNA2/GALR1/ADRA2C/GABRG2/GPR35/DRD3/DRD2/SSTR1/GRIA4/LPAR3/CHRNA5/GABRG1/GABRA4/ADRA2B/GABRA1/P2RX1/F2/GRM3/GABRA2/APLNR/HCRTR1/PTGER1/ADORA1/GALR3/PTH1R/PTGER2/S1PR2/TSPO/PTGFR/PRL/LTB4R/CHRM1/FPR1/EDNRA/CNR1/GABRA5/GRIA1/NTSR1/GRID2/GRIN2B/GRIK3/HRH3/GRIK2/CHRNA4/C3AR1/GABRD/GABBR2/GALR2/GRIA3/ADORA2A/ADCYAP1R1/CHRM4/SSTR2/GRIA2/CHRNB1/P2RX4/EDNRB |
| KEGG_PRION_DISEASES | KEGG_PRION_DISEASES | KEGG_PRION_DISEASES | 34 | -0.408116341 | -1.489061076 | 0.0265930746139613 | 0.043011407636494 | 0.0153351368483255 | 7969 | tags=47%, list=19%, signal=38% | C8G/IL6/CCL5/PRKACA/MAP2K1/C1QC/SOD1/C1QB/NCAM2/C1QA/HSPA5/STIP1/MAPK3/BAX/MAP2K2/ELK1 |
| KEGG_WNT_SIGNALING_PATHWAY | KEGG_WNT_SIGNALING_PATHWAY | KEGG_WNT_SIGNALING_PATHWAY | 147 | -0.267201875 | -1.343479585 | 0.0264771593072764 | 0.043011407636494 | 0.0153351368483255 | 10729 | tags=33%, list=26%, signal=25% | WNT6/SFRP4/DKK2/FRAT1/LRP5/CSNK1A1/DVL1/CTNNB1/FZD10/FZD2/JUN/PPP2R1B/WIF1/CSNK1E/SFRP1/FOSL1/WNT5A/PRKCG/DVL2/PORCN/PRKACA/DAAM2/CTBP1/CACYBP/WNT11/CTNNBIP1/SKP1/PPP2CA/PPP2R5D/CUL1/RUVBL1/CHP1/CCND1/CSNK2A2/WNT7B/APC2/PLCB3/CAMK2A/CCND3/MAPK10/RHOA/RAC1/CAMK2B/RAC3/CSNK2B/RAC2/FZD5/RBX1/PPP2R1A |
| KEGG_O_GLYCAN_BIOSYNTHESIS | KEGG_O_GLYCAN_BIOSYNTHESIS | KEGG_O_GLYCAN_BIOSYNTHESIS | 29 | -0.439615507 | -1.541933631 | 0.0311114967418114 | 0.0498856758101459 | 0.0177860643805274 | 5025 | tags=34%, list=12%, signal=30% | GALNT14/GALNT17/B4GALT5/ST3GAL2/GALNT2/GALNT13/GALNT16/GALNT8/C1GALT1C1/GALNT15 |

**Table S7**

|  | ID | Description | setSize | enrichmentScore | NES | pvalue | p.adjust | qvalue | rank | leading_edge | core_enrichment |
| --- | --- | --- | --- | --- | --- | --- | --- | --- | --- | --- | --- |
| KEGG_SPLICEOSOME | KEGG_SPLICEOSOME | KEGG_SPLICEOSOME | 126 | -0.706894629 | -3.201429634 | 1e-10 | 1.43076923076923e-09 | 4.21052631578947e-10 | 9293 | tags=86%, list=22%, signal=67% | PQBP1/ZMAT2/PRPF40A/PRPF18/DHX15/TCERG1/SF3B1/SRSF10/SNRPG/NCBP2/LSM8/HNRNPA3/NCBP1/BCAS2/DDX46/U2AF1/HNRNPA1L2/HNRNPA1/TRA2A/SRSF3/LSM5/SRSF6/SNRPA1/CDC5L/AQR/SF3B5/WBP11/SNRPE/PPIE/PLRG1/DDX5/SRSF7/SMNDC1/RBMX/HNRNPC/TRA2B/DDX42/PPIH/SRSF4/PRPF40B/BUD31/SNRPD2/HSPA8/CDC40/SRSF5/LSM2/SNRPD1/SNRPF/SF3A2/SF3B6/CHERP/SF3A3/SNRNP40/PPIL1/MAGOH/HNRNPU/LSM4/SF3A1/LSM3/THOC3/PHF5A/PRPF3/SNRNP200/HNRNPM/SNW1/RBM8A/ACIN1/ALYREF/PCBP1/SNRPC/PRPF38A/SNRPB/SNRNP27/RBM17/LSM7/CTNNBL1/SRSF1/SNU13/LSM6/EIF4A3/DHX38/SNRPB2/PUF60/PRPF8/CCDC12/RBM22/SNRPA/HSPA1L/DDX39B/XAB2/HNRNPK/DHX16/SRSF2/PRPF19/DHX8/SNRPD3/PRPF31/PRPF4/SART1/EFTUD2/U2AF2/PRPF6/SF3B4/DDX23/ISY1/USP39/SF3B2/SRSF9 |
| KEGG_OXIDATIVE_PHOSPHORYLATION | KEGG_OXIDATIVE_PHOSPHORYLATION | KEGG_OXIDATIVE_PHOSPHORYLATION | 126 | -0.683259688 | -3.094390203 | 1e-10 | 1.43076923076923e-09 | 4.21052631578947e-10 | 7307 | tags=74%, list=17%, signal=61% | ATP6V0D1/NDUFV2/MT-ND3/COX7C/COX11/NDUFB1/TCIRG1/MT-ND1/COX6C/NDUFA3/NDUFA2/COX7A2/ATP5MF/UQCRC1/ATP5F1D/COX4I1/MT-ND2/ATP6V1F/ATP6V0E1/NDUFB7/ATP5PF/NDUFA11/ATP5MG/COX7A2L/NDUFA1/NDUFS5/ATP6AP1/ATP5ME/NDUFS8/NDUFS4/COX8A/UQCRQ/NDUFB8/COX6A1/ATP5MC3/SDHC/PPA1/NDUFA6/NDUFS7/COX5B/NDUFB6/ATP6V1G1/NDUFA8/CYC1/ATP5PO/ATP5MC2/NDUFS6/UQCR10/NDUFB9/ATP6V1E2/ATP6V1E1/NDUFB10/NDUFC1/NDUFB2/NDUFB4/NDUFA7/ATP6V1B2/MT-ATP6/MT-ND6/COX10/ATP5MC1/ATP5PB/MT-ND5/ATP5F1C/COX17/NDUFV1/NDUFS1/ATP5F1B/MT-CO2/UQCRFS1/MT-CO3/NDUFAB1/SDHA/ATP5F1A/ATP5PD/MT-CYB/ATP6V1H/MT-ND4/NDUFA9/MT-ND4L/NDUFB5/SDHB/NDUFS3/MT-ATP8/COX5A/PPA2/NDUFA10/NDUFC2/UQCRC2/MT-CO1/SDHD/COX15/NDUFS2 |
| KEGG_PARKINSONS_DISEASE | KEGG_PARKINSONS_DISEASE | KEGG_PARKINSONS_DISEASE | 125 | -0.656499742 | -2.967276029 | 1e-10 | 1.43076923076923e-09 | 4.21052631578947e-10 | 7094 | tags=72%, list=17%, signal=60% | NDUFV2/MT-ND3/COX7C/NDUFB1/MT-ND1/UBE2J1/COX6C/NDUFA3/CYCS/NDUFA2/COX7A2/UQCRC1/ATP5F1D/COX4I1/MT-ND2/NDUFB7/ATP5PF/COX7A2L/NDUFA1/NDUFS5/NDUFS8/NDUFS4/PPID/COX8A/UQCRQ/UBE2G2/NDUFB8/COX6A1/ATP5MC3/SDHC/NDUFA6/NDUFS7/COX5B/NDUFB6/NDUFA8/CYC1/ATP5PO/VDAC3/UBE2G1/ATP5MC2/NDUFS6/UQCR10/NDUFB9/NDUFB10/NDUFC1/SLC25A6/NDUFB2/NDUFB4/UBA1/NDUFA7/MT-ATP6/MT-ND6/ATP5MC1/ATP5PB/MT-ND5/ATP5F1C/NDUFV1/NDUFS1/ATP5F1B/MT-CO2/SLC25A5/UQCRFS1/MT-CO3/NDUFAB1/SDHA/PARK7/ATP5F1A/ATP5PD/MT-CYB/CASP9/MT-ND4/NDUFA9/UBA7/MT-ND4L/NDUFB5/SDHB/NDUFS3/MT-ATP8/UBE2J2/VDAC2P5/COX5A/NDUFA10/NDUFC2/VDAC1/VDAC2/UQCRC2/MT-CO1/SDHD/UBE2L3/NDUFS2 |
| KEGG_HUNTINGTONS_DISEASE | KEGG_HUNTINGTONS_DISEASE | KEGG_HUNTINGTONS_DISEASE | 176 | -0.61032225 | -2.912577947 | 1e-10 | 1.43076923076923e-09 | 4.21052631578947e-10 | 6875 | tags=62%, list=16%, signal=52% | BAX/CASP8/COX7C/POLR2B/AP2M1/NDUFB1/DNAH1/COX6C/POLR2A/NDUFA3/PLCB2/CYCS/SIN3A/NDUFA2/COX7A2/POLR2I/POLR2F/UQCRC1/ATP5F1D/COX4I1/AP2A1/TFAM/NDUFB7/ATP5PF/POLR2J/COX7A2L/POLR2L/NDUFA1/NDUFS5/CLTC/NDUFS8/NDUFS4/POLR2J2/DCTN2/PPID/COX8A/UQCRQ/POLR2H/CLTB/NDUFB8/COX6A1/ATP5MC3/SDHC/NDUFA6/CREB3/NDUFS7/COX5B/NDUFB6/NDUFA8/DLG4/POLR2K/TBP/CYC1/ATP5PO/VDAC3/ATP5MC2/NDUFS6/UQCR10/NDUFB9/TP53/NDUFB10/HTT/NDUFC1/SLC25A6/NDUFB2/NDUFB4/NDUFA7/MT-ATP6/ATP5MC1/ATP5PB/CLTA/ATP5F1C/NDUFV1/NDUFS1/ATP5F1B/MT-CO2/SLC25A5/UQCRFS1/MT-CO3/NDUFAB1/SDHA/ATP5F1A/ATP5PD/MT-CYB/CASP9/NDUFA9/POLR2E/NDUFB5/SDHB/POLR2D/NDUFS3/MT-ATP8/POLR2G/VDAC2P5/NRF1/COX5A/NDUFA10/NDUFC2/VDAC1/VDAC2/UQCRC2/DNAL4/MT-CO1/HDAC1/DCTN1/SDHD/POLR2C/AP2A2/NDUFS2/PLCB3 |
| KEGG_ALZHEIMERS_DISEASE | KEGG_ALZHEIMERS_DISEASE | KEGG_ALZHEIMERS_DISEASE | 161 | -0.615807112 | -2.904005639 | 1e-10 | 1.43076923076923e-09 | 4.21052631578947e-10 | 9719 | tags=72%, list=23%, signal=55% | NDUFB3/NDUFA5/ADAM10/ATP2A1/NAE1/MAPK1/TNF/PPP3CC/APOE/MAPK3/COX6B1/TNFRSF1A/CACNA1F/NDUFA4/EIF2AK3/LPL/NDUFV2/CASP8/COX7C/NDUFB1/LRP1/CDK5/COX6C/NDUFA3/PLCB2/ITPR2/CYCS/NDUFA2/GAPDH/COX7A2/ATF6/UQCRC1/ATP5F1D/APP/ADAM17/COX4I1/IDE/CALM2/NDUFB7/ATP5PF/COX7A2L/CAPN1/NDUFA1/NDUFS5/PSENEN/NDUFS8/NDUFS4/BID/COX8A/UQCRQ/NDUFB8/COX6A1/CAPN2/ATP5MC3/ATP2A2/SDHC/NDUFA6/GSK3B/CHP1/NDUFS7/COX5B/NDUFB6/NDUFA8/CYC1/BACE1/ATP5PO/ATP5MC2/CALM1/NDUFS6/UQCR10/GRIN2D/HSD17B10/NDUFB9/NDUFB10/NDUFC1/NDUFB2/PSEN1/NDUFB4/NDUFA7/MT-ATP6/ATP5MC1/ATP5PB/FADD/ATP2A3/ATP5F1C/NDUFV1/NDUFS1/ATP5F1B/MT-CO2/CASP7/UQCRFS1/MT-CO3/NDUFAB1/SDHA/APH1A/ATP5F1A/ATP5PD/CALM3/MT-CYB/CASP9/NDUFA9/NDUFB5/SDHB/NDUFS3/MT-ATP8/PSEN2/COX5A/NDUFA10/NDUFC2/PPP3CB/NCSTN/UQCRC2/MT-CO1/SDHD/NDUFS2/PLCB3 |
| KEGG_LYSOSOME | KEGG_LYSOSOME | KEGG_LYSOSOME | 120 | -0.610823869 | -2.743442301 | 1e-10 | 1.43076923076923e-09 | 4.21052631578947e-10 | 5878 | tags=60%, list=14%, signal=52% | IDUA/SORT1/CD63/CD164/HGSNAT/GAA/CTSK/NPC2/FUCA1/ENTPD4/MANBA/CD68/AP4B1/AP3B1/CTSS/PSAP/LAPTM5/ATP6AP1/CLTC/HYAL1/CLTB/LIPA/AP1M1/AP1S2/PLA2G15/ARSG/SMPD1/CTSH/NAGA/CTSO/AP3M1/SCARB2/MCOLN1/MAN2B1/ACP2/NEU1/GUSB/SGSH/AGA/CLTA/AP1S1/GNS/GLB1/SUMF1/AP3S1/GM2A/GALNS/HEXA/CLN3/LAPTM4A/NAGPA/NAGLU/CTSZ/HEXB/AP4M1/GALC/ATP6V1H/PPT1/TPP1/GGA1/GNPTG/AP3S2/GGA3/CTSA/LAMP1/AP1B1/SLC17A5/AP3D1/M6PR/ARSB/CTNS/CTSC |
| KEGG_NEUROTROPHIN_SIGNALING_PATHWAY | KEGG_NEUROTROPHIN_SIGNALING_PATHWAY | KEGG_NEUROTROPHIN_SIGNALING_PATHWAY | 124 | -0.585679445 | -2.640950621 | 1e-10 | 1.43076923076923e-09 | 4.21052631578947e-10 | 9104 | tags=61%, list=22%, signal=48% | FRS2/IRAK2/MAPK1/KIDINS220/MAPK11/RPS6KA1/MAPK3/MAP3K5/MAGED1/CAMK2D/SHC4/SH2B2/RPS6KA2/SHC2/PIK3R5/MAPK14/BAX/RAP1B/IRAK4/MAP2K2/BEX3/NFKBIE/CAMK2G/SORT1/CRK/PIK3CD/PTPN11/SH2B1/PDPK1/NGFR/TRAF6/YWHAH/MAPK7/CALM2/PRKCD/SH2B3/RAPGEF1/ATF4/PRDM4/PIK3CG/NRAS/GSK3B/IKBKB/CRKL/MAP3K3/MAP2K7/CALM1/RELA/TP53/YWHAQ/MAPK9/PSEN1/ARHGDIA/MAP2K5/ABL1/ARHGDIB/PIK3CB/MAP2K1/RPS6KA4/NFKBIB/HRAS/RHOA/CALM3/RAC1/MAPKAPK2/AKT1/YWHAE/YWHAZ/SHC1/IRAK1/CDC42/AKT2/CSK/GRB2/YWHAB/NFKB1 |
| KEGG_RNA_DEGRADATION | KEGG_RNA_DEGRADATION | KEGG_RNA_DEGRADATION | 55 | -0.660765967 | -2.57023105 | 1e-10 | 1.43076923076923e-09 | 4.21052631578947e-10 | 8671 | tags=71%, list=21%, signal=56% | DIS3/C1DP2/LSM8/DCP1A/LSM1/TENT4A/LSM5/PATL1/HSPD1/ENO3/CNOT8/CNOT2/EXOSC2/C1D/MPHOSPH6/ENO1/CNOT7/EXOSC9/CNOT1/LSM2/EXOSC7/HSPA9/PARN/CNOT3/EXOSC6/LSM4/LSM3/EXOSC5/EXOSC10/LSM7/EXOSC1/EXOSC3/LSM6/EDC3/CNOT10/CNOT9/DCPS/EDC4/XRN2 |
| KEGG_PYRIMIDINE_METABOLISM | KEGG_PYRIMIDINE_METABOLISM | KEGG_PYRIMIDINE_METABOLISM | 98 | -0.578997775 | -2.525164718 | 1e-10 | 1.43076923076923e-09 | 4.21052631578947e-10 | 7347 | tags=63%, list=18%, signal=52% | POLR1C/NT5C2/NME1/POLR2B/PRIM2/POLR1H/POLR1A/POLA1/POLD4/POLR3F/UCK2/POLR2A/UPP1/POLR1B/POLR2I/POLR2F/POLR3A/ENTPD4/NME1-NME2/CAD/TXNRD2/POLR3D/POLE4/POLR2J/POLR2L/CTPS1/POLD2/UMPS/DTYMK/POLE/NUDT2/POLR2J2/NME3/POLR2H/DUT/POLR3C/POLA2/POLD1/POLR1E/ENTPD6/POLD3/POLR2K/POLR3H/NME2/POLR3K/DPYD/DCTD/CANT1/UPRT/DHODH/POLR3GL/UCKL1/POLR2E/NT5C/POLE3/POLR2D/ITPA/UCK1/POLR2G/NME6/TK2/POLR2C |
| KEGG_PROTEASOME | KEGG_PROTEASOME | KEGG_PROTEASOME | 45 | -0.679289886 | -2.523397377 | 1e-10 | 1.43076923076923e-09 | 4.21052631578947e-10 | 9229 | tags=87%, list=22%, signal=68% | PSMD12/PSMC6/PSMB9/SEM1/PSMD14/PSMD1/PSMB5/PSMD4/PSMD6/PSMA3/POMP/PSMB6/PSME2/PSME3/PSMB3/PSME1/PSMC2/PSMB7/PSMD13/PSMB10/PSMC3/PSMD11/PSMA5/PSMA2/PSMA6/PSMB8/PSMD8/PSMA4/PSMC5/PSMA7/PSMC1/PSMB1/PSMD2/PSMA1/PSMC4/PSMD3/PSMD7/PSMB4/PSMB2 |
| KEGG_UBIQUITIN_MEDIATED_PROTEOLYSIS | KEGG_UBIQUITIN_MEDIATED_PROTEOLYSIS | KEGG_UBIQUITIN_MEDIATED_PROTEOLYSIS | 133 | -0.517099502 | -2.370801704 | 1e-10 | 1.43076923076923e-09 | 4.21052631578947e-10 | 11214 | tags=67%, list=27%, signal=49% | SMURF2/UBE2L6/WWP1/DDB2/BIRC6/SOCS1/UBE2R2/RBX1/ERCC8/FBXW8/XIAP/CUL3/UBE2S/ELOB/UBE2D3/UBE2Q2/PRKN/UBR5/CDC16/FBXW7/UBA6/SOCS3/ANAPC1/HUWE1/FBXO2/CUL2/CBL/UBE2J1/UBE4A/UBE3A/SYVN1/RCHY1/ANAPC13/SMURF1/TRIM37/TRAF6/SKP1/SKP2/CUL5/FBXO4/UBA2/ANAPC11/NHLRC1/CDC23/RHOBTB2/UBE2D4/UBE2E2/STUB1/UBE2G2/ANAPC7/MGRN1/PPIL2/WWP2/FBXW11/CUL4A/ANAPC10/CDC26/KLHL9/CUL7/COP1/SAE1/DDB1/UBA3/UBE3B/UBE2G1/UBOX5/UBE2F/UBE2N/UBA1/VHL/UBE2E1/ELOC/KEAP1/PIAS3/ANAPC2/UBE2A/RNF7/CUL1/UBA7/UBE2J2/PRPF19/PIAS4/UBE2D2/UBE3C/UBE2I/UBE2Z/ANAPC5/UBE2L3/UBE2Q1 |
| KEGG_FC_GAMMA_R_MEDIATED_PHAGOCYTOSIS | KEGG_FC_GAMMA_R_MEDIATED_PHAGOCYTOSIS | KEGG_FC_GAMMA_R_MEDIATED_PHAGOCYTOSIS | 96 | -0.544832395 | -2.358826502 | 1e-10 | 1.43076923076923e-09 | 4.21052631578947e-10 | 13160 | tags=68%, list=32%, signal=46% | FCGR1A/PLCG2/FCGR2C/LYN/PLPP2/PLA2G4B/PAK1/LIMK2/PIKFYVE/LAT/VASP/PLA2G6/DNM1/PLD1/MAPK1/MARCKS/MAPK3/DNM1L/HCK/WAS/PLA2G4A/PRKCE/PRKCA/PIK3R5/INPP5D/VAV2/CFL2/VAV3/SPHK1/GSN/CRK/PIK3CD/PLPP1/CFL1/PRKCD/RAC2/ARPC5/ARPC4/PIK3CG/RPS6KB2/CRKL/PIP4K2B/SYK/WASF2/PIK3CB/ARPC3/PIP5K1C/MAP2K1/ARPC1B/ARPC5L/SPHK2/ARF6/RAC1/PRKCB/PIP5K1A/AKT1/DNM2/CDC42/AKT2/ARPC2/ARPC1A/VAV1/LIMK1/PLD2/DOCK2 |
| KEGG_ENDOCYTOSIS | KEGG_ENDOCYTOSIS | KEGG_ENDOCYTOSIS | 181 | -0.491531099 | -2.356659813 | 1e-10 | 1.43076923076923e-09 | 4.21052631578947e-10 | 11718 | tags=60%, list=28%, signal=44% | CLTCL1/AP2S1/PDGFRA/IL2RB/PIKFYVE/SMURF2/EGF/WWP1/RABEP1/AP2B1/ARRB2/VPS37A/LDLRAP1/CXCR4/STAM2/VPS36/ERBB3/DNM1/PLD1/PSD4/ADRB2/DNM1L/RAB11FIP2/TSG101/RAB11FIP1/LDLR/CBL/DAB2/GRK5/AP2M1/HLA-A/EPN1/HLA-B/SH3GLB2/CHMP6/CSF1R/GRK6/USP8/AGAP2/CHMP2A/GRK4/HLA-E/EPS15/RUFY1/VPS28/HLA-F/ACAP3/VPS4B/VTA1/PRKCZ/ARAP1/SMURF1/VPS37B/TRAF6/AP2A1/CHMP2B/CHMP3/IQSEC2/CLTC/MVB12A/VPS45/RAB11FIP4/EHD1/HSPA8/EHD4/CLTB/ARRB1/CHMP4A/CHMP1B/RAB22A/RAB5C/VPS37C/PSD/SMAP1/IL2RG/PARD6A/PIP4K2B/SRC/GIT1/ACAP1/VPS25/PDCD6IP/MVB12B/SNF8/RNF41/CHMP4B/PIP5K1C/CLTA/RBSN/HRAS/GRK3/RAB11A/ARF6/STAMBP/GIT2/PIP5K1A/SH3KBP1/HSPA1L/ARFGAP3/ARFGAP2/DNM2/ARFGAP1/CDC42/RAB11B/HGS/AP2A2/VPS4A/PLD2/GRK2 |
| KEGG_AMINOACYL_TRNA_BIOSYNTHESIS | KEGG_AMINOACYL_TRNA_BIOSYNTHESIS | KEGG_AMINOACYL_TRNA_BIOSYNTHESIS | 41 | -0.687583965 | -2.517261597 | 1.26076327256202e-10 | 1.67052240679595e-09 | 4.91608178570398e-10 | 10187 | tags=88%, list=24%, signal=66% | WARS1/NARS1/LARS1/FARSB/EPRS1/IARS1/RARS1/EARS2/FARSA/MARS2/AARS2/DARS2/PARS2/DARS1/WARS2/SARS2/VARS2/SARS1/RARS2/AARS1/HARS2/FARS2/HARS1/YARS2/YARS1/VARS1/LARS2/GARS1/QARS1/TARS2/MARS1/MTFMT/IARS2/KARS1/CARS2/CARS1 |
| KEGG_PURINE_METABOLISM | KEGG_PURINE_METABOLISM | KEGG_PURINE_METABOLISM | 158 | -0.466822139 | -2.194253617 | 1.34719548935157e-10 | 1.67052240679595e-09 | 4.91608178570398e-10 | 8375 | tags=54%, list=20%, signal=44% | PFAS/PPAT/PDE4A/PAPSS2/ADCY9/AK4/POLR1C/NT5C2/ADSS2/PDE6H/NUDT5/GMPS/PKM/NME1/PDE6G/POLR2B/ADCY7/PDE2A/PRIM2/POLR1H/POLR1A/POLA1/POLD4/POLR3F/POLR2A/PDE7B/PDE6B/POLR1B/POLR2I/ADCY5/POLR2F/POLR3A/ENTPD4/PDE1B/PRUNE1/AMPD3/NME1-NME2/PRPS1/IMPDH2/POLR3D/POLE4/POLR2J/POLR2L/POLD2/POLE/NUDT2/POLR2J2/NME3/POLR2H/ADSS1/POLR3C/IMPDH1/ATIC/POLA2/POLD1/HPRT1/GART/POLR1E/ENTPD6/POLD3/POLR2K/PAPSS1/ADCY3/APRT/POLR3H/ADK/ADA/NME2/PRPS2/POLR3K/CANT1/DGUOK/ADSL/POLR3GL/POLR2E/NT5C/POLE3/POLR2D/NUDT9/ITPA/POLR2G/AK2/NME6/PDE6D/GMPR2/POLR2C |
| KEGG_DNA_REPLICATION | KEGG_DNA_REPLICATION | KEGG_DNA_REPLICATION | 36 | -0.698999851 | -2.469839273 | 1.7061367584677e-10 | 1.9833839817187e-09 | 5.8367836473895e-10 | 10564 | tags=92%, list=25%, signal=69% | PCNA/MCM2/PRIM1/RFC4/RPA4/RFC1/MCM3/MCM5/RNASEH2B/RPA3/MCM7/MCM6/FEN1/PRIM2/POLA1/POLD4/DNA2/LIG1/RFC5/RNASEH2A/RNASEH1/POLE4/RFC2/POLD2/POLE/RNASEH2C/POLA2/RPA2/POLD1/POLD3/RPA1/SSBP1/POLE3 |
| KEGG_CITRATE_CYCLE_TCA_CYCLE | KEGG_CITRATE_CYCLE_TCA_CYCLE | KEGG_CITRATE_CYCLE_TCA_CYCLE | 30 | -0.734424304 | -2.465412475 | 3.44708685140087e-10 | 3.6689916646837e-09 | 1.07972590018988e-09 | 6716 | tags=83%, list=16%, signal=70% | SUCLG2/DLD/IDH1/PCK2/DLAT/SDHC/SUCLG1/SUCLA2/IDH2/FH/MDH1/IDH3A/PDHA1/IDH3G/SDHA/OGDH/MDH2/PDHB/SDHB/IDH3B/DLST/ACO2/SDHD/ACLY/CS |
| KEGG_CHEMOKINE_SIGNALING_PATHWAY | KEGG_CHEMOKINE_SIGNALING_PATHWAY | KEGG_CHEMOKINE_SIGNALING_PATHWAY | 185 | -0.449738912 | -2.1606374 | 3.5506370948552e-10 | 3.6689916646837e-09 | 1.07972590018988e-09 | 13378 | tags=58%, list=32%, signal=40% | ROCK2/CCR4/GRK1/GNG11/SOS1/LYN/PAK1/CCR5/CCL2/PPBP/KRAS/JAK2/CCL23/CCL3/NFKBIA/PREX1/ROCK1/CCL4/GNB4/CXCR6/ARRB2/CXCR3/GNG7/PLCB1/CXCR4/CXCL14/GNB2/STAT1/CCL5/ADCY8/GNAI3/MAPK1/FGR/CXCL16/MAPK3/HCK/GNG2/CX3CR1/CCR9/WAS/JAK3/CX3CL1/SHC4/ADCY9/TIAM1/SHC2/PIK3R5/GNG4/CXCL9/STAT2/CCR2/RAP1B/VAV2/GRK5/PXN/ADCY7/GSK3A/VAV3/GRK6/GNB5/GRK4/PLCB2/CRK/PIK3CD/GNGT2/ADCY5/PRKX/PRKCZ/ELMO1/RASGRP2/CHUK/PRKCD/RAC2/STAT5B/ARRB1/PIK3CG/NRAS/GSK3B/IKBKB/CRKL/ADCY3/RELA/STAT3/GNG5/PIK3CB/PRKACA/MAP2K1/PTK2B/GNAI2/NFKBIB/HRAS/GRK3/RHOA/IKBKG/RAC1/PRKCB/AKT1/SHC1/CDC42/AKT2/CSK/VAV1/GRB2/GNB1/PLCB3/DOCK2/GRK2/NFKB1 |
| KEGG_PEROXISOME | KEGG_PEROXISOME | KEGG_PEROXISOME | 77 | -0.574627061 | -2.387439144 | 5.28459789007596e-10 | 5.1733431976533e-09 | 1.52243263315208e-09 | 10588 | tags=73%, list=25%, signal=54% | PECR/ACSL4/NOS2/FAR1/PEX13/SLC27A2/ACOX2/PEX3/CROT/ECI2/PXMP2/MPV17L/PRDX5/PEX1/PIPOX/PEX11G/PEX11A/AGPS/IDH1/ABCD1/SCP2/ACSL3/PAOX/AMACR/MVK/SLC25A17/ABCD4/PEX2/PRDX1/HMGCL/PEX11B/MLYCD/IDH2/ECH1/PHYH/PEX16/PEX14/PMVK/PEX10/NUDT19/PXMP4/PEX26/HACL1/PEX7/ACAA1/ACOX3/PEX6/HSD17B4/DHRS4/PEX5/ACOT8/PEX19/GSTK1/GNPAT/ACSL5/MPV17 |
| KEGG_TOLL_LIKE_RECEPTOR_SIGNALING_PATHWAY | KEGG_TOLL_LIKE_RECEPTOR_SIGNALING_PATHWAY | KEGG_TOLL_LIKE_RECEPTOR_SIGNALING_PATHWAY | 95 | -0.533862498 | -2.305279659 | 9.01384359474264e-10 | 8.38287454311065e-09 | 2.46694666803483e-09 | 12445 | tags=63%, list=30%, signal=44% | MAPK10/TLR5/TBK1/MAP2K6/CCL3/NFKBIA/TLR7/CCL4/CD40/STAT1/TLR8/SPP1/CCL5/TLR1/MAPK1/TNF/MAPK11/IL6/CD14/MAPK3/IFNAR1/TICAM1/PIK3R5/CXCL9/MAPK14/IRAK4/CASP8/MAP3K7/MAP2K2/TOLLIP/PIK3CD/CTSK/IFNAR2/IRF3/TRAF6/CHUK/MAP3K8/PIK3CG/CD86/IKBKB/RIPK1/MAP2K7/RELA/MAPK9/IRF5/PIK3CB/MAP2K1/FADD/MYD88/TAB1/TICAM2/IKBKG/RAC1/AKT1/TIRAP/IKBKE/IRAK1/AKT2/TRAF3/NFKB1 |
| KEGG_INSULIN_SIGNALING_PATHWAY | KEGG_INSULIN_SIGNALING_PATHWAY | KEGG_INSULIN_SIGNALING_PATHWAY | 136 | -0.477376912 | -2.195959459 | 1.98183591303515e-09 | 1.75534038011685e-08 | 5.16568759287358e-09 | 8945 | tags=53%, list=21%, signal=42% | MAPK1/FLOT1/RPTOR/PRKAG2/PRKAB2/PPP1R3D/MAPK3/SHC4/SH2B2/SOCS3/SHC2/PIK3R5/PRKAA1/INPP5D/CBL/MAP2K2/EIF4EBP1/CRK/PRKAB1/PRKAR1B/PIK3CD/LIPE/RHOQ/PRKX/GYS1/PRKCZ/PDPK1/HK1/PHKB/CALM2/PCK2/FBP1/RAPGEF1/INSR/PPP1CA/PIK3CG/RPS6KB2/SREBF1/NRAS/GSK3B/IKBKB/CRKL/PTPN1/HK2/CALM1/ACACA/MAPK9/MTOR/PPP1CC/TRIP10/PIK3CB/PRKACA/MAP2K1/FASN/HRAS/PRKAG1/TSC2/CALM3/PYGB/AKT1/PRKAR2A/ARAF/INPP5K/PHKG2/RHEB/SHC1/ELK1/AKT2/EXOC7/GRB2/PHKA2/EIF4E2 |
| KEGG_RNA_POLYMERASE | KEGG_RNA_POLYMERASE | KEGG_RNA_POLYMERASE | 29 | -0.71190956 | -2.372137167 | 3.43090674652831e-09 | 2.90067570388303e-08 | 8.53622731193647e-09 | 7347 | tags=86%, list=18%, signal=71% | POLR1C/POLR2B/POLR1H/POLR1A/POLR3F/POLR2A/POLR1B/POLR2I/POLR2F/POLR3A/POLR3D/POLR2J/POLR2L/POLR2J2/POLR2H/POLR3C/POLR1E/POLR2K/POLR3H/POLR3K/POLR3GL/POLR2E/POLR2D/POLR2G/POLR2C |
| KEGG_AMINO_SUGAR_AND_NUCLEOTIDE_SUGAR_METABOLISM | KEGG_AMINO_SUGAR_AND_NUCLEOTIDE_SUGAR_METABOLISM | KEGG_AMINO_SUGAR_AND_NUCLEOTIDE_SUGAR_METABOLISM | 43 | -0.642487584 | -2.382733856 | 7.13197690784923e-09 | 5.76759871678242e-08 | 1.69731258218838e-08 | 8764 | tags=72%, list=21%, signal=57% | FPGT/NANP/GFPT1/PGM1/CYB5R1/PGM3/PGM2/UGP2/RENBP/GPI/CYB5R3/GNE/HK1/GNPDA1/AMDHD2/GNPNAT1/PMM2/PMM1/NANS/HK2/MPI/GALT/GALE/UXS1/GMPPA/HEXA/GMPPB/HEXB/FCSK/GALK2/GMDS |
| KEGG_T_CELL_RECEPTOR_SIGNALING_PATHWAY | KEGG_T_CELL_RECEPTOR_SIGNALING_PATHWAY | KEGG_T_CELL_RECEPTOR_SIGNALING_PATHWAY | 107 | -0.495773245 | -2.185947112 | 8.36442402725916e-09 | 6.48242862112585e-08 | 1.90767565533981e-08 | 13861 | tags=68%, list=33%, signal=46% | PPP3CA/MAPK12/JUN/PAK3/NFATC3/PPP3R1/SOS1/TEC/PAK1/KRAS/PRKCQ/ICOS/LCK/CD28/NFKBIA/LAT/GRAP2/CARD11/CD8B/PAK2/DLG1/MALT1/LCP2/CD3D/MAPK1/TNF/MAPK11/IL10/NCK1/PPP3CC/MAPK3/NFATC1/FYN/PIK3R5/NCK2/MAPK14/VAV2/CBL/MAP3K7/MAP2K2/VAV3/BCL10/NFKBIE/PIK3CD/CD4/PDPK1/CHUK/CDK4/MAP3K14/MAP3K8/PIK3CG/NRAS/GSK3B/IKBKB/CHP1/MAP2K7/PAK4/RELA/MAPK9/PIK3CB/MAP2K1/PTPN6/NFKBIB/HRAS/RHOA/IKBKG/AKT1/PPP3CB/CDC42/AKT2/VAV1/GRB2/NFKB1 |
| KEGG_B_CELL_RECEPTOR_SIGNALING_PATHWAY | KEGG_B_CELL_RECEPTOR_SIGNALING_PATHWAY | KEGG_B_CELL_RECEPTOR_SIGNALING_PATHWAY | 74 | -0.544954326 | -2.23478248 | 2.12403772530762e-08 | 1.58028406762887e-07 | 4.65052470383143e-08 | 14204 | tags=72%, list=34%, signal=47% | CR2/PPP3CA/JUN/NFATC3/PLCG2/PPP3R1/SOS1/LYN/KRAS/NFKBIA/CARD11/CD79B/MALT1/MAPK1/LILRB3/PPP3CC/MAPK3/NFATC1/PIK3R5/INPP5D/PIK3AP1/VAV2/MAP2K2/VAV3/BCL10/NFKBIE/PIK3CD/CHUK/RAC2/PIK3CG/NRAS/GSK3B/IKBKB/CHP1/CD81/RELA/SYK/PIK3CB/MAP2K1/PTPN6/RAC3/NFKBIB/HRAS/IKBKG/RAC1/PRKCB/AKT1/BTK/PPP3CB/AKT2/VAV1/GRB2/NFKB1 |
| KEGG_NOTCH_SIGNALING_PATHWAY | KEGG_NOTCH_SIGNALING_PATHWAY | KEGG_NOTCH_SIGNALING_PATHWAY | 46 | -0.610366564 | -2.268744552 | 3.89205058326332e-08 | 2.7843131095653e-07 | 8.19379070160699e-08 | 12596 | tags=78%, list=30%, signal=55% | RFNG/NOTCH3/JAG2/PTCRA/JAG1/DLL3/HDAC2/DTX4/CTBP2/NOTCH4/RBPJ/KAT2A/NOTCH2/DTX1/DTX3/DLL4/DTX2/EP300/DLL1/LFNG/MAML3/ADAM17/PSENEN/DVL1/NCOR2/DVL2/MAML1/SNW1/MFNG/PSEN1/APH1A/PSEN2/CTBP1/NCSTN/HDAC1/DVL3 |
| KEGG_MAPK_SIGNALING_PATHWAY | KEGG_MAPK_SIGNALING_PATHWAY | KEGG_MAPK_SIGNALING_PATHWAY | 257 | -0.383262272 | -1.929969761 | 7.07629863767044e-08 | 4.87478350595075e-07 | 1.43457126377724e-07 | 15381 | tags=60%, list=37%, signal=38% | IL1B/RASA2/FGF7/CACNA1D/ELK4/FGF2/CACNA1G/FGF11/CACNA2D3/RASGRP4/CASP3/FGF9/MAPT/NTRK2/PLA2G5/CACNB1/PPP3CA/MAPK12/CACNG7/MAP3K1/PRKCG/CACNA1H/JUN/FASLG/PPP3R1/SOS1/GADD45B/PLA2G4B/MAPK10/PAK1/TGFBR1/CACNA2D1/KRAS/CACNB3/RPS6KA3/MAP2K6/CACNG4/MKNK1/PDGFRA/DUSP14/PLA2G12A/EGF/DUSP10/ARRB2/PLA2G6/STMN1/PAK2/RASA1/JMJD7-PLA2G4B/NF1/RRAS/MAP4K1/PPM1B/FGF1/MAPK1/TNF/DUSP5/MAP3K12/MAP3K4/MAPK11/RPS6KA1/CACNA1A/PPP3CC/CD14/STK4/DUSP16/MAPK3/DUSP2/MYC/TNFRSF1A/PLA2G2C/DUSP8/CACNA1F/MAP3K5/PLA2G4A/RPS6KA2/PRKCA/JUND/MAP4K3/NR4A1/DUSP6/STK3/MAPK14/RAP1B/MAP3K7/PDGFA/MAP3K13/FLNB/MAP2K2/NFKB2/RELB/MAPK8IP1/CRK/NLK/PRKX/MAPK8IP3/TRAF6/FGFR1/CACNA2D4/RASGRP2/TGFB1/MAPK7/CHUK/MRAS/DUSP7/RAC2/TGFBR2/ATF4/MAP3K6/MAX/MAP3K14/HSPA8/ARRB1/MAP3K8/MAPKAPK5/MAPKAPK3/NRAS/IKBKB/CHP1/CRKL/MAP3K3/MAP2K7/FLNA/RELA/PTPN7/TP53/MAPK9/MAP2K5/MAP3K11/PRKACA/MAP2K1/MAP4K2/RPS6KA4/TAOK3/TAOK2/RAC3/PPP5C/TRAF2/TAB1/HRAS/IKBKG/RAC1/PRKCB/MAPKAPK2/HSPA1L/DAXX/AKT1/DUSP3/PPP3CB/CDC42/ELK1/AKT2/SRF/GRB2/NFKB1 |
| KEGG_LYSINE_DEGRADATION | KEGG_LYSINE_DEGRADATION | KEGG_LYSINE_DEGRADATION | 44 | -0.609838658 | -2.267546241 | 7.71637863480511e-08 | 5.12588009312054e-07 | 1.50846499627769e-07 | 9018 | tags=66%, list=22%, signal=52% | NSD2/SUV39H2/PIPOX/AASDHPPT/ALDH1B1/AASDH/KMT5C/GCDH/HADHA/NSD1/SETD1A/SUV39H1/ACAT1/ALDH2/ACAT2/HADH/SETD7/PLOD3/EHMT2/ALDH3A2/KMT5A/TMLHE/EHMT1/ECHS1/OGDH/DOT1L/DLST/ALDH9A1/SETDB1 |
| KEGG_APOPTOSIS | KEGG_APOPTOSIS | KEGG_APOPTOSIS | 85 | -0.515636235 | -2.192417662 | 9.3023403048555e-08 | 5.96632860932111e-07 | 1.75579562922862e-07 | 9683 | tags=60%, list=23%, signal=46% | IRAK3/PRKAR1A/XIAP/IRAK2/TNF/PPP3CC/TNFRSF1A/PIK3R5/BAX/IRAK4/CASP8/EXOG/CYCS/PRKAR1B/PIK3CD/PRKX/DFFB/TNFRSF10B/AIFM1/CHUK/CAPN1/BID/TRADD/MAP3K14/PIK3CG/CAPN2/CASP10/IL3RA/TNFRSF10A/IKBKB/CHP1/RIPK1/ENDOG/RELA/TP53/TNFRSF10D/PIK3CB/PRKACA/FADD/MYD88/TRAF2/CASP7/CASP9/IKBKG/AKT1/PRKAR2A/DFFA/PPP3CB/IRAK1/AKT2/NFKB1 |
| KEGG_SNARE_INTERACTIONS_IN_VESICULAR_TRANSPORT | KEGG_SNARE_INTERACTIONS_IN_VESICULAR_TRANSPORT | KEGG_SNARE_INTERACTIONS_IN_VESICULAR_TRANSPORT | 38 | -0.638589556 | -2.286080859 | 1.19112079074213e-07 | 7.1467247444528e-07 | 2.10316744035962e-07 | 9985 | tags=74%, list=24%, signal=56% | VAMP7/VTI1B/VAMP1/VAMP4/SEC22B/STX4/STX16/STX10/GOSR1/STX5/USE1/BNIP1/STX6/STX1A/STX11/BET1/VAMP3/VAMP8/BET1L/TSNARE1/SNAP23/STX8/GOSR2/SNAP47/VTI1A/STX12/SNAP29/YKT6 |
| KEGG_NATURAL_KILLER_CELL_MEDIATED_CYTOTOXICITY | KEGG_NATURAL_KILLER_CELL_MEDIATED_CYTOTOXICITY | KEGG_NATURAL_KILLER_CELL_MEDIATED_CYTOTOXICITY | 123 | -0.456649922 | -2.061851837 | 1.17224135457763e-07 | 7.1467247444528e-07 | 2.10316744035962e-07 | 13942 | tags=59%, list=33%, signal=40% | PRF1/PPP3CA/PRKCG/KLRC2/FASLG/NFATC3/PLCG2/PPP3R1/SOS1/PAK1/ICAM1/GZMB/KRAS/KLRC1/LCK/LAT/LCP2/KIR2DL4/MAPK1/TNF/TYROBP/PPP3CC/MAPK3/NFATC1/FYN/SHC4/IFNAR1/SHC2/PRKCA/PIK3R5/FCER1G/VAV2/HLA-A/HLA-B/MAP2K2/VAV3/PIK3CD/HLA-E/PTPN11/ICAM2/IFNAR2/ITGAL/TNFRSF10B/HCST/CD244/RAC2/ULBP3/BID/PIK3CG/NRAS/ITGB2/CD48/TNFRSF10A/CHP1/MICA/SH3BP2/IFNGR2/SYK/TNFRSF10D/PIK3CB/MAP2K1/PTK2B/PTPN6/RAC3/HRAS/RAC1/PRKCB/ARAF/MICB/PPP3CB/SHC1/VAV1/GRB2 |
| KEGG_VALINE_LEUCINE_AND_ISOLEUCINE_DEGRADATION | KEGG_VALINE_LEUCINE_AND_ISOLEUCINE_DEGRADATION | KEGG_VALINE_LEUCINE_AND_ISOLEUCINE_DEGRADATION | 44 | -0.605355324 | -2.250875985 | 1.38282061449914e-07 | 8.03764482177625e-07 | 2.36535105111695e-07 | 10969 | tags=82%, list=26%, signal=60% | OXCT2/MMUT/HIBCH/ACADSB/BCKDHB/ACADM/DBT/ABAT/ALDH6A1/IL4I1/ALDH1B1/DLD/MCCC1/HMGCS1/HADHA/MCCC2/ACADS/PCCB/BCAT2/ACAT1/BCKDHA/ALDH2/IVD/HIBADH/ACAT2/HMGCL/HADH/HSD17B10/ALDH3A2/MCEE/ACAA2/ACAA1/ACAD8/ECHS1/ALDH9A1/HADHB |
| KEGG_PROTEIN_EXPORT | KEGG_PROTEIN_EXPORT | KEGG_PROTEIN_EXPORT | 24 | -0.712511294 | -2.250830978 | 1.58787778279889e-07 | 8.94985659395736e-07 | 2.6338004690763e-07 | 7058 | tags=88%, list=17%, signal=73% | SEC63/SEC11C/IMMP1L/SRP9P1/SRP54/SRP72/SRP19/SPCS3/SRPRB/SRP9/HSPA5/SEC61G/SPCS2/SRP68/SEC11A/OXA1L/SEC61B/SRP14/SPCS1/SEC61A1/SRPRA |
| KEGG_NUCLEOTIDE_EXCISION_REPAIR | KEGG_NUCLEOTIDE_EXCISION_REPAIR | KEGG_NUCLEOTIDE_EXCISION_REPAIR | 44 | -0.599070026 | -2.227505534 | 2.4107766437541e-07 | 1.31858859411666e-06 | 3.88039654182604e-07 | 10718 | tags=84%, list=26%, signal=63% | DDB2/PCNA/RFC4/RBX1/ERCC8/RPA4/RFC1/ERCC5/ERCC4/MNAT1/CCNH/RPA3/CDK7/GTF2H3/POLD4/LIG1/RFC5/XPC/XPA/POLE4/RFC2/POLD2/POLE/GTF2H4/CUL4A/GTF2H2/RPA2/POLD1/DDB1/POLD3/ERCC1/RPA1/ERCC3/GTF2H1/POLE3/RAD23B/GTF2H5 |
| KEGG_PHOSPHATIDYLINOSITOL_SIGNALING_SYSTEM | KEGG_PHOSPHATIDYLINOSITOL_SIGNALING_SYSTEM | KEGG_PHOSPHATIDYLINOSITOL_SIGNALING_SYSTEM | 75 | -0.522742741 | -2.150003186 | 2.48121509645608e-07 | 1.31858859411666e-06 | 3.88039654182604e-07 | 13685 | tags=67%, list=33%, signal=45% | PRKCG/ITPR3/ITPR1/PLCG2/PLCE1/INPP4A/IMPA1/PIKFYVE/PLCB1/DGKQ/DGKA/ITPKB/IMPA2/PLCD3/INPP5B/PRKCA/PIK3R5/INPP5D/CDS1/OCRL/SYNJ2/INPP5E/PIK3C3/PLCB2/ITPR2/PIK3CD/DGKD/CALM2/PLCD1/DGKZ/CDIPT/PIK3CG/DGKG/INPP5A/PIP4K2B/CALM1/PIK3CB/PIP5K1C/INPP1/PI4KA/CALM3/PRKCB/PIP5K1A/PI4KB/INPP5K/CDS2/PIP4K2C/PLCB3/INPPL1/IPPK |
| KEGG_REGULATION_OF_ACTIN_CYTOSKELETON | KEGG_REGULATION_OF_ACTIN_CYTOSKELETON | KEGG_REGULATION_OF_ACTIN_CYTOSKELETON | 206 | -0.397398515 | -1.946931345 | 3.45155775207014e-07 | 1.78330483856957e-06 | 5.24798254700724e-07 | 7498 | tags=39%, list=18%, signal=32% | ARHGAP35/TIAM1/PIK3R5/RDX/ARHGEF4/ITGA11/VAV2/CFL2/PXN/PDGFA/MAP2K2/ITGAM/VAV3/GSN/VCL/CRK/PIK3CD/APC2/ITGAL/BRK1/CFL1/FGFR1/PFN1/MRAS/PFN2/ARHGEF7/RAC2/TMSB4XP8/ARPC5/ACTB/ARHGEF1/ARPC4/ARHGEF6/PPP1CA/PIK3CG/TMSB4X/SSH3/NRAS/ITGB2/EZR/CRKL/MYH9/PAK4/PIP4K2B/GIT1/MYL5/MYL12A/ITGAE/CYFIP2/ACTG1/SSH1/WASF2/PPP1CC/DIAPH2/PIK3CB/ARPC3/PIP5K1C/MAP2K1/ARPC1B/RAC3/ARPC5L/HRAS/RHOA/ACTN4/MYL12B/RAC1/PIP5K1A/ARAF/DIAPH1/MSN/SLC9A1/CDC42/ARPC2/ARPC1A/CSK/CYFIP1/VAV1/LIMK1/PIP4K2C/NCKAP1L |
| KEGG_GLYCOSAMINOGLYCAN_DEGRADATION | KEGG_GLYCOSAMINOGLYCAN_DEGRADATION | KEGG_GLYCOSAMINOGLYCAN_DEGRADATION | 21 | -0.728655426 | -2.189079021 | 5.06372909171641e-07 | 2.54555030016014e-06 | 7.4911497231651e-07 | 5878 | tags=71%, list=14%, signal=61% | IDUA/HGSNAT/HYAL1/HPSE2/GUSB/SGSH/GNS/GLB1/GALNS/HPSE/HEXA/NAGLU/HEXB/HS3ST3A1/ARSB |
| KEGG_OTHER_GLYCAN_DEGRADATION | KEGG_OTHER_GLYCAN_DEGRADATION | KEGG_OTHER_GLYCAN_DEGRADATION | 14 | -0.794676555 | -2.186162797 | 8.97479185698112e-07 | 4.39292443525918e-06 | 1.29276780211362e-06 | 6996 | tags=93%, list=17%, signal=77% | ENGASE/FUCA1/MANBA/NEU3/MAN2C1/MAN2B1/NEU1/AGA/MAN2B2/GLB1/HEXA/HEXB/FUCA2 |
| KEGG_N_GLYCAN_BIOSYNTHESIS | KEGG_N_GLYCAN_BIOSYNTHESIS | KEGG_N_GLYCAN_BIOSYNTHESIS | 46 | -0.576006575 | -2.141027795 | 1.12409452067861e-06 | 5.22703952115556e-06 | 1.53823460724442e-06 | 6832 | tags=65%, list=16%, signal=55% | ALG11/ST6GAL1/DPM1/DPM3/ALG9/ALG2/B4GALT1/MGAT5/MGAT1/ALG6/ALG10/ALG5/DAD1/ALG1/ALG8/DOLPP1/MAN1B1/MOGS/RPN1/ALG3/ALG12/DDOST/MGAT2/STT3A/ALG14/RPN2/MGAT4B/GANAB/RFT1/DPAGT1 |
| KEGG_ANTIGEN_PROCESSING_AND_PRESENTATION | KEGG_ANTIGEN_PROCESSING_AND_PRESENTATION | KEGG_ANTIGEN_PROCESSING_AND_PRESENTATION | 74 | -0.505584913 | -2.073333951 | 1.11085040261465e-06 | 5.22703952115556e-06 | 1.53823460724442e-06 | 10621 | tags=58%, list=25%, signal=43% | CD8B/HLA-DQA1/HLA-DQB1/B2M/NFYB/RFXANK/KIR2DL4/HLA-DQA2/HSP90AA1/RFXAP/HLA-DRB5/TAP2/HSP90AB1/TAP1/HLA-DRB1/HLA-A/HLA-B/HLA-E/HLA-DPB1/HLA-F/LTA/CD4/HLA-DPA1/HLA-DRA/HLA-DMB/CIITA/HLA-DMA/CTSS/HSPA4/IFI30/TAPBP/HSPA8/PSME2/PSME3/HSPA5/PSME1/CALR/CD74/CANX/HSPA1L/RFX5/PDIA3/NFYC |
| KEGG_PROPANOATE_METABOLISM | KEGG_PROPANOATE_METABOLISM | KEGG_PROPANOATE_METABOLISM | 33 | -0.635683093 | -2.188023416 | 1.2526103903287e-06 | 5.68257396588143e-06 | 1.67229115011791e-06 | 10652 | tags=76%, list=26%, signal=56% | ACACB/MMUT/HIBCH/ACADM/ABAT/ALDH6A1/LDHB/ALDH1B1/SUCLG2/ACSS2/LDHA/HADHA/PCCB/ACAT1/ALDH2/ACSS1/SUCLG1/ACAT2/SUCLA2/MLYCD/ACACA/ALDH3A2/MCEE/ECHS1/ALDH9A1 |
| KEGG_OOCYTE_MEIOSIS | KEGG_OOCYTE_MEIOSIS | KEGG_OOCYTE_MEIOSIS | 109 | -0.451108909 | -1.997064431 | 1.52292466343204e-06 | 6.74438065234189e-06 | 1.98476397239263e-06 | 13861 | tags=63%, list=33%, signal=42% | PPP3CA/CAMK2B/MAPK12/ITPR3/ITPR1/FBXO5/PPP3R1/PKMYT1/PLK1/PPP2R5C/SMC3/CCNE1/RPS6KA3/ANAPC4/STAG3/YWHAG/RBX1/ADCY8/MAPK1/RPS6KA1/PPP3CC/MAPK3/CDC16/SLK/CAMK2D/ADCY9/REC8/ANAPC1/RPS6KA2/PPP2R5E/ADCY7/CAMK2G/PPP2R1B/ITPR2/ADCY5/PRKX/ANAPC13/CDK2/SKP1/YWHAH/CALM2/ANAPC11/CDC23/ANAPC7/PPP1CA/FBXW11/ANAPC10/SMC1A/CDC26/CHP1/MAD2L2/ADCY3/CALM1/PPP2CA/YWHAQ/PPP1CC/PRKACA/MAP2K1/ANAPC2/CALM3/CUL1/PPP2R1A/YWHAE/YWHAZ/PPP2R5D/PPP3CB/ANAPC5/PPP2CB/YWHAB |
| KEGG_LEUKOCYTE_TRANSENDOTHELIAL_MIGRATION | KEGG_LEUKOCYTE_TRANSENDOTHELIAL_MIGRATION | KEGG_LEUKOCYTE_TRANSENDOTHELIAL_MIGRATION | 112 | -0.450749623 | -1.994028104 | 1.88550990413289e-06 | 8.1559265620632e-06 | 2.40015948628911e-06 | 14750 | tags=62%, list=35%, signal=40% | CLDN20/CLDN1/ITGB1/MYL9/NCF2/CLDN23/CLDN10/MAPK12/ESAM/CDH5/PRKCG/CTNNA2/ROCK2/PLCG2/ICAM1/CLDN11/ROCK1/VASP/THY1/OCLN/CXCR4/GNAI3/MAPK11/VCAM1/JAM2/ARHGAP35/PRKCA/PIK3R5/MAPK14/RAP1B/VAV2/PXN/CLDN7/ITGAM/CYBA/JAM3/VAV3/F11R/PECAM1/CD99/VCL/PIK3CD/PTPN11/SIPA1/ITGAL/RAC2/RHOH/ACTB/PIK3CG/ITGB2/EZR/CTNNB1/MYL5/MYL12A/ACTG1/PIK3CB/PTK2B/RASSF5/GNAI2/RHOA/ACTN4/CYBB/MYL12B/CTNNA1/RAC1/PRKCB/MSN/CDC42/VAV1 |
| KEGG_ERBB_SIGNALING_PATHWAY | KEGG_ERBB_SIGNALING_PATHWAY | KEGG_ERBB_SIGNALING_PATHWAY | 87 | -0.479949552 | -2.046553739 | 2.57482179744916e-06 | 1.08844739619442e-05 | 3.20312759491283e-06 | 13855 | tags=66%, list=33%, signal=44% | CAMK2B/PRKCG/JUN/PAK3/NRG2/NRG4/CDKN1B/PLCG2/NRG1/SOS1/MAPK10/PAK1/ABL2/KRAS/EGF/PAK2/CDKN1A/ERBB3/MAPK1/NCK1/MAPK3/MYC/CAMK2D/SHC4/SHC2/PRKCA/PIK3R5/NCK2/CBL/MAP2K2/EIF4EBP1/CAMK2G/CRK/PIK3CD/STAT5B/PIK3CG/STAT5A/RPS6KB2/NRAS/GSK3B/CRKL/MAP2K7/PAK4/SRC/MAPK9/MTOR/ABL1/PIK3CB/MAP2K1/HRAS/PRKCB/AKT1/ARAF/SHC1/ELK1/AKT2/GRB2 |
| KEGG_RIG_I_LIKE_RECEPTOR_SIGNALING_PATHWAY | KEGG_RIG_I_LIKE_RECEPTOR_SIGNALING_PATHWAY | KEGG_RIG_I_LIKE_RECEPTOR_SIGNALING_PATHWAY | 63 | -0.527395182 | -2.098789609 | 2.8519537328476e-06 | 1.17880754291034e-05 | 3.469043137031e-06 | 8943 | tags=51%, list=21%, signal=40% | TNF/MAPK11/OTUD5/DDX3X/CYLD/MAPK14/ATG5/CASP8/SIKE1/MAP3K7/IRF3/TRAF6/NLRX1/CHUK/TRADD/MAVS/CASP10/IKBKB/RIPK1/STING1/RELA/PIN1/MAPK9/ATG12/FADD/TRAF2/NFKBIB/IKBKG/TKFC/IKBKE/TRAF3/NFKB1 |
| KEGG_VEGF_SIGNALING_PATHWAY | KEGG_VEGF_SIGNALING_PATHWAY | KEGG_VEGF_SIGNALING_PATHWAY | 72 | -0.502620023 | -2.052590759 | 3.36693897727391e-06 | 1.36141445602814e-05 | 4.00642624298039e-06 | 14935 | tags=69%, list=36%, signal=45% | BAD/KDR/PLA2G5/PPP3CA/MAPK12/PRKCG/NFATC3/PLCG2/PPP3R1/PLA2G4B/KRAS/PLA2G12A/VEGFA/PLA2G6/JMJD7-PLA2G4B/MAPK1/MAPK11/PPP3CC/NOS3/MAPK3/NFATC1/PLA2G2C/PLA2G4A/SHC2/PRKCA/PIK3R5/MAPK14/PXN/MAP2K2/SPHK1/PIK3CD/RAC2/PIK3CG/MAPKAPK3/NRAS/CHP1/SRC/PIK3CB/MAP2K1/RAC3/HRAS/SPHK2/CASP9/RAC1/PRKCB/MAPKAPK2/AKT1/PPP3CB/CDC42/AKT2 |
| KEGG_LONG_TERM_POTENTIATION | KEGG_LONG_TERM_POTENTIATION | KEGG_LONG_TERM_POTENTIATION | 69 | -0.505067948 | -2.044239239 | 3.59589834370842e-06 | 1.42305764240376e-05 | 4.18783233757755e-06 | 17654 | tags=80%, list=42%, signal=46% | RAP1A/ADCY1/CAMK4/PPP1CB/PRKACB/PPP3R2/PPP1R12A/CACNA1C/GRIA2/RAPGEF3/PPP3CA/CAMK2B/PRKCG/CREBBP/ITPR3/ITPR1/GRIN2C/PPP3R1/GRIA1/KRAS/GRIN2B/RPS6KA3/PLCB1/GNAQ/ADCY8/MAPK1/RPS6KA1/PPP3CC/MAPK3/EP300/CAMK2D/RPS6KA2/PRKCA/RAP1B/MAP2K2/CAMK2G/PLCB2/ITPR2/PRKX/CALM2/ATF4/PPP1CA/NRAS/CHP1/CALM1/GRIN2D/PPP1CC/PRKACA/MAP2K1/HRAS/CALM3/PRKCB/ARAF/PPP3CB/PLCB3 |
| KEGG_PROGESTERONE_MEDIATED_OOCYTE_MATURATION | KEGG_PROGESTERONE_MEDIATED_OOCYTE_MATURATION | KEGG_PROGESTERONE_MEDIATED_OOCYTE_MATURATION | 84 | -0.479874297 | -2.035353865 | 4.59546046151825e-06 | 1.78074092883832e-05 | 5.240437368398e-06 | 9866 | tags=48%, list=24%, signal=36% | CCNB3/ADCY8/GNAI3/CCNA1/MAPK1/HSP90AA1/MAPK11/RPS6KA1/MAPK3/CDC16/ADCY9/HSP90AB1/ANAPC1/RPS6KA2/PIK3R5/MAPK14/ADCY7/PIK3CD/ADCY5/PRKX/ANAPC13/CDK2/ANAPC11/CDC23/ANAPC7/PIK3CG/ANAPC10/CDC26/MAD2L2/ADCY3/MAPK9/PIK3CB/PRKACA/MAP2K1/GNAI2/ANAPC2/AKT1/ARAF/AKT2/ANAPC5 |
| KEGG_CHRONIC_MYELOID_LEUKEMIA | KEGG_CHRONIC_MYELOID_LEUKEMIA | KEGG_CHRONIC_MYELOID_LEUKEMIA | 73 | -0.48782616 | -1.996933235 | 6.30469197592753e-06 | 2.39320960718882e-05 | 7.04283528997275e-06 | 8945 | tags=56%, list=21%, signal=44% | MAPK1/CDKN2A/SMAD3/MAPK3/MYC/RB1/SHC4/SHC2/PIK3R5/CBL/MAP2K2/CRK/PIK3CD/PTPN11/TGFB1/CHUK/CDK4/TGFBR2/STAT5B/PIK3CG/STAT5A/NRAS/IKBKB/CRKL/BCR/RELA/TP53/ABL1/PIK3CB/MAP2K1/HRAS/IKBKG/AKT1/ARAF/CTBP1/SHC1/AKT2/RUNX1/HDAC1/GRB2/NFKB1 |
| KEGG_PYRUVATE_METABOLISM | KEGG_PYRUVATE_METABOLISM | KEGG_PYRUVATE_METABOLISM | 39 | -0.582046485 | -2.095648929 | 7.69428197679191e-06 | 2.86227289536659e-05 | 8.42321395354061e-06 | 7048 | tags=64%, list=17%, signal=53% | LDHB/GRHPR/ALDH1B1/PKM/DLD/GLO1/ACSS2/AKR1B1/LDHA/PCK2/DLAT/ACAT1/ALDH2/ACSS1/ACAT2/HAGHL/MDH1/ACACA/ALDH3A2/ACYP2/PDHA1/MDH2/PDHB/ME2/ALDH9A1 |
| KEGG_PENTOSE_PHOSPHATE_PATHWAY | KEGG_PENTOSE_PHOSPHATE_PATHWAY | KEGG_PENTOSE_PHOSPHATE_PATHWAY | 26 | -0.65146365 | -2.109841035 | 9.22976238441885e-06 | 3.30141500673443e-05 | 9.71553935201984e-06 | 9244 | tags=77%, list=22%, signal=60% | RPIA/G6PD/TKT/TKTL2/PGM1/ALDOC/PFKP/TALDO1/PGM2/ALDOA/GPI/PRPS1/FBP1/PGLS/RBKS/RPE/PRPS2/H6PD/PFKL/DERA |
| KEGG_TERPENOID_BACKBONE_BIOSYNTHESIS | KEGG_TERPENOID_BACKBONE_BIOSYNTHESIS | KEGG_TERPENOID_BACKBONE_BIOSYNTHESIS | 15 | -0.755441556 | -2.105420188 | 9.05534107061668e-06 | 3.30141500673443e-05 | 9.71553935201984e-06 | 5401 | tags=73%, list=13%, signal=64% | HMGCS1/MVK/ACAT1/PDSS1/HMGCR/ACAT2/FDPS/PMVK/MVD/PDSS2/DHDDS |
| KEGG_BASE_EXCISION_REPAIR | KEGG_BASE_EXCISION_REPAIR | KEGG_BASE_EXCISION_REPAIR | 34 | -0.604354435 | -2.09751642 | 9.99517707963976e-06 | 3.5077413902132e-05 | 1.03227250872149e-05 | 8320 | tags=74%, list=20%, signal=59% | HMGB1/MBD4/TDG/FEN1/PARP3/PARP4/UNG/POLD4/PARP1/LIG1/APEX2/APEX1/POLE4/POLD2/POLE/MPG/POLD1/POLD3/NTHL1/MUTYH/OGG1/POLE3/XRCC1/SMUG1/LIG3 |
| KEGG_ACUTE_MYELOID_LEUKEMIA | KEGG_ACUTE_MYELOID_LEUKEMIA | KEGG_ACUTE_MYELOID_LEUKEMIA | 57 | -0.510277594 | -1.987005851 | 1.14864719459401e-05 | 3.95645144804603e-05 | 1.16432074305825e-05 | 9620 | tags=56%, list=23%, signal=43% | CEBPA/PIM2/CCNA1/MAPK1/MAPK3/MYC/PIK3R5/PPARD/MAP2K2/EIF4EBP1/PIK3CD/CHUK/TCF7L2/STAT5B/PIK3CG/STAT5A/RPS6KB2/NRAS/IKBKB/RELA/STAT3/MTOR/PIK3CB/MAP2K1/HRAS/IKBKG/AKT1/ARAF/AKT2/RUNX1/GRB2/NFKB1 |
| KEGG_INOSITOL_PHOSPHATE_METABOLISM | KEGG_INOSITOL_PHOSPHATE_METABOLISM | KEGG_INOSITOL_PHOSPHATE_METABOLISM | 53 | -0.525414361 | -2.02514363 | 1.20179268576203e-05 | 4.06424435548614e-05 | 1.19604248152393e-05 | 8592 | tags=66%, list=21%, signal=53% | PLCG2/PLCE1/INPP4A/IMPA1/PIKFYVE/PLCB1/ITPKB/IMPA2/ISYNA1/PLCD3/ALDH6A1/INPP5B/OCRL/SYNJ2/INPP5E/PIK3C3/PLCB2/PIK3CD/PLCD1/CDIPT/PIK3CG/INPP5A/TPI1/PIP4K2B/PIK3CB/PIP5K1C/INPP1/PI4KA/PIP5K1A/PI4KB/INPP5K/PIP4K2C/PLCB3/INPPL1/IPPK |
| KEGG_LEISHMANIA_INFECTION | KEGG_LEISHMANIA_INFECTION | KEGG_LEISHMANIA_INFECTION | 70 | -0.48584634 | -1.971459596 | 1.57156710624545e-05 | 5.21984788860096e-05 | 1.53611822414969e-05 | 16969 | tags=77%, list=41%, signal=46% | ITGA4/IL12A/HLA-DOA/FCGR3A/IFNG/IL1B/TLR2/TLR4/FCGR2A/ITGB1/NCF2/MAPK12/JUN/FCGR1A/FCGR2C/JAK2/NFKBIA/HLA-DQA1/NOS2/HLA-DQB1/STAT1/HLA-DQA2/MAPK1/TNF/MAPK11/IL10/HLA-DRB5/MAPK3/MAPK14/IRAK4/HLA-DRB1/MAP3K7/ITGAM/CYBA/HLA-DPB1/HLA-DPA1/HLA-DRA/HLA-DMB/TRAF6/HLA-DMA/TGFB1/JAK1/ITGB2/C3/RELA/IFNGR2/PTPN6/MYD88/TAB1/NFKBIB/PRKCB/IRAK1/ELK1/NFKB1 |
| KEGG_FC_EPSILON_RI_SIGNALING_PATHWAY | KEGG_FC_EPSILON_RI_SIGNALING_PATHWAY | KEGG_FC_EPSILON_RI_SIGNALING_PATHWAY | 75 | -0.482015626 | -1.982495502 | 1.75905316599524e-05 | 5.7400682258792e-05 | 1.68921079652359e-05 | 13147 | tags=64%, list=31%, signal=44% | PLCG2/SOS1/LYN/PLA2G4B/MAPK10/KRAS/MAP2K6/PLA2G12A/LAT/PLA2G6/JMJD7-PLA2G4B/LCP2/MAPK1/TNF/MAPK11/MAPK3/PLA2G2C/FYN/PLA2G4A/PRKCE/PRKCA/PIK3R5/FCER1G/INPP5D/MAPK14/VAV2/MAP2K2/VAV3/PIK3CD/PDPK1/PRKCD/RAC2/PIK3CG/NRAS/MAP2K7/MAPK9/SYK/PIK3CB/MAP2K1/RAC3/HRAS/RAC1/PRKCB/AKT1/BTK/AKT2/VAV1/GRB2 |
| KEGG_COLORECTAL_CANCER | KEGG_COLORECTAL_CANCER | KEGG_COLORECTAL_CANCER | 62 | -0.505889158 | -2.008262213 | 2.01787527016683e-05 | 6.47111724570743e-05 | 1.90434689743512e-05 | 8945 | tags=53%, list=21%, signal=42% | MAPK1/SMAD2/SMAD3/MSH6/MAPK3/MYC/APPL1/PIK3R5/BAX/RALGDS/CYCS/PIK3CD/APC2/MSH3/TGFB1/TCF7L2/RAC2/TGFBR2/PIK3CG/GSK3B/CTNNB1/TP53/MAPK9/MLH1/PIK3CB/MAP2K1/RAC3/RHOA/CASP9/RAC1/AKT1/ARAF/AKT2 |
| KEGG_FATTY_ACID_METABOLISM | KEGG_FATTY_ACID_METABOLISM | KEGG_FATTY_ACID_METABOLISM | 40 | -0.557212625 | -2.0214674 | 2.32258491288605e-05 | 7.26410830321038e-05 | 2.13771155499117e-05 | 10576 | tags=70%, list=25%, signal=52% | ACSL4/CPT1C/ACADSB/ACADM/ECI2/CPT1B/ALDH1B1/ACSL3/GCDH/HADHA/ACADS/ACAT1/ALDH2/ACAT2/HADH/ECI1/CPT1A/ALDH3A2/ACAA2/CPT2/ACAA1/ADH5/ECHS1/ACOX3/ACADVL/ALDH9A1/ACSL5/HADHB |
| KEGG_VIRAL_MYOCARDITIS | KEGG_VIRAL_MYOCARDITIS | KEGG_VIRAL_MYOCARDITIS | 67 | -0.481005772 | -1.938753814 | 2.34326074297109e-05 | 7.26410830321038e-05 | 2.13771155499117e-05 | 12315 | tags=60%, list=29%, signal=42% | ICAM1/ABL2/SGCA/CD55/CD28/MYH3/CD40/HLA-DQA1/HLA-DQB1/HLA-DQA2/HLA-DRB5/FYN/HLA-DRB1/CASP8/HLA-A/EIF4G3/HLA-B/CYCS/HLA-E/HLA-DPB1/HLA-F/ITGAL/HLA-DPA1/HLA-DRA/HLA-DMB/HLA-DMA/RAC2/ACTB/BID/EIF4G2/CD86/ITGB2/MYH9/ACTG1/ABL1/DAG1/RAC3/CASP9/RAC1/EIF4G1 |
| KEGG_TIGHT_JUNCTION | KEGG_TIGHT_JUNCTION | KEGG_TIGHT_JUNCTION | 128 | -0.406612574 | -1.847933966 | 2.46645683300509e-05 | 7.52067165473682e-05 | 2.21321406930569e-05 | 15602 | tags=59%, list=37%, signal=37% | CTTN/PRKCI/ACTN2/PRKCH/PPP2R2A/CLDN20/CLDN1/MYL9/CLDN23/CLDN10/PARD3/MYH14/MYH7B/PRKCG/MAGI2/CTNNA2/EPB41L1/ASH1L/KRAS/PRKCQ/PALS1/MYH3/CLDN11/CRB3/OCLN/RAB3B/RRAS/GNAI3/JAM2/CASK/PRKCE/PRKCA/CLDN7/JAM3/F11R/PPP2R1B/TJP2/PRKCZ/VAPA/PRKCD/MRAS/CDK4/LLGL1/ACTB/HCLS1/SPTAN1/CSNK2A2/NRAS/CSNK2A1/EPB41L3/CTNNB1/MYH9/PARD6A/SRC/MYL5/MYL12A/SYMPK/PPP2CA/ACTG1/CSNK2B/GNAI2/HRAS/RHOA/ACTN4/MYL12B/CTNNA1/PRKCB/PPP2R2D/AKT1/PPP2R1A/TJAP1/CDC42/AKT2/PPP2CB/EXOC4/EXOC3 |
| KEGG_MISMATCH_REPAIR | KEGG_MISMATCH_REPAIR | KEGG_MISMATCH_REPAIR | 23 | -0.656551662 | -2.045238687 | 2.57407588444535e-05 | 7.72222765333605e-05 | 2.27252879441695e-05 | 10564 | tags=83%, list=25%, signal=62% | PCNA/RFC4/RPA4/RFC1/MSH6/RPA3/POLD4/LIG1/RFC5/MSH3/RFC2/POLD2/PMS2/RPA2/POLD1/POLD3/RPA1/MLH1/SSBP1 |
| KEGG_MATURITY_ONSET_DIABETES_OF_THE_YOUNG | KEGG_MATURITY_ONSET_DIABETES_OF_THE_YOUNG | KEGG_MATURITY_ONSET_DIABETES_OF_THE_YOUNG | 21 | 0.70430702521045 | 2.29604780335062 | 4.23654472515646e-05 | 0.000125078939504619 | 3.68087428083769e-05 | 724 | tags=24%, list=2%, signal=23% | FOXA2/MAFA/NKX6-1/NR5A2/PKLR |
| KEGG_CELL_CYCLE | KEGG_CELL_CYCLE | KEGG_CELL_CYCLE | 124 | -0.410469145 | -1.85089088 | 4.43761096626405e-05 | 0.000128968068707049 | 3.7953251685153e-05 | 14980 | tags=64%, list=36%, signal=41% | CDKN2D/CDKN2C/E2F5/SMC1B/ORC5/MAD2L1/CREBBP/RBL2/CHEK1/ATR/CDKN1B/GADD45B/PKMYT1/PLK1/SMC3/MCM4/E2F3/CCNE1/CDC45/ANAPC4/ORC3/HDAC2/PCNA/MCM2/YWHAG/ORC6/RBX1/CCNB3/CDKN1A/CCNA1/RBL1/CDKN1C/SMAD2/CDKN2A/SMAD3/MYC/EP300/RB1/CDC16/MCM3/MCM5/CCNH/ANAPC1/MCM7/MCM6/CCND3/CDK7/CCND2/BUB3/MAD1L1/STAG1/ANAPC13/CDK2/SKP1/SKP2/TGFB1/YWHAH/CDK4/ANAPC11/CDC23/ANAPC7/E2F4/CHEK2/ANAPC10/SMC1A/CDC26/GSK3B/MAD2L2/TP53/YWHAQ/ABL1/ANAPC2/CUL1/ZBTB17/YWHAE/YWHAZ/HDAC1/ANAPC5/YWHAB |
| KEGG_PATHOGENIC_ESCHERICHIA_COLI_INFECTION | KEGG_PATHOGENIC_ESCHERICHIA_COLI_INFECTION | KEGG_PATHOGENIC_ESCHERICHIA_COLI_INFECTION | 56 | -0.50650901 | -1.972281946 | 5.31343548838922e-05 | 0.000152046000129291 | 4.4744719902225e-05 | 8658 | tags=55%, list=21%, signal=44% | NCK1/CD14/FYN/WAS/TUBB4A/PRKCA/NCK2/TUBA3D/NCL/TUBB/ARPC5/ACTB/ARPC4/HCLS1/EZR/CTNNB1/TUBA1B/TUBA1A/YWHAQ/ACTG1/ABL1/ARPC3/ARPC1B/ARPC5L/RHOA/ARHGEF2/TUBA1C/YWHAZ/CDC42/ARPC2/ARPC1A |
| KEGG_WNT_SIGNALING_PATHWAY | KEGG_WNT_SIGNALING_PATHWAY | KEGG_WNT_SIGNALING_PATHWAY | 147 | -0.387052255 | -1.809114883 | 5.43571768630376e-05 | 0.000153188407523106 | 4.50809122309083e-05 | 13861 | tags=57%, list=33%, signal=38% | PPP3CA/CAMK2B/APC/PRKCG/CREBBP/JUN/ROCK2/SIAH1/NFATC3/PPP3R1/FZD9/FZD8/PPP2R5C/MAPK10/WNT11/WNT16/WNT8B/FZD3/ROCK1/AXIN2/PLCB1/CTBP2/RBX1/FRAT1/SMAD2/SMAD3/PPP3CC/PRICKLE1/MYC/EP300/NFATC1/CAMK2D/PPP2R5E/PRKCA/CCND3/CSNK1E/PPARD/FZD2/MAP3K7/TBL1X/CCND2/CACYBP/CAMK2G/PPP2R1B/PLCB2/NLK/APC2/PRKX/SKP1/PORCN/TCF7L2/RAC2/CHD8/RUVBL1/SENP2/FBXW11/DVL1/CSNK2A2/CSNK2A1/GSK3B/CHP1/DVL2/CTNNB1/CTNNBIP1/CSNK1A1/TP53/PPP2CA/CSNK2B/MAPK9/PSEN1/PRKACA/RAC3/FZD1/RHOA/RAC1/PRKCB/CUL1/PPP2R1A/PPP2R5D/CTBP1/PPP3CB/PPP2CB/DVL3/PLCB3 |
| KEGG_SPHINGOLIPID_METABOLISM | KEGG_SPHINGOLIPID_METABOLISM | KEGG_SPHINGOLIPID_METABOLISM | 37 | -0.558283596 | -1.987217376 | 6.12822287958822e-05 | 0.000165195573275856 | 4.86144301660698e-05 | 9855 | tags=59%, list=24%, signal=45% | SGMS2/DEGS2/ARSA/UGCG/ASAH1/SPHK1/PLPP1/ACER3/SPTLC2/NEU3/SMPD2/SMPD1/SMPD4/NEU1/DEGS1/SPTLC1/GLB1/SPHK2/GALC/SGPL1/CERK/KDSR |
| KEGG_GLYCOLYSIS_GLUCONEOGENESIS | KEGG_GLYCOLYSIS_GLUCONEOGENESIS | KEGG_GLYCOLYSIS_GLUCONEOGENESIS | 59 | -0.492022349 | -1.929409105 | 6.08851042531102e-05 | 0.000165195573275856 | 4.86144301660698e-05 | 8087 | tags=58%, list=19%, signal=47% | PGAM4/PGM1/ALDOC/LDHB/ALDH1B1/PKM/PFKP/DLD/ALDH3B1/PGM2/ACSS2/ENO3/ALDOA/GAPDH/GPI/LDHA/HK1/PCK2/ENO1/FBP1/DLAT/PGK1/ALDH2/ACSS1/TPI1/HK2/ALDH3A2/PGAM1/AKR1A1/PDHA1/ADH5/PDHB/PFKL/ALDH9A1 |
| KEGG_GLYCEROPHOSPHOLIPID_METABOLISM | KEGG_GLYCEROPHOSPHOLIPID_METABOLISM | KEGG_GLYCEROPHOSPHOLIPID_METABOLISM | 74 | -0.45897645 | -1.882199079 | 5.9988416414331e-05 | 0.000165195573275856 | 4.86144301660698e-05 | 10884 | tags=58%, list=26%, signal=43% | LPCAT2/ETNK1/PLA2G6/DGKQ/JMJD7-PLA2G4B/DGKA/PLD1/LPGAT1/PLA2G2C/CHKA/PLA2G4A/LCAT/AGPAT2/PGS1/MBOAT1/CDS1/GPD2/LPCAT1/PLPP1/DGKD/CHKB/GPD1L/PEMT/DGKZ/CDIPT/DGKG/PLA2G15/AGPAT1/LPCAT3/LPCAT4/PTDSS1/PCYT1A/PCYT2/PTDSS2/AGPAT3/LYPLA2/TAFAZZIN/CDS2/CRLS1/GNPAT/LYPLA1/GPAT4/PLD2 |
| KEGG_FRUCTOSE_AND_MANNOSE_METABOLISM | KEGG_FRUCTOSE_AND_MANNOSE_METABOLISM | KEGG_FRUCTOSE_AND_MANNOSE_METABOLISM | 34 | -0.575266645 | -1.996562222 | 6.86670763831484e-05 | 0.000182458231532366 | 5.36945559687777e-05 | 7192 | tags=59%, list=17%, signal=49% | ALDOC/PFKFB3/PFKP/ALDOA/AKR1B1/HK1/PHPT1/FBP1/MTMR1/PMM2/PMM1/TPI1/HK2/MPI/KHK/GMPPA/GMPPB/FCSK/GMDS/PFKL |
| KEGG_EPITHELIAL_CELL_SIGNALING_IN_HELICOBACTER_PYLORI_INFECTION | KEGG_EPITHELIAL_CELL_SIGNALING_IN_HELICOBACTER_PYLORI_INFECTION | KEGG_EPITHELIAL_CELL_SIGNALING_IN_HELICOBACTER_PYLORI_INFECTION | 68 | -0.462243162 | -1.867130789 | 8.4606479595138e-05 | 0.000221645143728108 | 6.52266410518485e-05 | 13852 | tags=66%, list=33%, signal=44% | MAPK12/JUN/ATP6V1C2/PLCG2/LYN/PTPRZ1/MAPK10/PAK1/NFKBIA/ATP6V0A2/ATP6V1G2/ATP6V0B/ADAM10/CCL5/MAPK11/ATP6V0E2/JAM2/ATP6V0D1/MAPK14/TCIRG1/JAM3/F11R/PTPN11/ADAM17/ATP6V1F/ATP6V0E1/CHUK/ATP6AP1/MAP3K14/NOD1/IKBKB/ATP6V1G1/SRC/RELA/GIT1/ATP6V1E2/ATP6V1E1/MAPK9/ATP6V1B2/ATP6V1H/IKBKG/RAC1/CDC42/CSK/NFKB1 |
| KEGG_PATHWAYS_IN_CANCER | KEGG_PATHWAYS_IN_CANCER | KEGG_PATHWAYS_IN_CANCER | 319 | -0.314763216 | -1.619141767 | 8.58501474613551e-05 | 0.000221779547608501 | 6.52661939764688e-05 | 14369 | tags=55%, list=34%, signal=36% | RARA/CASP3/FGF9/PML/HGF/TRAF5/PPARG/APC/RARB/PRKCG/CREBBP/JUN/CTNNA2/PIAS2/CDKN1B/FASLG/PLCG2/SOS1/RXRA/FLT3LG/FZD9/FZD8/SMO/SPI1/MAPK10/RALB/WNT11/TGFBR1/COL4A6/ITGAV/RAD51/KRAS/E2F3/CCNE1/FN1/WNT16/WNT8B/FZD3/PDGFRA/COL4A1/NFKBIA/LAMA4/EGF/VEGFA/HDAC2/ITGA2B/AXIN2/NOS2/TRAF1/TPR/CTBP2/RBX1/BMP2/CDKN1A/CEBPA/STAT1/XIAP/CSF2RA/CCNA1/PLD1/FGF1/LAMB2/PTCH2/MITF/MAPK1/ELOB/HSP90AA1/SMAD2/IL6/CDKN2A/SMAD3/MSH6/STK4/MAPK3/MYC/EP300/RB1/HSP90B1/HSP90AB1/EGLN2/APPL1/PRKCA/PIK3R5/CUL2/BAX/RALGDS/VEGFB/PPARD/CASP8/CBL/FZD2/PDGFA/MAP2K2/NFKB2/CSF1R/CKS1B/CRK/RASSF1/CYCS/PIK3CD/ARNT/STK36/APC2/EPAS1/PAX8/MSH3/CCDC6/TRAF6/FGFR1/CDK2/TFG/SKP2/TGFB1/CHUK/CDK4/TCF7L2/RAC2/JAK1/TGFBR2/BID/MAX/STAT5B/GSTP1/PIK3CG/DVL1/STAT5A/NRAS/DAPK1/GSK3B/IKBKB/DVL2/CTNNB1/CRKL/BCR/RELA/FH/TP53/STAT3/MAPK9/DAPK3/MTOR/MLH1/ABL1/PIK3CB/MAP2K1/VHL/SUFU/FADD/RASSF5/RAC3/ELOC/TRAF2/RXRB/HRAS/FZD1/RHOA/PIAS3/CASP9/IKBKG/CTNNA1/RAC1/RALA/PRKCB/AKT1/ARAF/PIAS4/CTBP1/CDC42/AKT2/RUNX1/HDAC1/TPM3/GRB2/TRAF3/DVL3/NFKB1 |
| KEGG_BASAL_TRANSCRIPTION_FACTORS | KEGG_BASAL_TRANSCRIPTION_FACTORS | KEGG_BASAL_TRANSCRIPTION_FACTORS | 33 | -0.572160554 | -1.969378614 | 8.92352579097521e-05 | 0.000227366547550875 | 6.69103592113498e-05 | 11075 | tags=82%, list=27%, signal=60% | TAF13/TAF9B/TAF4/TAF1/GTF2IRD1/TAF5/TAF7/GTF2I/TAF2/GTF2H3/TAF9/GTF2F2/TAF11/GTF2H4/GTF2H2/TAF12/TBP/GTF2E1/STON1/TAF6L/TAF10/TAF5L/TAF6/GTF2A2/GTF2H1/GTF2F1/GTF2E2 |
| KEGG_BUTANOATE_METABOLISM | KEGG_BUTANOATE_METABOLISM | KEGG_BUTANOATE_METABOLISM | 33 | -0.571382235 | -1.96669964 | 9.25710600395705e-05 | 0.000232678610369731 | 6.84736148230109e-05 | 11545 | tags=67%, list=28%, signal=48% | ACSM5/OXCT2/BDH2/L2HGDH/ABAT/ALDH1B1/ACSM1/HMGCS1/HADHA/ACADS/BDH1/ACAT1/ALDH2/ACAT2/HMGCL/HADH/ALDH3A2/AACS/PDHA1/ECHS1/PDHB/ALDH9A1 |
| KEGG_GALACTOSE_METABOLISM | KEGG_GALACTOSE_METABOLISM | KEGG_GALACTOSE_METABOLISM | 26 | -0.611866937 | -1.981602461 | 0.000111909837468915 | 0.000273565288789121 | 8.05059140748971e-05 | 10376 | tags=65%, list=25%, signal=49% | HK3/GALK1/PGM1/PFKP/PGM2/UGP2/GAA/AKR1B1/HK1/B4GALT1/HK2/GALT/GALE/GLB1/GANC/GALK2/PFKL |
| KEGG_GLYOXYLATE_AND_DICARBOXYLATE_METABOLISM | KEGG_GLYOXYLATE_AND_DICARBOXYLATE_METABOLISM | KEGG_GLYOXYLATE_AND_DICARBOXYLATE_METABOLISM | 15 | -0.70965202 | -1.977804473 | 0.000111894594369363 | 0.000273565288789121 | 8.05059140748971e-05 | 8099 | tags=80%, list=19%, signal=65% | ACO1/GRHPR/MTHFD1/MTHFD1L/AFMID/MDH1/PGP/MDH2/GLYCTK/MTHFD2/ACO2/CS |
| KEGG_VIBRIO_CHOLERAE_INFECTION | KEGG_VIBRIO_CHOLERAE_INFECTION | KEGG_VIBRIO_CHOLERAE_INFECTION | 54 | -0.495269722 | -1.917308151 | 0.000113250146434206 | 0.000273565288789121 | 8.05059140748971e-05 | 7631 | tags=54%, list=18%, signal=44% | ADCY9/PRKCA/ATP6V0D1/SLC12A2/TCIRG1/TJP2/PRKX/ATP6V1F/ATP6V0E1/KCNQ1/ATP6AP1/ACTB/PDIA4/ERO1A/ATP6V1G1/ADCY3/ATP6V1E2/ATP6V1E1/SEC61G/ACTG1/KDELR1/ATP6V1B2/PRKACA/ATP6V1H/PRKCB/KDELR2/ARF1/SEC61B/SEC61A1 |
| KEGG_GNRH_SIGNALING_PATHWAY | KEGG_GNRH_SIGNALING_PATHWAY | KEGG_GNRH_SIGNALING_PATHWAY | 97 | -0.418152308 | -1.817453665 | 0.0001155041141493 | 0.000275432887586792 | 8.10555187012629e-05 | 14441 | tags=64%, list=35%, signal=42% | GNRHR/PLA2G5/CAMK2B/MAPK12/MAP3K1/MMP14/ITPR3/JUN/ITPR1/SOS1/PLA2G4B/MAPK10/KRAS/MAP2K6/PLA2G12A/PLA2G6/PLCB1/JMJD7-PLA2G4B/GNAQ/ADCY8/PLD1/MAPK1/MAP3K4/MAPK11/MAPK3/PLA2G2C/CACNA1F/CAMK2D/PLA2G4A/ADCY9/PRKCA/MAPK14/ADCY7/MAP2K2/GNA11/CAMK2G/PLCB2/ITPR2/ADCY5/PRKX/MAPK7/CALM2/PRKCD/ATF4/NRAS/MAP3K3/MAP2K7/ADCY3/SRC/CALM1/MAPK9/PRKACA/MAP2K1/PTK2B/HRAS/CALM3/PRKCB/CDC42/ELK1/GRB2/PLCB3/PLD2 |
| KEGG_SELENOAMINO_ACID_METABOLISM | KEGG_SELENOAMINO_ACID_METABOLISM | KEGG_SELENOAMINO_ACID_METABOLISM | 26 | -0.602434004 | -1.951052806 | 0.000180955570608826 | 0.000426047292825844 | 0.000125378942993457 | 7803 | tags=62%, list=19%, signal=50% | PAPSS2/GGT1/MARS2/AHCY/LCMT2/BUD23/SCLY/PAPSS1/AHCYL2/METTL2B/METTL6/SEPHS1/MARS1/SEPHS2/LCMT1/AHCYL1 |
| KEGG_CARDIAC_MUSCLE_CONTRACTION | KEGG_CARDIAC_MUSCLE_CONTRACTION | KEGG_CARDIAC_MUSCLE_CONTRACTION | 76 | -0.442159747 | -1.827671607 | 0.000187744743298629 | 0.000436506528169312 | 0.000128456929625378 | 6678 | tags=36%, list=16%, signal=30% | COX7C/COX6C/TPM4/COX7A2/UQCRC1/COX4I1/CACNA2D4/COX7A2L/COX8A/UQCRQ/COX6A1/ATP2A2/COX5B/SLC9A6/CYC1/UQCR10/MT-CO2/UQCRFS1/MT-CO3/MT-CYB/COX5A/ATP1A1/SLC9A1/UQCRC2/MT-CO1/ATP1B3/TPM3 |
| KEGG_PROSTATE_CANCER | KEGG_PROSTATE_CANCER | KEGG_PROSTATE_CANCER | 87 | -0.427516416 | -1.822973512 | 0.000192362024114942 | 0.0004417202035232 | 0.000129991231370721 | 12133 | tags=53%, list=29%, signal=38% | KRAS/E2F3/CCNE1/PDGFRA/NFKBIA/CREB3L4/EGF/CREB3L2/CDKN1A/MAPK1/HSP90AA1/MAPK3/EP300/RB1/HSP90B1/HSP90AB1/PIK3R5/PDGFA/MAP2K2/PIK3CD/PDPK1/FGFR1/CDK2/CHUK/TCF7L2/ATF4/GSTP1/PIK3CG/NRAS/GSK3B/CREB3/IKBKB/CTNNB1/RELA/TP53/MTOR/PIK3CB/MAP2K1/HRAS/CASP9/IKBKG/AKT1/ARAF/AKT2/GRB2/NFKB1 |
| KEGG_RIBOSOME | KEGG_RIBOSOME | KEGG_RIBOSOME | 88 | -0.420893158 | -1.797459338 | 0.000244088538738785 | 0.000553664246407489 | 0.000162934582983528 | 17147 | tags=82%, list=41%, signal=48% | RPL8/RPS3A/RPL9/RPS4Y1/RPS11/RPS25/RPS29/RPS6/RPL6/RPS20/RPLP2/RPL35A/RPLP1/RPL30/RPL38/RPL32/RPL37/RPS28/RPS27A/RPL24/RPL23A/RPS15A/RPL4/RPS4X/RPL19/RPL34/RPS15/RPL18A/RPS5/RPL22/RPS17/RPL22L1/RPL5/RPL35/RPLP0/RPS27/RPL7A/RPS13/RPS19/RPL36AL/RPL39/RPS3/RPL36/RPS23/RPS7/RPL14/RPS9/RPL37A/RPS8/RPL11/RPL13/RPL27A/RPL17/RPL3/RPL28/RPL18/RPS18/RPS26/FAU/RPL23/RPS2/RPS21/RSL24D1/MRPL13/RPL13A/RPL10A/RPL27/RPS16/RPL15/RPL10/RPSA/RPL26L1 |
| KEGG_ADHERENS_JUNCTION | KEGG_ADHERENS_JUNCTION | KEGG_ADHERENS_JUNCTION | 73 | -0.436028905 | -1.784899384 | 0.000307647829970353 | 0.000689427667162477 | 0.000202887598712217 | 8945 | tags=49%, list=21%, signal=39% | MAPK1/SMAD2/PTPRM/SMAD3/MAPK3/EP300/FYN/WAS/NECTIN2/NECTIN1/FARP2/MAP3K7/VCL/NLK/FGFR1/TCF7L2/RAC2/TGFBR2/ACTB/INSR/CSNK2A2/CSNK2A1/CTNNB1/PTPRJ/PTPN1/SRC/ACTG1/CSNK2B/WASF2/PTPN6/RAC3/RHOA/ACTN4/CTNNA1/RAC1/CDC42 |
| KEGG_CALCIUM_SIGNALING_PATHWAY | KEGG_CALCIUM_SIGNALING_PATHWAY | KEGG_CALCIUM_SIGNALING_PATHWAY | 174 | -0.352808176 | -1.683299282 | 0.000332275512468899 | 0.000735752920466849 | 0.000216520384064947 | 16439 | tags=59%, list=39%, signal=36% | PPP3R2/PLCG1/DRD1/ADCY4/CACNA1C/GNA14/EDNRA/ERBB4/CACNA1D/ADRB1/ATP2B2/ADCY2/CACNA1G/TNNC2/P2RX5/PHKA1/MYLK2/EDNRB/HRH2/PPP3CA/CAMK2B/PRKCG/CACNA1H/ITPR3/ITPR1/GRIN2C/PLCG2/PPP3R1/PLCE1/TACR2/NTSR1/SLC8A1/P2RX6/PDGFRA/GRPR/TBXA2R/PLCB1/NOS2/GNAQ/PHKG1/CHRM3/SLC8A3/ITPKB/ERBB3/ADCY8/ATP2A1/ADORA2B/ADRB2/CACNA1A/PPP3CC/P2RX1/NOS3/SLC25A4/CD38/CACNA1F/PLCD3/CAMK2D/ADCY9/PRKCA/BST1/RYR1/ADCY7/HTR7/SPHK1/GNA15/GNA11/CAMK2G/PLCB2/ITPR2/PRKX/LTB4R2/PLN/PDE1B/ADORA2A/PHKB/CALM2/PLCD1/PPID/ATP2A2/CHP1/ADCY3/P2RX4/VDAC3/CALM1/GRIN2D/PTAFR/SLC25A6/P2RX7/PRKACA/PTK2B/ATP2A3/SLC25A5/SPHK2/CALM3/PRKCB/PHKG2/VDAC2P5/PPP3CB/VDAC1/VDAC2/PHKA2/PLCB3 |
| KEGG_AXON_GUIDANCE | KEGG_AXON_GUIDANCE | KEGG_AXON_GUIDANCE | 128 | -0.374117412 | -1.700253057 | 0.000389397355447967 | 0.000852093036627317 | 0.000250757430133676 | 18667 | tags=74%, list=45%, signal=41% | NGEF/NRP1/EFNB3/PLXNB1/NTN1/ABLIM2/SEMA6B/SEMA3F/PLXNA1/EPHA4/EPHA3/EPHA8/CXCL12/PLXNA3/DCC/PPP3R2/ABLIM1/PAK6/ROBO3/EPHB3/SRGAP3/SEMA4C/UNC5C/ITGB1/EPHA1/EPHB2/UNC5D/SEMA5B/EFNA2/PPP3CA/SLIT3/PAK3/ROCK2/NFATC3/DPYSL5/PPP3R1/SEMA4F/SEMA3D/SEMA5A/SLIT1/PAK1/KRAS/ABLIM3/LIMK2/EPHB4/EPHB6/SEMA6A/ROCK1/SEMA3G/PAK2/CXCR4/RASA1/SEMA3B/SEMA4A/PLXNB3/SEMA4D/UNC5B/GNAI3/MAPK1/NCK1/PPP3CC/MAPK3/NFATC1/SEMA4B/FYN/NCK2/CFL2/CDK5/FES/UNC5A/SEMA4G/EFNA1/CFL1/EFNA4/RAC2/SEMA6C/PLXNB2/NRAS/GSK3B/CHP1/RGS3/SRGAP2/PAK4/ABL1/RAC3/EFNA3/GNAI2/HRAS/RHOA/EFNB1/RAC1/DPYSL2/PPP3CB/CDC42/LIMK1 |
| KEGG_HOMOLOGOUS_RECOMBINATION | KEGG_HOMOLOGOUS_RECOMBINATION | KEGG_HOMOLOGOUS_RECOMBINATION | 28 | -0.572705483 | -1.892919771 | 0.0004032837395947 | 0.00087221832051877 | 0.000256679981137386 | 16415 | tags=93%, list=39%, signal=56% | RAD51C/RAD52/BRCA2/RAD54L/XRCC2/NBN/RAD51/RAD50/RPA4/SEM1/EME1/RPA3/XRCC3/BLM/POLD4/POLD2/RPA2/POLD1/POLD3/RPA1/RAD51D/SSBP1/TOP3B/MUS81/RAD51B/TOP3A |
| KEGG_ARGININE_AND_PROLINE_METABOLISM | KEGG_ARGININE_AND_PROLINE_METABOLISM | KEGG_ARGININE_AND_PROLINE_METABOLISM | 54 | -0.469888767 | -1.819052374 | 0.000584377399883136 | 0.0012493585790605 | 0.000367666361692959 | 11379 | tags=54%, list=27%, signal=39% | PYCR1/AMD1/GLS/NOS2/LAP3/PYCR3/CPS1/NOS3/NAGS/CKB/AGMAT/ODC1/ALDH1B1/GAMT/GOT2/ALDH4A1/GLUD2/ASL/ALDH18A1/SAT2/ALDH2/ACY1/P4HA1/ALDH3A2/SRM/ALDH9A1/PYCR2/SMS/GLUD1 |
| KEGG_VALINE_LEUCINE_AND_ISOLEUCINE_BIOSYNTHESIS | KEGG_VALINE_LEUCINE_AND_ISOLEUCINE_BIOSYNTHESIS | KEGG_VALINE_LEUCINE_AND_ISOLEUCINE_BIOSYNTHESIS | 10 | -0.723393774 | -1.828097123 | 0.000944951851558853 | 0.0019972845953403 | 0.000587769094271057 | 9498 | tags=90%, list=23%, signal=70% | LARS1/IARS1/BCAT2/VARS2/VARS1/LARS2/PDHA1/PDHB/IARS2 |
| KEGG_VASOPRESSIN_REGULATED_WATER_REABSORPTION | KEGG_VASOPRESSIN_REGULATED_WATER_REABSORPTION | KEGG_VASOPRESSIN_REGULATED_WATER_REABSORPTION | 44 | -0.482121233 | -1.792658067 | 0.000967515065922565 | 0.00202199777822019 | 0.000595041790987267 | 9967 | tags=64%, list=24%, signal=48% | AQP4/CREB3L4/CREB3L2/DCTN4/DYNC1LI2/DYNC1I1/ADCY9/STX4/DCTN6/PRKX/DYNLL2/AVPR2/DCTN2/DYNC1H1/DYNLL1/RAB5C/CREB3/DYNC1I2/DYNC1LI1/ADCY3/ARHGDIA/ARHGDIB/PRKACA/NSF/RAB11A/DCTN5/DCTN1/RAB11B |
| KEGG_MELANOGENESIS | KEGG_MELANOGENESIS | KEGG_MELANOGENESIS | 99 | -0.386265596 | -1.688518763 | 0.00112646808317276 | 0.00232803403855704 | 0.000685103395613843 | 17013 | tags=63%, list=41%, signal=37% | WNT5A/PRKACB/POMC/ADCY4/WNT3/WNT1/WNT10A/WNT7A/LEF1/ADCY2/WNT7B/EDNRB/CAMK2B/PRKCG/CREBBP/FZD9/FZD8/MC1R/WNT11/KRAS/WNT16/WNT8B/FZD3/CREB3L4/PLCB1/GNAQ/CREB3L2/ADCY8/GNAI3/MITF/MAPK1/MAPK3/EP300/CAMK2D/ADCY9/PRKCA/FZD2/ADCY7/MAP2K2/CAMK2G/PLCB2/ADCY5/PRKX/CALM2/TCF7L2/DVL1/NRAS/GSK3B/CREB3/DVL2/CTNNB1/ADCY3/CALM1/PRKACA/MAP2K1/GNAI2/HRAS/FZD1/CALM3/PRKCB/DVL3/PLCB3 |
| KEGG_STEROID_BIOSYNTHESIS | KEGG_STEROID_BIOSYNTHESIS | KEGG_STEROID_BIOSYNTHESIS | 17 | -0.630337385 | -1.823363712 | 0.00150279109960299 | 0.00307163895083689 | 0.0009039344960018 | 13677 | tags=88%, list=33%, signal=59% | CYP27B1/DHCR24/FDFT1/DHCR7/SOAT2/LSS/HSD17B7/SC5D/MSMO1/EBP/LIPA/SQLE/NSDHL/CYP51A1/SOAT1 |
| KEGG_CYTOSOLIC_DNA_SENSING_PATHWAY | KEGG_CYTOSOLIC_DNA_SENSING_PATHWAY | KEGG_CYTOSOLIC_DNA_SENSING_PATHWAY | 47 | -0.463586473 | -1.729784621 | 0.0019969062727713 | 0.00403557772760307 | 0.00118760634881358 | 12183 | tags=60%, list=29%, signal=42% | CASP1/TBK1/NFKBIA/CCL4/CCL5/ADAR/IL6/POLR1C/PYCARD/POLR3F/POLR3A/IRF3/CHUK/POLR3D/MAVS/POLR3C/IKBKB/RIPK1/STING1/RELA/POLR3H/POLR3K/RIPK3/NFKBIB/POLR3GL/IKBKG/IKBKE/NFKB1 |
| KEGG_FOCAL_ADHESION | KEGG_FOCAL_ADHESION | KEGG_FOCAL_ADHESION | 197 | -0.320557658 | -1.564671018 | 0.00201778886380154 | 0.00403557772760307 | 0.00118760634881358 | 14935 | tags=55%, list=36%, signal=35% | BAD/ITGA10/ITGA1/MYLK2/KDR/ITGB1/MYL9/HGF/COL6A3/PRKCG/VTN/JUN/COL6A1/PAK3/VWF/ROCK2/COL5A2/SOS1/COL11A1/MAPK10/PAK1/COL4A6/ITGAV/COL5A3/FN1/ITGB4/PDGFRA/COL4A1/ITGB7/LAMA4/ITGB3/ITGA9/EGF/ROCK1/VEGFA/VASP/ITGA2B/PAK2/TNC/ZYX/SPP1/ITGA5/XIAP/LAMB2/MAPK1/THBS4/MAPK3/FYN/PARVB/SHC4/ARHGAP35/SHC2/PRKCA/PIK3R5/CCND3/VEGFB/RAP1B/ITGA11/VAV2/PXN/PDGFA/CCND2/FLNB/VAV3/VCL/CRK/PIK3CD/PDPK1/RAC2/COL11A2/RAPGEF1/ACTB/PPP1CA/PIK3CG/CAPN2/ILK/GSK3B/CTNNB1/CRKL/PAK4/FLNA/SRC/MYL5/MYL12A/ACTG1/MAPK9/PPP1CC/PIK3CB/PIP5K1C/MAP2K1/RAC3/THBS3/HRAS/RHOA/ACTN4/TLN1/MYL12B/RAC1/PRKCB/AKT1/PARVG/DIAPH1/SHC1/CDC42/ELK1/AKT2/VAV1/GRB2 |
| KEGG_GLYCOSAMINOGLYCAN_BIOSYNTHESIS_HEPARAN_SULFATE | KEGG_GLYCOSAMINOGLYCAN_BIOSYNTHESIS_HEPARAN_SULFATE | KEGG_GLYCOSAMINOGLYCAN_BIOSYNTHESIS_HEPARAN_SULFATE | 25 | -0.553955522 | -1.775370665 | 0.00250524694387841 | 0.00495719076129132 | 0.00145882240852942 | 10414 | tags=60%, list=25%, signal=45% | HS3ST3B1/HS3ST5/EXTL1/HS3ST1/B4GALT7/HS2ST1/HS6ST1/NDST1/EXT1/XYLT2/B3GALT6/EXT2/HS3ST3A1/B3GAT3/NDST2 |
| KEGG_GLYCOSAMINOGLYCAN_BIOSYNTHESIS_CHONDROITIN_SULFATE | KEGG_GLYCOSAMINOGLYCAN_BIOSYNTHESIS_CHONDROITIN_SULFATE | KEGG_GLYCOSAMINOGLYCAN_BIOSYNTHESIS_CHONDROITIN_SULFATE | 22 | -0.573278146 | -1.743361823 | 0.00258944845842712 | 0.00501705638820254 | 0.00147643991050669 | 10245 | tags=73%, list=25%, signal=55% | CSGALNACT1/XYLT1/CHST13/CSGALNACT2/CHST11/CHST12/CHPF/CHST14/UST/B4GALT7/DSE/CHST7/XYLT2/B3GALT6/B3GAT3/CHPF2 |
| KEGG_GLIOMA | KEGG_GLIOMA | KEGG_GLIOMA | 65 | -0.425478034 | -1.705423834 | 0.0025791676215352 | 0.00501705638820254 | 0.00147643991050669 | 8945 | tags=60%, list=21%, signal=47% | CAMK2B/PRKCG/PLCG2/SOS1/KRAS/E2F3/PDGFRA/EGF/CDKN1A/MAPK1/CDKN2A/MAPK3/RB1/CAMK2D/SHC4/SHC2/PRKCA/PIK3R5/PDGFA/MAP2K2/CAMK2G/PIK3CD/CALM2/CDK4/PIK3CG/NRAS/CALM1/TP53/MTOR/PIK3CB/MAP2K1/HRAS/CALM3/PRKCB/AKT1/ARAF/SHC1/AKT2/GRB2 |
| KEGG_LIMONENE_AND_PINENE_DEGRADATION | KEGG_LIMONENE_AND_PINENE_DEGRADATION | KEGG_LIMONENE_AND_PINENE_DEGRADATION | 10 | -0.692121441 | -1.749068432 | 0.00295092497309493 | 0.00565847469067688 | 0.00166519911666778 | 6863 | tags=70%, list=16%, signal=59% | ALDH1B1/HADHA/NAA80/ALDH2/ALDH3A2/ECHS1/ALDH9A1 |
| KEGG_GLYCOSYLPHOSPHATIDYLINOSITOL_GPI_ANCHOR_BIOSYNTHESIS | KEGG_GLYCOSYLPHOSPHATIDYLINOSITOL_GPI_ANCHOR_BIOSYNTHESIS | KEGG_GLYCOSYLPHOSPHATIDYLINOSITOL_GPI_ANCHOR_BIOSYNTHESIS | 25 | -0.54971997 | -1.761796157 | 0.00305418590498964 | 0.00579672018702115 | 0.0017058825677708 | 7482 | tags=72%, list=18%, signal=59% | PIGB/PIGL/PIGM/PIGW/PIGN/PIGY/GPAA1/PIGP/PIGZ/PIGG/PIGU/PIGV/PIGH/PIGC/PIGT/PIGF/PIGS/PIGO |
| KEGG_ENDOMETRIAL_CANCER | KEGG_ENDOMETRIAL_CANCER | KEGG_ENDOMETRIAL_CANCER | 52 | -0.441364636 | -1.69118286 | 0.00313718375498148 | 0.00589410281238945 | 0.00173454072577392 | 8945 | tags=50%, list=21%, signal=39% | MAPK1/MAPK3/MYC/PIK3R5/MAP2K2/PIK3CD/APC2/PDPK1/TCF7L2/PIK3CG/ILK/NRAS/GSK3B/CTNNB1/TP53/MLH1/PIK3CB/MAP2K1/HRAS/CASP9/CTNNA1/AKT1/ARAF/ELK1/AKT2/GRB2 |
| KEGG_NOD_LIKE_RECEPTOR_SIGNALING_PATHWAY | KEGG_NOD_LIKE_RECEPTOR_SIGNALING_PATHWAY | KEGG_NOD_LIKE_RECEPTOR_SIGNALING_PATHWAY | 61 | -0.424178213 | -1.677417133 | 0.00358137588885975 | 0.00666135915327914 | 0.00196033206548113 | 12445 | tags=57%, list=30%, signal=40% | MAPK10/RIPK2/CCL2/CASP1/NLRP3/NAIP/NFKBIA/CCL5/XIAP/TNFAIP3/MAPK1/TNF/HSP90AA1/MAPK11/IL6/MAPK3/HSP90B1/HSP90AB1/MAPK14/CASP8/MAP3K7/PYCARD/TRAF6/PSTPIP1/CHUK/NOD1/CARD9/IKBKB/RELA/TRIP6/MAPK9/TAB1/NFKBIB/IKBKG/NFKB1 |
| KEGG_DRUG_METABOLISM_OTHER_ENZYMES | KEGG_DRUG_METABOLISM_OTHER_ENZYMES | KEGG_DRUG_METABOLISM_OTHER_ENZYMES | 44 | -0.448359486 | -1.667122695 | 0.00401619204124986 | 0.00739615564032152 | 0.00217657098639909 | 6784 | tags=36%, list=16%, signal=30% | GMPS/UCK2/UPP1/IMPDH2/UMPS/IMPDH1/HPRT1/GUSB/DPYD/UCKL1/ITPA/NAT1/UCK1/TK2/CES2/TPMT |
| KEGG_CYSTEINE_AND_METHIONINE_METABOLISM | KEGG_CYSTEINE_AND_METHIONINE_METABOLISM | KEGG_CYSTEINE_AND_METHIONINE_METABOLISM | 34 | -0.487596086 | -1.692286409 | 0.00425218956778154 | 0.0077539927412487 | 0.00228187675464025 | 15765 | tags=74%, list=38%, signal=46% | LDHAL6A/DNMT3L/MPST/GOT1/MTR/SDS/AMD1/MAT2A/MTAP/TRDMT1/DNMT3A/CDO1/IL4I1/LDHB/GOT2/LDHA/DNMT1/AHCY/AHCYL2/ENOPH1/SRM/APIP/ADI1/SMS/AHCYL1 |
| KEGG_NON_SMALL_CELL_LUNG_CANCER | KEGG_NON_SMALL_CELL_LUNG_CANCER | KEGG_NON_SMALL_CELL_LUNG_CANCER | 54 | -0.429609034 | -1.663119845 | 0.00436505423706937 | 0.00788252512713498 | 0.00231970179179977 | 8945 | tags=63%, list=21%, signal=50% | RARB/PRKCG/PLCG2/SOS1/RXRA/KRAS/E2F3/EGF/MAPK1/CDKN2A/STK4/MAPK3/RB1/PRKCA/PIK3R5/MAP2K2/RASSF1/PIK3CD/PDPK1/CDK4/PIK3CG/NRAS/TP53/PIK3CB/MAP2K1/RASSF5/RXRB/HRAS/CASP9/PRKCB/AKT1/ARAF/AKT2/GRB2 |
| KEGG_ALANINE_ASPARTATE_AND_GLUTAMATE_METABOLISM | KEGG_ALANINE_ASPARTATE_AND_GLUTAMATE_METABOLISM | KEGG_ALANINE_ASPARTATE_AND_GLUTAMATE_METABOLISM | 31 | -0.508821515 | -1.71801124 | 0.00582403999660688 | 0.0104160715323931 | 0.00306528420874047 | 13663 | tags=68%, list=33%, signal=46% | GOT1/GPT/DDO/ACY3/GLS/CPS1/ABAT/PPAT/GFPT1/NIT2/IL4I1/ADSS2/GOT2/ALDH4A1/GPT2/GLUD2/CAD/ASL/ADSS1/ADSL/GLUD1 |
| KEGG_OLFACTORY_TRANSDUCTION | KEGG_OLFACTORY_TRANSDUCTION | KEGG_OLFACTORY_TRANSDUCTION | 178 | 0.293860664915342 | 1.49015313794557 | 0.00609117059249158 | 0.0107900736209851 | 0.00317534707578508 | 3206 | tags=11%, list=8%, signal=10% | OR52A1/OR5AU1/OR1J4/OR2W3/OR13A1/OR2T33/OR52N4/OR2T8/OR2T10/OR52A5/PDC/OR52K2/OR1B1/OR52K1/OR13C5/OR4F17/OR2L2/OR10Z1/OR4N2 |
| KEGG_INTESTINAL_IMMUNE_NETWORK_FOR_IGA_PRODUCTION | KEGG_INTESTINAL_IMMUNE_NETWORK_FOR_IGA_PRODUCTION | KEGG_INTESTINAL_IMMUNE_NETWORK_FOR_IGA_PRODUCTION | 46 | -0.445975695 | -1.657700449 | 0.00626776343768384 | 0.0109981509378226 | 0.00323658092114756 | 11779 | tags=57%, list=28%, signal=41% | ICOS/ITGB7/CD28/CD40/ICOSLG/HLA-DQA1/CXCR4/HLA-DQB1/IL15/HLA-DQA2/IL10/HLA-DRB5/IL6/CCR9/HLA-DRB1/TNFSF13/HLA-DPB1/HLA-DPA1/HLA-DRA/HLA-DMB/HLA-DMA/TGFB1/MAP3K14/CD86/IL15RA/LTBR |
| KEGG_GLUTATHIONE_METABOLISM | KEGG_GLUTATHIONE_METABOLISM | KEGG_GLUTATHIONE_METABOLISM | 45 | -0.45219024 | -1.679777203 | 0.00653240922910221 | 0.0112560982814518 | 0.00331249072232878 | 13397 | tags=64%, list=32%, signal=44% | ANPEP/RRM1/GSTM2/PGD/GSTM5/LAP3/G6PD/GSTM1/GSTA4/GGT1/MGST3/GSTM4/ODC1/GPX4/GSTO1/GGCT/MGST1/IDH1/MGST2/GSTZ1/GSTP1/GPX7/GSS/IDH2/GSR/SRM/GSTK1/TXNDC12/SMS |
| KEGG_PRIMARY_IMMUNODEFICIENCY | KEGG_PRIMARY_IMMUNODEFICIENCY | KEGG_PRIMARY_IMMUNODEFICIENCY | 35 | -0.468480751 | -1.639712315 | 0.00655173588362618 | 0.0112560982814518 | 0.00331249072232878 | 11779 | tags=57%, list=28%, signal=41% | ICOS/LCK/CD8B/CD40/RAG1/RFXANK/CD3D/RFXAP/TAP2/JAK3/TAP1/UNG/CD4/CIITA/DCLRE1C/IL2RG/ADA/IKBKG/RFX5/BTK |
| KEGG_TGF_BETA_SIGNALING_PATHWAY | KEGG_TGF_BETA_SIGNALING_PATHWAY | KEGG_TGF_BETA_SIGNALING_PATHWAY | 85 | -0.370309677 | -1.574508193 | 0.00659631565956048 | 0.0112560982814518 | 0.00331249072232878 | 17647 | tags=64%, list=42%, signal=37% | LEFTY1/DCN/BMPR2/INHBC/ID1/ID3/IFNG/SP1/SMAD4/CHRD/BMP8A/THBS2/E2F5/AMHR2/ACVRL1/CREBBP/RBL2/ROCK2/TGFBR1/ID4/SMAD6/SMURF2/ROCK1/RBX1/BMP2/BMP7/RBL1/MAPK1/TNF/SMAD1/SMAD2/BMPR1B/SMAD3/THBS4/MAPK3/MYC/EP300/BMP8B/PPP2R1B/SMURF1/ID2/SKP1/SMAD7/TGFB1/TGFBR2/E2F4/RPS6KB2/ACVR1/PPP2CA/THBS3/RHOA/CUL1/PPP2R1A/PPP2CB |
| KEGG_ALLOGRAFT_REJECTION | KEGG_ALLOGRAFT_REJECTION | KEGG_ALLOGRAFT_REJECTION | 35 | -0.463712193 | -1.623022061 | 0.00776295488829674 | 0.0131264509929381 | 0.00386290578173617 | 16949 | tags=74%, list=41%, signal=44% | IL12A/HLA-C/HLA-DOA/IFNG/PRF1/FASLG/GZMB/CD28/CD40/HLA-DQA1/HLA-DQB1/HLA-DQA2/TNF/IL10/HLA-DRB5/HLA-DRB1/HLA-A/HLA-B/HLA-E/HLA-DPB1/HLA-F/HLA-DPA1/HLA-DRA/HLA-DMB/HLA-DMA/CD86 |
| KEGG_ONE_CARBON_POOL_BY_FOLATE | KEGG_ONE_CARBON_POOL_BY_FOLATE | KEGG_ONE_CARBON_POOL_BY_FOLATE | 17 | -0.578845359 | -1.674413809 | 0.00811309838709037 | 0.0134735383928465 | 0.00396504808391635 | 13078 | tags=82%, list=31%, signal=57% | MTR/MTHFS/ALDH1L1/MTHFD2L/SHMT1/DHFR/MTHFD1/MTHFD1L/ATIC/GART/SHMT2/MTFMT/MTHFR/MTHFD2 |
| KEGG_GLYCEROLIPID_METABOLISM | KEGG_GLYCEROLIPID_METABOLISM | KEGG_GLYCEROLIPID_METABOLISM | 45 | -0.444311087 | -1.650508058 | 0.00806941396317819 | 0.0134735383928465 | 0.00396504808391635 | 7205 | tags=47%, list=17%, signal=39% | AGPAT2/LPL/MBOAT1/MGLL/ALDH1B1/DGAT1/PLPP1/AKR1B1/DGKD/DGKZ/LIPC/ALDH2/DGKG/AGPAT1/ALDH3A2/AKR1A1/AGPAT3/TKFC/GLYCTK/ALDH9A1/GPAT4 |
| KEGG_GLYCOSPHINGOLIPID_BIOSYNTHESIS_GANGLIO_SERIES | KEGG_GLYCOSPHINGOLIPID_BIOSYNTHESIS_GANGLIO_SERIES | KEGG_GLYCOSPHINGOLIPID_BIOSYNTHESIS_GANGLIO_SERIES | 15 | -0.599281969 | -1.670202472 | 0.0108669782477362 | 0.0178872385316721 | 0.00526392984520061 | 9890 | tags=67%, list=24%, signal=51% | ST6GALNAC3/ST3GAL2/B3GALT4/ST8SIA5/SLC33A1/ST6GALNAC6/ST3GAL5/GLB1/HEXA/HEXB |
| KEGG_GRAFT_VERSUS_HOST_DISEASE | KEGG_GRAFT_VERSUS_HOST_DISEASE | KEGG_GRAFT_VERSUS_HOST_DISEASE | 37 | -0.454821721 | -1.618943551 | 0.0110618584215721 | 0.0180482953194071 | 0.00531132629659971 | 16866 | tags=70%, list=40%, signal=42% | HLA-C/HLA-DOA/IFNG/IL1B/PRF1/FASLG/GZMB/KLRC1/CD28/HLA-DQA1/HLA-DQB1/HLA-DQA2/TNF/HLA-DRB5/IL6/HLA-DRB1/HLA-A/HLA-B/HLA-E/HLA-DPB1/HLA-F/HLA-DPA1/HLA-DRA/HLA-DMB/HLA-DMA/CD86 |
| KEGG_LONG_TERM_DEPRESSION | KEGG_LONG_TERM_DEPRESSION | KEGG_LONG_TERM_DEPRESSION | 67 | -0.39151425 | -1.578047061 | 0.0114315331727262 | 0.0183606566481446 | 0.00540324926827119 | 15741 | tags=63%, list=38%, signal=39% | GUCY1B1/PPP1R17/GRIA2/GUCY1A2/GNA13/PLA2G5/PRKCG/ITPR3/ITPR1/GRIA1/LYN/PLA2G4B/KRAS/PRKG1/PLA2G12A/PLA2G6/PLCB1/JMJD7-PLA2G4B/GNAQ/GNAI3/MAPK1/CACNA1A/MAPK3/PLA2G2C/PLA2G4A/PRKCA/RYR1/MAP2K2/GNA11/PPP2R1B/PLCB2/ITPR2/NRAS/PPP2CA/MAP2K1/GNAI2/HRAS/PRKCB/PPP2R1A/ARAF/PPP2CB/PLCB3 |
| KEGG_CELL_ADHESION_MOLECULES_CAMS | KEGG_CELL_ADHESION_MOLECULES_CAMS | KEGG_CELL_ADHESION_MOLECULES_CAMS | 130 | -0.324060271 | -1.477416323 | 0.011450732103144 | 0.0183606566481446 | 0.00540324926827119 | 17753 | tags=65%, list=43%, signal=38% | CD8A/ITGB8/CLDN2/CD22/CD2/ITGA6/ITGA4/HLA-C/HLA-DOA/CADM3/CNTN2/PTPRC/CLDN19/NCAM1/SELL/PDCD1/SELP/NLGN4X/CD58/CD6/NLGN3/CLDN20/MAG/CLDN1/ITGB1/CLDN23/CLDN10/ESAM/CDH5/NRCAM/ALCAM/NRXN2/ICAM1/ITGAV/NCAM2/ICOS/ITGB7/CD28/SDC3/CLDN11/ITGA9/CD276/OCLN/CD8B/CD40/ICOSLG/HLA-DQA1/HLA-DQB1/NLGN2/NFASC/HLA-DQA2/PTPRM/HLA-DRB5/VCAM1/JAM2/SELPLG/ICAM3/VCAN/SPN/NECTIN2/NECTIN1/HLA-DRB1/HLA-A/CLDN7/HLA-B/ITGAM/JAM3/F11R/PECAM1/CD99/HLA-E/HLA-DPB1/ICAM2/HLA-F/ITGAL/CD4/HLA-DPA1/HLA-DRA/HLA-DMB/HLA-DMA/MPZ/GLG1/PVR/CD86/ITGB2 |
| KEGG_PANCREATIC_CANCER | KEGG_PANCREATIC_CANCER | KEGG_PANCREATIC_CANCER | 70 | -0.372431493 | -1.511246622 | 0.0127067643242009 | 0.0202004971307808 | 0.00594468506395361 | 12445 | tags=64%, list=30%, signal=45% | MAPK10/RALB/TGFBR1/RAD51/KRAS/E2F3/EGF/VEGFA/STAT1/PLD1/MAPK1/SMAD2/CDKN2A/SMAD3/MAPK3/RB1/PIK3R5/RALGDS/VEGFB/PIK3CD/TGFB1/CHUK/CDK4/RAC2/JAK1/TGFBR2/ARHGEF6/PIK3CG/IKBKB/RELA/TP53/STAT3/MAPK9/PIK3CB/MAP2K1/RAC3/CASP9/IKBKG/RAC1/RALA/AKT1/ARAF/CDC42/AKT2/NFKB1 |
| KEGG_PRION_DISEASES | KEGG_PRION_DISEASES | KEGG_PRION_DISEASES | 34 | -0.459967782 | -1.59639761 | 0.0129660504407018 | 0.0204380117116148 | 0.00601458182797944 | 13702 | tags=59%, list=33%, signal=40% | C1QC/C1QB/EGR1/C8G/NCAM2/C1QA/CCL5/MAPK1/IL6/MAPK3/FYN/BAX/MAP2K2/PRKX/PRNP/HSPA5/STIP1/PRKACA/MAP2K1/ELK1 |
| KEGG_BETA_ALANINE_METABOLISM | KEGG_BETA_ALANINE_METABOLISM | KEGG_BETA_ALANINE_METABOLISM | 22 | -0.523182827 | -1.591019949 | 0.0156809439715183 | 0.0245097107453983 | 0.00721281810277709 | 10885 | tags=68%, list=26%, signal=50% | DPYS/CNDP1/HIBCH/ACADM/ABAT/ALDH1B1/HADHA/ALDH2/MLYCD/DPYD/ALDH3A2/ECHS1/SRM/ALDH9A1/SMS |
| KEGG_PPAR_SIGNALING_PATHWAY | KEGG_PPAR_SIGNALING_PATHWAY | KEGG_PPAR_SIGNALING_PATHWAY | 67 | -0.382577552 | -1.542026584 | 0.0167587088373865 | 0.0259759986979491 | 0.00764432332933421 | 15079 | tags=57%, list=36%, signal=36% | ACADL/FABP7/CD36/PPARG/ME1/FABP5/RXRA/APOA2/OLR1/ACSL4/CPT1C/SLC27A2/ACOX2/ACADM/PPARA/SLC27A5/LPL/CPT1B/PPARD/SCD/SCD5/FABP3/NR1H3/PDPK1/SCP2/ACSL3/UBC/PCK2/ILK/DBI/CPT1A/SLC27A1/CPT2/RXRB/ACAA1/ACOX3/SLC27A4/ACSL5 |
| KEGG_ADIPOCYTOKINE_SIGNALING_PATHWAY | KEGG_ADIPOCYTOKINE_SIGNALING_PATHWAY | KEGG_ADIPOCYTOKINE_SIGNALING_PATHWAY | 67 | -0.376655311 | -1.518156251 | 0.0205321428528736 | 0.0315618063688801 | 0.00928813769768966 | 8943 | tags=49%, list=21%, signal=39% | TNF/PRKAG2/PRKAB2/TNFRSF1A/PPARA/SOCS3/PRKAA1/CPT1B/NFKBIE/PRKAB1/PTPN11/ACSL3/PCK2/CHUK/TRADD/CAMKK2/IKBKB/TNFRSF1B/RELA/ADIPOR2/STAT3/MAPK9/CPT1A/MTOR/TRAF2/RXRB/NFKBIB/PRKAG1/IKBKG/AKT1/ACSL5/AKT2/NFKB1 |
| KEGG_MTOR_SIGNALING_PATHWAY | KEGG_MTOR_SIGNALING_PATHWAY | KEGG_MTOR_SIGNALING_PATHWAY | 51 | -0.403567218 | -1.538707078 | 0.0226779947842331 | 0.0342935530883525 | 0.0100920473151915 | 8945 | tags=51%, list=21%, signal=40% | MAPK1/RPTOR/RPS6KA1/MAPK3/RPS6KA2/PIK3R5/CAB39L/PRKAA1/VEGFB/EIF4EBP1/PIK3CD/PDPK1/EIF4B/ULK3/PIK3CG/RPS6KB2/ULK2/MLST8/MTOR/PIK3CB/STRADA/TSC2/AKT1/RHEB/AKT2/EIF4E2 |
| KEGG_JAK_STAT_SIGNALING_PATHWAY | KEGG_JAK_STAT_SIGNALING_PATHWAY | KEGG_JAK_STAT_SIGNALING_PATHWAY | 143 | -0.292391803 | -1.360174675 | 0.0226167447810368 | 0.0342935530883525 | 0.0100920473151915 | 12293 | tags=43%, list=29%, signal=30% | SOCS4/JAK2/SPRY2/IFNLR1/IL6R/IL2RB/CRLF2/SPRED1/SOCS1/STAM2/SOCS2/IL15/CLCF1/STAT1/CSF2RA/IL10/IL6/MYC/EP300/IL23A/JAK3/IFNAR1/SOCS3/PIK3R5/CCND3/IL12RB2/STAT2/CBL/SPRED2/IL10RB/CCND2/SOCS5/CNTF/IRF9/IL21R/PIK3CD/SOCS7/PTPN11/IFNAR2/IL12RB1/IL4R/CISH/JAK1/STAT5B/PIK3CG/STAT5A/IL3RA/IL2RG/IL15RA/STAT3/IFNGR2/IL10RA/STAT6/PIK3CB/PTPN6/PIAS3/AKT1/TYK2/PIAS4/AKT2/GRB2 |
| KEGG_RENAL_CELL_CARCINOMA | KEGG_RENAL_CELL_CARCINOMA | KEGG_RENAL_CELL_CARCINOMA | 70 | -0.354627809 | -1.439003118 | 0.0242065879231524 | 0.0363098818847286 | 0.0106854208149739 | 8945 | tags=47%, list=21%, signal=37% | MAPK1/ELOB/MAPK3/EP300/EGLN2/PIK3R5/CUL2/VEGFB/RAP1B/MAP2K2/CRK/PIK3CD/ARNT/PTPN11/EPAS1/TGFB1/RAPGEF1/PIK3CG/NRAS/CRKL/PAK4/FH/PIK3CB/MAP2K1/VHL/ELOC/HRAS/RAC1/AKT1/ARAF/CDC42/AKT2/GRB2 |
| KEGG_VASCULAR_SMOOTH_MUSCLE_CONTRACTION | KEGG_VASCULAR_SMOOTH_MUSCLE_CONTRACTION | KEGG_VASCULAR_SMOOTH_MUSCLE_CONTRACTION | 111 | -0.322938228 | -1.432795624 | 0.0258408806781765 | 0.0384512304491267 | 0.0113155856443383 | 16354 | tags=60%, list=39%, signal=37% | PPP1R12A/ADCY4/CACNA1C/KCNMB3/EDNRA/GUCY1B1/KCNMA1/PRKCH/CACNA1D/ADCY2/MYLK2/GUCY1A2/RAMP2/MYL9/GNA13/PLA2G5/PRKCG/ITPR3/ITPR1/ROCK2/PLA2G4B/PRKCQ/PRKG1/MYL6/PLA2G12A/ROCK1/PLA2G6/PLCB1/JMJD7-PLA2G4B/GNAQ/ADCY8/ADORA2B/MAPK1/MAPK3/PLA2G2C/CACNA1F/PLA2G4A/ADCY9/PRKCE/KCNMB4/PRKCA/PTGIR/ADCY7/MAP2K2/GNA11/PLCB2/ITPR2/ADCY5/PRKX/CALCRL/KCNMB1/ADORA2A/CALM2/PRKCD/ARHGEF11/ARHGEF1/PPP1CA/ADCY3/CALM1/PPP1CC/PRKACA/MAP2K1/RHOA/CALM3/PRKCB/ARAF/PLCB3 |
| KEGG_GAP_JUNCTION | KEGG_GAP_JUNCTION | KEGG_GAP_JUNCTION | 90 | -0.333669556 | -1.431107818 | 0.0277861447023467 | 0.0410176421796547 | 0.0120708398038599 | 17626 | tags=66%, list=42%, signal=38% | PDGFC/LPAR1/ADCY1/PDGFB/PRKACB/DRD1/ADCY4/TUBB4B/GUCY1B1/ADRB1/ADCY2/TUBB1/GUCY1A2/DRD2/TUBA8/PRKCG/ITPR3/ITPR1/SOS1/KRAS/PRKG1/PDGFRA/EGF/TUBB6/PLCB1/GNAQ/ADCY8/GNAI3/MAPK1/MAPK3/TUBB4A/ADCY9/PRKCA/PDGFA/ADCY7/MAP2K2/GNA11/PLCB2/ITPR2/TUBA3D/ADCY5/PRKX/MAPK7/TUBB/CSNK1D/NRAS/ADCY3/TUBA1B/SRC/TUBA1A/MAP2K5/PRKACA/MAP2K1/GNAI2/HRAS/PRKCB/TUBA1C/GRB2/PLCB3 |
| KEGG_PANTOTHENATE_AND_COA_BIOSYNTHESIS | KEGG_PANTOTHENATE_AND_COA_BIOSYNTHESIS | KEGG_PANTOTHENATE_AND_COA_BIOSYNTHESIS | 16 | -0.543027476 | -1.551603873 | 0.029208929220238 | 0.0427784317713722 | 0.0125890121794644 | 3950 | tags=69%, list=9%, signal=62% | VNN1/UPB1/PANK3/DPYS/BCAT2/PANK2/PANK1/DPYD/PANK4/COASY/PPCS |

**Table S8**

|  | ID | Description | setSize | enrichmentScore | NES | pvalue | p.adjust | qvalue | rank | leading_edge | core_enrichment |
| --- | --- | --- | --- | --- | --- | --- | --- | --- | --- | --- | --- |
| KEGG_SYSTEMIC_LUPUS_ERYTHEMATOSUS | KEGG_SYSTEMIC_LUPUS_ERYTHEMATOSUS | KEGG_SYSTEMIC_LUPUS_ERYTHEMATOSUS | 127 | 0.591859375669086 | 2.15629070996706 | 1e-10 | 9.3e-09 | 6.94736842105263e-09 | 10083 | tags=59%, list=24%, signal=45% | H4C4/H2AC21/H2BC12/H3C13/H2AC13/H2BC6/H2BC5/H2BC7/H4C9/H2AC16/H2BC4/H2AC18/H2AC19/H2BC21/H2BC13/H3C2/C1R/H2BC9/H4C5/H3C7/H2BC15/H4C8/H2AB2/H2BC10/FCGR2A/H2BC14/H2AC17/H2BC17/H2AC8/H2BC8/H2AC20/H2AB3/H2BC11/H2AC6/H2AC4/H2BC18/H2AC15/TRIM21/H3-3A/H2BW1/FCGR2B/CD80/C3/H2AJ/H4C1/HLA-DQA2/H4C14/C1S/H4C12/H4C3/C5/H4C11/H2AC12/ACTN2/H2AC7/CD40/H3-3B/H4C15/H2AC14/H3C1/ACTN3/HLA-DMA/H2AZ1/H3C10/H3C4/C7/H3C3/H3C11/HLA-DOA/HLA-DMB/FCGR3B/IL10/H4C2/H3-5/H2AC11 |
| KEGG_OXIDATIVE_PHOSPHORYLATION | KEGG_OXIDATIVE_PHOSPHORYLATION | KEGG_OXIDATIVE_PHOSPHORYLATION | 126 | 0.567745255517467 | 2.06891240770128 | 1e-10 | 9.3e-09 | 6.94736842105263e-09 | 12754 | tags=70%, list=31%, signal=49% | UQCRHL/COX7A1/NDUFB6/NDUFB3/ATP6V0A1/NDUFV2/COX6B2/ATP6V0E1/COX6A2/COX7B/TCIRG1/ATP6V1E1/ATP5F1D/COX5A/COX4I2/NDUFB4/UQCRH/SDHB/ATP5PO/NDUFA6/SDHD/NDUFB10/ATP5PB/ATP5PF/MT-ND1/NDUFA8/NDUFAB1/NDUFA1/NDUFS5/UQCR10/UQCRC2/COX4I1/NDUFS4/UQCR11/COX6A1/ATP6V1F/COX5B/NDUFA9/ATP5F1C/ATP6V1C1/ATP6AP1/ATP5PD/ATP5F1E/NDUFA2/ATP6V0B/COX6B1/ATP6V1B1/ATP6V1H/NDUFB5/COX7A2/COX7C/COX17/ATP5MC1/SDHA/NDUFS2/CYC1/ATP5MG/COX6C/MT-ND2/ATP6V1A/NDUFS7/NDUFB8/ATP6V1D/ATP6V1G1/ATP6V0C/ATP6V1G2/PPA1/NDUFB9/COX8A/MT-ATP6/UQCRQ/ATP5ME/NDUFS6/ATP5MF/NDUFB7/UQCRFS1/NDUFB1/ATP5F1A/NDUFA4/MT-CO3/NDUFA7/ATP5F1B/ATP5MC3/NDUFC2/ATP6V0D1/ATP5MC2/NDUFA11/MT-ND3 |
| KEGG_PARKINSONS_DISEASE | KEGG_PARKINSONS_DISEASE | KEGG_PARKINSONS_DISEASE | 125 | 0.544702109878426 | 1.98407883911423 | 4.43275472055838e-10 | 2.7483079267462e-08 | 2.05306534425862e-08 | 13283 | tags=70%, list=32%, signal=48% | UBE2J1/UQCRHL/COX7A1/NDUFB6/NDUFB3/HTRA2/NDUFV2/COX6B2/COX6A2/UBE2L6/COX7B/SLC6A3/ATP5F1D/UBA1/COX5A/COX4I2/NDUFB4/UQCRH/SDHB/TH/ATP5PO/NDUFA6/SDHD/NDUFB10/ATP5PB/ATP5PF/MT-ND1/NDUFA8/NDUFAB1/NDUFA1/NDUFS5/UQCR10/LRRK2/UQCRC2/COX4I1/NDUFS4/PPID/UQCR11/COX6A1/SLC25A4/COX5B/NDUFA9/UBE2L3/ATP5F1C/PARK7/ATP5PD/ATP5F1E/NDUFA2/COX6B1/NDUFB5/VDAC2P5/COX7A2/COX7C/ATP5MC1/SDHA/NDUFS2/CYC1/UBE2J2/COX6C/MT-ND2/NDUFS7/NDUFB8/NDUFB9/COX8A/SLC25A5/SLC25A6/MT-ATP6/UQCRQ/NDUFS6/NDUFB7/VDAC3/VDAC1/UQCRFS1/NDUFB1/ATP5F1A/NDUFA4/MT-CO3/NDUFA7/ATP5F1B/ATP5MC3/NDUFC2/ATP5MC2/VDAC2/MT-ND3/UCHL1/CYCS/NDUFS8 |
| KEGG_ALZHEIMERS_DISEASE | KEGG_ALZHEIMERS_DISEASE | KEGG_ALZHEIMERS_DISEASE | 161 | 0.507831410280738 | 1.88943523658728 | 5.05983720198186e-09 | 2.35282429892157e-07 | 1.75762765963581e-07 | 14051 | tags=61%, list=34%, signal=41% | ATP2A1/CDK5R1/UQCRHL/ATF6/MAPK3/COX7A1/NDUFB6/LPL/NDUFB3/NDUFV2/COX6B2/CALML3/GRIN1/COX6A2/COX7B/CACNA1S/MME/ATP5F1D/NOS1/COX5A/COX4I2/NDUFB4/UQCRH/SDHB/ATP5PO/NDUFA6/SDHD/FAS/NDUFB10/ATP5PB/ATP5PF/CACNA1D/NDUFA8/NDUFAB1/NDUFA1/NDUFS5/ADAM17/UQCR10/CALML6/UQCRC2/PSENEN/COX4I1/NDUFS4/UQCR11/CALM3/COX6A1/MAPT/BID/COX5B/NDUFA9/ATP5F1C/BAD/CASP7/ATP5PD/ATP5F1E/NDUFA2/COX6B1/NDUFB5/COX7A2/COX7C/ATP5MC1/SDHA/NDUFS2/CYC1/COX6C/GSK3B/PPP3R1/NDUFS7/NDUFB8/FADD/NDUFB9/COX8A/PLCB3/MT-ATP6/UQCRQ/NDUFS6/NDUFB7/HSD17B10/PPP3R2/ITPR2/UQCRFS1/NDUFB1/ATP5F1A/PSEN1/NDUFA4/MT-CO3/NDUFA7/ATP5F1B/ATP5MC3/NDUFC2/ATP5MC2/GRIN2A/CYCS/NDUFS8/CALM2/MT-CO2/APBB1/GRIN2D/NDUFV1 |
| KEGG_HUNTINGTONS_DISEASE | KEGG_HUNTINGTONS_DISEASE | KEGG_HUNTINGTONS_DISEASE | 176 | 0.490114952053302 | 1.82901299595445 | 1.98990425982864e-08 | 7.40244384656253e-07 | 5.52983920626063e-07 | 15772 | tags=66%, list=38%, signal=42% | CREB5/UQCRHL/COX7A1/NDUFB6/NDUFB3/NDUFV2/COX6B2/BBC3/GRIN1/COX6A2/COX7B/CREB3L1/ATP5F1D/COX5A/COX4I2/NDUFB4/UQCRH/SDHB/ATP5PO/SOD2/NDUFA6/SDHD/NDUFB10/ATP5PB/POLR2J/ATP5PF/NDUFA8/POLR2E/IFT57/NDUFAB1/NDUFA1/NDUFS5/UQCR10/UQCRC2/COX4I1/NDUFS4/SOD1/PPID/UQCR11/COX6A1/SLC25A4/CREBBP/COX5B/NDUFA9/ATP5F1C/SP1/NRF1/ATP5PD/ATP5F1E/NDUFA2/COX6B1/NDUFB5/VDAC2P5/COX7A2/COX7C/ATP5MC1/SDHA/NDUFS2/CYC1/COX6C/NDUFS7/PPARG/CLTA/NDUFB8/POLR2I/CLTB/NDUFB9/COX8A/SLC25A5/AP2S1/PLCB3/SLC25A6/MT-ATP6/UQCRQ/NDUFS6/NDUFB7/VDAC3/VDAC1/POLR2G/POLR2H/UQCRFS1/NDUFB1/ATP5F1A/NDUFA4/MT-CO3/NDUFA7/ATP5F1B/GRM5/ATP5MC3/BAX/NDUFC2/ATP5MC2/VDAC2/DNAH2/POLR2D/TAF4B/CYCS/CREB3L2/NDUFS8/POLR2A/CREB3/PPARGC1A/MT-CO2/POLR2F/NDUFV1/POLR2L/AP2M1/AP2A1/NDUFS3/POLR2K/UQCRC1/MT-ATP8/MT-CYB/CASP9/NDUFA3/POLR2C/CASP8 |
| KEGG_SPLICEOSOME | KEGG_SPLICEOSOME | KEGG_SPLICEOSOME | 126 | 0.480226224455832 | 1.74998555183813 | 5.3309305534539e-06 | 0.000165258847157071 | 0.000123453128606301 | 14063 | tags=55%, list=34%, signal=36% | HSPA6/BUD31/SYF2/PCBP1/HNRNPC/ISY1/SF3B6/SART1/RBM22/LSM3/SF3B4/ZMAT2/MAGOH/SF3B2/ALYREF/SNRPB2/PQBP1/PRPF31/SNRNP27/EIF4A3/SF3A1/USP39/SMNDC1/SF3B5/PPIL1/XAB2/SNRPD2/SNW1/CCDC12/RBM8A/TXNL4A/LSM6/SNRNP70/PRPF6/PRPF18/CHERP/SRSF3/SF3A2/SRSF4/SNRPC/HNRNPA1L2/PUF60/SNRPA/HSPA2/PHF5A/CWC15/SNRPG/SNRPE/HNRNPK/SNU13/HSPA1L/U2AF2/HSPA1B/BCAS2/LSM5/HSPA8/CRNKL1/LSM7/PRPF4/SF3B3/SNRPB/SF3A3/HSPA1A/SNRPD1/THOC3/ACIN1/DHX38/SRSF9/PLRG1 |
| KEGG_B_CELL_RECEPTOR_SIGNALING_PATHWAY | KEGG_B_CELL_RECEPTOR_SIGNALING_PATHWAY | KEGG_B_CELL_RECEPTOR_SIGNALING_PATHWAY | 74 | 0.519068779860351 | 1.78713727908906 | 4.07585536504985e-05 | 0.00108301299699896 | 0.000809041967197865 | 11253 | tags=58%, list=27%, signal=43% | IFITM1/NFKBIA/INPP5D/MAPK3/LYN/VAV1/NFKB1/RELA/PLCG2/RAC1/FCGR2B/NFATC1/NFKBIB/CD72/NFATC4/BLNK/RAC2/CD79A/PRKCB/LILRB3/SOS2/RAF1/CD19/VAV3/BCL10/GRB2/MAP2K2/JUN/NFKBIE/CD79B/IKBKG/PIK3AP1/PIK3CD/HRAS/GSK3B/PPP3R1/CARD11/CD81/SYK/RASGRP3/FOS/PTPN6/PIK3R5 |
| KEGG_PROTEASOME | KEGG_PROTEASOME | KEGG_PROTEASOME | 45 | 0.569095795554976 | 1.84926871525364 | 0.000117391053374848 | 0.00272934199096522 | 0.00203889724282631 | 16465 | tags=87%, list=39%, signal=53% | POMP/PSMA6/PSME1/PSMA8/PSMD4/PSMC1/PSMB10/PSMB1/PSMB3/PSMD7/PSMB8/PSMB9/PSMB11/PSME2/PSMA4/PSMC1P4/PSMA5/PSMD13/PSMC4/PSMA2/PSMD3/PSMA7/PSMB4/PSMD8/PSMB2/PSMA1/PSMC3/PSMC5/PSMB7/PSMD2/PSMD6/SEM1/PSMB6/PSMD14/PSMD1/PSMA3/PSMC2/PSMD11/PSMB5 |
| KEGG_NOD_LIKE_RECEPTOR_SIGNALING_PATHWAY | KEGG_NOD_LIKE_RECEPTOR_SIGNALING_PATHWAY | KEGG_NOD_LIKE_RECEPTOR_SIGNALING_PATHWAY | 61 | 0.521986151164327 | 1.76275051813631 | 0.000255037460305694 | 0.00527077417965101 | 0.00393742043980721 | 3569 | tags=23%, list=9%, signal=21% | TNFAIP3/MAPK10/NFKBIA/TRIP6/MAPK13/MAPK3/CASP5/CASP1/NAIP/NFKB1/RELA/CCL11/CCL13/NFKBIB |
| KEGG_EPITHELIAL_CELL_SIGNALING_IN_HELICOBACTER_PYLORI_INFECTION | KEGG_EPITHELIAL_CELL_SIGNALING_IN_HELICOBACTER_PYLORI_INFECTION | KEGG_EPITHELIAL_CELL_SIGNALING_IN_HELICOBACTER_PYLORI_INFECTION | 68 | 0.502939797557996 | 1.71914580361568 | 0.000366548357444944 | 0.0061979994986145 | 0.00463008451509403 | 10542 | tags=47%, list=25%, signal=35% | MAPK10/NFKBIA/MAPK13/ATP6V0A1/ATP6V0E1/LYN/NFKB1/RELA/PLCG2/RAC1/MET/TCIRG1/ATP6V1E1/CXCR2/CSK/JUN/ADAM17/CDC42/IKBKG/ATP6V1F/ATP6V1C1/ATP6AP1/ATP6V0B/ATP6V1B1/ATP6V1H/MAPK14/CXCR1/ATP6V1A/ATP6V1D/ATP6V1G1/ATP6V0C/ATP6V1G2 |
| KEGG_RIBOSOME | KEGG_RIBOSOME | KEGG_RIBOSOME | 88 | 0.467056345384354 | 1.64928849995729 | 0.000357901575679576 | 0.0061979994986145 | 0.00463008451509403 | 17481 | tags=81%, list=42%, signal=47% | RPL28/RPL36AL/RPS9/FAU/RPL26L1/RPS15/MRPL13/RPL8/RPL18/RPL24/RPS5/RPL9/RPL35/RPS11/RPL13A/RPS26/RPL13/RPL18A/RPS16/RPS19/RPL36/RPS2/RPS20/RPL41/RPSA/RPL11/RPLP2/RPS7/RPL23/RPLP0/RPS10/RPL38/RPL32/RPL27A/RPS3/RPS15A/RPL19/RPL37/RPL39/RPL29/RPL37A/RPL27/RPS18/RPS21/RPL10/RPS4X/RPL14/RPL30/RPS13/RPLP1/RPS27/RPL22L1/RPL12/RPS28/RPL34/RPS29/RPL10A/RPS17/RPL3/RPL35A/RPS6/RPL23A/RPS25/RPS3A/RPS8/RPS23/RPL4/RPS27A/RPL15/RPL17/RPL31 |
| KEGG_CARDIAC_MUSCLE_CONTRACTION | KEGG_CARDIAC_MUSCLE_CONTRACTION | KEGG_CARDIAC_MUSCLE_CONTRACTION | 76 | 0.474795498408873 | 1.64196232983661 | 0.00046560161834554 | 0.00721682508435586 | 0.00539117663347467 | 9369 | tags=50%, list=22%, signal=39% | SLC8A1/UQCRHL/MYL3/COX7A1/CACNA2D1/COX6B2/SLC9A1/FXYD2/TNNC1/MYL2/MYH7/CACNG1/ATP1B4/MYH6/COX6A2/COX7B/CACNA1S/ATP1A2/COX5A/COX4I2/UQCRH/ATP1B1/CACNA1D/CACNG2/UQCR10/UQCRC2/COX4I1/UQCR11/TPM2/COX6A1/COX5B/ATP1B3/COX6B1/COX7A2/COX7C/CACNG3/CYC1/COX6C |
| KEGG_CHEMOKINE_SIGNALING_PATHWAY | KEGG_CHEMOKINE_SIGNALING_PATHWAY | KEGG_CHEMOKINE_SIGNALING_PATHWAY | 185 | 0.405282285253701 | 1.51305179298003 | 0.000564966091732101 | 0.00808336100478237 | 0.00603850397640788 | 13421 | tags=50%, list=32%, signal=34% | WAS/JAK3/NFKBIA/ADCY4/PREX1/PRKCZ/CXCR4/MAPK3/NCF1/GNG10/GNG5/RAP1A/ARRB2/GNB2/LYN/VAV1/FOXO3/NFKB1/RELA/CXCL13/GNG8/XCR1/CCL11/CCL13/CCL21/RAC1/NFKBIB/CXCL12/GRK1/CXCR5/RAC2/CXCR2/GRK3/CRK/CXCL16/PRKCB/PTK2B/SOS2/RAF1/CCR6/GNB3/CXCR3/VAV3/PF4/CSK/GRB2/GNG3/STAT3/PRKCD/GNAI2/STAT2/CCL22/CDC42/STAT5B/IKBKG/ADCY2/PIK3CD/CCR10/CXCR1/HRAS/CXCR6/CXCL14/SHC3/STAT1/GNG11/JAK2/GSK3B/CCL14/CCL18/ADCY5/PLCB3/RHOA/GRK5/GNG12/PIK3R5/CCL3/GSK3A/GNG13/CCR5/HCK/CCL4/PPBP/PARD3/CCL20/CCL25/PRKACA/GRK2/CCL24/MAP2K1/CX3CL1/FGR/ADCY1 |
| KEGG_NEUROTROPHIN_SIGNALING_PATHWAY | KEGG_NEUROTROPHIN_SIGNALING_PATHWAY | KEGG_NEUROTROPHIN_SIGNALING_PATHWAY | 124 | 0.423167385344818 | 1.54055862253966 | 0.000679970479566111 | 0.00903389351423547 | 0.00674857919569373 | 13514 | tags=48%, list=32%, signal=32% | IRS2/MAPK10/IRAK3/NFKBIA/MAPK13/MAPK3/RAP1A/TP73/FOXO3/NFKB1/RELA/PLCG2/CALML3/NGF/RAC1/NFKBIB/MAPKAPK2/CAMK2B/GAB1/CRK/SH2B2/ARHGDIA/SOS2/RAF1/CSK/GRB2/CAMK2A/MAP2K2/JUN/PRKCD/CALML6/ARHGDIB/NFKBIE/CDC42/CALM3/MAP3K5/BAD/MAP3K3/NTRK3/PIK3CD/BCL2/MAPK14/HRAS/SHC3/GSK3B/ATF4/YWHAZ/RHOA/PIK3R5/NTRK2/YWHAE/PSEN1/RPS6KA3/BAX/IRAK1/BEX3/MAP2K1/IRAK4/CALM2 |
| KEGG_LEISHMANIA_INFECTION | KEGG_LEISHMANIA_INFECTION | KEGG_LEISHMANIA_INFECTION | 70 | 0.486208760481875 | 1.66854974488288 | 0.00118663971157131 | 0.0147143324234842 | 0.01099203101245 | 9437 | tags=44%, list=23%, signal=34% | NFKBIA/TLR2/TGFB1/MAPK13/MAPK3/NCF1/FCGR2A/IFNGR2/NFKB1/RELA/IL1A/NFKBIB/C3/HLA-DQA2/IFNGR1/TLR4/NCF2/MYD88/PRKCB/CR1/CYBA/HLA-DMA/JUN/IL12A/MAPK14/HLA-DOA/HLA-DMB/STAT1/FCGR3B/IL10/JAK2 |
| KEGG_FC_GAMMA_R_MEDIATED_PHAGOCYTOSIS | KEGG_FC_GAMMA_R_MEDIATED_PHAGOCYTOSIS | KEGG_FC_GAMMA_R_MEDIATED_PHAGOCYTOSIS | 96 | 0.439436004734638 | 1.56404461976169 | 0.00149176651234207 | 0.0173417857059766 | 0.0129548144492864 | 8397 | tags=39%, list=20%, signal=31% | WAS/MYO10/INPP5D/PRKCE/MAPK3/NCF1/GAB2/FCGR2A/AMPH/LIMK1/LYN/VAV1/WASF3/PLCG2/RAC1/FCGR2B/ARPC1A/RAC2/CRK/PLPP3/PRKCB/ARPC3/RAF1/VAV3/VASP/PLPP2/PRKCD/ARPC2/SPHK2/PRKCG/CDC42/LIMK2/SPHK1/DNM2/PIK3CD/PLPP1/MARCKS |
| KEGG_ARGININE_AND_PROLINE_METABOLISM | KEGG_ARGININE_AND_PROLINE_METABOLISM | KEGG_ARGININE_AND_PROLINE_METABOLISM | 54 | 0.489926858713773 | 1.63643415674199 | 0.00175727590679532 | 0.0192266658037606 | 0.0143628742846429 | 13209 | tags=48%, list=32%, signal=33% | MAOA/P4HA2/CKMT1B/GOT2/GOT1/GATM/CKM/CKMT1A/SMS/NOS1/AOC1/ALDH2/ODC1/CKMT2/SAT1/PYCR3/SRM/DAO/ACY1/ARG2/CPS1/LAP3/ALDH1B1/NOS2/SAT2/PYCR1 |
| KEGG_TOLL_LIKE_RECEPTOR_SIGNALING_PATHWAY | KEGG_TOLL_LIKE_RECEPTOR_SIGNALING_PATHWAY | KEGG_TOLL_LIKE_RECEPTOR_SIGNALING_PATHWAY | 95 | 0.446752340691491 | 1.58873362551073 | 0.00224857654626196 | 0.0232352909780403 | 0.0173574329886888 | 11304 | tags=43%, list=27%, signal=32% | MAPK10/NFKBIA/TLR2/MAPK13/TLR6/MAPK3/TLR5/LY96/IRF7/MAP2K3/NFKB1/RELA/IFNAR1/RAC1/CD80/IFNA14/IFNA21/TLR4/CD40/MAP2K6/MYD88/MAP2K2/JUN/IL12A/IRF3/TLR9/IKBKG/PIK3CD/TICAM1/TRAF3/MAPK14/STAT1/IRF5/CTSK/FADD/IKBKE/FOS/MAP3K8/PIK3R5/CCL3/TBK1 |
| KEGG_TYPE_I_DIABETES_MELLITUS | KEGG_TYPE_I_DIABETES_MELLITUS | KEGG_TYPE_I_DIABETES_MELLITUS | 40 | 0.524030987996265 | 1.68169197595572 | 0.00314101250106619 | 0.0307488592209637 | 0.0229702853263566 | 10000 | tags=48%, list=24%, signal=36% | HLA-B/HLA-E/IL1A/PTPRN/CD80/HLA-C/HLA-DQA2/FAS/IL2/HLA-A/HLA-DMA/GAD2/IL12A/CPE/LTA/HLA-DOA/HLA-DMB/HLA-F/PTPRN2 |
| KEGG_DNA_REPLICATION | KEGG_DNA_REPLICATION | KEGG_DNA_REPLICATION | 36 | 0.532173317164005 | 1.67851077567251 | 0.00376593185549601 | 0.0350231662561129 | 0.0261633160487091 | 16411 | tags=81%, list=39%, signal=49% | RPA4/MCM2/RFC2/MCM5/RPA3/POLD1/MCM6/PRIM2/MCM4/RFC4/MCM3/RPA1/POLE2/POLD4/POLD3/FEN1/PCNA/POLA2/PRIM1/LIG1/POLE4/RNASEH2A/SSBP1/RNASEH2C/MCM7/RPA2/DNA2/POLA1/RFC3 |
| KEGG_GLYCOSYLPHOSPHATIDYLINOSITOL_GPI_ANCHOR_BIOSYNTHESIS | KEGG_GLYCOSYLPHOSPHATIDYLINOSITOL_GPI_ANCHOR_BIOSYNTHESIS | KEGG_GLYCOSYLPHOSPHATIDYLINOSITOL_GPI_ANCHOR_BIOSYNTHESIS | 25 | -0.437885089 | -1.743478701 | 0.0040396691654141 | 0.0357799268936677 | 0.0267286380869504 | 6380 | tags=40%, list=15%, signal=34% | PIGB/PIGF/PIGK/PIGO/PIGQ/PGAP1/PIGM/PIGL/DPM2/PIGC |
| KEGG_CITRATE_CYCLE_TCA_CYCLE | KEGG_CITRATE_CYCLE_TCA_CYCLE | KEGG_CITRATE_CYCLE_TCA_CYCLE | 30 | 0.545222735001976 | 1.6694904835042 | 0.00444721152199928 | 0.0375991519587212 | 0.0280876517178902 | 13883 | tags=60%, list=33%, signal=40% | PCK1/ACO1/SDHB/SDHD/PDHB/OGDHL/PCK2/SDHA/MDH2/IDH3G/IDH3B/FH/SUCLG2/IDH3A/DLD/SUCLG2P2/MDH1/ACO2 |
| KEGG_OOCYTE_MEIOSIS | KEGG_OOCYTE_MEIOSIS | KEGG_OOCYTE_MEIOSIS | 109 | 0.411934292872352 | 1.47884395944945 | 0.00468082113265335 | 0.0378535969858054 | 0.0282777294970363 | 16883 | tags=61%, list=40%, signal=36% | ADCY4/MAPK3/IGF1R/PTTG2/CPEB1/ANAPC13/RBX1/REC8/CALML3/CDC26/CAMK2B/MAD2L2/STAG3/CAMK2A/CALML6/CALM3/PPP2R1A/ADCY2/CDC25C/MAD2L1/CDC20/MOS/CCNB2/PPP3R1/PLK1/PTTG1/SPDYA/ADCY5/YWHAZ/CCNE1/PPP2CA/IGF1/BUB1/CCNB1/AR/YWHAE/PPP3R2/ITPR2/FBXO43/PPP1CB/RPS6KA3/PRKACA/PKMYT1/CDK1/MAP2K1/ADCY1/CALM2/CUL1/CCNE2/SGO1/FBXW11/ANAPC11/PPP1CA/RPS6KA1/YWHAB/ESPL1/PPP2R5A/ANAPC10/SMC1A/PPP2R1B/FBXO5/MAPK1/PPP2R5C/ITPR1/CDK2/SPDYC |

**Table S9**

|  | ID | Description | setSize | enrichmentScore | NES | pvalue | p.adjust | qvalue | rank | leading_edge | core_enrichment |
| --- | --- | --- | --- | --- | --- | --- | --- | --- | --- | --- | --- |
| KEGG_GRAFT_VERSUS_HOST_DISEASE | KEGG_GRAFT_VERSUS_HOST_DISEASE | KEGG_GRAFT_VERSUS_HOST_DISEASE | 37 | -0.681794114 | -3.12036684 | 1e-10 | 6.16666666666667e-09 | 2.21052631578947e-09 | 9255 | tags=78%, list=22%, signal=61% | HLA-DRB1/IL1A/HLA-DOA/HLA-G/HLA-DPB1/HLA-DPA1/KIR3DL1/HLA-DQA2/KLRC1/HLA-DRA/CD80/KIR2DL3/FASLG/HLA-DMB/KLRD1/HLA-DMA/IL6/HLA-DQB1/HLA-A/KIR2DL1/IFNG/HLA-F/GZMB/HLA-E/PRF1/HLA-C/HLA-B/HLA-DOB/FAS |
| KEGG_OXIDATIVE_PHOSPHORYLATION | KEGG_OXIDATIVE_PHOSPHORYLATION | KEGG_OXIDATIVE_PHOSPHORYLATION | 126 | 0.561847567793921 | 2.00635865149848 | 1e-10 | 6.16666666666667e-09 | 2.21052631578947e-09 | 15971 | tags=84%, list=38%, signal=52% | ATP6V0A4/PPA2/ATP12A/MT-CO1/COX15/MT-ND4L/NDUFA4L2/NDUFS1/ATP5PB/ATP6V1H/COX7A2L/NDUFA10/UQCRFS1/ATP6AP1/ATP5F1A/NDUFV3/ATP6V1E1/UQCRC2/ATP6V0A2/ATP5F1B/ATP6V1C1/ATP6V1B1/MT-ATP8/ATP6V1A/NDUFC2/ATP6V1D/MT-ND4/ATP5F1C/NDUFA4/NDUFS2/ATP5PF/MT-CO2/ATP6V1G3/MT-CO3/ATP5PD/NDUFS5/UQCRC1/NDUFS3/NDUFB3/SDHC/SDHD/COX17/NDUFB5/COX5A/UQCRH/SDHB/MT-CYB/ATP5MF/NDUFC1/ATP5MC3/ATP5MG/UQCRQ/ATP5MC2/MT-ATP6/NDUFS4/ATP5PO/NDUFA8/UQCRB/NDUFB8/ATP5ME/NDUFB10/NDUFB9/NDUFB1/COX6B1/NDUFAB1/NDUFS6/NDUFB4/NDUFB2/CYC1/COX7C/COX8A/COX7B/COX4I1/ATP6V1B2/ATP6V0D1/NDUFA7/MT-ND5/NDUFS8/NDUFA5/LHPP/NDUFA2/ATP5MC1/ATP6V1C2/ATP6V1F/COX7A2/ATP6V1E2/NDUFS7/ATP6V1G2/NDUFA11/ATP6V0B/UQCR11/ATP6V0C/UQCR10/NDUFB6/NDUFA9/MT-ND6/COX6A1/NDUFB7/ATP6V0E1/NDUFA1/MT-ND2/COX6C/NDUFA3/UQCRHL/COX5B/MT-ND3 |
| KEGG_HUNTINGTONS_DISEASE | KEGG_HUNTINGTONS_DISEASE | KEGG_HUNTINGTONS_DISEASE | 176 | 0.542120908717438 | 1.96682848391362 | 1e-10 | 6.16666666666667e-09 | 2.21052631578947e-09 | 14336 | tags=72%, list=34%, signal=48% | CREB3L4/RCOR1/CLTCL1/PLCB4/HDAC2/GNAQ/PLCB1/DCTN4/MT-CO1/HIP1/CASP3/POLR2K/AP2M1/NDUFA4L2/NDUFS1/REST/POLR2J3/POLR2J2/ATP5PB/COX7A2L/NDUFA10/UQCRFS1/VDAC2P5/ATP5F1A/NDUFV3/UQCRC2/DNAI1/ATP5F1B/DNALI1/VDAC3/DCTN1/MT-ATP8/DCTN2/BDNF/NDUFC2/DLG4/PLCB3/VDAC2/DNAL4/ATP5F1C/VDAC1/NDUFA4/CASP9/NDUFS2/ATP5PF/AP2A2/MT-CO2/CLTC/MT-CO3/SLC25A5/ATP5PD/NDUFS5/PPARGC1A/CLTA/POLR2L/APAF1/POLR2C/PPARG/POLR2D/UQCRC1/NDUFS3/NDUFB3/POLR2B/SDHC/SDHD/NDUFB5/COX5A/UQCRH/SDHB/MT-CYB/POLR2G/NDUFC1/ATP5MC3/HAP1/UQCRQ/AP2B1/ATP5MC2/MT-ATP6/NDUFS4/PLCB2/ATP5PO/NDUFA8/SLC25A6/HDAC1/UQCRB/NDUFB8/POLR2E/NDUFB10/NDUFB9/NDUFB1/COX6B1/NDUFAB1/NDUFS6/TFAM/NDUFB4/NDUFB2/CYC1/POLR2A/COX7C/COX8A/AP2A1/COX7B/COX4I1/AP2S1/NDUFA7/NDUFS8/CYCS/TBPL1/POLR2F/NDUFA5/NDUFA2/ATP5MC1/POLR2J/NRF1/COX7A2/CLTB/TBP/NDUFS7/UQCR11/UQCR10/POLR2I/SP1/TP53/GPX1/NDUFB6/CREB3/NDUFA9 |
| KEGG_ALLOGRAFT_REJECTION | KEGG_ALLOGRAFT_REJECTION | KEGG_ALLOGRAFT_REJECTION | 35 | -0.668446347 | -2.995450095 | 2.7179122923509e-10 | 1.25703443521229e-08 | 4.50601248468702e-09 | 10157 | tags=77%, list=24%, signal=58% | CD28/IL5/HLA-DRB1/HLA-DOA/HLA-G/HLA-DPB1/HLA-DPA1/HLA-DQA2/IL12A/HLA-DRA/CD80/FASLG/HLA-DMB/HLA-DMA/HLA-DQB1/IL10/HLA-A/IFNG/HLA-F/GZMB/HLA-E/PRF1/HLA-C/HLA-B/CD40/HLA-DOB/FAS |
| KEGG_SPLICEOSOME | KEGG_SPLICEOSOME | KEGG_SPLICEOSOME | 126 | 0.538146767961763 | 1.9217230536665 | 2.04876292849382e-09 | 7.58042283542713e-08 | 2.7173066209497e-08 | 15479 | tags=76%, list=37%, signal=48% | CTNNBL1/PRPF38A/SNRPD3/HNRNPK/CDC5L/SRSF9/RBMX/SF3A3/EFTUD2/DDX42/TXNL4A/HNRNPA1/AQR/PRPF4/PRPF8/LSM2/SRSF1/PRPF19/THOC1/U2AF2/SF3B4/CDC40/TCERG1/PRPF6/PPIH/RBM8A/NCBP1/HSPA1B/LSM5/CWC15/RBM17/HSPA1A/SNU13/LSM6/LSM4/PLRG1/SRSF8/SF3B2/DHX15/HNRNPM/SNRPC/LSM3/SNRPB/DDX46/SNRPA/PCBP1/SF3B6/SNRPE/PUF60/RBM25/PRPF40A/THOC2/SNW1/SRSF6/MAGOH/SLU7/SNRNP200/USP39/ISY1/SNRPG/HNRNPA3/SRSF2/SNRNP40/HNRNPU/DDX39B/TRA2A/SNRPD1/LSM7/HSPA2/CHERP/PPIE/SF3A2/ALYREF/NCBP2/CCDC12/SART1/PRPF31/RBM22/WBP11/SNRNP27/SRSF3/SF3B1/THOC3/PHF5A/SNRPB2/U2SURP/PRPF40B/MAGOHB/SNRPF/SF3B3/ACIN1/PQBP1/SNRPA1/PRPF18/DHX8/BCAS2 |
| KEGG_LYSOSOME | KEGG_LYSOSOME | KEGG_LYSOSOME | 120 | 0.541112340904132 | 1.92597930485304 | 4.50683201296908e-09 | 1.38960653733213e-07 | 4.9812353827553e-08 | 13831 | tags=64%, list=33%, signal=43% | HGSNAT/SUMF1/ATP6V0A4/LAPTM4B/CLTCL1/PPT2/AP4M1/CTSG/HEXA/CTNS/SORT1/AP3M1/GUSB/M6PR/GNS/GALC/AP1B1/CTSV/CTSC/ATP6V1H/SLC17A5/ATP6AP1/AGA/GLA/LAMP1/ASAH1/CD164/AP3B2/ATP6V0A2/PPT1/DNASE2B/CTSO/LAPTM4A/AP4B1/AP3S1/GLB1/LAMP2/NAGA/HEXB/TPP1/CD63/CLTC/AP1S2/ARSB/GNPTG/GGA1/GM2A/CLTA/AP3S2/AP1M1/CLN3/MAN2B1/NAGLU/CTSA/SCARB2/FUCA1/CLN5/MANBA/SLC11A2/CTSS/CTSD/PSAP/GGA3/AP1S1/LIPA/ATP6V0D1/AP3B1/LAPTM5/CD68/GALNS/HYAL1/CLTB/AP3D1/CTSF/ATP6V0B/ATP6V0C/DNASE2 |
| KEGG_ALZHEIMERS_DISEASE | KEGG_ALZHEIMERS_DISEASE | KEGG_ALZHEIMERS_DISEASE | 161 | 0.497632729627385 | 1.79728576513324 | 1.58004836304407e-08 | 4.17584210233076e-07 | 1.49688792288386e-07 | 15846 | tags=71%, list=38%, signal=44% | APP/CHP1/PLCB4/GNAQ/PLCB1/CACNA1C/MT-CO1/CASP3/CALM2/ATP2A3/MAPK1/NDUFA4L2/NCSTN/NDUFS1/BACE1/PPP3CB/ATP5PB/COX7A2L/NDUFA10/UQCRFS1/ATP5F1A/NDUFV3/UQCRC2/ATP5F1B/PPP3R1/CALML5/CAPN1/FADD/MT-ATP8/GAPDH/NDUFC2/PLCB3/ATP5F1C/NDUFA4/CASP9/NDUFS2/ATP5PF/PPP3CA/ADAM10/MT-CO2/MT-CO3/ATP5PD/NDUFS5/APOE/APAF1/PSEN1/UQCRC1/NDUFS3/NDUFB3/SDHC/SDHD/NDUFB5/COX5A/IDE/CDK5/UQCRH/CACNA1D/SDHB/MT-CYB/NDUFC1/ATP5MC3/UQCRQ/LPL/ATP5MC2/CALM1/MT-ATP6/NDUFS4/PLCB2/ATP5PO/NDUFA8/UQCRB/NDUFB8/HSD17B10/APH1A/NDUFB10/LRP1/NDUFB9/NDUFB1/COX6B1/NDUFAB1/NDUFS6/NDUFB4/NDUFB2/CYC1/COX7C/COX8A/COX7B/COX4I1/NDUFA7/NDUFS8/CYCS/NDUFA5/NDUFA2/ATP5MC1/ATP2A2/COX7A2/BAD/NDUFS7/MAPT/UQCR11/PSEN2/UQCR10/NDUFB6/NDUFA9/ATF6/COX6A1/NDUFB7/GRIN2B/NAE1/NDUFA1/COX6C/NDUFA3/GRIN2A/UQCRHL/COX5B |
| KEGG_AUTOIMMUNE_THYROID_DISEASE | KEGG_AUTOIMMUNE_THYROID_DISEASE | KEGG_AUTOIMMUNE_THYROID_DISEASE | 43 | -0.567759353 | -2.628929731 | 3.22520502305049e-08 | 7.45828661580426e-07 | 2.67352521647606e-07 | 10157 | tags=86%, list=24%, signal=65% | IL4/TPO/IFNA21/IFNA2/IFNA1/IL2/CD28/TSHR/IL5/CGA/IFNA14/HLA-DRB1/HLA-DOA/HLA-G/HLA-DPB1/IFNA13/HLA-DPA1/HLA-DQA2/HLA-DRA/CD80/CTLA4/FASLG/HLA-DMB/HLA-DMA/HLA-DQB1/IL10/HLA-A/HLA-F/TG/GZMB/HLA-E/PRF1/HLA-C/HLA-B/CD40/HLA-DOB/FAS |
| KEGG_PARKINSONS_DISEASE | KEGG_PARKINSONS_DISEASE | KEGG_PARKINSONS_DISEASE | 125 | 0.517140879698103 | 1.84639229843581 | 3.76443998228492e-08 | 7.73801551914122e-07 | 2.77379788168363e-07 | 16311 | tags=82%, list=39%, signal=50% | SLC18A2/MT-CO1/CASP3/MT-ND4L/NDUFA4L2/NDUFS1/ATP5PB/COX7A2L/NDUFA10/UQCRFS1/VDAC2P5/ATP5F1A/NDUFV3/UCHL1/UQCRC2/ATP5F1B/VDAC3/MT-ATP8/NDUFC2/MT-ND4/VDAC2/ATP5F1C/VDAC1/NDUFA4/CASP9/NDUFS2/ATP5PF/MT-CO2/MT-CO3/SLC25A5/ATP5PD/NDUFS5/APAF1/UQCRC1/NDUFS3/NDUFB3/SDHC/SDHD/NDUFB5/COX5A/UQCRH/SDHB/UBE2G1/MT-CYB/NDUFC1/ATP5MC3/UQCRQ/ATP5MC2/MT-ATP6/NDUFS4/ATP5PO/NDUFA8/SLC25A6/UQCRB/NDUFB8/NDUFB10/SNCAIP/NDUFB9/UBE2G2/NDUFB1/COX6B1/PARK7/NDUFAB1/SEPTIN5/NDUFS6/NDUFB4/NDUFB2/CYC1/COX7C/COX8A/COX7B/COX4I1/NDUFA7/UBE2J1/MT-ND5/UBE2J2/NDUFS8/CYCS/NDUFA5/NDUFA2/ATP5MC1/COX7A2/NDUFS7/PRKN/UQCR11/UQCR10/NDUFB6/NDUFA9/MT-ND6/COX6A1/NDUFB7/UBE2L3/NDUFA1/MT-ND2/COX6C/NDUFA3/GPR37/UQCRHL/COX5B/MT-ND3/NDUFV1/PINK1 |
| KEGG_PURINE_METABOLISM | KEGG_PURINE_METABOLISM | KEGG_PURINE_METABOLISM | 158 | 0.482801571034659 | 1.74423566219738 | 1.8977689324213e-07 | 3.5108725249794e-06 | 1.25852044992149e-06 | 15077 | tags=61%, list=36%, signal=39% | AMPD3/PDE6G/ADSS2/ADCY6/PDE3A/PAPSS1/GUCY1A1/GUCY2D/AMPD1/PDE4C/NT5E/NME6/AK2/PDE10A/PAPSS2/POLR3B/POLR2K/POLE/NME5/POLR2J3/POLR2J2/IMPDH2/PDE8B/PNP/PDE7B/ADK/POLR3C/PFAS/NUDT5/PDE6D/POLE3/ADSS1/HPRT1/GART/ADSL/POLR3F/POLR2L/PKM/POLD2/PKLR/POLR2C/POLR2D/POLR2B/POLR1D/PAICS/GUCY1A2/POLR2G/ENTPD1/GUCY1B1/ADCY3/GMPS/IMPDH1/ITPA/ENTPD2/NT5C2/ADA/POLR1A/PDE6H/ENTPD5/ENPP1/POLR2E/POLD1/POLA2/RRM2/NME1/DGUOK/POLR2A/PPAT/CANT1/GMPR2/NME7/POLR1H/POLD3/NME2/PRPS1/POLR2F/NUDT2/POLR2J/POLR3A/ENTPD8/POLR1C/POLR3D/APRT/NT5C/ADCY2/POLR2I/AMPD2/ADCY8/PDE11A/PDE7A/RRM1/POLR3H/ADPRM/POLD4/POLA1/NME1-NME2 |
| KEGG_PATHWAYS_IN_CANCER | KEGG_PATHWAYS_IN_CANCER | KEGG_PATHWAYS_IN_CANCER | 319 | 0.412500607113828 | 1.52908613550702 | 4.1726740661907e-07 | 7.01767911132072e-06 | 2.51558340832549e-06 | 12652 | tags=44%, list=30%, signal=31% | PLD1/DAPK1/PIK3CB/LAMB2/CCNA1/E2F3/ZBTB16/KIT/BMP2/MSH3/HGF/CDK6/ARNT/CRKL/FZD6/LAMB4/HDAC2/FGF20/FGF16/HHIP/FZD9/LAMB1/CTNNA1/MECOM/VEGFA/FZD10/RALBP1/COL4A2/JUP/CDC42/CEBPA/APPL1/MITF/STAT5A/FZD1/CASP3/ABL1/LAMA3/SMO/NTRK1/CCND1/MAPK1/TPM3/DVL3/MAP2K1/PIK3CG/NCOA4/FZD8/LAMA5/RALA/WNT11/CDK4/EGFR/ELOC/FZD7/E2F2/SKP2/MSH6/RHOA/LAMA4/CTBP2/MAPK8/COL4A1/EGLN2/BCR/FADD/RXRG/CDKN2A/TGFB2/CASP9/TGFBR1/SMAD2/FGF18/GSTP1/CTNNA2/CDK2/MAX/ITGAV/PDGFA/CTNNB1/ARAF/WNT7B/SUFU/PIK3R1/RUNX1T1/NRAS/CBL/PIK3R3/LAMA1/KITLG/PPARG/RASSF1/RUNX1/AKT2/STK36/MMP9/RXRA/FH/CKS1B/MLH1/CDH1/CSF2RA/RBX1/RAC3/CBLC/CDKN1B/BMP4/PDGFRA/DCC/HSP90AB1/HDAC1/AKT1/RET/RAC1/PIK3R2/APC2/GRB2/FGF1/KRAS/RAD51/DVL2/WNT5B/RXRB/TGFBR2/TCF7L2/PIAS4/LAMC2/STAT3/FZD5/FGF2/CYCS/ELOB/RAC2/GLI2/CSF1R/BIRC5/MTOR/NOS2/BIRC2 |
| KEGG_INTESTINAL_IMMUNE_NETWORK_FOR_IGA_PRODUCTION | KEGG_INTESTINAL_IMMUNE_NETWORK_FOR_IGA_PRODUCTION | KEGG_INTESTINAL_IMMUNE_NETWORK_FOR_IGA_PRODUCTION | 46 | -0.478235584 | -2.24105535 | 2.36922175569005e-06 | 3.65255020668883e-05 | 1.30930675972345e-05 | 10157 | tags=59%, list=24%, signal=44% | CD28/IL5/HLA-DRB1/HLA-DOA/PIGR/HLA-DPB1/HLA-DPA1/HLA-DQA2/TNFRSF13C/HLA-DRA/CD80/AICDA/IL15RA/ICOS/HLA-DMB/HLA-DMA/TNFRSF13B/IL6/HLA-DQB1/IL10/MAP3K14/TNFSF13B/ITGB7/CCR10/CD40/HLA-DOB/IL15 |
| KEGG_PEROXISOME | KEGG_PEROXISOME | KEGG_PEROXISOME | 77 | 0.546345230081452 | 1.87866410512512 | 2.70967174704796e-06 | 3.85607133233749e-05 | 1.38226170092325e-05 | 11288 | tags=56%, list=27%, signal=41% | CAT/AMACR/HSD17B4/AGPS/FAR2/IDH1/SLC27A2/PECR/PEX5/PEX11A/NUDT12/PEX10/PEX13/PXMP2/DDO/GNPAT/ACOX2/MLYCD/PEX7/PHYH/MVK/NUDT19/IDH2/PEX19/ACAA1/PRDX5/MPV17/PEX2/SCP2/ECH1/PEX12/PIPOX/ACOX3/DHRS4/HMGCL/PEX26/PEX3/HACL1/PXMP4/ACOT8/SLC25A17/PMVK/DAO |
| KEGG_FOCAL_ADHESION | KEGG_FOCAL_ADHESION | KEGG_FOCAL_ADHESION | 197 | 0.434715416717033 | 1.58212426805938 | 5.09621678187315e-06 | 6.73428646176094e-05 | 2.41399742299254e-05 | 13179 | tags=44%, list=32%, signal=30% | ITGA9/PDGFC/PIK3CB/LAMB2/SHC4/HGF/CRKL/ACTN4/LAMB4/MYLK2/TLN2/LAMB1/VEGFA/THBS4/VCL/COL4A2/CDC42/SPP1/COL2A1/LAMA3/CCND1/MAPK1/ITGA4/MAP2K1/PIK3CG/LAMA5/ITGA5/ACTG1/DIAPH1/EGFR/TNC/RHOA/LAMA4/MAPK8/ITGB4/COL4A1/SHC1/THBS3/ACTB/FLNB/PARVB/ACTN1/ELK1/KDR/COL11A1/ITGAV/PDGFA/CTNNB1/RELN/PARVG/PIK3R1/PAK4/ITGA7/PIK3R3/LAMA1/AKT2/MYL12B/CCND3/RAC3/COL11A2/VAV3/PDGFRA/RAP1B/TLN1/VWF/AKT1/RAC1/PIK3R2/GRB2/PAK5/DOCK1/CCND2/ARHGAP35/LAMC2/RAC2/PPP1CB/PPP1CA/BIRC2/ILK/COL6A6/PIK3CA/PARVA/BAD/MAPK9/PPP1CC/PAK2 |
| KEGG_PYRIMIDINE_METABOLISM | KEGG_PYRIMIDINE_METABOLISM | KEGG_PYRIMIDINE_METABOLISM | 98 | 0.505597049118982 | 1.77610282165184 | 6.83560742528555e-06 | 8.43058249118552e-05 | 3.02205801959993e-05 | 16153 | tags=74%, list=39%, signal=46% | TXNRD1/UPP2/NT5E/NME6/TK2/POLR3B/UCK2/POLR2K/POLE/NME5/POLR2J3/POLR2J2/PNP/DUT/POLR3C/TXNRD2/POLE3/TK1/POLR3F/DPYD/POLR2L/POLD2/AK3/POLR2C/POLR2D/UPRT/POLR2B/POLR1D/POLR2G/ENTPD1/ITPA/DTYMK/NT5C2/CDA/POLR1A/ENTPD5/POLR2E/POLD1/POLA2/RRM2/NME1/CTPS1/POLR2A/DCTD/CANT1/TYMS/NME7/POLR1H/POLD3/NME2/POLR2F/NUDT2/POLR2J/POLR3A/ENTPD8/UMPS/UCK1/POLR1C/POLR3D/NT5C/DHODH/POLR2I/RRM1/POLR3H/POLD4/POLA1/NME1-NME2/UPP1/CMPK1/POLE4/UCKL1/NT5M/POLR3K |
| KEGG_ADHERENS_JUNCTION | KEGG_ADHERENS_JUNCTION | KEGG_ADHERENS_JUNCTION | 73 | 0.53218872496811 | 1.81575549458558 | 1.34475843914226e-05 | 0.000155487694525824 | 5.5736698464449e-05 | 11319 | tags=53%, list=27%, signal=39% | CTNND1/INSR/NECTIN1/SORBS1/NLK/WASF1/WASF2/ACTN4/CSNK2A1/CTNNA1/VCL/CDC42/NECTIN3/BAIAP2/MAPK1/WASL/FER/YES1/ACTG1/EGFR/RHOA/PTPRF/ACTB/TJP1/TGFBR1/ACP1/ACTN1/SMAD2/CTNNA2/CTNNB1/CSNK2B/CDH1/RAC3/NECTIN4/RAC1/TGFBR2/TCF7L2/MAP3K7/SSX2IP |
| KEGG_N_GLYCAN_BIOSYNTHESIS | KEGG_N_GLYCAN_BIOSYNTHESIS | KEGG_N_GLYCAN_BIOSYNTHESIS | 46 | 0.591839424526017 | 1.92181318714439 | 1.61897211275818e-05 | 0.000176182259329566 | 6.31549492902571e-05 | 12796 | tags=67%, list=31%, signal=47% | STT3B/ALG2/GANAB/RPN1/TUSC3/STT3A/RPN2/DDOST/MGAT2/MGAT5/DAD1/B4GALT2/MAN2A2/DOLPP1/ALG8/ALG3/ALG14/ALG10B/RFT1/DPM3/DPAGT1/MOGS/ALG9/MGAT5B/ALG11/MAN1B1/ALG5/MGAT4B/ALG6/ALG1/ALG10 |
| KEGG_VALINE_LEUCINE_AND_ISOLEUCINE_DEGRADATION | KEGG_VALINE_LEUCINE_AND_ISOLEUCINE_DEGRADATION | KEGG_VALINE_LEUCINE_AND_ISOLEUCINE_DEGRADATION | 44 | 0.59101929961474 | 1.90772034649965 | 1.89033605455041e-05 | 0.000194284538939904 | 6.96439599044889e-05 | 10495 | tags=61%, list=25%, signal=46% | HADHB/PCCB/HADHA/ACAT2/ACAD8/ACADM/AUH/IVD/BCKDHB/DLD/MMUT/BCKDHA/HIBADH/ALDH7A1/ALDH3A2/HADH/ALDH2/ACAA1/MCCC1/ECHS1/ALDH9A1/MCEE/HIBCH/HMGCL/ACADS/HSD17B10/BCAT1 |
| KEGG_GLYCOLYSIS_GLUCONEOGENESIS | KEGG_GLYCOLYSIS_GLUCONEOGENESIS | KEGG_GLYCOLYSIS_GLUCONEOGENESIS | 59 | 0.544617867261692 | 1.82435171359607 | 2.29051679902527e-05 | 0.000223024004115618 | 7.99460157000509e-05 | 8820 | tags=53%, list=21%, signal=42% | ALDH3B2/HK2/PDHA1/PGM2/GPI/LDHA/ADH6/PGAM4/ENO1/DLD/ALDH3B1/ACSS2/PDHB/ADH1A/HK3/PGAM1/G6PC2/ALDH7A1/GAPDH/PGK1/ALDH3A2/PGM1/ALDH2/PFKL/PKM/PKLR/ALDH9A1/ADH5/TPI1/LDHC/DLAT |
| KEGG_REGULATION_OF_ACTIN_CYTOSKELETON | KEGG_REGULATION_OF_ACTIN_CYTOSKELETON | KEGG_REGULATION_OF_ACTIN_CYTOSKELETON | 206 | 0.4158748443124 | 1.51786974316489 | 2.44833773083787e-05 | 0.000226471240102503 | 8.1181724759361e-05 | 16270 | tags=55%, list=39%, signal=34% | ITGA9/PIP5K1B/PDGFC/PIK3CB/ARHGEF6/IQGAP3/WASF1/WASF2/CRKL/ACTN4/FGF20/FGF16/MYLK2/MSN/CHRM3/GSN/ITGAE/VCL/ARPC5/CDC42/ENAH/BAIAP2/NCKAP1L/MYH14/MAPK1/SSH1/ITGA4/MAP2K1/WASL/PIK3CG/ITGA5/ITGAM/ACTG1/DIAPH1/EGFR/ARPC2/RDX/RHOA/ITGB4/ACTB/ACTN1/FGF18/MYH9/ITGAV/CYFIP1/PDGFA/DIAPH2/ARAF/PIK3R1/BRK1/PAK4/NRAS/ITGA7/PIK3R3/GNG12/PFN1/MYL12B/ARPC4/ARPC1B/ARPC3/RAC3/CFL1/VAV3/ITGB2/CHRM2/PDGFRA/ARPC1A/RAC1/PIP4K2B/DIAPH3/PIK3R2/APC2/FGF1/PAK5/KRAS/DOCK1/IQGAP2/GIT1/ARHGAP35/FGF2/PIKFYVE/CD14/RAC2/PPP1CB/PPP1CA/PIK3CA/PPP1CC/PAK2/ARHGEF7/BCAR1/PFN2/FGF17/TMSB4XP8/ITGB8/FGD1/MRAS/CYFIP2/PFN4/MYL12A/BRAF/BDKRB1/PIP5K1C/FGF11/FGF12/ROCK1/BDKRB2/TMSB4Y/ROCK2/FGFR2/MAP2K2/FGF5/MYLK/TMSB4X |
| KEGG_CITRATE_CYCLE_TCA_CYCLE | KEGG_CITRATE_CYCLE_TCA_CYCLE | KEGG_CITRATE_CYCLE_TCA_CYCLE | 30 | 0.6460193042733 | 1.94885505017194 | 2.82969525775187e-05 | 0.000249282677468617 | 8.9358797613217e-05 | 12010 | tags=73%, list=29%, signal=52% | PDHA1/IDH1/CS/DLD/ACLY/PDHB/PC/OGDH/IDH2/SUCLG1/SDHC/MDH2/SDHD/FH/ACO2/SDHB/DLAT/IDH3G/DLST/SUCLA2/MDH1/IDH3B |
| KEGG_AMINO_SUGAR_AND_NUCLEOTIDE_SUGAR_METABOLISM | KEGG_AMINO_SUGAR_AND_NUCLEOTIDE_SUGAR_METABOLISM | KEGG_AMINO_SUGAR_AND_NUCLEOTIDE_SUGAR_METABOLISM | 43 | 0.580394341556089 | 1.86768827537251 | 5.24646220057945e-05 | 0.000441179775957818 | 0.000158146946716031 | 13718 | tags=70%, list=33%, signal=47% | HK2/PGM2/FPGT/CMAS/GPI/HEXA/GFPT2/GALE/CHIT1/HK3/CYB5R1/PGM1/HEXB/UXS1/NANS/CYB5R3/GMDS/NANP/GNPDA1/UGP2/UGDH/GMPPA/MPI/HK1/RENBP/GALK1/GNE/PMM1/GALK2/NPL |
| KEGG_INSULIN_SIGNALING_PATHWAY | KEGG_INSULIN_SIGNALING_PATHWAY | KEGG_INSULIN_SIGNALING_PATHWAY | 136 | 0.452564500433592 | 1.62295139027789 | 5.6295202107933e-05 | 0.000452809234346418 | 0.000162315685711661 | 13160 | tags=50%, list=32%, signal=34% | PIK3CB/HK2/INSR/SORBS1/SHC4/PDE3A/CRKL/RHEB/ACACA/PHKA2/PYGL/PRKAA1/CALM2/EIF4E/MAPK1/EXOC7/GYS1/PHKG2/MAP2K1/PIK3CG/PRKACA/PRKAB1/SOCS4/PTPRF/CALML5/HK3/MAPK8/PRKCI/SHC1/G6PC2/PHKB/EIF4EBP1/PRKAB2/PRKAR2A/ELK1/ARAF/PRKAR1A/PPARGC1A/PIK3R1/NRAS/CBL/EIF4E2/PIK3R3/PKLR/AKT2/CBLC/CALM1/SOCS2/PPP1R3D/FLOT2/AKT1/PIK3R2/GRB2/KRAS/FBP1/RPS6/PRKACG/HK1/PYGB/MKNK1/PPP1CB/MTOR/PPP1CA/RPS6KB2/PIK3CA/BAD/MAPK9/PPP1CC |
| KEGG_ANTIGEN_PROCESSING_AND_PRESENTATION | KEGG_ANTIGEN_PROCESSING_AND_PRESENTATION | KEGG_ANTIGEN_PROCESSING_AND_PRESENTATION | 74 | -0.347547482 | -1.875908576 | 8.29980967969279e-05 | 0.000619008321495692 | 0.000221892029896038 | 8369 | tags=47%, list=20%, signal=38% | KLRC4/HLA-G/CD8B/HLA-DPB1/IFNA13/HLA-DPA1/KIR3DL1/HLA-DQA2/KLRC1/KIR2DS4/LTA/HLA-DRA/KIR2DL3/CD74/LGMN/HLA-DMB/KLRD1/HLA-DMA/PSME1/HLA-DQB1/HLA-A/KIR2DL1/KLRC3/HLA-F/PSME2/HSPA6/HLA-E/KIR2DL4/HLA-C/B2M/HLA-B/HLA-DOB/TAPBP/TAP1/TAP2 |
| KEGG_CHRONIC_MYELOID_LEUKEMIA | KEGG_CHRONIC_MYELOID_LEUKEMIA | KEGG_CHRONIC_MYELOID_LEUKEMIA | 73 | 0.510184078382445 | 1.74067863543805 | 8.69957641020973e-05 | 0.000619008321495692 | 0.000221892029896038 | 11050 | tags=49%, list=26%, signal=36% | PIK3CB/E2F3/SHC4/CDK6/CRKL/HDAC2/MECOM/STAT5A/ABL1/CCND1/MAPK1/MAP2K1/PIK3CG/CDK4/E2F2/CTBP2/SHC1/BCR/CDKN2A/TGFB2/TGFBR1/ARAF/PIK3R1/NRAS/CBL/PIK3R3/RUNX1/AKT2/CBLC/CDKN1B/HDAC1/AKT1/PIK3R2/GRB2/KRAS/TGFBR2 |
| KEGG_GAP_JUNCTION | KEGG_GAP_JUNCTION | KEGG_GAP_JUNCTION | 90 | 0.491830454732222 | 1.71207063567099 | 8.47760241208402e-05 | 0.000619008321495692 | 0.000221892029896038 | 11184 | tags=46%, list=27%, signal=33% | PDGFC/GNAI1/GJA1/HTR2A/ADCY6/PLCB4/GUCY1A1/TUBAL3/TUBA3E/GNAQ/PLCB1/TUBA1C/TUBB/GJD2/DRD2/MAPK1/MAP2K1/PRKACA/EGFR/GNAI3/TUBA1A/DRD1/TJP1/GNA11/PLCB3/PDGFA/TUBA1B/NRAS/GNAI2/ADRB1/GUCY1A2/TUBB2B/MAP2K5/GUCY1B1/ADCY3/PDGFRA/PLCB2/GRB2/KRAS/GRM1/PRKACG |
| KEGG_ECM_RECEPTOR_INTERACTION | KEGG_ECM_RECEPTOR_INTERACTION | KEGG_ECM_RECEPTOR_INTERACTION | 84 | 0.486988107501204 | 1.68669849045338 | 9.4099328933222e-05 | 0.000644754661209114 | 0.000231121158783352 | 7612 | tags=32%, list=18%, signal=26% | ITGA9/LAMB2/SV2A/LAMB4/LAMB1/SV2C/THBS4/CD36/COL4A2/SPP1/COL2A1/LAMA3/ITGA4/LAMA5/ITGA5/CD44/TNC/LAMA4/ITGB4/COL4A1/THBS3/COL11A1/ITGAV/RELN/HSPG2/ITGA7/LAMA1 |
| KEGG_PATHOGENIC_ESCHERICHIA_COLI_INFECTION | KEGG_PATHOGENIC_ESCHERICHIA_COLI_INFECTION | KEGG_PATHOGENIC_ESCHERICHIA_COLI_INFECTION | 56 | 0.523148512170985 | 1.74450654094222 | 0.000105493289155004 | 0.000672974430816402 | 0.000241236922568611 | 10384 | tags=50%, list=25%, signal=38% | TUBAL3/TUBA3E/TUBA1C/KRT18/ARPC5/TUBB/CDC42/ABL1/ARHGEF2/WASL/OCLN/ACTG1/TUBA1A/ARPC2/RHOA/ACTB/CTNNB1/TUBA1B/YWHAQ/CDH1/ARPC4/ARPC1B/TUBB2B/YWHAZ/ARPC3/NCL/ARPC1A/NCK1 |
| KEGG_ENDOCYTOSIS | KEGG_ENDOCYTOSIS | KEGG_ENDOCYTOSIS | 181 | 0.41870202022874 | 1.52076214120226 | 0.000103735872739611 | 0.000672974430816402 | 0.000241236922568611 | 14756 | tags=53%, list=35%, signal=34% | PIP5K1B/PLD1/NEDD4/CLTCL1/KIT/STAM/RAB5B/GRK1/ERBB4/GRK4/PDCD6IP/VPS37A/DNM1/VPS37C/RAB31/TFRC/CDC42/GRK2/RNF41/NTRK1/TSG101/AP2M1/ARFGAP3/ARFGAP2/STAMBP/ARRB1/DNM3/CHMP3/VPS36/PSD2/EGFR/DNM1L/SH3KBP1/PRKCI/EHD2/IQSEC2/GIT2/AP2A2/KDR/VPS25/CLTC/RAB11B/DAB2/RAB22A/CLTA/CBL/VTA1/RAB11FIP2/ADRB1/RAB11A/GRK6/ERBB3/CHMP6/HSPA1B/PARD6B/VPS37B/DNM2/HSPA1A/CBLC/VPS28/VPS4A/AP2B1/MVB12B/PDGFRA/GRK3/SH3GL1/RET/PIP4K2B/LDLR/RAB11FIP1/PSD3/EPS15/RAB4A/AP2A1/AGAP2/GIT1/RAB5C/AP2S1/ADRB2/PIKFYVE/CSF1R/HSPA2/ARAP1/CLTB/DNAJC6/SMAP1/SNF8/RUFY1/VPS45/SMURF2/ASAP3/CHMP4B/MVB12A/ACAP1/EPN3/RBSN |
| KEGG_TYPE_I_DIABETES_MELLITUS | KEGG_TYPE_I_DIABETES_MELLITUS | KEGG_TYPE_I_DIABETES_MELLITUS | 40 | -0.446673831 | -2.05444321 | 0.000110393956227183 | 0.000680762730067629 | 0.000244028745344299 | 9255 | tags=62%, list=22%, signal=49% | HLA-DRB1/IL1A/HLA-DOA/HLA-G/HLA-DPB1/HLA-DPA1/HLA-DQA2/IL12A/LTA/HLA-DRA/CD80/FASLG/HLA-DMB/HLA-DMA/HLA-DQB1/HLA-A/IFNG/HLA-F/GZMB/HLA-E/PRF1/HLA-C/HLA-B/HLA-DOB/FAS |
| KEGG_RIBOSOME | KEGG_RIBOSOME | KEGG_RIBOSOME | 88 | 0.484941847642346 | 1.68391853130589 | 0.000160952174541467 | 0.000960521041618433 | 0.000344311952329794 | 18694 | tags=84%, list=45%, signal=47% | RSL24D1/RPL22L1/RPS24/RPL15/RPL6/RPL7/RPL5/MRPL13/RPL7A/RPLP0/RPS23/RPL4/RPL26L1/RPL26/RPL22/RPS3A/RPS7/RPL24/RPL17/RPS2/RPS6/RPL31/RPL36A/RPL27/RPS4Y1/RPS8/RPLP1/RPS5/RPL19/RPL37/RPL35A/RPL10/RPL29/RPS13/RPS3/RPL37A/RPL10A/RPSA/RPL12/RPL8/RPL34/RPS11/RPL3/RPL39/RPS21/RPL23/RPL27A/RPS4X/RPS17/RPS15A/RPS18/RPL11/RPL21/RPL18/RPL35/RPL14/RPS25/RPS26/RPL36AL/RPS20/RPL32/RPS29/RPL18A/RPL30/RPL13/RPL13A/RPL38/RPS28/RPS10/RPS27A/RPS16/RPL23A/RPL36/RPS15 |
| KEGG_LYSINE_DEGRADATION | KEGG_LYSINE_DEGRADATION | KEGG_LYSINE_DEGRADATION | 44 | 0.557292583116841 | 1.79885563882325 | 0.000184898466134937 | 0.0010689442573426 | 0.000383177742319112 | 10724 | tags=59%, list=26%, signal=44% | AADAT/KMT5A/SETMAR/TMLHE/HADHA/ACAT2/GCDH/ALDH7A1/OGDH/ALDH3A2/HADH/ALDH2/AASDHPPT/KMT5B/SETDB1/SUV39H1/ECHS1/NSD2/ALDH9A1/DOT1L/PIPOX/PLOD3/AASDH/DLST/EHMT2/SETD7 |
| KEGG_PANCREATIC_CANCER | KEGG_PANCREATIC_CANCER | KEGG_PANCREATIC_CANCER | 70 | 0.501489218737695 | 1.70462038532567 | 0.000202177894520007 | 0.00113342152988489 | 0.000406290505733985 | 13143 | tags=54%, list=31%, signal=37% | PLD1/PIK3CB/ARHGEF6/E2F3/CDK6/VEGFA/RALBP1/CDC42/CCND1/MAPK1/MAP2K1/PIK3CG/RALA/CDK4/EGFR/E2F2/MAPK8/CDKN2A/TGFB2/CASP9/TGFBR1/SMAD2/ARAF/PIK3R1/PIK3R3/AKT2/RAC3/AKT1/RAC1/PIK3R2/KRAS/RAD51/TGFBR2/STAT3/RAC2/PIK3CA/BAD/MAPK9 |
| KEGG_PYRUVATE_METABOLISM | KEGG_PYRUVATE_METABOLISM | KEGG_PYRUVATE_METABOLISM | 39 | 0.560732020528914 | 1.77652397305098 | 0.000309548510341204 | 0.00163618498323208 | 0.000586512966962282 | 13172 | tags=67%, list=32%, signal=46% | PDHA1/ME3/LDHA/ACACA/ACAT2/ME2/DLD/ACSS2/PDHB/PC/ALDH7A1/ALDH3A2/ME1/ALDH2/PKM/PKLR/MDH2/ALDH9A1/LDHC/DLAT/GLO1/ACSS1/MDH1/ACYP1/HAGHL/LDHD |
| KEGG_WNT_SIGNALING_PATHWAY | KEGG_WNT_SIGNALING_PATHWAY | KEGG_WNT_SIGNALING_PATHWAY | 147 | 0.425701152363981 | 1.53200334183156 | 0.000300884605582163 | 0.00163618498323208 | 0.000586512966962282 | 11400 | tags=44%, list=27%, signal=32% | VANGL2/NLK/CHP1/PLCB4/NKD2/CTNNBIP1/FZD6/CSNK2A1/FZD9/PLCB1/FZD10/TBL1Y/FZD1/CCND1/VANGL1/DVL3/PPP2CB/FZD8/PPP3CB/PRKACA/SIAH1/WNT11/FZD7/SFRP1/RHOA/PPP3R1/CTBP2/MAPK8/CAMK2G/PLCB3/SMAD2/MMP7/PPP3CA/BTRC/CTNNB1/WNT7B/CSNK1A1L/PSEN1/PPP2CA/CSNK2B/FBXW11/SENP2/WIF1/CCND3/RBX1/RAC3/DAAM1/FRAT1/PPP2R5D/PLCB2/LRP5/SKP1/RAC1/FRAT2/APC2/PPP2R1A/DVL2/WNT5B/DKK1/PRKACG/CCND2/TCF7L2/MAP3K7/PPP2R5A/CSNK1A1 |
| KEGG_ARRHYTHMOGENIC_RIGHT_VENTRICULAR_CARDIOMYOPATHY_ARVC | KEGG_ARRHYTHMOGENIC_RIGHT_VENTRICULAR_CARDIOMYOPATHY_ARVC | KEGG_ARRHYTHMOGENIC_RIGHT_VENTRICULAR_CARDIOMYOPATHY_ARVC | 74 | 0.481185526238452 | 1.65082446738196 | 0.000362952220151299 | 0.00186517113133307 | 0.000668596195015551 | 9919 | tags=36%, list=24%, signal=28% | ITGA9/CACNA2D4/GJA1/PKP2/ACTN4/CDH2/CACNA1C/CTNNA1/JUP/DSP/ITGA4/ITGA5/ACTG1/DSG2/ITGB4/ACTB/ACTN1/CTNNA2/CACNA2D3/ITGAV/CTNNB1/ITGA7/CACNG5/CACNA1D/CACNG4/CACNB4/CACNG7 |
| KEGG_ARACHIDONIC_ACID_METABOLISM | KEGG_ARACHIDONIC_ACID_METABOLISM | KEGG_ARACHIDONIC_ACID_METABOLISM | 52 | 0.533652805383904 | 1.76333804493466 | 0.000386526048026034 | 0.00193263024013017 | 0.000692777838567287 | 8925 | tags=38%, list=21%, signal=30% | PLA2G4A/CYP4F2/AKR1C3/HPGDS/CYP2E1/LTC4S/GGT1/GGT5/PTGES/LTA4H/TBXAS1/GPX7/PLA2G4E/CYP2C18/PLA2G5/ALOX12B/ALOX5/PTGES2/CYP2C8/CYP4F3 |
| KEGG_RNA_POLYMERASE | KEGG_RNA_POLYMERASE | KEGG_RNA_POLYMERASE | 29 | 0.599419820690889 | 1.80580576058834 | 0.000407999717360814 | 0.00193538327466027 | 0.000693764701585189 | 14634 | tags=79%, list=35%, signal=52% | POLR3B/POLR2K/POLR2J3/POLR2J2/POLR3C/POLR3F/POLR2L/POLR2C/POLR2D/POLR2B/POLR1D/POLR2G/POLR1A/POLR2E/POLR2A/POLR1H/POLR2F/POLR2J/POLR3A/POLR1C/POLR3D/POLR2I/POLR3H |
| KEGG_BUTANOATE_METABOLISM | KEGG_BUTANOATE_METABOLISM | KEGG_BUTANOATE_METABOLISM | 33 | 0.569338217288216 | 1.7357925054447 | 0.000406644236572239 | 0.00193538327466027 | 0.000693764701585189 | 9778 | tags=52%, list=23%, signal=39% | PDHA1/GAD1/HADHA/ACAT2/BDH1/AACS/BDH2/PDHB/ALDH7A1/ALDH3A2/HADH/ALDH2/ECHS1/ALDH9A1/ALDH5A1/HMGCL/ACADS |
| KEGG_PROPANOATE_METABOLISM | KEGG_PROPANOATE_METABOLISM | KEGG_PROPANOATE_METABOLISM | 33 | 0.568178854235388 | 1.73225785128469 | 0.000436952747547222 | 0.0020209064574059 | 0.000724421660407237 | 10839 | tags=61%, list=26%, signal=45% | LDHA/ACACA/PCCB/HADHA/ACAT2/ACADM/MLYCD/ACSS2/MMUT/ALDH7A1/SUCLG1/ALDH3A2/ALDH2/ECHS1/ALDH9A1/MCEE/LDHC/HIBCH/ACSS1/SUCLA2 |
| KEGG_LIMONENE_AND_PINENE_DEGRADATION | KEGG_LIMONENE_AND_PINENE_DEGRADATION | KEGG_LIMONENE_AND_PINENE_DEGRADATION | 10 | 0.756026689973766 | 1.83431038171561 | 0.000468367022480526 | 0.00211336339411945 | 0.000757564118517924 | 8154 | tags=80%, list=20%, signal=64% | NAA80/HADHA/YOD1/ALDH7A1/ALDH3A2/ALDH2/ECHS1/ALDH9A1 |
| KEGG_UBIQUITIN_MEDIATED_PROTEOLYSIS | KEGG_UBIQUITIN_MEDIATED_PROTEOLYSIS | KEGG_UBIQUITIN_MEDIATED_PROTEOLYSIS | 133 | 0.427521257824882 | 1.53117250353837 | 0.000530880891260098 | 0.00233840392578853 | 0.000838232986200155 | 15073 | tags=56%, list=36%, signal=36% | NEDD4/KLHL13/UBE4B/UBE2E3/UBE2E1/MGRN1/ANAPC7/CUL7/RCHY1/DDB1/KLHL9/ERCC8/UBE2K/SIAH1/UBE2H/UBE3C/ELOC/CDC23/SKP2/UBE2I/DET1/KEAP1/HERC2/NHLRC1/UBA2/BTRC/PRPF19/UBE3A/CDC16/CBL/RNF7/FBXW11/FANCL/UBE2E2/UBE2G1/UBA3/RBX1/ANAPC1/UBE2Q2/UBE2A/CBLC/ANAPC11/ANAPC5/SKP1/ANAPC10/UBE2D2/UBA6/FBXO4/UBE2G2/MID1/COP1/PIAS4/UBE2J1/UBE2J2/UBE2W/ELOB/UBE2D4/UBE2F/BIRC2/STUB1/UBE2N/CUL5/PRKN/CUL4B/SAE1/UBE2C/SMURF2/CUL3/UBE2Q1/TRIP12/FBXW8/UBE2M/CUL4A/UBE2L3/CDC27 |
| KEGG_CYSTEINE_AND_METHIONINE_METABOLISM | KEGG_CYSTEINE_AND_METHIONINE_METABOLISM | KEGG_CYSTEINE_AND_METHIONINE_METABOLISM | 34 | 0.562843966303234 | 1.7225438114741 | 0.000553334651980042 | 0.00238062582828623 | 0.000853368006725646 | 6360 | tags=38%, list=15%, signal=32% | AHCYL1/DNMT3B/MAT2A/LDHA/APIP/ADI1/AHCYL2/MPST/AHCY/AMD1/SMS/MTAP/DNMT3A |
| KEGG_FATTY_ACID_METABOLISM | KEGG_FATTY_ACID_METABOLISM | KEGG_FATTY_ACID_METABOLISM | 40 | 0.548212115588126 | 1.7393034935065 | 0.000591649061848984 | 0.00248761537368323 | 0.000891719877906364 | 9778 | tags=52%, list=23%, signal=40% | HADHB/ADH6/HADHA/ACAT2/ACADM/GCDH/ADH1A/ACADVL/ALDH7A1/CPT2/ALDH3A2/HADH/ALDH2/CPT1A/ACAA1/ECHS1/ALDH9A1/ADH5/ECI1/ACOX3/ACADS |
| KEGG_VASOPRESSIN_REGULATED_WATER_REABSORPTION | KEGG_VASOPRESSIN_REGULATED_WATER_REABSORPTION | KEGG_VASOPRESSIN_REGULATED_WATER_REABSORPTION | 44 | 0.534686005905326 | 1.72588504828715 | 0.000746916337994337 | 0.00300390266367288 | 0.0010767901440193 | 7964 | tags=43%, list=19%, signal=35% | DYNC1I2/CREB3L4/ADCY6/RAB5B/ARHGDIB/DYNC2LI1/DCTN4/PRKACA/NSF/DCTN1/DCTN2/DYNC1LI1/DYNC1LI2/DCTN6/AQP4/RAB11B/DYNLL1/DCTN5/RAB11A |
| KEGG_METABOLISM_OF_XENOBIOTICS_BY_CYTOCHROME_P450 | KEGG_METABOLISM_OF_XENOBIOTICS_BY_CYTOCHROME_P450 | KEGG_METABOLISM_OF_XENOBIOTICS_BY_CYTOCHROME_P450 | 60 | 0.489652304223857 | 1.64320125924402 | 0.000732102757178165 | 0.00300390266367288 | 0.0010767901440193 | 6546 | tags=33%, list=16%, signal=28% | MGST1/ALDH3B2/AKR1C3/CYP2E1/UGT2B11/UGT2B28/UGT2A1/GSTA4/ADH6/CYP2S1/EPHX1/GSTM2/ALDH3B1/MGST2/GSTT2/GSTZ1/ADH1A/CYP2C18/CYP1B1/GSTP1 |
| KEGG_FC_GAMMA_R_MEDIATED_PHAGOCYTOSIS | KEGG_FC_GAMMA_R_MEDIATED_PHAGOCYTOSIS | KEGG_FC_GAMMA_R_MEDIATED_PHAGOCYTOSIS | 96 | 0.455881228455346 | 1.60110097586746 | 0.000793386250566958 | 0.00312290332669973 | 0.00111944756518966 | 10672 | tags=42%, list=26%, signal=31% | PIP5K1B/PLD1/PLA2G4A/PIK3CB/MARCKSL1/WASF1/WASF2/CRKL/DNM1/GSN/ARPC5/CDC42/SYK/MAPK1/MAP2K1/WASL/PIK3CG/DNM3/DOCK2/DNM1L/ARPC2/PLA2G4D/PLA2G4E/PIK3R1/MYO10/PIK3R3/AKT2/PRKCD/ARPC4/ARPC1B/ARPC3/DNM2/CFL1/VAV3/AKT1/ARPC1A/RAC1/PIP4K2B/PIK3R2/HCK |
| KEGG_SPHINGOLIPID_METABOLISM | KEGG_SPHINGOLIPID_METABOLISM | KEGG_SPHINGOLIPID_METABOLISM | 37 | 0.556100988228896 | 1.73958030394509 | 0.000865757129425353 | 0.00333677226966022 | 0.00119611182354819 | 6483 | tags=38%, list=16%, signal=32% | SPTLC2/B4GALT6/UGCG/NEU3/SGMS2/ACER3/SPTLC1/GALC/DEGS2/GLA/ASAH1/DEGS1/GLB1/NEU4 |
| KEGG_GLIOMA | KEGG_GLIOMA | KEGG_GLIOMA | 65 | 0.489579171645002 | 1.65497076248665 | 0.000918704817609939 | 0.00339920782515678 | 0.0012184927054616 | 10627 | tags=43%, list=25%, signal=32% | PIK3CB/E2F3/SHC4/CDK6/CALM2/CCND1/MAPK1/MAP2K1/PIK3CG/CDK4/EGFR/E2F2/CALML5/SHC1/CDKN2A/CAMK2G/PDGFA/ARAF/PIK3R1/NRAS/PIK3R3/AKT2/CALM1/PDGFRA/AKT1/PIK3R2/GRB2/KRAS |
| KEGG_COLORECTAL_CANCER | KEGG_COLORECTAL_CANCER | KEGG_COLORECTAL_CANCER | 62 | 0.476662356641924 | 1.60599504441543 | 0.000910129744454865 | 0.00339920782515678 | 0.0012184927054616 | 13143 | tags=58%, list=31%, signal=40% | PIK3CB/MSH3/APPL1/CASP3/CCND1/MAPK1/MAP2K1/PIK3CG/MSH6/RHOA/MAPK8/TGFB2/CASP9/TGFBR1/SMAD2/CTNNB1/ARAF/PIK3R1/PIK3R3/AKT2/MLH1/RAC3/DCC/AKT1/RAC1/PIK3R2/APC2/KRAS/TGFBR2/TCF7L2/CYCS/RAC2/BIRC5/PIK3CA/BAD/MAPK9 |
| KEGG_GLYCOSYLPHOSPHATIDYLINOSITOL_GPI_ANCHOR_BIOSYNTHESIS | KEGG_GLYCOSYLPHOSPHATIDYLINOSITOL_GPI_ANCHOR_BIOSYNTHESIS | KEGG_GLYCOSYLPHOSPHATIDYLINOSITOL_GPI_ANCHOR_BIOSYNTHESIS | 25 | 0.602675957358344 | 1.77971493410103 | 0.00101577348341062 | 0.00368466851825422 | 0.00132082000938843 | 10180 | tags=68%, list=24%, signal=51% | PIGN/PIGF/PIGP/PIGM/PIGL/PIGS/PIGV/PIGK/PIGZ/PIGY/PIGO/PIGT/GPAA1/PIGU/PIGC/PIGW/PIGX |
| KEGG_VALINE_LEUCINE_AND_ISOLEUCINE_BIOSYNTHESIS | KEGG_VALINE_LEUCINE_AND_ISOLEUCINE_BIOSYNTHESIS | KEGG_VALINE_LEUCINE_AND_ISOLEUCINE_BIOSYNTHESIS | 10 | 0.733491906540367 | 1.77963534477606 | 0.00118692905302622 | 0.00414374222990047 | 0.00148538128297997 | 10495 | tags=80%, list=25%, signal=60% | PDHA1/LARS1/PDHB/VARS1/IARS1/LARS2/IARS2/BCAT1 |
| KEGG_PROTEIN_EXPORT | KEGG_PROTEIN_EXPORT | KEGG_PROTEIN_EXPORT | 24 | 0.605798815917356 | 1.76928670739434 | 0.00118712615234986 | 0.00414374222990047 | 0.00148538128297997 | 9953 | tags=62%, list=24%, signal=48% | SRP9/SEC11A/SEC63/SRP72/SRP9P1/SEC62/SEC61A1/IMMP1L/SPCS1/SRPRA/OXA1L/SRP14/SRP54/SEC61B/SRP68 |
| KEGG_BETA_ALANINE_METABOLISM | KEGG_BETA_ALANINE_METABOLISM | KEGG_BETA_ALANINE_METABOLISM | 22 | 0.609621332344107 | 1.7444800491632 | 0.00129178986345047 | 0.00434511135887885 | 0.0015575648114331 | 9852 | tags=59%, list=24%, signal=45% | GAD1/HADHA/ACADM/MLYCD/SMS/ALDH7A1/ALDH3A2/ALDH2/DPYD/ECHS1/ALDH9A1/HIBCH/CNDP1 |
| KEGG_STARCH_AND_SUCROSE_METABOLISM | KEGG_STARCH_AND_SUCROSE_METABOLISM | KEGG_STARCH_AND_SUCROSE_METABOLISM | 45 | 0.52829259771944 | 1.70769496339408 | 0.00128947230460909 | 0.00434511135887885 | 0.0015575648114331 | 11315 | tags=49%, list=27%, signal=36% | GBE1/HK2/PGM2/UGT2B11/GPI/UGT2B28/UGT2A1/PYGL/GUSB/GYS1/HK3/GANC/G6PC2/PGM2L1/PGM1/AGL/UXS1/UGP2/ENPP1/UGDH/HK1/PYGB |
| KEGG_ALANINE_ASPARTATE_AND_GLUTAMATE_METABOLISM | KEGG_ALANINE_ASPARTATE_AND_GLUTAMATE_METABOLISM | KEGG_ALANINE_ASPARTATE_AND_GLUTAMATE_METABOLISM | 31 | 0.561179551457048 | 1.69996271000742 | 0.001487672935951 | 0.0049146338062667 | 0.00176171795046829 | 9378 | tags=45%, list=22%, signal=35% | GAD1/ADSS2/ALDH4A1/ACY3/GLUD2/DDO/GFPT2/ASL/GLUD1/ADSS1/ADSL/GPT2/ALDH5A1/NIT2 |
| KEGG_TIGHT_JUNCTION | KEGG_TIGHT_JUNCTION | KEGG_TIGHT_JUNCTION | 128 | 0.419617611233914 | 1.49988167454524 | 0.00153091017130873 | 0.00496874353845817 | 0.00178111432673038 | 8891 | tags=32%, list=21%, signal=25% | F11R/CLDN10/GNAI1/AMOTL1/EXOC4/ACTN4/CSNK2A1/RAB13/CTNNA1/CDC42/VAPA/MYH14/PPP2CB/YES1/OCLN/CLDN3/ACTG1/CDK4/GNAI3/CLDN23/RHOA/CLDN4/PRKCI/ACTB/TJP1/ACTN1/MYH9/CTNNA2/PALS1/CTNNB1/NRAS/GNAI2/CLDN22/PPP2CA/AKT2/PRKCD/CSNK2B/PARD6B/MYL12B/MAP3K20/EXOC3 |
| KEGG_AMINOACYL_TRNA_BIOSYNTHESIS | KEGG_AMINOACYL_TRNA_BIOSYNTHESIS | KEGG_AMINOACYL_TRNA_BIOSYNTHESIS | 41 | 0.531615892632459 | 1.69659719935722 | 0.00162353236278282 | 0.00509263821925741 | 0.00182552607575088 | 15683 | tags=73%, list=38%, signal=46% | TARS1/QARS1/NARS1/NARS2/LARS1/WARS2/YARS2/VARS1/IARS1/LARS2/IARS2/MARS1/FARSA/MTFMT/PARS2/RARS2/TARS2/HARS2/GARS1/YARS1/EPRS1/DARS1/SARS1/HARS1/DARS2/SEPSECS/RARS1/TARS3/SARS2/FARSB |
| KEGG_GLUTATHIONE_METABOLISM | KEGG_GLUTATHIONE_METABOLISM | KEGG_GLUTATHIONE_METABOLISM | 45 | 0.523639396461928 | 1.6926535859729 | 0.00162413867533074 | 0.00509263821925741 | 0.00182552607575088 | 9759 | tags=51%, list=23%, signal=39% | MGST1/IDH1/GSTA4/GGT1/GGT5/GCLC/GSTM2/MGST2/GSTT2/GSTZ1/PGD/GPX7/SMS/IDH2/GSS/GSTP1/G6PD/ANPEP/GCLM/OPLAH/GSR/GSTM1/GSTO1 |
| KEGG_GLYOXYLATE_AND_DICARBOXYLATE_METABOLISM | KEGG_GLYOXYLATE_AND_DICARBOXYLATE_METABOLISM | KEGG_GLYOXYLATE_AND_DICARBOXYLATE_METABOLISM | 15 | 0.671495435352508 | 1.77423177516858 | 0.00192836341461465 | 0.00594578719506185 | 0.00213134903720567 | 8256 | tags=60%, list=20%, signal=48% | CS/MTHFD2L/AFMID/MTHFD1L/GLYCTK/PGP/HYI/MDH2/ACO2 |
| KEGG_CELL_CYCLE | KEGG_CELL_CYCLE | KEGG_CELL_CYCLE | 124 | 0.417444536793223 | 1.49181863807793 | 0.00203061637987619 | 0.00615842672585402 | 0.00220757259589646 | 16807 | tags=60%, list=40%, signal=36% | YWHAG/CCNA1/GADD45A/E2F3/YWHAH/CDK6/HDAC2/CDKN2C/TFDP2/TFDP1/ANAPC7/YWHAE/ABL1/CCND1/YWHAB/CHEK2/CDK4/CDC23/E2F2/SKP2/CDKN1C/CDKN2A/TGFB2/WEE2/MCM7/CCNH/SMAD2/CDK2/CDK7/CDC16/YWHAQ/CCND3/YWHAZ/RBX1/ANAPC1/ANAPC11/CDKN1B/E2F4/ANAPC5/SKP1/HDAC1/ANAPC10/ORC5/CDC25A/CCND2/ORC2/BUB1/PKMYT1/PLK1/ORC4/CCNA2/CDC25C/TP53/ORC1/ZBTB17/PTTG1/CCNE2/CDC45/ORC6/PCNA/CCNB3/CDC27/CCNB2/ORC3/E2F1/MAD2L2/DBF4/ANAPC2/BUB1B/ANAPC13/RBL2/SMC3/SMC1A/CDC20 |
| KEGG_BIOSYNTHESIS_OF_UNSATURATED_FATTY_ACIDS | KEGG_BIOSYNTHESIS_OF_UNSATURATED_FATTY_ACIDS | KEGG_BIOSYNTHESIS_OF_UNSATURATED_FATTY_ACIDS | 22 | 0.594791727048747 | 1.70204395120835 | 0.00221011529901404 | 0.00659469887609027 | 0.00236396033680619 | 11471 | tags=68%, list=27%, signal=49% | HSD17B12/FADS1/FADS2/HACD1/PECR/HADHA/SCD/YOD1/ELOVL5/ACAA1/ACOT1/ACOX3/ACOT2/ELOVL2/TECR |
| KEGG_SMALL_CELL_LUNG_CANCER | KEGG_SMALL_CELL_LUNG_CANCER | KEGG_SMALL_CELL_LUNG_CANCER | 84 | 0.44595493998668 | 1.54457883570371 | 0.00225317486230749 | 0.00661646586550611 | 0.00237176301295525 | 13041 | tags=45%, list=31%, signal=31% | PIK3CB/LAMB2/E2F3/CDK6/LAMB4/LAMB1/COL4A2/LAMA3/CCND1/PIK3CG/LAMA5/CDK4/E2F2/SKP2/LAMA4/COL4A1/RXRG/CASP9/CDK2/MAX/ITGAV/PIK3R1/PIK3R3/LAMA1/APAF1/AKT2/RXRA/CKS1B/CDKN1B/AKT1/PIK3R2/RXRB/PIAS4/LAMC2/CYCS/NOS2/BIRC2/PIK3CA |
| KEGG_PENTOSE_PHOSPHATE_PATHWAY | KEGG_PENTOSE_PHOSPHATE_PATHWAY | KEGG_PENTOSE_PHOSPHATE_PATHWAY | 26 | 0.58467196648606 | 1.74023240413659 | 0.00257073821346613 | 0.00743104014830052 | 0.00266375834619023 | 10836 | tags=65%, list=26%, signal=48% | PGM2/GPI/RPEL1/H6PD/DERA/RPE/PGD/PGM1/G6PD/PFKL/TKT/TALDO1/RBKS/RPIA/PGLS/ALDOA/FBP1 |
| KEGG_NICOTINATE_AND_NICOTINAMIDE_METABOLISM | KEGG_NICOTINATE_AND_NICOTINAMIDE_METABOLISM | KEGG_NICOTINATE_AND_NICOTINAMIDE_METABOLISM | 24 | 0.583247198900886 | 1.70342280147523 | 0.00263934845868294 | 0.00751199176702067 | 0.00269277656513401 | 10218 | tags=54%, list=24%, signal=41% | NT5E/QPRT/NMNAT3/NUDT12/BST1/NMNAT2/NADSYN1/PNP/NNMT/NMNAT1/NNT/NT5C2/ENPP1 |
| KEGG_MTOR_SIGNALING_PATHWAY | KEGG_MTOR_SIGNALING_PATHWAY | KEGG_MTOR_SIGNALING_PATHWAY | 51 | 0.497126641080077 | 1.63459383769542 | 0.00284132763871689 | 0.00784545691287501 | 0.00281231172410313 | 14039 | tags=59%, list=34%, signal=39% | RPS6KA2/PIK3CB/VEGFA/RHEB/PRKAA1/EIF4E/MAPK1/PIK3CG/ULK2/EIF4B/EIF4EBP1/PIK3R1/EIF4E2/PIK3R3/STK11/AKT2/STRADA/CAB39/MLST8/AKT1/PIK3R2/RPS6/CAB39L/MTOR/RPS6KB2/PIK3CA/PDPK1/RPS6KA1/ULK1/TSC2 |
| KEGG_AXON_GUIDANCE | KEGG_AXON_GUIDANCE | KEGG_AXON_GUIDANCE | 128 | 0.412010425942592 | 1.47269054264834 | 0.00281586968838323 | 0.00784545691287501 | 0.00281231172410313 | 11052 | tags=39%, list=26%, signal=29% | GNAI1/CHP1/NTN1/SRGAP3/SEMA6A/EPHA5/EFNB2/EFNA4/CDC42/ABL1/PLXNB1/SEMA3C/MAPK1/DPYSL5/PPP3CB/GNAI3/RHOA/PPP3R1/EFNA2/EFNA1/FES/SEMA5B/UNC5B/PPP3CA/SEMA5A/ROBO2/EFNB3/EPHA2/PAK4/NRAS/SRGAP1/GNAI2/DPYSL2/CDK5/SEMA4A/RAC3/CFL1/PLXNB2/DCC/EPHB3/EPHA7/RAC1/NCK1/PAK5/KRAS/EPHB2/RASA1/EPHB6/ROBO1/EFNB1 |
| KEGG_ERBB_SIGNALING_PATHWAY | KEGG_ERBB_SIGNALING_PATHWAY | KEGG_ERBB_SIGNALING_PATHWAY | 87 | 0.443739815714681 | 1.54117632454868 | 0.00292880403903321 | 0.00796806981207564 | 0.00285626400091474 | 13179 | tags=46%, list=32%, signal=32% | PIK3CB/SHC4/ERBB4/CRKL/NRG1/STAT5A/ABL1/MAPK1/MAP2K1/PIK3CG/EGFR/MAPK8/SHC1/CAMK2G/EIF4EBP1/ELK1/ARAF/PIK3R1/PAK4/NRAS/CBL/PIK3R3/HBEGF/AKT2/ERBB3/CBLC/CDKN1B/MAP2K7/AKT1/NCK1/PIK3R2/GRB2/PAK5/KRAS/MTOR/RPS6KB2/PIK3CA/BAD/MAPK9/PAK2 |
| KEGG_NEUROTROPHIN_SIGNALING_PATHWAY | KEGG_NEUROTROPHIN_SIGNALING_PATHWAY | KEGG_NEUROTROPHIN_SIGNALING_PATHWAY | 124 | 0.411554727800859 | 1.47077026864178 | 0.00326013592386356 | 0.00874094414369216 | 0.00313331141423958 | 13143 | tags=44%, list=31%, signal=30% | RPS6KA2/PIK3CB/YWHAG/SHC4/YWHAH/CRKL/ARHGDIB/CDC42/YWHAE/ABL1/NTRK1/SORT1/CALM2/MAPK1/MAP2K1/PIK3CG/YWHAB/MAGED1/RHOA/CALML5/MAPK8/SHC1/BDNF/CAMK2G/SH2B3/IRAK1/PIK3R1/NTRK2/NRAS/PIK3R3/YWHAQ/PSEN1/AKT2/PRKCD/MAP2K5/ATF4/YWHAZ/MAP3K3/CALM1/MAP2K7/RAP1B/AKT1/RAC1/PIK3R2/GRB2/KRAS/NTRK3/PTPN11/RPS6KA4/MAPK14/PRDM4/IRAK3/PIK3CA/BAD/MAPK9 |
| KEGG_SELENOAMINO_ACID_METABOLISM | KEGG_SELENOAMINO_ACID_METABOLISM | KEGG_SELENOAMINO_ACID_METABOLISM | 26 | 0.578050867357828 | 1.7205251975072 | 0.00353213420626969 | 0.0093349261165699 | 0.00334623240593971 | 7720 | tags=42%, list=18%, signal=35% | AHCYL1/MAT2A/PAPSS1/PAPSS2/AHCYL2/GGT1/GGT5/AHCY/SEPHS1/LCMT1/MARS1 |
| KEGG_MELANOMA | KEGG_MELANOMA | KEGG_MELANOMA | 67 | 0.461194012129788 | 1.56072460977042 | 0.00363051701232935 | 0.00945979784902718 | 0.0033909943925389 | 10627 | tags=43%, list=25%, signal=32% | PDGFC/PIK3CB/E2F3/HGF/CDK6/FGF20/FGF16/MITF/CCND1/MAPK1/MAP2K1/PIK3CG/CDK4/EGFR/E2F2/CDKN2A/FGF18/PDGFA/ARAF/PIK3R1/NRAS/PIK3R3/AKT2/CDH1/PDGFRA/AKT1/PIK3R2/FGF1/KRAS |
| KEGG_OTHER_GLYCAN_DEGRADATION | KEGG_OTHER_GLYCAN_DEGRADATION | KEGG_OTHER_GLYCAN_DEGRADATION | 14 | 0.65540009587901 | 1.70345459926271 | 0.00382403187026175 | 0.00982563744442256 | 0.00352213461734635 | 12252 | tags=86%, list=29%, signal=61% | FUCA2/HEXA/NEU3/AGA/GLB1/NEU4/HEXB/MAN2B1/FUCA1/MANBA/MAN2C1/ENGASE |
| KEGG_GLYCOSAMINOGLYCAN_BIOSYNTHESIS_HEPARAN_SULFATE | KEGG_GLYCOSAMINOGLYCAN_BIOSYNTHESIS_HEPARAN_SULFATE | KEGG_GLYCOSAMINOGLYCAN_BIOSYNTHESIS_HEPARAN_SULFATE | 25 | 0.570738599617465 | 1.68540323669019 | 0.00397706636350624 | 0.0100788668116254 | 0.00361290816007056 | 7364 | tags=48%, list=18%, signal=40% | GLCE/HS3ST5/NDST3/XYLT1/EXT2/EXTL2/HS6ST1/B3GALT6/NDST2/HS2ST1/NDST1/EXT1 |
| KEGG_CARDIAC_MUSCLE_CONTRACTION | KEGG_CARDIAC_MUSCLE_CONTRACTION | KEGG_CARDIAC_MUSCLE_CONTRACTION | 76 | 0.4440877916244 | 1.52683156993132 | 0.0043268202332159 | 0.0107074947057704 | 0.00383824845782951 | 15846 | tags=53%, list=38%, signal=33% | CACNA2D4/TPM4/CACNA1C/MT-CO1/TPM3/COX7A2L/UQCRFS1/TNNI3/UQCRC2/SLC9A6/CACNA2D3/MT-CO2/MT-CO3/UQCRC1/CACNG5/COX5A/UQCRH/CACNA1D/MT-CYB/CACNG4/UQCRQ/CACNB4/CACNG7/UQCRB/COX6B1/CYC1/COX7C/COX8A/COX7B/COX4I1/TPM1/ATP2A2/COX7A2/UQCR11/UQCR10/COX6A1/ATP1A1/COX6C/UQCRHL/COX5B |
| KEGG_OOCYTE_MEIOSIS | KEGG_OOCYTE_MEIOSIS | KEGG_OOCYTE_MEIOSIS | 109 | 0.406562006330769 | 1.4369363577831 | 0.00434087623206909 | 0.0107074947057704 | 0.00383824845782951 | 16807 | tags=61%, list=40%, signal=37% | RPS6KA2/YWHAG/CHP1/ADCY6/YWHAH/ANAPC7/YWHAE/CALM2/MAPK1/PPP2CB/MAP2K1/YWHAB/PPP3CB/PRKACA/CDC23/PPP3R1/CALML5/CAMK2G/CDK2/PPP3CA/BTRC/CDC16/YWHAQ/PPP2CA/FBXW11/YWHAZ/RBX1/ANAPC1/ANAPC11/AURKA/CALM1/ADCY3/ANAPC5/PPP2R5D/SKP1/ANAPC10/FBXO43/PPP2R1A/PRKACG/PPP2R5A/BUB1/PKMYT1/FBXO5/PPP1CB/PPP1CA/PLK1/PPP1CC/CDC25C/RPS6KA1/ADCY2/ADCY8/PTTG1/CCNE2/PPP2R1B/AR/CDC27/CCNB2/MAD2L2/SPDYC/RPS6KA3/ANAPC2/PPP2R5B/MAPK12/ANAPC13/SMC3/SMC1A/CDC20 |
| KEGG_NON_SMALL_CELL_LUNG_CANCER | KEGG_NON_SMALL_CELL_LUNG_CANCER | KEGG_NON_SMALL_CELL_LUNG_CANCER | 54 | 0.475795616656035 | 1.58291342905573 | 0.00446774944135712 | 0.010875442719093 | 0.00389845172860801 | 11032 | tags=46%, list=26%, signal=34% | PIK3CB/E2F3/CDK6/CCND1/MAPK1/MAP2K1/PIK3CG/CDK4/EGFR/E2F2/RXRG/CDKN2A/CASP9/ARAF/PIK3R1/NRAS/PIK3R3/RASSF1/AKT2/RXRA/AKT1/PIK3R2/GRB2/KRAS/RXRB |
| KEGG_LEUKOCYTE_TRANSENDOTHELIAL_MIGRATION | KEGG_LEUKOCYTE_TRANSENDOTHELIAL_MIGRATION | KEGG_LEUKOCYTE_TRANSENDOTHELIAL_MIGRATION | 112 | 0.410605252609314 | 1.45579354172603 | 0.00492981051469087 | 0.0118443499378936 | 0.00424576982126486 | 13048 | tags=46%, list=31%, signal=31% | CTNND1/PIK3CB/F11R/CLDN10/GNAI1/PECAM1/ACTN4/MSN/RAPGEF3/CTNNA1/CD99/VCL/CYBB/CDC42/RAPGEF4/ITGA4/PIK3CG/ITGAM/OCLN/CLDN3/ACTG1/GNAI3/CLDN23/RHOA/CLDN4/ACTB/ACTN1/CTNNA2/CTNNB1/PIK3R1/THY1/PIK3R3/GNAI2/CLDN22/MMP9/MYL12B/VAV3/ITGB2/RAP1B/RAC1/PIK3R2/NCF4/CLDN19/CLDN8/PTPN11/ARHGAP35/MAPK14/RAC2/CLDN11/PIK3CA/PTK2B |
| KEGG_NUCLEOTIDE_EXCISION_REPAIR | KEGG_NUCLEOTIDE_EXCISION_REPAIR | KEGG_NUCLEOTIDE_EXCISION_REPAIR | 44 | 0.493232677727851 | 1.59208001409309 | 0.00511304332311285 | 0.0121270899330241 | 0.0043471218536587 | 18064 | tags=80%, list=43%, signal=45% | CETN2/RAD23B/DDB1/POLE/ERCC8/POLE3/CCNH/CDK7/GTF2H4/POLD2/ERCC1/RFC3/RBX1/RFC2/GTF2H1/GTF2H5/POLD1/RAD23A/POLD3/RFC5/XPA/CUL4B/ERCC3/PCNA/CUL4A/POLD4/POLE4/RPA1/GTF2H3/MNAT1/RFC4/ERCC5/RPA2/RPA3/ERCC4 |
| KEGG_RENAL_CELL_CARCINOMA | KEGG_RENAL_CELL_CARCINOMA | KEGG_RENAL_CELL_CARCINOMA | 70 | 0.439269347706833 | 1.49312778175882 | 0.00532782954145064 | 0.0124765628502325 | 0.00447239521800654 | 10627 | tags=39%, list=25%, signal=29% | PIK3CB/HGF/ARNT/CRKL/VEGFA/CDC42/MAPK1/MAP2K1/PIK3CG/ELOC/EGLN2/TGFB2/ARAF/PIK3R1/PAK4/NRAS/PIK3R3/AKT2/FH/RBX1/RAP1B/AKT1/RAC1/PIK3R2/GRB2/PAK5/KRAS |
| KEGG_VIBRIO_CHOLERAE_INFECTION | KEGG_VIBRIO_CHOLERAE_INFECTION | KEGG_VIBRIO_CHOLERAE_INFECTION | 54 | 0.469317828435075 | 1.56136262529339 | 0.00571092136433697 | 0.0132065056550292 | 0.0047340532362267 | 14465 | tags=65%, list=35%, signal=42% | ATP6V0A4/KDELR2/PRKACA/ATP6V1H/ATP6AP1/ACTG1/ATP6V1E1/ATP6V0A2/SEC61A1/ATP6V1C1/ATP6V1B1/ATP6V1A/ATP6V1D/ACTB/TJP1/ARF1/ERO1A/ATP6V1G3/KDELR1/SEC61B/ADCY3/SLC12A2/PRKACG/ATP6V1B2/ATP6V0D1/TJP2/ATP6V1C2/ATP6V1F/ATP6V1E2/ATP6V1G2/ATP6V0B/ATP6V0C/KCNQ1/SEC61G/GNAS |
| KEGG_DNA_REPLICATION | KEGG_DNA_REPLICATION | KEGG_DNA_REPLICATION | 36 | 0.505672407312818 | 1.56599404017281 | 0.00611410998164021 | 0.0139643252667091 | 0.00500570407853584 | 19793 | tags=92%, list=47%, signal=48% | POLE/MCM7/POLE3/POLD2/RNASEH2C/RNASEH2A/RFC3/RFC2/FEN1/SSBP1/POLD1/POLA2/RNASEH2B/POLD3/RFC5/RNASEH1/PCNA/POLD4/POLA1/POLE4/RPA1/RFC4/MCM2/RPA2/RPA3/PRIM2/MCM3/MCM6/LIG1/MCM4/PRIM1/RFC1/POLE2 |
| KEGG_INOSITOL_PHOSPHATE_METABOLISM | KEGG_INOSITOL_PHOSPHATE_METABOLISM | KEGG_INOSITOL_PHOSPHATE_METABOLISM | 53 | 0.47129349447772 | 1.56663163958335 | 0.00631931640277215 | 0.0142569943233274 | 0.00511061531931509 | 4533 | tags=25%, list=11%, signal=22% | PIP5K1B/OCRL/PIK3CB/ITPKA/PLCB4/MINPP1/PLCB1/INPP1/PLCD3/INPP5A/PIK3CG/IMPA1/IMPA2 |
| KEGG_PROGESTERONE_MEDIATED_OOCYTE_MATURATION | KEGG_PROGESTERONE_MEDIATED_OOCYTE_MATURATION | KEGG_PROGESTERONE_MEDIATED_OOCYTE_MATURATION | 84 | 0.432077154240947 | 1.49651269218273 | 0.00658355808838098 | 0.0146741957391624 | 0.00526016689369692 | 16447 | tags=63%, list=39%, signal=38% | RPS6KA2/PIK3CB/CCNA1/GNAI1/ADCY6/PDE3A/ANAPC7/MAPK1/MAP2K1/PIK3CG/PRKACA/GNAI3/CDC23/MAPK8/CDK2/ARAF/PIK3R1/CDC16/PIK3R3/GNAI2/AKT2/ANAPC1/ANAPC11/ADCY3/ANAPC5/HSP90AB1/AKT1/ANAPC10/PIK3R2/KRAS/CDC25A/PRKACG/BUB1/PKMYT1/MAPK14/PLK1/PIK3CA/MAPK9/CCNA2/CDC25C/RPS6KA1/ADCY2/ADCY8/CCNB3/CDC27/CCNB2/BRAF/MAD2L2/SPDYC/RPS6KA3/ANAPC2/MAPK12/ANAPC13 |
| KEGG_HISTIDINE_METABOLISM | KEGG_HISTIDINE_METABOLISM | KEGG_HISTIDINE_METABOLISM | 29 | 0.532397369800008 | 1.60389463965142 | 0.00682330993120956 | 0.0150275278246877 | 0.00538682362990229 | 14533 | tags=69%, list=35%, signal=45% | ALDH3B2/UROC1/HDC/ACY3/FTCD/ALDH3B1/ALDH7A1/ALDH3A2/ALDH2/LCMT1/HNMT/ALDH9A1/MAOB/CNDP1/DDC/ALDH1A3/ASPA/TRMT11/METTL6/BUD23 |
| KEGG_GLYCEROLIPID_METABOLISM | KEGG_GLYCEROLIPID_METABOLISM | KEGG_GLYCEROLIPID_METABOLISM | 45 | 0.494448824746584 | 1.59829566289756 | 0.00711754868232947 | 0.0154619171530771 | 0.0055425364474757 | 10455 | tags=49%, list=25%, signal=37% | DGKG/MGLL/CEL/MBOAT1/TKFC/GLA/GK2/LIPF/ALDH7A1/LIPG/ALDH3A2/GLYCTK/GPAT3/ALDH2/AGPAT2/DGAT1/ALDH9A1/LPL/DGKD/PNPLA3/GPAT4/GPAM |
| KEGG_GNRH_SIGNALING_PATHWAY | KEGG_GNRH_SIGNALING_PATHWAY | KEGG_GNRH_SIGNALING_PATHWAY | 97 | 0.41369860846906 | 1.4538442520229 | 0.0071877020279169 | 0.0154619171530771 | 0.0055425364474757 | 10627 | tags=36%, list=25%, signal=27% | PLD1/PLA2G4A/ADCY6/PLCB4/GNAQ/PLCB1/CACNA1C/CDC42/CALM2/MAPK1/MAP2K1/PRKACA/EGFR/GNRH2/PLA2G4E/CALML5/MAPK8/PLA2G5/GNA11/CAMK2G/PLCB3/ELK1/NRAS/HBEGF/MMP14/PRKCD/CACNA1D/ATF4/MAP3K3/CALM1/ADCY3/MAP2K7/PLCB2/GRB2/KRAS |
| KEGG_PRIMARY_BILE_ACID_BIOSYNTHESIS | KEGG_PRIMARY_BILE_ACID_BIOSYNTHESIS | KEGG_PRIMARY_BILE_ACID_BIOSYNTHESIS | 15 | 0.62985959829798 | 1.66422116124816 | 0.00769871401920687 | 0.0161847965176508 | 0.00580166247858891 | 3376 | tags=33%, list=8%, signal=31% | AMACR/CYP39A1/HSD17B4/CYP7B1/ACOX2 |
| KEGG_BASE_EXCISION_REPAIR | KEGG_BASE_EXCISION_REPAIR | KEGG_BASE_EXCISION_REPAIR | 34 | 0.51088875931269 | 1.56353860642016 | 0.00762495171742335 | 0.0161847965176508 | 0.00580166247858891 | 7670 | tags=44%, list=18%, signal=36% | APEX1/TDG/LIG3/HMGB1P1/NEIL3/POLE/XRCC1/PARP2/OGG1/MPG/HMGB1/SMUG1/POLE3/POLD2/PARP4 |
| KEGG_HYPERTROPHIC_CARDIOMYOPATHY_HCM | KEGG_HYPERTROPHIC_CARDIOMYOPATHY_HCM | KEGG_HYPERTROPHIC_CARDIOMYOPATHY_HCM | 83 | 0.416451543532354 | 1.44157417046971 | 0.00788623680493085 | 0.0163927394259799 | 0.0058762024684878 | 9919 | tags=29%, list=24%, signal=22% | ITGA9/CACNA2D4/TPM4/CACNA1C/PRKAA1/TPM3/ITGA4/ITGA5/TNNI3/ACTG1/PRKAB1/TTN/ITGB4/TGFB2/ACTB/PRKAB2/CACNA2D3/ITGAV/ITGA7/CACNG5/CACNA1D/CACNG4/CACNB4/CACNG7 |
| KEGG_PORPHYRIN_AND_CHLOROPHYLL_METABOLISM | KEGG_PORPHYRIN_AND_CHLOROPHYLL_METABOLISM | KEGG_PORPHYRIN_AND_CHLOROPHYLL_METABOLISM | 34 | 0.509011873318853 | 1.55779453071343 | 0.00811953851001034 | 0.016690162492799 | 0.0059828178494813 | 6828 | tags=35%, list=16%, signal=30% | ALAD/UGT2B11/UGT2B28/UGT2A1/UROD/COX15/GUSB/FECH/MMAB/UROS/HCCS/ALAS1 |
| KEGG_LONG_TERM_DEPRESSION | KEGG_LONG_TERM_DEPRESSION | KEGG_LONG_TERM_DEPRESSION | 67 | 0.442606072577622 | 1.49782124602114 | 0.00892373386668462 | 0.0181416567619413 | 0.00650312589474993 | 10788 | tags=40%, list=26%, signal=30% | PLA2G4A/GNAI1/PLCB4/GUCY1A1/GNAQ/PLCB1/MAPK1/PPP2CB/MAP2K1/GNAI3/PLA2G4E/PLA2G5/GNA11/PLCB3/ARAF/NRAS/GNAI2/PPP2CA/GUCY1A2/GUCY1B1/PLCB2/GRIA3/CRH/GRID2/KRAS/PPP2R1A/GRM1 |
| KEGG_BLADDER_CANCER | KEGG_BLADDER_CANCER | KEGG_BLADDER_CANCER | 42 | 0.482366508173659 | 1.54614110320889 | 0.00957170829004097 | 0.0192474568875824 | 0.0068995151289769 | 8406 | tags=36%, list=20%, signal=29% | DAPK1/E2F3/VEGFA/CCND1/MAPK1/MAP2K1/CDK4/EGFR/E2F2/CDKN2A/ARAF/NRAS/RASSF1/MMP9/CDH1 |
| KEGG_GALACTOSE_METABOLISM | KEGG_GALACTOSE_METABOLISM | KEGG_GALACTOSE_METABOLISM | 26 | 0.545860530959027 | 1.62471306743719 | 0.00970934474885325 | 0.0193106001517719 | 0.00692214969878595 | 7108 | tags=42%, list=17%, signal=35% | HK2/PGM2/GALE/GLA/B4GALT2/HK3/GANC/G6PC2/PGM1/GLB1/PFKL |
| KEGG_STEROID_BIOSYNTHESIS | KEGG_STEROID_BIOSYNTHESIS | KEGG_STEROID_BIOSYNTHESIS | 17 | 0.587624020002008 | 1.59787721943759 | 0.00981187250954898 | 0.0193106001517719 | 0.00692214969878595 | 13022 | tags=65%, list=31%, signal=45% | DHCR7/CYP51A1/CEL/FDFT1/SQLE/EBP/NSDHL/CYP27B1/DHCR24/LIPA/MSMO1 |
| KEGG_GLYCOSAMINOGLYCAN_DEGRADATION | KEGG_GLYCOSAMINOGLYCAN_DEGRADATION | KEGG_GLYCOSAMINOGLYCAN_DEGRADATION | 21 | 0.561209630379835 | 1.59358654938015 | 0.010910738670119 | 0.0212472279365475 | 0.00761636051210523 | 8705 | tags=52%, list=21%, signal=41% | HGSNAT/HYAL3/HEXA/GUSB/GNS/HYAL2/GLB1/HEXB/ARSB/NAGLU/HPSE |
| KEGG_CALCIUM_SIGNALING_PATHWAY | KEGG_CALCIUM_SIGNALING_PATHWAY | KEGG_CALCIUM_SIGNALING_PATHWAY | 174 | 0.370408720374806 | 1.34383079492667 | 0.0111793023186029 | 0.0215434471764744 | 0.0077225443648244 | 10788 | tags=33%, list=26%, signal=25% | CHRNA7/P2RX1/CHP1/ITPKA/HTR2A/PLCB4/ERBB4/GNA15/GNAQ/AVPR1A/MYLK2/PLCB1/CACNA1C/CHRM3/PLCD3/PHKA2/AVPR1B/CALM2/BST1/ATP2A3/PHKG2/PPP3CB/PRKACA/VDAC2P5/EGFR/LTB4R2/ADRA1D/CCKAR/TRHR/PPP3R1/CALML5/VDAC3/DRD1/PHKB/GNA11/CAMK2G/PLCB3/HRH2/VDAC2/VDAC1/P2RX6/PPP3CA/SLC25A5/SLC8A3/ADRB1/ERBB3/CACNA1D/NTSR1/CALM1/ADCY3/CHRM2/PDGFRA/PLCB2/SLC25A6/EDNRA/P2RX4/PTGER1/GRM1 |
| KEGG_BASAL_TRANSCRIPTION_FACTORS | KEGG_BASAL_TRANSCRIPTION_FACTORS | KEGG_BASAL_TRANSCRIPTION_FACTORS | 33 | 0.495002702007654 | 1.50915915044712 | 0.0113489845210718 | 0.0216449704783328 | 0.0077589367859742 | 16245 | tags=64%, list=39%, signal=39% | TAF7L/GTF2F2/GTF2A2/GTF2I/TAF9/TAF6L/GTF2H4/TAF1L/GTF2IRD1/GTF2H1/GTF2B/TAF5L/TAF9B/TAF11/TBPL1/TBP/TAF12/TAF13/TAF4B/GTF2F1/GTF2H3 |
| KEGG_ETHER_LIPID_METABOLISM | KEGG_ETHER_LIPID_METABOLISM | KEGG_ETHER_LIPID_METABOLISM | 30 | 0.512408267903253 | 1.54578885498853 | 0.0119652668493966 | 0.0225874935422283 | 0.00809679711613303 | 6423 | tags=27%, list=15%, signal=23% | PLD1/PLA2G4A/AGPS/PAFAH1B2/PLA2G7/PLA2G4E/PLA2G5/PAFAH1B3 |
| KEGG_VASCULAR_SMOOTH_MUSCLE_CONTRACTION | KEGG_VASCULAR_SMOOTH_MUSCLE_CONTRACTION | KEGG_VASCULAR_SMOOTH_MUSCLE_CONTRACTION | 111 | 0.391846733466121 | 1.38719848679026 | 0.012189672968726 | 0.0227786818102455 | 0.00816533117522314 | 12655 | tags=39%, list=30%, signal=27% | PLA2G4A/ADCY6/PLCB4/RAMP1/GUCY1A1/GNAQ/AVPR1A/MYLK2/PLCB1/CACNA1C/AVPR1B/KCNMB4/CALM2/MAPK1/MAP2K1/MYL6B/CALCRL/PRKACA/ADRA1D/PLA2G4E/RHOA/CALML5/PLA2G5/GNA11/PLCB3/KCNMB3/KCNMB2/ARAF/PRKCD/GUCY1A2/CACNA1D/GUCY1B1/CALM1/ADCY3/PLCB2/EDNRA/MYL6/PRKACG/KCNMB1/ADORA2B/PPP1CB/PPP1CA/PPP1R14A |
| KEGG_P53_SIGNALING_PATHWAY | KEGG_P53_SIGNALING_PATHWAY | KEGG_P53_SIGNALING_PATHWAY | 68 | 0.429028609330842 | 1.45210024510676 | 0.0126352356434592 | 0.0233751859403995 | 0.0083791562688203 | 6643 | tags=29%, list=16%, signal=25% | STEAP3/GADD45A/CD82/CDK6/RCHY1/SESN1/SERPINE1/CASP3/CCND1/PPM1D/SIAH1/CHEK2/CDK4/EI24/TP53I3/CDKN2A/CASP9/ZMAT3/SERPINB5/CDK2 |
| KEGG_GLYCOSPHINGOLIPID_BIOSYNTHESIS_GLOBO_SERIES | KEGG_GLYCOSPHINGOLIPID_BIOSYNTHESIS_GLOBO_SERIES | KEGG_GLYCOSPHINGOLIPID_BIOSYNTHESIS_GLOBO_SERIES | 14 | 0.607172880999797 | 1.5781069352752 | 0.0135751612188307 | 0.0248653943117197 | 0.00891334191543863 | 8402 | tags=43%, list=20%, signal=34% | HEXA/B3GALNT1/GLA/NAGA/HEXB/GBGT1 |
| KEGG_STEROID_HORMONE_BIOSYNTHESIS | KEGG_STEROID_HORMONE_BIOSYNTHESIS | KEGG_STEROID_HORMONE_BIOSYNTHESIS | 46 | 0.466929007413384 | 1.51620572527073 | 0.0139114089999608 | 0.025231477107772 | 0.00904456931885994 | 1752 | tags=20%, list=4%, signal=19% | HSD17B12/CYP7B1/CYP19A1/AKR1C3/SRD5A3/UGT2B11/UGT2B28/COMT/UGT2A1 |
| KEGG_DRUG_METABOLISM_CYTOCHROME_P450 | KEGG_DRUG_METABOLISM_CYTOCHROME_P450 | KEGG_DRUG_METABOLISM_CYTOCHROME_P450 | 61 | 0.434634023719222 | 1.46097078874285 | 0.0141035967356581 | 0.0253317028747258 | 0.00908049662081209 | 9759 | tags=36%, list=23%, signal=28% | MGST1/ALDH3B2/CYP2E1/UGT2B11/FMO5/UGT2B28/UGT2A1/GSTA4/ADH6/GSTM2/ALDH3B1/MGST2/GSTT2/GSTZ1/ADH1A/CYP2C18/GSTP1/ADH5/CYP2C8/GSTM1/MAOB/GSTO1 |
| KEGG_RIBOFLAVIN_METABOLISM | KEGG_RIBOFLAVIN_METABOLISM | KEGG_RIBOFLAVIN_METABOLISM | 15 | 0.604238054727978 | 1.59652366944481 | 0.0165721577310059 | 0.0294748237127003 | 0.0105656551573264 | 13439 | tags=67%, list=32%, signal=45% | ACP6/RFK/MTMR7/ACP1/MTMR6/ACP3/ENPP1/FLAD1/MTMR2/MTMR1 |
| KEGG_GLYCOSAMINOGLYCAN_BIOSYNTHESIS_CHONDROITIN_SULFATE | KEGG_GLYCOSAMINOGLYCAN_BIOSYNTHESIS_CHONDROITIN_SULFATE | KEGG_GLYCOSAMINOGLYCAN_BIOSYNTHESIS_CHONDROITIN_SULFATE | 22 | 0.537502859332449 | 1.53810728845092 | 0.0167289539991002 | 0.0294748237127003 | 0.0105656551573264 | 12482 | tags=59%, list=30%, signal=41% | XYLT1/CHST3/CHPF2/CSGALNACT1/CHST13/CHST14/B3GALT6/CHSY1/B4GALT7/CSGALNACT2/B3GAT3/CHST12/B3GAT2 |
| KEGG_PPAR_SIGNALING_PATHWAY | KEGG_PPAR_SIGNALING_PATHWAY | KEGG_PPAR_SIGNALING_PATHWAY | 67 | 0.425311505199173 | 1.43929477730523 | 0.0188713485450584 | 0.0329358441588283 | 0.0118063054452699 | 9425 | tags=33%, list=23%, signal=25% | SORBS1/FADS2/SLC27A6/OLR1/SLC27A2/CD36/SCD/ACADM/ACOX2/ANGPTL4/SLC27A1/GK2/RXRG/CPT2/ME1/CPT1A/ACAA1/PPARG/RXRA/SCP2/LPL/ACOX3 |
| KEGG_SULFUR_METABOLISM | KEGG_SULFUR_METABOLISM | KEGG_SULFUR_METABOLISM | 13 | 0.615503465769181 | 1.57893402443156 | 0.0190767793766171 | 0.0329832166791978 | 0.0118232867754735 | 5132 | tags=46%, list=12%, signal=40% | SUOX/PAPSS1/SULT1A1/PAPSS2/SULT1A2/CHST13 |
| KEGG_TYROSINE_METABOLISM | KEGG_TYROSINE_METABOLISM | KEGG_TYROSINE_METABOLISM | 40 | 0.472562297981201 | 1.49929057094331 | 0.019404873119276 | 0.0332398289543153 | 0.0119152729679765 | 14533 | tags=52%, list=35%, signal=34% | ALDH3B2/NAA80/COMT/ADH6/PNMT/FAH/ALDH3B1/GSTZ1/ADH1A/HPD/LCMT1/ADH5/MAOB/DDC/MIF/ADH4/AOX1/ALDH1A3/TRMT11/METTL6/BUD23 |
| KEGG_TERPENOID_BACKBONE_BIOSYNTHESIS | KEGG_TERPENOID_BACKBONE_BIOSYNTHESIS | KEGG_TERPENOID_BACKBONE_BIOSYNTHESIS | 15 | 0.59526440782446 | 1.57281341225296 | 0.0211626920638026 | 0.0359183305670043 | 0.0128754186385279 | 6876 | tags=47%, list=16%, signal=39% | HMGCR/ACAT2/PDSS2/PDSS1/MVK/DHDDS/FDPS |
| KEGG_PRIMARY_IMMUNODEFICIENCY | KEGG_PRIMARY_IMMUNODEFICIENCY | KEGG_PRIMARY_IMMUNODEFICIENCY | 35 | -0.32396121 | -1.451739011 | 0.0227445822236157 | 0.0375691759943653 | 0.0134671868429304 | 7855 | tags=46%, list=19%, signal=37% | CD8B/CD19/TNFRSF13C/CD3E/IL2RG/BLNK/LCK/AICDA/ICOS/TNFRSF13B/JAK3/AIRE/ZAP70/CD40/TAP1/TAP2 |
| KEGG_MELANOGENESIS | KEGG_MELANOGENESIS | KEGG_MELANOGENESIS | 99 | 0.394625860318037 | 1.38639121732971 | 0.0227196831099396 | 0.0375691759943653 | 0.0134671868429304 | 11277 | tags=37%, list=27%, signal=27% | CREB3L4/GNAI1/ADCY6/KIT/PLCB4/FZD6/GNAQ/FZD9/PLCB1/FZD10/MITF/FZD1/CALM2/MAPK1/DVL3/MAP2K1/FZD8/PRKACA/WNT11/GNAI3/FZD7/CALML5/CAMK2G/PLCB3/CTNNB1/WNT7B/NRAS/KITLG/GNAI2/CALM1/ADCY3/PLCB2/KRAS/DVL2/WNT5B/PRKACG/TCF7L2 |
| KEGG_LEISHMANIA_INFECTION | KEGG_LEISHMANIA_INFECTION | KEGG_LEISHMANIA_INFECTION | 70 | -0.251062158 | -1.354732492 | 0.0226243368186027 | 0.0375691759943653 | 0.0134671868429304 | 4317 | tags=23%, list=10%, signal=21% | HLA-DMB/HLA-DMA/HLA-DQB1/IL10/NCF1/FCGR1A/IFNG/JAK2/FOS/MAPK13/NFKBIA/HLA-DOB/MAPK11/FCGR3B/FCGR3A/STAT1 |
| KEGG_TRYPTOPHAN_METABOLISM | KEGG_TRYPTOPHAN_METABOLISM | KEGG_TRYPTOPHAN_METABOLISM | 40 | 0.468888292420633 | 1.48763411439127 | 0.0233183765916636 | 0.0381301368521115 | 0.0136682709626346 | 10079 | tags=50%, list=24%, signal=38% | CAT/AADAT/ACMSD/HADHA/ACAT2/AFMID/GCDH/WARS2/HAAO/ALDH7A1/OGDH/ALDH3A2/HADH/CYP1B1/ALDH2/ECHS1/ALDH9A1/MAOB/TPH1/DDC |
| KEGG_DILATED_CARDIOMYOPATHY | KEGG_DILATED_CARDIOMYOPATHY | KEGG_DILATED_CARDIOMYOPATHY | 90 | 0.400218739301532 | 1.39316861087115 | 0.0234964086548147 | 0.0381301368521115 | 0.0136682709626346 | 9919 | tags=28%, list=24%, signal=21% | ITGA9/CACNA2D4/ADCY6/TPM4/CACNA1C/TPM3/ITGA4/ITGA5/PRKACA/TNNI3/ACTG1/TTN/ITGB4/TGFB2/ACTB/CACNA2D3/ITGAV/ITGA7/ADRB1/CACNG5/CACNA1D/CACNG4/ADCY3/CACNB4/CACNG7 |
| KEGG_LONG_TERM_POTENTIATION | KEGG_LONG_TERM_POTENTIATION | KEGG_LONG_TERM_POTENTIATION | 69 | 0.417101907822226 | 1.41720291849813 | 0.0257997936016512 | 0.0415040157939606 | 0.0148776841821879 | 16106 | tags=55%, list=39%, signal=34% | RPS6KA2/CHP1/PLCB4/GNAQ/PLCB1/CACNA1C/RAPGEF3/CALM2/MAPK1/MAP2K1/PPP3CB/PRKACA/PPP3R1/CALML5/CAMK2G/PLCB3/PPP3CA/ARAF/NRAS/ATF4/CALM1/RAP1B/PLCB2/KRAS/GRM1/PRKACG/PPP1CB/PPP1CA/PPP1CC/RPS6KA1/ADCY8/GRIN2B/RAP1A/BRAF/GRIN2A/GRIA2/RPS6KA3/MAP2K2 |
| KEGG_ENDOMETRIAL_CANCER | KEGG_ENDOMETRIAL_CANCER | KEGG_ENDOMETRIAL_CANCER | 52 | 0.444890582228077 | 1.47004284726199 | 0.0270622957769346 | 0.0431596958511457 | 0.0154711854260152 | 13965 | tags=56%, list=33%, signal=37% | PIK3CB/CTNNA1/CCND1/MAPK1/MAP2K1/PIK3CG/EGFR/CASP9/ELK1/CTNNA2/CTNNB1/ARAF/PIK3R1/NRAS/PIK3R3/AKT2/MLH1/CDH1/AKT1/PIK3R2/APC2/GRB2/KRAS/TCF7L2/ILK/PIK3CA/BAD/PDPK1/TP53 |
| KEGG_DRUG_METABOLISM_OTHER_ENZYMES | KEGG_DRUG_METABOLISM_OTHER_ENZYMES | KEGG_DRUG_METABOLISM_OTHER_ENZYMES | 44 | 0.459739646370737 | 1.48396960648446 | 0.0276890308839191 | 0.0437818009702994 | 0.0156941875455412 | 10279 | tags=41%, list=25%, signal=31% | UPP2/UGT2B11/UGT2B28/UGT2A1/TK2/UCK2/GUSB/CES2/IMPDH2/TPMT/TK1/HPRT1/DPYD/GMPS/IMPDH1/ITPA/CDA/NAT1 |
| KEGG_RNA_DEGRADATION | KEGG_RNA_DEGRADATION | KEGG_RNA_DEGRADATION | 55 | 0.434619326074287 | 1.44667027655976 | 0.0292580982236155 | 0.0458707472149903 | 0.0164429990016751 | 13064 | tags=53%, list=31%, signal=36% | XRN2/EXOSC3/ENO1/CNOT8/CNOT7/C1DP2/LSM2/EDC3/HSPA9/EXOSC5/CNOT6/MTREX/LSM5/CNOT10/EXOSC1/LSM6/LSM4/LSM3/EXOSC6/CNOT2/CNOT9/EXOSC10/DCP1A/C1D/EXOSC7/LSM7/DIS3/LSM1/EXOSC8 |
| KEGG_GLYCEROPHOSPHOLIPID_METABOLISM | KEGG_GLYCEROPHOSPHOLIPID_METABOLISM | KEGG_GLYCEROPHOSPHOLIPID_METABOLISM | 74 | 0.408931268103031 | 1.402938588 | 0.0318275154004107 | 0.0494797508325712 | 0.0177366958887738 | 10665 | tags=34%, list=26%, signal=25% | PLD1/PLA2G4A/DGKG/CRLS1/PTDSS1/CHKA/LPCAT3/PCYT1B/GNPAT/MBOAT1/PLA2G4E/PEMT/PLA2G5/LYPLA1/TAFAZZIN/GPAT3/PISD/AGPAT2/DGKD/GPAT4/PTDSS2/GPD2/PCYT1A/GPAM/LYPLA2 |

**Table S10**

|  | ID | Description | setSize | enrichmentScore | NES | pvalue | p.adjust | qvalue | rank | leading_edge | core_enrichment |
| --- | --- | --- | --- | --- | --- | --- | --- | --- | --- | --- | --- |
| KEGG_RIBOSOME | KEGG_RIBOSOME | KEGG_RIBOSOME | 88 | 0.72434927472876 | 2.32101160556175 | 1e-10 | 3.72e-09 | 2.61052631578947e-09 | 9886 | tags=86%, list=24%, signal=66% | RPL36AL/RPL28/RPS9/FAU/RPL26L1/RPL38/RPS11/RPS15/RPL24/RPL8/RPL23/RPL39/RPS13/RPS16/RPLP2/RPL11/RPL41/RPS19/RPL18/RPS26/RPL19/RPL13/RPS20/RPL27A/RPS24/RPL18A/RPL37A/RPL32/RPS4X/RPS7/RSL24D1P11/RPL37/RPL35A/RPL36/RPL13A/RPL35/RPL29/RPS15A/RPS3/RPL34/RPLP1/RPS2/RPL9/RPL30/RPS21/RPS5/RPL23A/RPL12/RPL27/RPS8/RPS3A/RPL10/RPS29/RPS18/RPS27/RPL36A/RPL6/RPL26/RPL10A/RPS6/RPS17/RPL3/RPL31/RPS25/RPS27A/RPLP0/RPL14/RPL17/RPL22L1/RPSA/RPL22/RPL7A/RPL15/RPS23/RPS10/RPL5 |
| KEGG_SYSTEMIC_LUPUS_ERYTHEMATOSUS | KEGG_SYSTEMIC_LUPUS_ERYTHEMATOSUS | KEGG_SYSTEMIC_LUPUS_ERYTHEMATOSUS | 127 | 0.688353805723614 | 2.24780636539404 | 1e-10 | 3.72e-09 | 2.61052631578947e-09 | 8445 | tags=62%, list=20%, signal=50% | H4C1/H2AC20/C1QB/H2AC18/H2AC19/H4C5/C1QA/H2BC5/H4C2/C1R/C1QC/H2AB2/H2AB3/H2AC16/C2/H4C12/H2AJ/ACTN2/H3C1/C7/H4C11/H2BC12/H3-3B/C1S/H3C13/H4C6/H2AC21/H3-5/H3-3A/H2AC17/C3/H2BC21/CD86/H2BC17/H2BW1/H4C8/HLA-DMA/TNF/H4C3/H2AB1/H2AC8/H2BC10/H3C8/FCGR1A/H3C11/HLA-DRA/H4C4/H4C14/H2BC11/TRIM21/H4C9/H2BC15/IL10/H2BC8/H2AC13/H4C15/H3C2/FCGR2A/H2AC6/CD40/HLA-DQA2/H2AC14/H2AC4/HLA-DPB1/H3C3/HLA-DMB/C4B/C4A/IFNG/FCGR3A/H2BC9/H2AC11/H3C15/H2BC6/H3C7/H2BC4/HLA-DRB1/H3C10/SNRPB |
| KEGG_OXIDATIVE_PHOSPHORYLATION | KEGG_OXIDATIVE_PHOSPHORYLATION | KEGG_OXIDATIVE_PHOSPHORYLATION | 126 | 0.645842191017403 | 2.10657540102455 | 1e-10 | 3.72e-09 | 2.61052631578947e-09 | 10063 | tags=69%, list=24%, signal=53% | COX4I2/TCIRG1/UQCRHL/COX7B/ATP6V1D/NDUFC2/NDUFB6/SDHB/ATP5F1E/NDUFA1/ATP5F1D/UQCR11/NDUFS7/ATP6V0E1/COX5B/COX6B1/ATP6V1G1/UQCR10/ATP6V0B/ATP5ME/ATP5PO/ATP5PF/NDUFA7/NDUFB3/COX6A2/COX5A/NDUFS6/UQCRH/COX4I1/ATP6V1F/NDUFB5/PPA1/NDUFA6/NDUFA2/ATP5MC2/ATP6V0D1/ATP5MF/ATP5PD/COX7A2/NDUFS5/COX7C/ATP6V1H/NDUFS4/NDUFA3/COX6A1/ATP5MG/COX8A/NDUFB10/NDUFA8/CYC1/ATP6V1E1/ATP6V1B2/NDUFB8/ATP6AP1/NDUFV2/NDUFA11/ATP6V0C/UQCRQ/NDUFB7/ATP5F1C/COX6C/NDUFB1/NDUFS8/COX17/NDUFAB1/NDUFA4/NDUFB9/NDUFB2/ATP5PB/NDUFA9/UQCRFS1/UQCRC1/ATP5MC1/UQCRB/NDUFA4L2/NDUFB4/NDUFS3/COX7A2L/MT-CO3/ATP5F1B/NDUFV1/COX7A1/SDHA/SDHD/UQCRC2/NDUFC1/ATP5MC3 |
| KEGG_PARKINSONS_DISEASE | KEGG_PARKINSONS_DISEASE | KEGG_PARKINSONS_DISEASE | 125 | 0.63152712663992 | 2.06506080211374 | 1e-10 | 3.72e-09 | 2.61052631578947e-09 | 10063 | tags=69%, list=24%, signal=52% | COX4I2/SLC18A1/UBE2L6/UQCRHL/COX7B/UBE2L3/NDUFC2/NDUFB6/SDHB/ATP5F1E/NDUFA1/ATP5F1D/VDAC2/UQCR11/NDUFS7/SLC25A6/COX5B/COX6B1/UQCR10/ATP5PO/ATP5PF/UBE2J2/NDUFA7/NDUFB3/COX6A2/COX5A/NDUFS6/UQCRH/COX4I1/NDUFB5/NDUFA6/NDUFA2/CYCS/ATP5MC2/PARK7/ATP5PD/VDAC2P5/COX7A2/NDUFS5/HTRA2/COX7C/NDUFS4/NDUFA3/COX6A1/COX8A/UBE2J1/NDUFB10/NDUFA8/CYC1/NDUFB8/NDUFV2/UQCRQ/PPID/NDUFB7/ATP5F1C/COX6C/NDUFB1/NDUFS8/NDUFAB1/NDUFA4/NDUFB9/NDUFB2/UBA7/ATP5PB/SLC25A5/NDUFA9/UQCRFS1/UQCRC1/ATP5MC1/UQCRB/CASP9/NDUFA4L2/NDUFB4/NDUFS3/COX7A2L/MT-CO3/ATP5F1B/GPR37/NDUFV1/COX7A1/SDHA/SDHD/UQCRC2/NDUFC1/UCHL1/ATP5MC3 |
| KEGG_ALZHEIMERS_DISEASE | KEGG_ALZHEIMERS_DISEASE | KEGG_ALZHEIMERS_DISEASE | 161 | 0.586494695407235 | 1.93729407814885 | 1e-10 | 3.72e-09 | 2.61052631578947e-09 | 10295 | tags=61%, list=25%, signal=46% | IL1B/NOS1/COX4I2/BID/UQCRHL/COX7B/GRIN2D/PSENEN/NDUFC2/NDUFB6/SDHB/ATP5F1E/NDUFA1/ATP5F1D/CASP7/UQCR11/NDUFS7/COX5B/COX6B1/UQCR10/PLCB3/CALML3/ATP5PO/ATP5PF/NDUFA7/TNF/NDUFB3/COX6A2/COX5A/NDUFS6/ATP2A1/CACNA1S/UQCRH/COX4I1/NDUFB5/MAPK3/NDUFA6/NDUFA2/BAD/CYCS/ATP5MC2/ATP5PD/COX7A2/NDUFS5/COX7C/NDUFS4/NDUFA3/COX6A1/COX8A/NDUFB10/NDUFA8/APOE/CYC1/CALM3/NDUFB8/NDUFV2/LRP1/UQCRQ/HSD17B10/NDUFB7/ATP5F1C/COX6C/NDUFB1/NDUFS8/CDK5/NDUFAB1/NDUFA4/NDUFB9/NDUFB2/ATP5PB/NDUFA9/UQCRFS1/UQCRC1/ATP5MC1/UQCRB/PSEN2/CASP9/GAPDH/NDUFA4L2/NDUFB4/NDUFS3/COX7A2L/MT-CO3/ATP5F1B/CHP1/NDUFV1/COX7A1/MAPT/SDHA/GRIN2A/SDHD/UQCRC2/NDUFC1/ATP5MC3/TNFRSF1A/FAS/PPP3R1/NDUFV3 |
| KEGG_HUNTINGTONS_DISEASE | KEGG_HUNTINGTONS_DISEASE | KEGG_HUNTINGTONS_DISEASE | 176 | 0.562377630485306 | 1.86760365306033 | 7.41540214288558e-10 | 2.29877466429453e-08 | 1.61317520301371e-08 | 10063 | tags=57%, list=24%, signal=44% | UCP1/COX4I2/UQCRHL/COX7B/SOD2/NDUFC2/NDUFB6/SDHB/ATP5F1E/NDUFA1/ATP5F1D/VDAC2/UQCR11/NDUFS7/BBC3/POLR2E/SLC25A6/COX5B/COX6B1/POLR2L/UQCR10/PLCB3/CLTB/ATP5PO/ATP5PF/NDUFA7/POLR2J/NDUFB3/COX6A2/COX5A/NDUFS6/PPARG/UQCRH/COX4I1/CLTA/NDUFB5/NDUFA6/NDUFA2/AP2S1/CYCS/ATP5MC2/ATP5PD/VDAC2P5/COX7A2/NDUFS5/COX7C/NDUFS4/NDUFA3/COX6A1/CREB3/COX8A/NDUFB10/POLR2I/NDUFA8/CYC1/POLR2G/NDUFB8/NDUFV2/UQCRQ/PPID/NDUFB7/ATP5F1C/COX6C/NDUFB1/NDUFS8/GPX1/NDUFAB1/NDUFA4/NDUFB9/NDUFB2/ATP5PB/SOD1/SLC25A5/NDUFA9/UQCRFS1/POLR2H/NRF1/UQCRC1/POLR2F/ATP5MC1/DLG4/AP2M1/UQCRB/CASP9/NDUFA4L2/NDUFB4/NDUFS3/COX7A2L/MT-CO3/ATP5F1B/NDUFV1/COX7A1/BAX/DCTN2/SDHA/SDHD/UQCRC2/DNAL4/NDUFC1/AP2A2/ATP5MC3 |
| KEGG_CYTOKINE_CYTOKINE_RECEPTOR_INTERACTION | KEGG_CYTOKINE_CYTOKINE_RECEPTOR_INTERACTION | KEGG_CYTOKINE_CYTOKINE_RECEPTOR_INTERACTION | 250 | 0.524742503768649 | 1.76464185895603 | 1.18754448137749e-09 | 3.15547533623162e-08 | 2.21436865700464e-08 | 7462 | tags=32%, list=18%, signal=26% | CCL20/IFNA1/OSM/CXCL2/IL1B/CCL3L1/IL12B/TSLP/CCL19/TNFSF14/AMH/CXCL8/IL15RA/CXCL12/CCL3/TNFRSF14/IL6/CXCL10/CXCL1/CCL17/ACVRL1/IL1A/TNFRSF6B/IL15/IFNGR2/RELT/GH1/CCL4L2/CXCL11/TGFB1/CSF3/CX3CL1/CCL2/KDR/CXCL16/IL2RG/GHR/LTBR/CXCL14/CCL21/CCL11/CCL13/TNF/CCL14/TNFRSF10D/TNFRSF18/PDGFA/PDGFRA/CCL8/EPOR/XCR1/CXCL9/IL10/TNFRSF1B/IL10RB/FLT1/LIFR/TNFSF13B/IL25/LTB/CD40/TNFSF18/VEGFA/CCL4/VEGFB/TNFSF12/IL18/INHBB/CLCF1/IL12RB1/TNFSF10/IL5/VEGFD/TNFSF13/INHBC/IL23A/TNFRSF11B/KITLG/IFNG/TNFRSF10B |
| KEGG_LYSOSOME | KEGG_LYSOSOME | KEGG_LYSOSOME | 120 | 0.574599917656445 | 1.86850623679094 | 9.03274082147525e-08 | 2.100112240993e-06 | 1.47376297613544e-06 | 10298 | tags=59%, list=25%, signal=45% | FUCA1/NPC2/TCIRG1/CTSL/ACP2/CTSZ/LAPTM4A/CTSS/CTSH/NEU1/GLA/LAPTM5/DNASE2/PLA2G15/CTSB/HEXB/SMPD1/GNPTG/LGMN/ARSA/CLN3/CTSA/GAA/ATP6V0B/PPT1/PSAPL1/CLTB/ARSB/MCOLN1/CLTA/MAN2B1/ATP6V0D1/AP3S1/SCARB2/ACP5/ATP6V1H/ATP6AP1/CTNS/GM2A/ATP6V0C/AP1B1/AP3S2/MANBA/LAMP1/CD63/PSAP/NAGPA/AP1S1/IDUA/LAMP3/HYAL1/CTSD/CTSC/ASAH1/SGSH/CD68/GGA1/SLC17A5/CTSK/M6PR/NAGA/GLB1/NAGLU/GNS/AP3D1/TPP1/AP1S2/GALNS/SUMF1/GUSB/AP4M1 |
| KEGG_COMPLEMENT_AND_COAGULATION_CASCADES | KEGG_COMPLEMENT_AND_COAGULATION_CASCADES | KEGG_COMPLEMENT_AND_COAGULATION_CASCADES | 65 | 0.651590494007263 | 2.029615831 | 2.74804684705645e-07 | 5.67929681725e-06 | 3.98547145070175e-06 | 7673 | tags=46%, list=18%, signal=38% | PLAUR/F3/C1QB/C1QA/C1R/C1QC/CFB/C2/F12/BDKRB2/C7/CD55/C1S/THBD/C3/C4BPA/A2M/PLAU/SERPING1/CFD/CFI/SERPIND1/PLAT/C5AR1/C4B/SERPINE1/SERPINA5/C4A/SERPINC1/BDKRB1 |
| KEGG_PROTEASOME | KEGG_PROTEASOME | KEGG_PROTEASOME | 45 | 0.684278139079977 | 2.02932698116685 | 5.62205783993346e-07 | 1.04570275822762e-05 | 7.33826497001841e-06 | 8234 | tags=64%, list=20%, signal=52% | PSMB10/POMP/PSME1/PSME2/PSMB9/PSMB3/PSMB8/PSMA6/PSMD4/PSMC4/PSMB1/PSMB4/PSMA7/PSMA4/PSMB11/PSMC1/PSMB6/PSMA5/PSMB2/PSMB7/PSMA2/PSMD8/PSMD13/PSMC5/PSMB5/IFNG/PSMA1/PSMD12/PSMC3 |
| KEGG_EPITHELIAL_CELL_SIGNALING_IN_HELICOBACTER_PYLORI_INFECTION | KEGG_EPITHELIAL_CELL_SIGNALING_IN_HELICOBACTER_PYLORI_INFECTION | KEGG_EPITHELIAL_CELL_SIGNALING_IN_HELICOBACTER_PYLORI_INFECTION | 68 | 0.631168398556468 | 1.98302357176624 | 6.72384910104337e-07 | 1.13694175708551e-05 | 7.97853864621414e-06 | 5521 | tags=41%, list=13%, signal=36% | IGSF5/CXCL8/NFKBIA/RAC1/TCIRG1/CXCL1/JUN/HBEGF/ATP6V1D/RELA/CSK/JAM2/ATP6V0E1/ATP6V1G1/SRC/ATP6V0B/ATP6V1F/NFKB1/IKBKG/PTPRZ1/PAK1/ATP6V0D1/ATP6V1H/TJP1/ATP6V1E1/ATP6V1B2/ATP6AP1/ATP6V0C |
| KEGG_TOLL_LIKE_RECEPTOR_SIGNALING_PATHWAY | KEGG_TOLL_LIKE_RECEPTOR_SIGNALING_PATHWAY | KEGG_TOLL_LIKE_RECEPTOR_SIGNALING_PATHWAY | 95 | 0.572028728616759 | 1.83713125608823 | 3.14766601184821e-06 | 4.87888231836472e-05 | 3.4237770655191e-05 | 6746 | tags=38%, list=16%, signal=32% | IFNA1/IL1B/IL12B/MAP3K8/CXCL8/TICAM1/NFKBIA/RAC1/CCL3/IL6/CXCL10/IRF5/IRF7/JUN/CXCL11/MAP2K3/RELA/MAP2K2/IKBKE/CD86/SPP1/STAT1/TNF/NFKB1/MAPK3/IKBKG/LY96/MYD88/CXCL9/CD40/TRAF3/TOLLIP/CCL4/CD14/IRAK1/TLR2 |
| KEGG_CHEMOKINE_SIGNALING_PATHWAY | KEGG_CHEMOKINE_SIGNALING_PATHWAY | KEGG_CHEMOKINE_SIGNALING_PATHWAY | 185 | 0.497964356054292 | 1.6536673757604 | 4.27275811621594e-06 | 6.11333084320127e-05 | 4.29005673207107e-05 | 10118 | tags=41%, list=24%, signal=31% | CCL20/CXCL2/CCL3L1/CCL19/CXCL8/NCF1/CXCL12/NFKBIA/RAC1/CCL3/NFKBIB/CXCL10/CXCL1/CCL17/GNG5/CCL4L2/CXCL11/GRB2/RELA/CSK/CX3CL1/CCL2/CXCL16/GNB2/WAS/PLCB3/CXCL14/CCL21/CCL11/CCL13/STAT1/CCL14/NFKB1/MAPK3/IKBKG/GNAI2/GNG8/PAK1/ARRB2/CCL8/XCR1/CXCL9/VAV1/STAT2/GRK3/HCK/PRKCD/CCL4/GRK2/GNGT2/GNG13/HRAS/GNG10/GNG12/GNG3/SHC3/CRK/FGR/RHOA/RAC2/GNB1/LYN/PRKACA/GSK3A/CCL18/PARD3/AKT1/CCL22/CXCL3/GRK5/RAP1A/CXCR4/ADCY1/JAK2/GNG11/GRK6 |
| KEGG_NOD_LIKE_RECEPTOR_SIGNALING_PATHWAY | KEGG_NOD_LIKE_RECEPTOR_SIGNALING_PATHWAY | KEGG_NOD_LIKE_RECEPTOR_SIGNALING_PATHWAY | 61 | 0.621928679470903 | 1.92769669292039 | 6.33394756368003e-06 | 7.85409497896323e-05 | 5.51164559927244e-05 | 4348 | tags=38%, list=10%, signal=34% | CXCL2/IL1B/CXCL8/NFKBIA/NFKBIB/IL6/CXCL1/CASP1/RIPK2/TNFAIP3/NLRP3/RELA/PYCARD/CCL2/TRIP6/CCL11/CCL13/CARD9/TNF/NFKB1/MAPK3/IKBKG/CCL8 |
| KEGG_CARDIAC_MUSCLE_CONTRACTION | KEGG_CARDIAC_MUSCLE_CONTRACTION | KEGG_CARDIAC_MUSCLE_CONTRACTION | 76 | 0.587179063549108 | 1.85614345881684 | 6.03760753989888e-06 | 7.85409497896323e-05 | 5.51164559927244e-05 | 8785 | tags=58%, list=21%, signal=46% | COX4I2/SLC9A1/UQCRHL/COX7B/CACNA2D1/MYL2/UQCR11/COX5B/MYH7/COX6B1/UQCR10/ATP1B4/CACNG1/MYH6/COX6A2/COX5A/CACNA1S/UQCRH/COX4I1/TNNC1/COX7A2/COX7C/ATP1B3/COX6A1/COX8A/ATP1B2/CYC1/ACTC1/UQCRQ/MYL3/COX6C/ATP1A1/ATP1A4/UQCRFS1/UQCRC1/CACNG3/UQCRB/TPM1/CACNG7/SLC8A1/ATP1A2/TPM3/COX7A2L/MT-CO3 |
| KEGG_LEISHMANIA_INFECTION | KEGG_LEISHMANIA_INFECTION | KEGG_LEISHMANIA_INFECTION | 70 | 0.590728326645222 | 1.8577080313501 | 8.52844495032384e-06 | 9.91431725475147e-05 | 6.95741561736945e-05 | 7690 | tags=49%, list=18%, signal=40% | IL1B/IL12B/NCF1/NFKBIA/NFKBIB/IL1A/JUN/IFNGR2/TGFB1/RELA/NOS2/C3/CYBA/STAT1/HLA-DMA/TNF/NFKB1/MAPK3/FCGR1A/HLA-DRA/MYD88/ITGB2/IL10/PTPN6/FCGR2A/HLA-DQA2/HLA-DPB1/IRAK1/NCF2/HLA-DMB/TLR2/IFNG/FCGR3A/NCF4 |
| KEGG_ECM_RECEPTOR_INTERACTION | KEGG_ECM_RECEPTOR_INTERACTION | KEGG_ECM_RECEPTOR_INTERACTION | 84 | 0.550167456216822 | 1.75355869645339 | 6.88822927751512e-05 | 0.000753653320951654 | 0.000528879523474845 | 5692 | tags=30%, list=14%, signal=26% | THBS1/LAMB3/ITGA11/THBS2/COL11A1/COL1A2/FN1/COL3A1/COL1A1/COL5A2/AGRN/TNC/COL5A1/SDC4/COL6A3/SPP1/COL6A1/ITGB6/IBSP/CD36/GP9/COL4A1/LAMA4/GP1BB/SDC3 |
| KEGG_GLUTATHIONE_METABOLISM | KEGG_GLUTATHIONE_METABOLISM | KEGG_GLUTATHIONE_METABOLISM | 45 | 0.615811753626109 | 1.82627989348506 | 8.95092857986258e-05 | 0.0009249292865858 | 0.000649073183568982 | 12672 | tags=73%, list=30%, signal=51% | LAP3/GSTT2/GSTM1/GPX4/MGST2/GSTK1/MGST3/GPX2/GSTO1/GSTO2/GPX3/GSTM5/GSTA1/GPX1/TXNDC12/SMS/GSTP1/GSTM3/GSR/GSTM4/ANPEP/G6PD/SRM/MGST1/GGT1/IDH1/GGT5/IDH2/GSTZ1/GSS/GGCT/PGD/GSTM2 |
| KEGG_CYTOSOLIC_DNA_SENSING_PATHWAY | KEGG_CYTOSOLIC_DNA_SENSING_PATHWAY | KEGG_CYTOSOLIC_DNA_SENSING_PATHWAY | 47 | 0.618727182799619 | 1.85241757611712 | 9.7088292623438e-05 | 0.000950443285682078 | 0.0006669777443383 | 8540 | tags=51%, list=20%, signal=41% | IFNA1/IL1B/NFKBIA/NFKBIB/IL6/CXCL10/IRF7/CASP1/CCL4L2/RELA/PYCARD/TREX1/IKBKE/NFKB1/IKBKG/AIM2/CCL4/IL18/POLR3K/ZBP1/IRF3/POLR3GL/POLR3H/TBK1 |
| KEGG_VIRAL_MYOCARDITIS | KEGG_VIRAL_MYOCARDITIS | KEGG_VIRAL_MYOCARDITIS | 67 | 0.573724436234254 | 1.7931938199433 | 0.000118392378103374 | 0.00110104911636138 | 0.000772666046569389 | 8372 | tags=46%, list=20%, signal=37% | ICAM1/RAC1/BID/HLA-B/CD55/HLA-F/HLA-A/CD86/MYH7/MYH1/MYH2/MYH6/HLA-E/HLA-DMA/HLA-DRA/CYCS/ITGB2/SGCG/CCND1/SGCA/HLA-C/CD40/HLA-DQA2/HLA-DPB1/ACTB/HLA-DMB/CAV1/CXADR/RAC2/CASP9/HLA-DRB1 |
| KEGG_ANTIGEN_PROCESSING_AND_PRESENTATION | KEGG_ANTIGEN_PROCESSING_AND_PRESENTATION | KEGG_ANTIGEN_PROCESSING_AND_PRESENTATION | 74 | 0.543610547079583 | 1.71547990680443 | 0.000379328783604588 | 0.00335976922621206 | 0.00235773279032425 | 8487 | tags=46%, list=20%, signal=37% | IFNA1/HSPA2/HLA-B/CTSL/PSME1/PSME2/IFI30/TAPBP/CTSS/TAP1/CD74/PDIA3/CTSB/HLA-F/HLA-A/LGMN/HLA-E/HLA-DMA/B2M/RFXANK/NFYC/HLA-DRA/HSPA5/HSPA6/HLA-C/HLA-DQA2/HLA-DPB1/HLA-DMB/CALR/KIR2DL4/TAP2/KIR3DL3/HLA-DRB1/LTA |
| KEGG_TYPE_I_DIABETES_MELLITUS | KEGG_TYPE_I_DIABETES_MELLITUS | KEGG_TYPE_I_DIABETES_MELLITUS | 40 | 0.61706452252614 | 1.79885141960932 | 0.000494032194503572 | 0.00387625047869802 | 0.00272017577452493 | 8487 | tags=50%, list=20%, signal=40% | IL1B/IL12B/HLA-B/IL1A/HLA-F/HLA-A/CD86/HLA-E/HLA-DMA/TNF/HLA-DRA/HLA-C/HLA-DQA2/PTPRN/HLA-DPB1/HLA-DMB/CPE/IFNG/HLA-DRB1/LTA |
| KEGG_CELL_ADHESION_MOLECULES_CAMS | KEGG_CELL_ADHESION_MOLECULES_CAMS | KEGG_CELL_ADHESION_MOLECULES_CAMS | 130 | 0.485730804395194 | 1.59113089303989 | 0.00046908802356564 | 0.00387625047869802 | 0.00272017577452493 | 8372 | tags=33%, list=20%, signal=27% | ICAM1/CLDN16/CLDN1/HLA-B/NECTIN2/CLDN7/CNTN2/MPZ/CDH5/JAM2/CLDN11/SDC4/HLA-F/HLA-A/CD86/HLA-E/CLDN18/HLA-DMA/SIGLEC1/ICAM2/CDH15/HLA-DRA/CDH2/ITGB2/CD276/SELE/NLGN4X/MAG/HLA-C/CD40/SDC3/HLA-DQA2/ICAM3/CLDN2/HLA-DPB1/HLA-DMB/ESAM/CDH4/SELL/PVR/CADM3/CD274/HLA-DRB1 |
| KEGG_NEUROACTIVE_LIGAND_RECEPTOR_INTERACTION | KEGG_NEUROACTIVE_LIGAND_RECEPTOR_INTERACTION | KEGG_NEUROACTIVE_LIGAND_RECEPTOR_INTERACTION | 250 | 0.431361783096344 | 1.45061445059027 | 0.000500161352090068 | 0.00387625047869802 | 0.00272017577452493 | 9971 | tags=31%, list=24%, signal=24% | GABRA6/TACR3/GPR35/ADRA2B/CHRNB4/HTR5A/GABRG3/GALR2/S1PR2/ADORA2B/GLRA2/LPAR1/ADRA1A/CHRNA1/CHRNA2/GH1/BDKRB2/CHRNB2/GRIN2D/HRH3/P2RX4/ADORA2A/CHRNA6/PTH1R/LPAR2/CHRNE/GHR/GRM3/HTR7/PTGIR/NTSR1/ADORA1/NPFFR1/NMUR2/HTR2B/P2RY6/SSTR3/AGTR1/PTGER2/CHRNA4/PTGFR/S1PR3/C5AR1/GRM8/CHRM1/GRIK3/CHRNA10/GRIK1/DRD1/FPR1/CRHR2/GRPR/LHB/GABRA4/GRID2/CHRNA9/EDNRA/TBXA2R/BDKRB1/GABRA1/P2RY13/GALR1/LPAR6/GABBR2/GABRA5/CHRNB1/GRM4/PARD3/UTS2R/PTAFR/CHRM4/GRIN2A/P2RY14/SSTR2/P2RX6/GRIN3B/S1PR4 |
| KEGG_TIGHT_JUNCTION | KEGG_TIGHT_JUNCTION | KEGG_TIGHT_JUNCTION | 128 | 0.483910143160663 | 1.58065405457665 | 0.000544500079304477 | 0.00405108059002531 | 0.00284286357194758 | 10389 | tags=45%, list=25%, signal=34% | IGSF5/MYL7/CLDN16/CLDN1/CLDN7/EPB41L3/ACTN2/MAGI2/MYL12A/MYL2/PPP2CA/MYL12B/PRKCG/JAM2/CLDN11/MYH7/SRC/MYH1/MYH2/MYH6/CLDN18/MPDZ/MRAS/MYL9/RRAS/GNAI2/VAPA/RAB13/TJP1/RAB3B/EXOC3/PRKCD/PPP2R1A/PPP2R2C/CLDN2/ACTB/HRAS/EPB41L1/CTTN/TJP2/MYL5/RHOA/HCLS1/CSNK2B/PPP2R2A/CTNNA3/PARD6A/PPP2CB/PARD3/CTNNA2/ACTG1/AKT1/CLDN5/MYH14/PRKCE/SYMPK/CDC42 |
| KEGG_PRION_DISEASES | KEGG_PRION_DISEASES | KEGG_PRION_DISEASES | 34 | 0.626865956836479 | 1.78945165491118 | 0.000686294453529053 | 0.00490964493678476 | 0.00344536486791913 | 4245 | tags=29%, list=10%, signal=26% | IL1B/C1QB/C1QA/IL6/C1QC/IL1A/C7/MAP2K2/MAPK3/HSPA5 |
| KEGG_AMYOTROPHIC_LATERAL_SCLEROSIS_ALS | KEGG_AMYOTROPHIC_LATERAL_SCLEROSIS_ALS | KEGG_AMYOTROPHIC_LATERAL_SCLEROSIS_ALS | 52 | 0.559993811541629 | 1.71421985379763 | 0.00108217368187341 | 0.0071887251724448 | 0.00504471941925951 | 11864 | tags=63%, list=28%, signal=45% | NOS1/RAC1/BID/PRPH/CASP1/MAP2K3/GRIN2D/TOMM40L/TNF/DERL1/SLC1A2/BAD/CYCS/TNFRSF1B/CCS/GPX1/SOD1/CASP9/NEFM/CHP1/BAX/GRIN2A/MAPK11/PRPH2/TNFRSF1A/PPP3R1/TOMM40/DAXX/GRIA1/GRIA2/GRIN2B/RAB5A/MAP2K6 |
| KEGG_MAPK_SIGNALING_PATHWAY | KEGG_MAPK_SIGNALING_PATHWAY | KEGG_MAPK_SIGNALING_PATHWAY | 257 | 0.422836211222997 | 1.42218316090007 | 0.00105953587803204 | 0.0071887251724448 | 0.00504471941925951 | 10248 | tags=39%, list=25%, signal=29% | IL1B/RELB/MAP3K8/DDIT3/NFKB2/RAC1/GADD45G/DUSP5/GADD45B/FLNC/HSPA2/IL1A/DUSP2/JUN/JUND/MAP3K11/MAP2K3/TGFB1/FGF8/CACNA2D1/DUSP3/GRB2/RELA/DUSP1/FGF5/MAP2K2/MAP3K6/HSPB1/NR4A1/PLA2G2A/PRKCG/MAPKAPK2/PLA2G4A/CACNA1G/PLA2G5/NGF/CACNG1/DUSP8/FGF7/TNF/CACNA1S/ATF4/FGF1/NFKB1/MAPK3/FGF12/MRAS/CACNA1A/IKBKG/RRAS/PDGFA/MAP3K13/PAK1/PDGFRA/ARRB2/HSPA6/ECSIT/PLA2G2C/MAPK8IP1/MAP2K5/RASGRP4/CD14/HRAS/DUSP9/GNG12/RPS6KA4/PLA2G4B/CRK/PTPN5/LAMTOR3/TRAF2/CACNG3/MKNK2/CACNG7/RAC2/FGFR3/SRF/FGF14/MAP3K3/PRKACA/PDGFB/CHP1/MAPKAPK3/MAPT/STK3/AKT1/FOS/RAP1A/HSPA1B/FGFR4/MAPK11/MAPK7/MAX/TNFRSF1A/HSPA1A/RPS6KA1/DUSP4/FAS/PPP3R1 |
| KEGG_FOCAL_ADHESION | KEGG_FOCAL_ADHESION | KEGG_FOCAL_ADHESION | 197 | 0.440788125309909 | 1.4690363575213 | 0.00131882278865499 | 0.00845865650654577 | 0.00593589930283914 | 9684 | tags=35%, list=23%, signal=27% | MYL7/THBS1/LAMB3/ITGA11/RAC1/FLNC/THBS2/JUN/PARVA/COL11A1/ACTN2/COL1A2/GRB2/FN1/COL3A1/COL1A1/COL5A2/KDR/MYL12A/MYL2/MYL12B/PRKCG/TNC/COL5A1/COL6A3/SPP1/SRC/COL6A1/ITGB6/IBSP/MAPK3/MYL9/PDGFA/BAD/PAK1/PDGFRA/VAV1/CCND1/FLT1/COL4A1/LAMA4/VEGFA/PAK4/VEGFB/ZYX/ACTB/HRAS/CHAD/CAV1/PARVG/SHC3/VEGFD/CRK/MYL5/RHOA/PPP1CA/MYLK2/RAC2/TNN/TNXB/ILK/ITGA2B/PDGFB/VASP/LAMB2/COL4A2/ACTG1/AKT1/RAP1A |
| KEGG_AXON_GUIDANCE | KEGG_AXON_GUIDANCE | KEGG_AXON_GUIDANCE | 128 | 0.470858317689976 | 1.53802130314243 | 0.0015433439595021 | 0.00956873254891305 | 0.00671490003432495 | 9784 | tags=41%, list=23%, signal=32% | SEMA6B/UNC5A/CXCL12/RAC1/RHOD/DPYSL2/UNC5D/PLXNB2/SEMA3B/EFNA2/EPHA3/LIMK1/DPYSL5/SLIT1/SLIT3/MAPK3/GNAI2/PAK1/EPHA7/EFNA1/UNC5C/SRGAP1/SEMA3D/PAK4/CDK5/SEMA6D/EFNA4/HRAS/LIMK2/FES/EPHA8/LRRC4C/RHOA/PLXNB3/SEMA3A/RAC2/SEMA4B/EFNA3/NFATC1/ABLIM3/EPHB2/CFL1/EPHB4/EPHB6/NRP1/CHP1/ABLIM2/EPHB1/SEMA5A/SRGAP2/NGEF/SEMA4A/CXCR4 |
| KEGG_VIBRIO_CHOLERAE_INFECTION | KEGG_VIBRIO_CHOLERAE_INFECTION | KEGG_VIBRIO_CHOLERAE_INFECTION | 54 | 0.554878751863215 | 1.70523420049405 | 0.00188812956722936 | 0.0113287774033761 | 0.00795001923043939 | 7086 | tags=43%, list=17%, signal=35% | TCIRG1/KDELR3/ATP6V1D/KCNQ1/PRKCG/ATP6V0E1/ATP6V1G1/ATP6V0B/ATP6V1F/SEC61B/SEC61G/ARF1/ATP6V0D1/ATP6V1H/TJP1/ATP6V1E1/ATP6V1B2/ATP6AP1/ATP6V0C/KDELR1/ACTB/KDELR2/TJP2 |
| KEGG_ENDOCYTOSIS | KEGG_ENDOCYTOSIS | KEGG_ENDOCYTOSIS | 181 | 0.435884019309303 | 1.44656944924666 | 0.00239596561471359 | 0.0139265501355228 | 0.00977301763896334 | 11703 | tags=50%, list=28%, signal=36% | HSPA2/HLA-B/CHMP4B/CHMP4A/CHMP2A/SH3GL1/SNF8/CHMP1B/KDR/EHD1/IL2RG/MVB12A/ARFGAP1/HLA-F/HLA-A/VPS37B/SRC/LDLR/CLTB/PLD2/HLA-E/CLTA/EPN1/EHD4/RAB11B/VPS28/AP2S1/PDGFRA/ARRB2/HGS/PSD/HSPA6/CHMP5/SH3GL3/RUFY1/FLT1/ARAP1/HLA-C/GRK3/CHMP2B/RAB5C/GRK2/SH3GL2/HRAS/SH3GLB2/MVB12B/VPS25/AP2M1/CBLC/ARF6/ACAP1/CSF1R/DNM2/FGFR3/ERBB3/VPS4A/SMURF1/TSG101/PARD6A/CHMP6/PARD3/RAB11FIP1/GIT2/SH3GLB1/GRK5/HSPA1B/DAB2/ACAP3/CXCR4/FGFR4/VPS4B/VPS37C/AP2A2/GRK6/HSPA1A/ERBB4/GRK1/CDC42/HLA-G/SMAP1/HSPA8/IQSEC2/CHMP3/SH3KBP1/ASAP3/RAB11A/CHMP4C/HSPA1L/RAB5A/EHD2 |
| KEGG_ARGININE_AND_PROLINE_METABOLISM | KEGG_ARGININE_AND_PROLINE_METABOLISM | KEGG_ARGININE_AND_PROLINE_METABOLISM | 54 | 0.549196185487985 | 1.68777073393116 | 0.00248359291659328 | 0.0139984328026167 | 0.00982346161587135 | 12027 | tags=52%, list=29%, signal=37% | ARG2/NOS1/LAP3/AOC1/ALDH2/NOS2/SAT1/CKM/SAT2/P4HA2/MAOB/CKMT1B/SMS/CKMT2/ALDH4A1/CKB/ASL/SRM/NAGS/CPS1/OTC/CKMT1A/ASS1/ACY1/NOS3/GLUL/OAT/P4HA3 |
| KEGG_PENTOSE_PHOSPHATE_PATHWAY | KEGG_PENTOSE_PHOSPHATE_PATHWAY | KEGG_PENTOSE_PHOSPHATE_PATHWAY | 26 | 0.626365122227404 | 1.71676218403398 | 0.00256398877640222 | 0.0140265268356122 | 0.00984317672674537 | 9880 | tags=54%, list=24%, signal=41% | RBKS/TKTL2/PGLS/TALDO1/ALDOA/FBP1/TKTL1/PFKL/PRPS2/ALDOC/DERA/G6PD/TKT/ALDOB |
| KEGG_TRYPTOPHAN_METABOLISM | KEGG_TRYPTOPHAN_METABOLISM | KEGG_TRYPTOPHAN_METABOLISM | 40 | 0.583359521421086 | 1.70059542388694 | 0.00305509458089415 | 0.0162356454870375 | 0.0113934354295 | 5611 | tags=32%, list=13%, signal=28% | CYP1A1/IL4I1/KYNU/AOC1/ALDH2/CYP1B1/ECHS1/WARS1/HAAO/MAOB/ACAT2/AANAT/TDO2 |
| KEGG_ALLOGRAFT_REJECTION | KEGG_ALLOGRAFT_REJECTION | KEGG_ALLOGRAFT_REJECTION | 35 | 0.590717689904897 | 1.69419287270095 | 0.00322320054148089 | 0.0166532027976513 | 0.0116864581036149 | 7436 | tags=49%, list=18%, signal=40% | IL12B/HLA-B/HLA-F/HLA-A/CD86/HLA-E/HLA-DMA/TNF/HLA-DRA/IL10/HLA-C/CD40/HLA-DQA2/HLA-DPB1/HLA-DMB/IL5/IFNG |
| KEGG_BLADDER_CANCER | KEGG_BLADDER_CANCER | KEGG_BLADDER_CANCER | 42 | 0.551785839208123 | 1.62208398687454 | 0.00365804670938329 | 0.0179051759985603 | 0.0125650357884634 | 7141 | tags=33%, list=17%, signal=28% | THBS1/CDKN1A/TYMP/CXCL8/DAPK3/MMP2/MAP2K2/MAPK3/ARAF/CCND1/VEGFA/VEGFB/HRAS/VEGFD |
| KEGG_RIG_I_LIKE_RECEPTOR_SIGNALING_PATHWAY | KEGG_RIG_I_LIKE_RECEPTOR_SIGNALING_PATHWAY | KEGG_RIG_I_LIKE_RECEPTOR_SIGNALING_PATHWAY | 63 | 0.517814426260199 | 1.60786682874948 | 0.00357082624615355 | 0.0179051759985603 | 0.0125650357884634 | 4597 | tags=29%, list=11%, signal=25% | IFNA1/IL12B/CXCL8/NFKBIA/NFKBIB/CXCL10/IRF7/OTUD5/RELA/IKBKE/IFIH1/ISG15/TNF/DHX58/NFKB1/TRADD/IKBKG/TANK |
| KEGG_INTESTINAL_IMMUNE_NETWORK_FOR_IGA_PRODUCTION | KEGG_INTESTINAL_IMMUNE_NETWORK_FOR_IGA_PRODUCTION | KEGG_INTESTINAL_IMMUNE_NETWORK_FOR_IGA_PRODUCTION | 46 | 0.55370675682944 | 1.65132499953744 | 0.003895212743893 | 0.0185771684708743 | 0.0130366094532451 | 7177 | tags=39%, list=17%, signal=32% | IL15RA/CXCL12/IL6/IL15/TGFB1/LTBR/CD86/HLA-DMA/HLA-DRA/IL10/PIGR/TNFSF13B/CD40/HLA-DQA2/HLA-DPB1/HLA-DMB/IL5/TNFSF13 |
| KEGG_LEUKOCYTE_TRANSENDOTHELIAL_MIGRATION | KEGG_LEUKOCYTE_TRANSENDOTHELIAL_MIGRATION | KEGG_LEUKOCYTE_TRANSENDOTHELIAL_MIGRATION | 112 | 0.461845041126794 | 1.49702752794891 | 0.00429149925172026 | 0.0199554715204992 | 0.0140038396635082 | 10108 | tags=40%, list=24%, signal=31% | MYL7/ICAM1/NCF1/CXCL12/RAC1/CLDN16/CLDN1/CLDN7/ACTN2/MMP2/MYL12A/MYL2/MYL12B/PRKCG/CDH5/JAM2/CLDN11/CYBA/CLDN18/SIPA1/MYL9/GNAI2/ITGB2/VAV1/THY1/RAPGEF4/CLDN2/ACTB/NCF2/ESAM/MYL5/RHOA/NCF4/RAC2/CTNNA3/VASP/CTNNA2/ACTG1/ITGAM/RAP1A/CXCR4/CYBB/MAPK11/CLDN5/VCAM1 |
| KEGG_CALCIUM_SIGNALING_PATHWAY | KEGG_CALCIUM_SIGNALING_PATHWAY | KEGG_CALCIUM_SIGNALING_PATHWAY | 174 | 0.431587120824259 | 1.43217870125551 | 0.00465693484845877 | 0.021126582483252 | 0.0148256719180716 | 9889 | tags=36%, list=24%, signal=27% | TACR3/NOS1/HTR5A/SPHK1/ADORA2B/ADRA1A/BDKRB2/GRIN2D/P2RX4/ADORA2A/NOS2/PLN/PRKCG/VDAC2/HTR7/PDE1C/NTSR1/CACNA1G/SLC25A6/PLCB3/CALML3/TNNC2/HTR2B/PHKG1/ATP2A1/CACNA1S/AGTR1/CACNA1A/PDGFRA/TNNC1/PTGFR/VDAC2P5/GNA15/SLC8A2/GNA11/CAMK2A/CALM3/CHRM1/PHKG2/PPID/DRD1/PDE1A/GRPR/SLC25A5/PDE1B/EDNRA/TBXA2R/BDKRB1/MYLK2/SLC8A1/ERBB3/SPHK2/PRKACA/SLC8A3/CHP1/BST1/ATP2B2/PTAFR/RYR1/GRIN2A/P2RX6/ADCY1 |
| KEGG_NATURAL_KILLER_CELL_MEDIATED_CYTOTOXICITY | KEGG_NATURAL_KILLER_CELL_MEDIATED_CYTOTOXICITY | KEGG_NATURAL_KILLER_CELL_MEDIATED_CYTOTOXICITY | 123 | 0.458781579681007 | 1.49773923842475 | 0.00502030176443173 | 0.0222327649567691 | 0.0156019403205397 | 7462 | tags=30%, list=18%, signal=25% | ULBP2/IFNA1/ULBP1/ICAM1/RAC1/BID/HLA-B/FCER1G/IFNGR2/CD48/SH3BP2/RAET1L/GRB2/MAP2K2/PRKCG/HLA-A/HLA-E/TYROBP/TNF/ICAM2/MAPK3/TNFRSF10D/PAK1/HCST/ITGB2/ARAF/VAV1/HLA-C/LCP2/PTPN6/HRAS/TNFSF10/SHC3/KIR2DL4/IFNG/FCGR3A/TNFRSF10B |
| KEGG_PPAR_SIGNALING_PATHWAY | KEGG_PPAR_SIGNALING_PATHWAY | KEGG_PPAR_SIGNALING_PATHWAY | 67 | 0.500082731390833 | 1.56302434889537 | 0.00584351712681525 | 0.0252766089671543 | 0.0177379712050206 | 6468 | tags=27%, list=15%, signal=23% | UCP1/UBC/CPT1B/CYP27A1/ACAA1/NR1H3/HMGCS2/FABP1/CD36/PPARG/CPT1C/ACOX2/RXRA/PCK2/DBI/SLC27A1/ME1/ACSL3 |
| KEGG_OTHER_GLYCAN_DEGRADATION | KEGG_OTHER_GLYCAN_DEGRADATION | KEGG_OTHER_GLYCAN_DEGRADATION | 14 | 0.679027003841288 | 1.65087054545911 | 0.00602865896723854 | 0.0254847856342356 | 0.0178840600942004 | 8651 | tags=64%, list=21%, signal=51% | FUCA1/NEU1/HEXB/MAN2B1/NEU4/MANBA/FUCA2/MAN2C1/GLB1 |
| KEGG_FC_GAMMA_R_MEDIATED_PHAGOCYTOSIS | KEGG_FC_GAMMA_R_MEDIATED_PHAGOCYTOSIS | KEGG_FC_GAMMA_R_MEDIATED_PHAGOCYTOSIS | 96 | 0.464117869918399 | 1.49323489556829 | 0.00854245930842642 | 0.0353088318081625 | 0.0247781275846754 | 10586 | tags=44%, list=25%, signal=33% | SPHK1/NCF1/RAC1/ARPC1A/MARCKS/PRKCG/LIMK1/PLA2G4A/WAS/ARPC1B/PLD2/MAPK3/FCGR1A/ARPC3/PAK1/VAV1/ARPC2/FCGR2A/HCK/PRKCD/GSN/LIMK2/ARPC4/PLPP1/PLA2G4B/CRK/FCGR3A/RAC2/ARF6/RPS6KB2/LYN/DNM2/SPHK2/CFL1/VASP/AKT1/MYO10/PRKCE/CDC42/MARCKSL1/CFL2/LAT |
| KEGG_SPLICEOSOME | KEGG_SPLICEOSOME | KEGG_SPLICEOSOME | 126 | 0.435097262074392 | 1.41917824831972 | 0.0096545812222653 | 0.0390380892900293 | 0.0273951503789679 | 10307 | tags=44%, list=25%, signal=33% | EIF4A3/HSPA2/BUD31/RBM22/PCBP1/SF3B5/SRSF3/LSM6/HNRNPA1L2/SF3B6/PQBP1/TXNL4A/SART1/ISY1/ALYREF/HSPA6/LSM7/XAB2/SRSF2/TRA2A/PRPF6/SF3B4/PRPF3/SYF2/CTNNBL1/PUF60/MAGOH/BCAS2/PRPF31/SNU13/PHF5A/SNRPA/SNRPD2/TRA2B/ZMAT2/SRSF9/SF3A2/SNRPE/SNRPB/HNRNPC/SNRPG/LSM4/CDC40/CCDC12/SNRPC/SMNDC1/SNRPB2/SRSF5/HNRNPM/RBM8A/HSPA1B/LSM3/CRNKL1/HSPA1A/USP39 |
| KEGG_AMINO_SUGAR_AND_NUCLEOTIDE_SUGAR_METABOLISM | KEGG_AMINO_SUGAR_AND_NUCLEOTIDE_SUGAR_METABOLISM | KEGG_AMINO_SUGAR_AND_NUCLEOTIDE_SUGAR_METABOLISM | 43 | 0.523173587565882 | 1.54271121403441 | 0.01156589339954 | 0.045771407921584 | 0.0321202862607607 | 10056 | tags=49%, list=24%, signal=37% | NAGK/RENBP/NANS/AMDHD2/HEXB/NPL/GCK/CYB5R1/GMPPA/CYB5R3/GMDS/GALE/GALT/FCSK/GALK1/HK1/PMM1/GNPDA1/GFPT2/HK2/HK3 |
| KEGG_GRAFT_VERSUS_HOST_DISEASE | KEGG_GRAFT_VERSUS_HOST_DISEASE | KEGG_GRAFT_VERSUS_HOST_DISEASE | 37 | 0.553373223658681 | 1.59710322057673 | 0.0121556233674552 | 0.0471030405488888 | 0.0330547652974658 | 7436 | tags=43%, list=18%, signal=36% | IL1B/IL6/HLA-B/IL1A/HLA-F/HLA-A/CD86/HLA-E/HLA-DMA/TNF/HLA-DRA/HLA-C/HLA-DQA2/HLA-DPB1/HLA-DMB/IFNG |

**Table S11**

| Gene | Drugs |
| --- | --- |
| ALOX15 | (2E)-3-(2-OCT-1-YN-1-YLPHENYL)ACRYLIC ACID |
| ALOX15 | Resveratrol |
| ALOX15 | Cannabidiol |
| ALOX15 | Nabiximols |
| ALOX15 | Medical Cannabis |
| ALOX15 | Mangostin |
| ALOX15 | Kaempherol |
| CAT | Fusidic acid |
| MT2A | Cisplatin |
| MT2A | Carboplatin |
| MT2A | Oxaliplatin |
| MT2A | Zinc |
| MT2A | Zinc acetate |
| MT2A | Zinc chloride |
| MT2A | Zinc sulfate, unspecified form |

Fig S1 Expression profiles of key genes between the control group and different etiological subgroups: (A) ALOX15; (B) CAT; (C) CYGB; (D) HBZ; (E) MT2A. ns represents p>0.05, * represents p<0.05, and ** represents p<0.01.


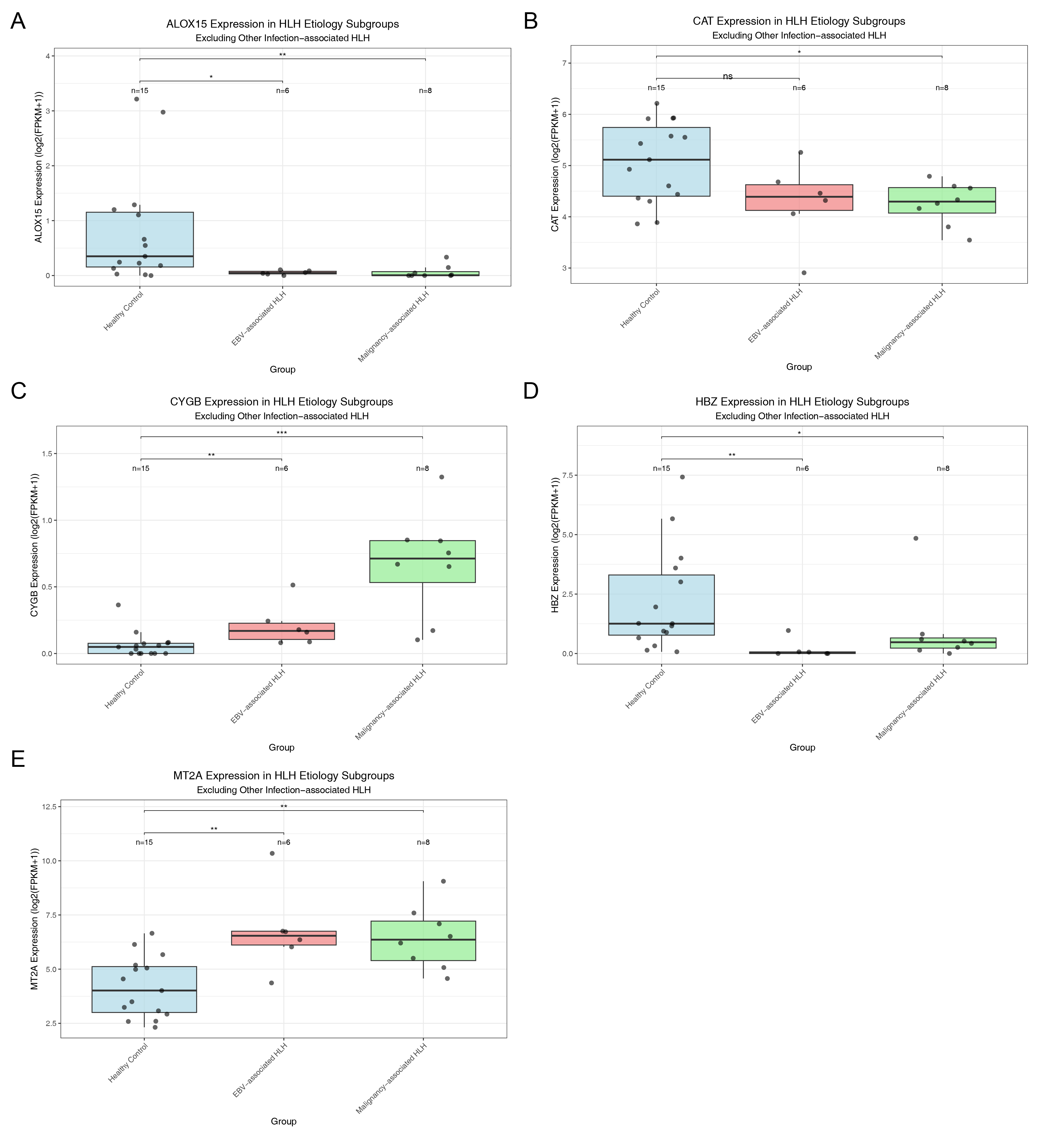

Supplement: Supplementary file 1 [file Table_1.docx]
